# Supplementary material for: Experiences of coping with the first wave of COVID-19 epidemic in Philadelphia, PA: Mixed methods analysis of a cross-sectional survey of worries and symptoms of mood disorders
Source: PLoS One. 2021 Oct 4;16(10):e0258213. doi: 10.1371/journal.pone.0258213 (PMC8489717; doi:10.1371/journal.pone.0258213)
Supplement: S1 Table — (PDF) [file pone.0258213.s001.pdf]

Table S1: Demographics of participants who did not report employment at start of the epidemic (see Table 1 for definitions of abbreviations)

Health and demongraphics		Did not have a job at start of epidemic (n=369)								
			Anxiety				Depression			
		Total	case				case			
		N	N	%	mean	sd	N	%	mean	sd
Demographics										
Gender	Female	275	84	31	8.9	4.3	33	12	6.2	3.9
	Male	88	22	25	7.4	4.4	14	16	5.7	4.4
	Other	6	3	50	13.2	5.1	2	33	9.5	3.7
Race	White	323	97	30	8.7	4.4	43	13	6.2	4
	Black or African American	21	3	14	6.2	3.6	0	0	4.7	2.8
	Other	25	9	36	8.9	4.6	6	24	7	5.1
Age (years)	<35	26	14	54	11.3	4.9	7	27	7.5	4.6
	35-54	73	36	48	10.8	4.7	18	25	7.8	4.5
	55+	270	60	22	7.7	3.9	24	9	5.6	3.7
Personal income in 2019	<40,000	124	53	43	9.9	4.9	26	21	7.3	4.4
	40,000-<100,000	140	34	24	8.2	4	16	11	5.8	3.8
	100,000+	86	15	17	7.4	3.6	4	5	5.2	3.6
	missing	19	7	37	8.6	5	3	16	5.9	4.3
Education	college degree	272	72	26	8.4	4.1	36	13	6.1	4
	no college degree	94	37	39	9.2	5.1	13	14	6.6	4.3
	missing	3	0	0	3.3	3.1	0	0	1.3	0.6
Marital status	Married, or living as married	208	64	31	8.8	4.2	25	12	6.0	4.0
	Single	79	29	37	9	5.3	16	20	6.8	4.7
	Widowed, divorced	81	16	20	7.7	3.8	8	10	5.9	3.5
	missing	1	0	0			0	0		
Children <18 years living in your household	Yes	54	22	41	9.7	4.8	12	22	7.1	4.5
	No	315	87	28	8.4	4.3	37	12	6.0	4.0
	missing	0								
Health										
Were unwell for two or more consecutive days?	Yes	79	33	42	9.8	4.1	13	16	7.1	3.6
	No	288	75	26	8.3	4.4	35	12	5.9	4.2
	missing	2	1	50	9.0	7.1	1	50	8.5	4.9
Believe infected	Yes	3	1	33	8.3	4.2	2	67	10.3	2.1
	No	31	14	45	10.9	4.0	11	35	8.5	5.1
	maybe	10	7	70	11.5	4.6	3	30	8.7	3.8
	missing	325	87	27	8.3	4.3	33	10	5.8	3.9
COVID-19 diagnosis	Reported									
	Not reported									
Self-rated health compared to others	good to excellent	317	81	26	8.2	4.2	34	11	5.9	4.0
	poor or fair	52	28	54	11.2	4.7	15	29	8.1	4.1
Self-rated health	good to excellent	327	90	28	8.3	4.3	40	12	6.0	4.1
	poor or fair	42	19	45	10.7	4.6	9	21	7.4	4.0
Upset by experience people trying to avoid you in the public places										
Yes		23	8	35	9.8	4.0	5	22	8.2	4.2
No		232	67	29	8.6	4.3	29	13	6.0	4.0
Do not want to answer		3	2	67	9.7	9.1	0	0	5.7	3.2
Maybe avoided by other		69	21	30	9.0	4.4	9	13	6.6	4.0
Did not experience		105	31	30	8.3	4.6	15	14	6.2	4.3
Phase of restrictions during interview	Most restrictive (early)	179	46	26	8.0	4.3	20	11	5.5	4.2
	Restrictions relaxed (late)	190	63	33	9.2	4.4	29	15	6.8	3.8
