# Supplementary material for: Experiences of coping with the first wave of COVID-19 epidemic in Philadelphia, PA: Mixed methods analysis of a cross-sectional survey of worries and symptoms of mood disorders
Source: PLoS One. 2021 Oct 4;16(10):e0258213. doi: 10.1371/journal.pone.0258213 (PMC8489717; doi:10.1371/journal.pone.0258213)
Supplement: S3 Table — (PDF) [file pone.0258213.s003.pdf]

Table S3: Correlations of support, worries and themes of most difficult experiences with anxiety and depression scores for those who did not report employment at the start of epidemic see Table 2 for definitions of abbreviations)

	Did not have a job at start of epidemic (n=369)						
	**Mean**	**SD**	**Median**	**rank correlation (p-value)**			
				**HADS Anx**		**HADS Dep**	
Where you will find support (no support at all =0, very strong support=100)							
My immediate family	80	28	92	-0.04	0.43	-0.19	0.0002
My doctor	50	29	45	0.02	0.72	-0.12	0.02
Federal Government	23	20	23	0.10	0.06	0.09	0.09
City of Philadelphia	35	23	36	0.11	0.03	0.03	0.54
Department of Public Health (City)	39	25	39	0.05	0.34	-0.04	0.44
My religious community	40	28	38	0.02	0.67	-0.06	0.24
Social services organization	25	19	26	0.13	0.01	0.11	0.03
My neighbors	44	27	42	-0.07	0.19	-0.22	<.0001
Other	62	20	64	-0.05	0.37	-0.10	0.05
Worries about the COVID-19 epidemic (not at all worried=0, very worried=100)							
I will be infected	65	26	70	0.24	<.0001	0.17	0.001
I will infect my family	59	31	61	0.22	<.0001	0.12	0.03
I will not be able to cope with the work	33	26	37	0.28	<.0001	0.19	0.00
I will become poor	38	29	40	0.30	<.0001	0.23	<.0001
I will be short of food	31	25	31	0.29	<.0001	0.20	0.0001
I will be short of medicines	32	27	31	0.30	<.0001	0.22	<.0001
I will fail myself and my family	39	30	40	0.41	<.0001	0.27	<.0001
I will be confined at home and not able to leave	52	30	48	0.32	<.0001	0.34	<.0001
Themes of most difficult experiences	**N**	**%**		**biserial correlation (p-value)**			
change in work: self or family	5	1.4		0.10	0.06	0.04	0.4
change in schooling	0						
childcare	4	1.1		0.004	0.9	0.02	0.8
lost income (self or family)	15	4.1		0.06	0.2	0.11	0.0
unavoidable proximity to strangers/ fear of infection	16	4.3		0.07	0.2	0.05	0.4
social isolation; cabin fever	14	3.8		-0.01	0.9	0.04	0.4
poor health self or others	19	5.1		0.05	0.3	0.06	0.3
trouble sleeping	2	0.5		0.04	0.4	0.10	0.06
uncertainty	9	2.4		0.00	1.0	0.06	0.2
media coverage	3	0.8		0.04	0.5	0.01	0.8
food access	2	0.5		-0.03	0.6	-0.003	1.0
death family or friend	6	1.6		0.02	0.7	-0.01	0.9
anger	5	1.4		0.0003	1.0	0.05	0.4
