# Supplementary material for: Experiences of coping with the first wave of COVID-19 epidemic in Philadelphia, PA: Mixed methods analysis of a cross-sectional survey of worries and symptoms of mood disorders
Source: PLoS One. 2021 Oct 4;16(10):e0258213. doi: 10.1371/journal.pone.0258213 (PMC8489717; doi:10.1371/journal.pone.0258213)

The FACTOR Procedure

**Input Data Type**	Raw Data
**Number of Records Read**	1293
**Number of Records Used**	1293
**N for Significance Tests**	1293

The FACTOR Procedure
Initial Factor Method: Maximum Likelihood

Prior Communality Estimates: SMC					
AscoreR	DscoreR	worries_self_infected	worries_family_infected	worries_unabletocope	worries_poor
0.48622444	0.42537210	0.30193902	0.28013531	0.27828462	0.44434293

worries_nofood	worries_nomedicine	worries_failfamily	worries_beingconfined
0.53593165	0.48757861	0.39987123	0.16462367

Preliminary Eigenvalues: Total = 6.66332844 Average = 0.66633284				
	Eigenvalue	Difference	Proportion	Cumulative
1	5.83386741	4.60863694	0.8755	0.8755
2	1.22523047	0.48000954	0.1839	1.0594
3	0.74522093	0.56643725	0.1118	1.1712
4	0.17878368	0.14188270	0.0268	1.1981
5	0.03690098	0.13801566	0.0055	1.2036
6	-.10111468	0.10140472	-0.0152	1.1884
7	-.20251940	0.09628210	-0.0304	1.1580
8	-.29880150	0.03572714	-0.0448	1.1132
9	-.33452864	0.08518219	-0.0502	1.0630
10	-.41971082		-0.0630	1.0000

3 factors will be retained by the NFACTOR criterion.

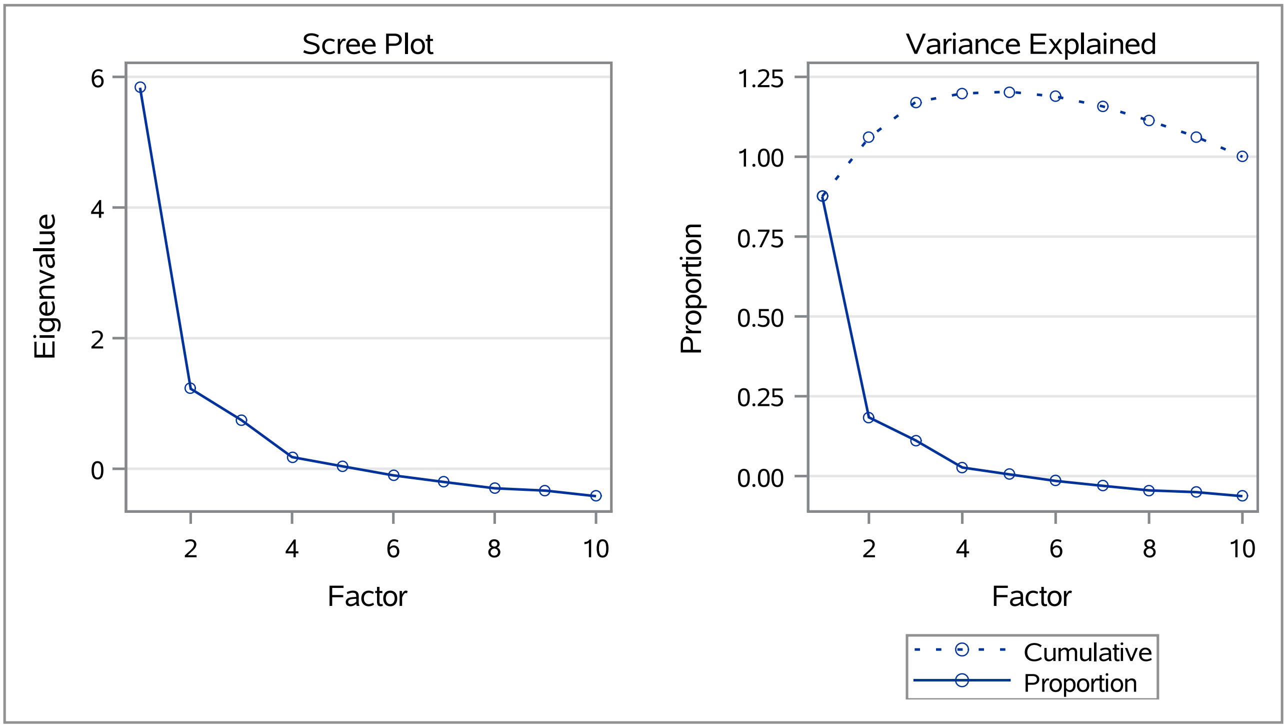

The FACTOR Procedure
Initial Factor Method: Maximum Likelihood

Iteration	Criterion	Ridge	Change	Communalities									
1	0.1091235	0.0000	0.3226	0.77221	0.50807	0.39587	0.60272	0.30293	0.50821	0.66992	0.57474	0.42417	0.17135
2	0.1059292	0.0000	0.1182	0.76747	0.53960	0.35725	0.72097	0.29553	0.50028	0.67547	0.59046	0.41403	0.16733
3	0.1057251	0.0000	0.0412	0.76994	0.53854	0.35256	0.76216	0.29286	0.49711	0.67822	0.59185	0.41253	0.16751
4	0.1057188	0.0000	0.0076	0.76807	0.54005	0.35125	0.76979	0.29212	0.49671	0.67799	0.59283	0.41219	0.16753
5	0.1057186	0.0000	0.0016	0.76842	0.53978	0.35086	0.77138	0.29200	0.49653	0.67818	0.59279	0.41214	0.16757
6	0.1057186	0.0000	0.0002	0.76826	0.53990	0.35084	0.77149	0.29197	0.49652	0.67814	0.59286	0.41213	0.16757

Convergence criterion satisfied.

Significance Tests Based on 1293 Observations			
Test	DF	Chi-Square	Pr > ChiSq
H0: No common factors	45	4003.4672	<.0001
HA: At least one common factor			
H0: 3 Factors are sufficient	18	135.9365	<.0001
HA: More factors are needed			

Chi-Square without Bartlett's Correction	136.58839
Akaike's Information Criterion	100.58839
Schwarz's Bayesian Criterion	7.62343
Tucker and Lewis's Reliability Coefficient	0.92552

Squared Canonical Correlations		
Factor1	Factor2	Factor3
0.89740143	0.75051472	0.71544285

Eigenvalues of the Weighted Reduced Correlation Matrix: Total = 14.2692095 Average = 1.42692095				
	Eigenvalue	Difference	Proportion	Cumulative
1	8.74672441	5.73847183	0.6130	0.6130
2	3.00825257	0.49402000	0.2108	0.8238
3	2.51423257	2.19075331	0.1762	1.0000
4	0.32347927	0.19729822	0.0227	1.0227
5	0.12618104	0.10259475	0.0088	1.0315
6	0.02358629	0.02001756	0.0017	1.0332
7	0.00356873	0.07555029	0.0003	1.0334

The FACTOR Procedure
Initial Factor Method: Maximum Likelihood

Eigenvalues of the Weighted Reduced Correlation Matrix: Total = 14.2692095 Average = 1.42692095				
	Eigenvalue	Difference	Proportion	Cumulative
**8**	-.07198156	0.07612453	-0.0050	1.0284
**9**	-.14810610	0.10862163	-0.0104	1.0180
**10**	-.25672773		-0.0180	1.0000

Factor Pattern				
		Factor1	Factor2	Factor3
**AscoreR**	anxiety	0.69131	-0.32025	-0.43335
**worries_nofood**	short of food	0.68896	-0.10226	0.43934
**worries_nomedicine**	short of medicines	0.65201	-0.01649	0.40922
**worries_poor**	become poor	0.63052	-0.08491	0.30290
**worries_failfamily**	fail myself/family	0.62467	-0.05357	0.13800
**DscoreR**	depression	0.54749	-0.33226	-0.36023
**worries_unabletocope**	cope with work	0.53532	0.01828	0.07121
**worries_self_infected**	infected	0.47096	0.35747	-0.03535
**worries_beingconfined**	confined at home	0.39756	-0.08127	0.05402
**worries_family_infected**	infect family	0.50688	0.68746	-0.20484

Variance Explained by Each Factor		
Factor	Weighted	Unweighted
**Factor1**	8.74672441	3.38857184
**Factor2**	3.00825257	0.84108412
**Factor3**	2.51423257	0.84002774

Final Communality Estimates and Variable Weights		
Total Communality: Weighted = 14.269210 Unweighted = 5.069684		
Variable	Communality	Weight
**AscoreR**	0.76825829	4.31513939
**DscoreR**	0.53990028	2.17345357
**worries_self_infected**	0.35084345	1.54045811
**worries_family_infected**	0.77148834	4.37613942
**worries_unabletocope**	0.29197055	1.41236970
**worries_poor**	0.49651841	1.98619192
**worries_nofood**	0.67814680	3.10695904
**worries_nomedicine**	0.59285370	2.45613892

The FACTOR Procedure
Initial Factor Method: Maximum Likelihood

Final Communality Estimates and Variable Weights		
Total Communality: Weighted = 14.269210 Unweighted = 5.069684		
Variable	Communality	Weight
worries_failfamily	0.41212954	1.70105574
worries_beingconfined	0.16757435	1.20130368

The FACTOR Procedure
Rotation Method: Oblique Varimax

Oblique Transformation Matrix			
	1	2	3
1	0.44125	0.47428	0.38162
2	-0.25774	-0.64914	1.00739
3	1.03803	-0.79762	-0.26785

Inter-Factor Correlations			
	Factor1	Factor2	Factor3
Factor1	1.00000	0.43086	0.39037
Factor2	0.43086	1.00000	0.33956
Factor3	0.39037	0.33956	1.00000

Rotated Factor Pattern (Standardized Regression Coefficients)				
		Factor1	Factor2	Factor3
worries_nofood	short of food	0.78641	0.04272	0.04222
worries_nomedicine	short of medicines	0.71674	-0.00646	0.12259
worries_poor	become poor	0.61452	0.11257	0.07395
worries_failfamily	fail myself/family	0.43269	0.22097	0.14746
worries_unabletocope	cope with work	0.30542	0.18522	0.20363
worries_beingconfined	confined at home	0.25244	0.19823	0.05537
AscoreR	anxiety	-0.06224	0.88141	0.05728
DscoreR	depression	-0.04671	0.76267	-0.02929
worries_family_infected	infect family	-0.16615	-0.04246	0.94084
worries_self_infected	infected	0.07898	0.01951	0.54931

Reference Axis Correlations			
	Factor1	Factor2	Factor3
Factor1	1.00000	-0.34448	-0.28754
Factor2	-0.34448	1.00000	-0.20626
Factor3	-0.28754	-0.20626	1.00000

The FACTOR Procedure
Rotation Method: Oblique Varimax

Reference Structure (Semipartial Correlations)				
		**Factor1**	**Factor2**	**Factor3**
**worries_nofood**	short of food	0.67970	0.03772	0.03804
**worries_nomedicine**	short of medicines	0.61948	-0.00570	0.11044
**worries_poor**	become poor	0.53114	0.09940	0.06662
**worries_failfamily**	fail myself/family	0.37398	0.19512	0.13284
**worries_unabletocope**	cope with work	0.26398	0.16355	0.18344
**worries_beingconfined**	confined at home	0.21819	0.17504	0.04988
**AscoreR**	anxiety	-0.05380	0.77829	0.05160
**DscoreR**	depression	-0.04037	0.67345	-0.02639
**worries_family_infected**	infect family	-0.14361	-0.03749	0.84757
**worries_self_infected**	infected	0.06827	0.01723	0.49485

Variance Explained by Each Factor Eliminating Other Factors		
**Factor**	**Weighted**	**Unweighted**
**Factor1**	3.44525563	1.41481649
**Factor2**	3.76967338	1.16777053
**Factor3**	3.65770376	1.03847241

Factor Structure (Correlations)				
		**Factor1**	**Factor2**	**Factor3**
**worries_nofood**	short of food	0.82130	0.39589	0.36372
**worries_nomedicine**	short of medicines	0.76181	0.34398	0.40019
**worries_poor**	become poor	0.69189	0.40245	0.35206
**worries_failfamily**	fail myself/family	0.58546	0.45747	0.39140
**worries_unabletocope**	cope with work	0.46471	0.38596	0.38575
**worries_beingconfined**	confined at home	0.35946	0.32580	0.22123
**AscoreR**	anxiety	0.33988	0.87404	0.33227
**DscoreR**	depression	0.27046	0.73260	0.21145
**worries_family_infected**	infect family	0.18282	0.20542	0.86156
**worries_self_infected**	infected	0.30183	0.24007	0.58677

The FACTOR Procedure
Rotation Method: Oblique Varimax

Variance Explained by Each Factor Ignoring Other Factors		
**Factor**	**Weighted**	**Unweighted**
**Factor1**	6.45933739	2.73472163
**Factor2**	6.52960050	2.30187105
**Factor3**	5.93240587	2.00902406

Final Communality Estimates and Variable Weights		
**Total Communality: Weighted = 14.269210 Unweighted = 5.069684**		
**Variable**	**Communality**	**Weight**
**AscoreR**	0.76825829	4.31513939
**DscoreR**	0.53990028	2.17345357
**worries_self_infected**	0.35084345	1.54045811
**worries_family_infected**	0.77148834	4.37613942
**worries_unabletocope**	0.29197055	1.41236970
**worries_poor**	0.49651841	1.98619192
**worries_nofood**	0.67814680	3.10695904
**worries_nomedicine**	0.59285370	2.45613892
**worries_failfamily**	0.41212954	1.70105574
**worries_beingconfined**	0.16757435	1.20130368

The FACTOR Procedure
Rotation Method: Oblique Varimax

Scoring Coefficients Estimated by Regression

Squared Multiple Correlations of the Variables with Each Factor		
Factor1	Factor2	Factor3
0.83105002	0.83628207	0.82496420

Standardized Scoring Coefficients				
		Factor1	Factor2	Factor3
**worries_nofood**	short of food	0.41503	0.03610	0.05076
**worries_nomedicine**	short of medicines	0.30070	0.00846	0.07235
**worries_poor**	become poor	0.20913	0.04406	0.04013
**worries_failfamily**	fail myself/family	0.12930	0.06659	0.05342
**worries_unabletocope**	cope with work	0.07759	0.04627	0.05606
**worries_beingconfined**	confined at home	0.05334	0.04094	0.01585
**AscoreR**	anxiety	-0.02095	0.62200	0.05320
**DscoreR**	depression	-0.00842	0.27059	-0.00664
**worries_family_infected**	infect family	-0.07783	-0.02055	0.71190
**worries_self_infected**	infected	0.03020	0.00850	0.14958

The FACTOR Procedure
Rotation Method: Oblique Varimax

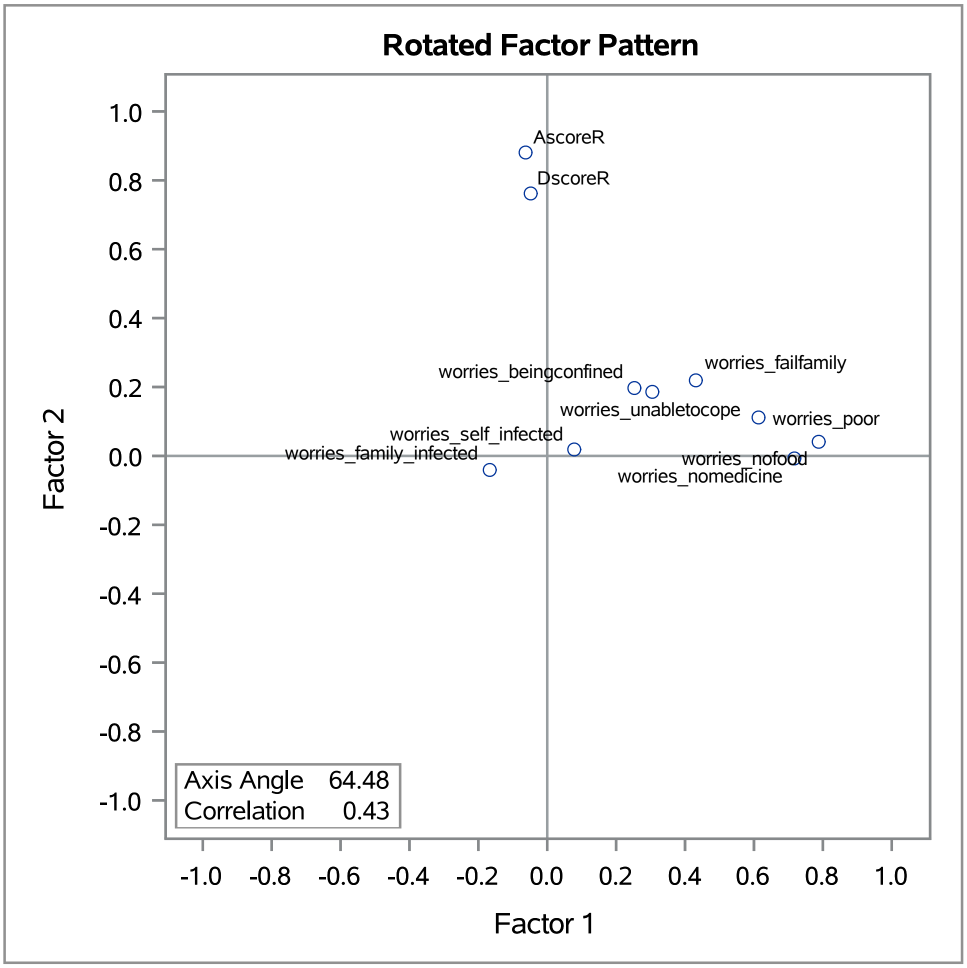

The FACTOR Procedure
Rotation Method: Oblique Varimax

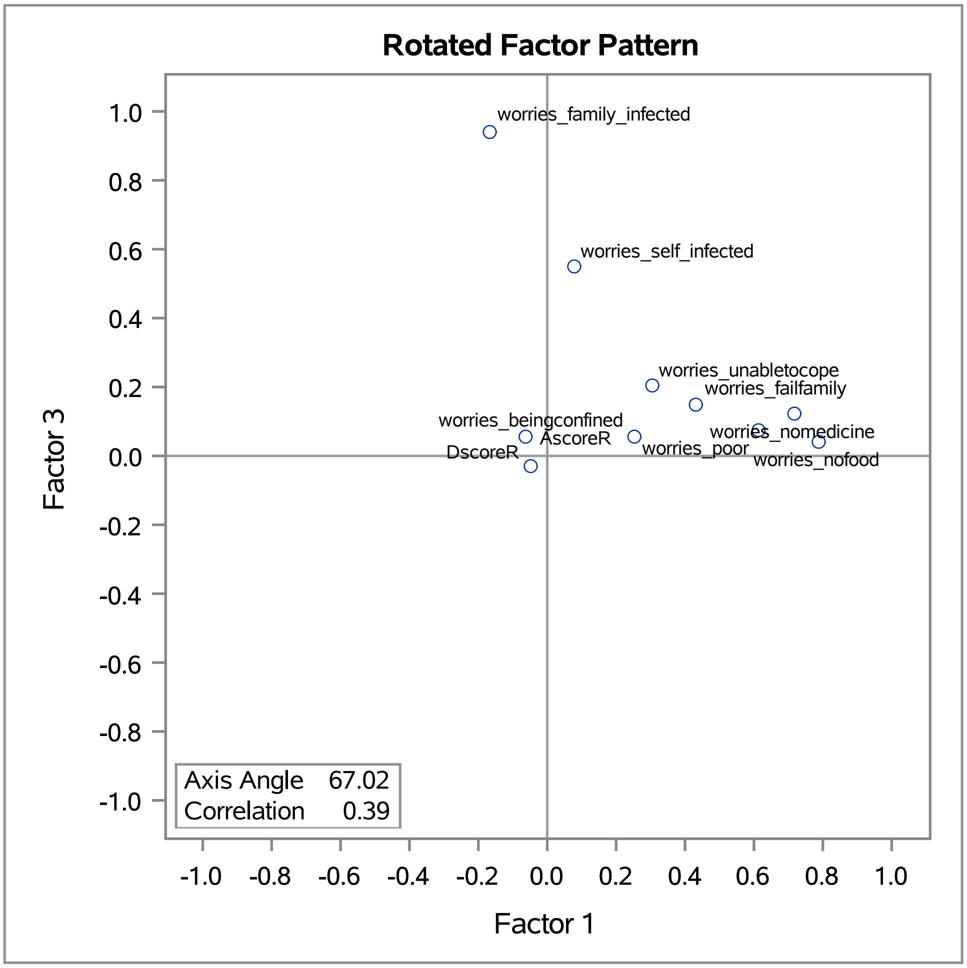

The FACTOR Procedure
Rotation Method: Oblique Varimax

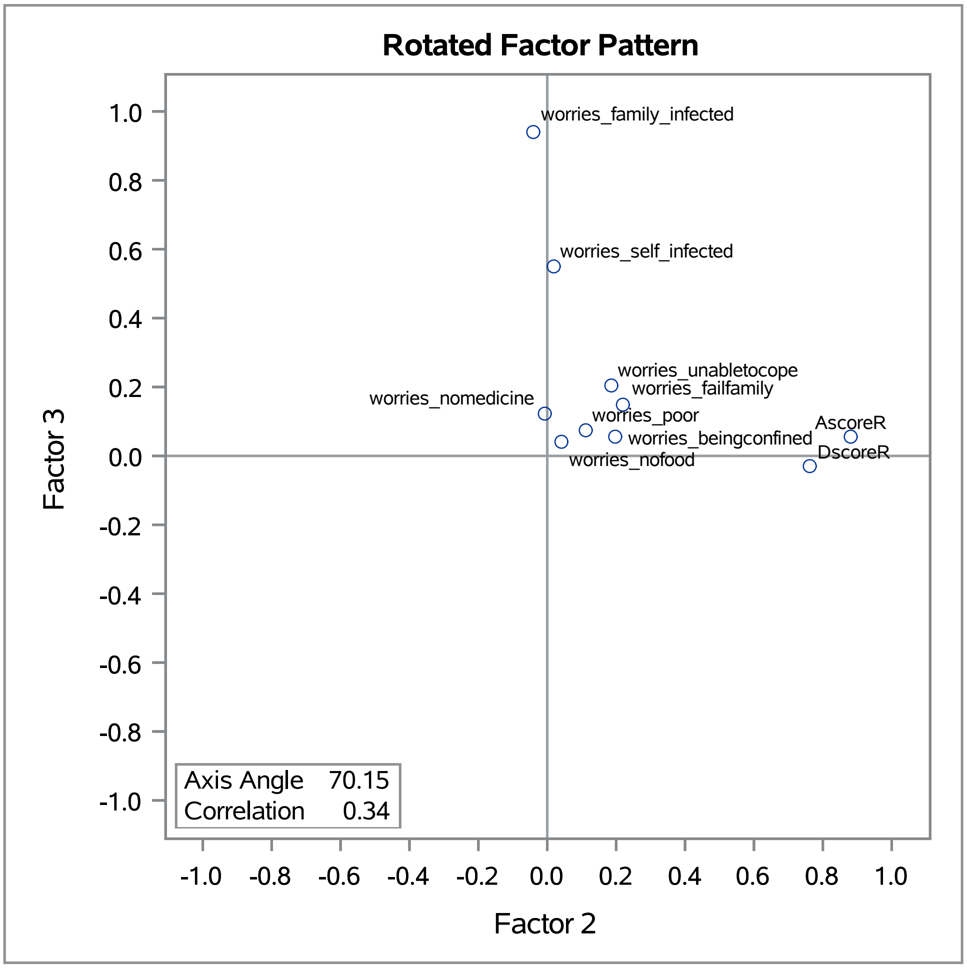

Supplement: S4 Table — (PDF) [file pone.0258213.s004.pdf]
