# Supplementary material for: Experiences of coping with the first wave of COVID-19 epidemic in Philadelphia, PA: Mixed methods analysis of a cross-sectional survey of worries and symptoms of mood disorders
Source: PLoS One. 2021 Oct 4;16(10):e0258213. doi: 10.1371/journal.pone.0258213 (PMC8489717; doi:10.1371/journal.pone.0258213)
Supplement: S5 Table — (PDF) [file pone.0258213.s005.pdf]

The definitions of x variables (x1 to x14) in SAS outputs that code themes as present=1 or absent=0.

```
x1="change in work: self or family"
x2="change in schooling"
x3="childcare"
x4="lost income: self or family"
x5="unavoidable proximity to strangers/fear infection"
x6="social isolation"
x7="return to work fear"
x8="poor health: self or others"
x9="trouble_sleeping"
x10="uncertainty"
x11="media coverage"
x12="food_access"
x13="death: family or friend"
x14="anger"
```

Fully adjusted models of Anxietry score with gender*theme interaction

The GENMOD Procedure

Model Information		
**Data Set**	WORK.Z	
**Distribution**	Negative Binomial	
**Link Function**	Log	
**Dependent Variable**	AscoreR	HADS Anxiety score

**Number of Observations Read**	1293
**Number of Observations Used**	1293

Class Level Information				
**Class**	**Value**	**Design Variables**		
**gender**	**Female**	0	0	
	**Male**	1	0	
	**Other**	0	1	
**race**	**black**	1	0	
	**other race**	0	1	
	**white**	0	0	
**age_cat**	**35-54**	1	0	
	**greater than 55**	0	1	
	**less than 35**	0	0	
**inc**	**$100,000 and above**	0	0	0
	**40,000 - $99,999**	1	0	0
	**less than $40,000**	0	1	0
	**missing**	0	0	1
**edu**	**college degree or higher**	0	0	
	**missing**	1	0	
	**no college degree**	0	1	

Fully adjusted models of Anxietry score with gender*theme interaction

The GENMOD Procedure

Class Level Information				
**Class**	**Value**	**Design Variables**		
**kids**	**missing**	1	0	
	**no**	0	0	
	**yes**	0	1	
**marital_status**	**married or living as married**	1	0	0
	**missing**	0	1	0
	**single**	0	0	0
	**widowed, divorced**	0	0	1
**feltunwell**	**missing**	1	0	
	**no**	0	0	
	**yes**	0	1	
**compare_others**	**good to excellent**	0		
	**poor or fair**	1		
**ratehealth**	**good to excellent**	0		
	**poor or fair**	1		
**x1**	**no**	0		
	**yes**	1		
**x2**	**no**	0		
	**yes**	1		
**x3**	**no**	0		
	**yes**	1		
**x4**	**no**	0		
	**yes**	1		
**x5**	**no**	0		
	**yes**	1		

The GENMOD Procedure

Class Level Information				
Class	Value	Design Variables		
x6	no	0		
	yes	1		
x8	no	0		
	yes	1		
x9	no	0		
	yes	1		
x10	no	0		
	yes	1		
x11	no	0		
	yes	1		
x12	no	0		
	yes	1		
x13	no	0		
	yes	1		
x14	no	0		
	yes	1		
upset	do not want to answer	1	0	0
	missing	0	0	0
	no	0	1	0
	yes	0	0	1
hadajob	no	1		
	yes	0		
infected	no	0		
	yes	1		

Fully adjusted models of Anxietry score with gender*theme interaction

The GENMOD Procedure

Criteria For Assessing Goodness Of Fit			
Criterion	DF	Value	Value/DF
Deviance	1242	1503.0872	1.2102
Scaled Deviance	1242	1503.0872	1.2102
Pearson Chi-Square	1242	1285.2141	1.0348
Scaled Pearson X2	1242	1285.2141	1.0348
Log Likelihood		15323.7760	
Full Log Likelihood		-3618.8301	
AIC (smaller is better)		7341.6602	
AICC (smaller is better)		7346.1054	
BIC (smaller is better)		7610.2257	

Algorithm converged.

Analysis Of Maximum Likelihood Parameter Estimates									
Parameter			DF	Estimate	Standard Error	Wald 95% Confidence Limits		Wald Chi-Square	Pr > ChiSq
Intercept			1	2.2552	0.0836	2.0914	2.4191	727.82	<.0001
gender*x1	Male	yes	1	0.0072	0.0771	-0.1438	0.1583	0.01	0.9253
gender*x1	Other	yes	1	-0.4438	0.2854	-1.0032	0.1155	2.42	0.1199
x1	yes		1	0.0104	0.0361	-0.0604	0.0811	0.08	0.7737
x2	yes		1	0.1431	0.1600	-0.1706	0.4567	0.80	0.3714
x3	yes		1	-0.0610	0.0553	-0.1693	0.0473	1.22	0.2696
x4	yes		1	0.0680	0.0463	-0.0228	0.1588	2.16	0.1420
x5	yes		1	0.0774	0.0401	-0.0012	0.1561	3.73	0.0535
x6	yes		1	-0.0467	0.0422	-0.1294	0.0361	1.22	0.2692
x8	yes		1	0.1013	0.0392	0.0245	0.1780	6.69	0.0097
x9	yes		1	0.1717	0.1368	-0.0964	0.4398	1.58	0.2095

Fully adjusted models of Anxietry score with gender*theme interaction

The GENMOD Procedure

Analysis Of Maximum Likelihood Parameter Estimates									
Parameter			DF	Estimate	Standard Error	Wald 95% Confidence Limits		Wald Chi-Square	Pr > ChiSq
x10	yes		1	0.0745	0.0437	-0.0112	0.1603	2.90	0.0884
x11	yes		1	0.0198	0.1128	-0.2013	0.2410	0.03	0.8605
x12	yes		1	-0.0022	0.1465	-0.2893	0.2849	0.00	0.9881
x13	yes		1	0.0936	0.0758	-0.0550	0.2421	1.52	0.2171
x14	yes		1	0.0571	0.1407	-0.2186	0.3329	0.16	0.6848
upset	do not want to answer		1	-0.0454	0.1562	-0.3515	0.2606	0.08	0.7711
upset	no		1	-0.0019	0.0311	-0.0630	0.0591	0.00	0.9505
upset	yes		1	0.1666	0.0478	0.0730	0.2602	12.17	0.0005
dur			1	0.0017	0.0005	0.0008	0.0026	13.98	0.0002
support_family			1	-0.0021	0.0005	-0.0030	-0.0011	17.99	<.0001
support_doctor			1	-0.0004	0.0005	-0.0013	0.0006	0.59	0.4417
support_fedgovt			1	-0.0008	0.0007	-0.0021	0.0005	1.37	0.2414
support_city			1	0.0010	0.0007	-0.0004	0.0024	1.91	0.1675
support_deptpubhealt			1	0.0002	0.0007	-0.0012	0.0015	0.05	0.8237
support_faithcommuni			1	-0.0006	0.0005	-0.0016	0.0003	1.62	0.2038
support_socialservic			1	0.0011	0.0007	-0.0002	0.0024	2.64	0.1041
support_neighbors			1	-0.0006	0.0005	-0.0016	0.0005	1.16	0.2820
support_other			1	-0.0002	0.0006	-0.0014	0.0010	0.10	0.7493
infected	yes		1	0.0494	0.1156	-0.1773	0.2761	0.18	0.6693
feltunwell	missing		1	-0.1002	0.2344	-0.5596	0.3592	0.18	0.6690
feltunwell	yes		1	0.0551	0.0289	-0.0016	0.1118	3.63	0.0568
compare_others	poor or fair		1	0.1354	0.0467	0.0439	0.2268	8.42	0.0037
ratehealth	poor or fair		1	0.0262	0.0624	-0.0962	0.1485	0.18	0.6751
gender	Male		1	-0.1640	0.0320	-0.2268	-0.1013	26.24	<.0001
gender	Other		1	0.0962	0.1093	-0.1180	0.3104	0.77	0.3787

Fully adjusted models of Anxietry score with gender*theme interaction

The GENMOD Procedure

Analysis Of Maximum Likelihood Parameter Estimates									
**Parameter**			**DF**	**Estimate**	**Standard Error**	**Wald 95% Confidence Limits**		**Wald Chi-Square**	**Pr > ChiSq**
**race**	black		1	-0.1297	0.0509	-0.2295	-0.0299	6.49	0.0108
**race**	other race		1	-0.0941	0.0486	-0.1893	0.0011	3.75	0.0528
**age_cat**	35-54		1	-0.0315	0.0374	-0.1048	0.0418	0.71	0.4000
**age_cat**	greater than 55		1	-0.1959	0.0406	-0.2755	-0.1162	23.23	<.0001
**inc**	40,000 - $99,999		1	0.0780	0.0306	0.0181	0.1380	6.50	0.0108
**inc**	less than $40,000		1	0.1399	0.0390	0.0634	0.2164	12.85	0.0003
**inc**	missing		1	0.0675	0.0807	-0.0906	0.2256	0.70	0.4027
**edu**	missing		1	-0.1931	0.1873	-0.5602	0.1740	1.06	0.3025
**edu**	no college degree		1	0.0130	0.0363	-0.0582	0.0841	0.13	0.7208
**marital_status**	married or living as married		1	0.0940	0.0316	0.0321	0.1559	8.86	0.0029
**marital_status**	missing		1	0.3956	0.1542	0.0934	0.6979	6.58	0.0103
**marital_status**	widowed, divorced		1	-0.0092	0.0451	-0.0975	0.0791	0.04	0.8383
**kids**	missing		1	-0.3012	0.1872	-0.6682	0.0657	2.59	0.1077
**kids**	yes		1	-0.0150	0.0354	-0.0844	0.0545	0.18	0.6727
**hadajob**	no		1	-0.0534	0.0329	-0.1179	0.0112	2.62	0.1053
**Dispersion**			1	0.0744	0.0078	0.0606	0.0914		

Note: The negative binomial dispersion parameter was estimated by maximum likelihood.

LR Statistics For Joint Tests			
**Source**	**DF**	**Chi-Square**	**Pr > ChiSq**
**gender*x1**	2	2.50	0.2860
**x1**	1	0.08	0.7738
**x2**	1	0.79	0.3729
**x3**	1	1.22	0.2694
**x4**	1	2.15	0.1427

The GENMOD Procedure

LR Statistics For Joint Tests			
Source	DF	Chi-Square	Pr > ChiSq
x5	1	3.71	0.0540
x6	1	1.22	0.2688
x8	1	6.65	0.0099
x9	1	1.57	0.2106
x10	1	2.89	0.0891
x11	1	0.03	0.8605
x12	1	0.00	0.9881
x13	1	1.52	0.2179
x14	1	0.16	0.6853
upset	3	16.89	0.0007
dur	1	13.91	0.0002
support_family	1	17.84	<.0001
support_doctor	1	0.59	0.4417
support_fedgovt	1	1.37	0.2413
support_city	1	1.91	0.1675
support_deptpubhealt	1	0.05	0.8238
support_faithcommuni	1	1.61	0.2038
support_socialservic	1	2.64	0.1043
support_neighbors	1	1.16	0.2820
support_other	1	0.10	0.7493
infected	1	0.18	0.6696
feltunwell	2	3.87	0.1446
compare_others	1	8.37	0.0038
ratehealth	1	0.18	0.6751
gender	2	27.70	<.0001
race	2	9.50	0.0086

The GENMOD Procedure

LR Statistics For Joint Tests			
**Source**	**DF**	**Chi-Square**	**Pr > ChiSq**
**age_cat**	2	32.96	<.0001
**inc**	3	13.13	0.0044
**edu**	2	1.24	0.5380
**marital_status**	3	17.00	0.0007
**kids**	2	2.76	0.2516
**hadajob**	1	2.62	0.1054

Note: Under full-rank parameterizations, Type 3 effect tests are replaced by joint tests. The joint test for an effect is a test that all the parameters associated with that effect are zero. Such joint tests might not be equivalent to Type 3 effect tests under GLM parameterization.

The GENMOD Procedure

Model Information		
**Data Set**	WORK.Z	
**Distribution**	Negative Binomial	
**Link Function**	Log	
**Dependent Variable**	AscoreR	HADS Anxiety score

**Number of Observations Read**	1293
**Number of Observations Used**	1293

Class Level Information				
**Class**	**Value**	**Design Variables**		
**gender**	**Female**	0	0	
	**Male**	1	0	
	**Other**	0	1	
**race**	**black**	1	0	
	**other race**	0	1	
	**white**	0	0	
**age_cat**	**35-54**	1	0	
	**greater than 55**	0	1	
	**less than 35**	0	0	
**inc**	**$100,000 and above**	0	0	0
	**40,000 - $99,999**	1	0	0
	**less than $40,000**	0	1	0
	**missing**	0	0	1
**edu**	**college degree or higher**	0	0	
	**missing**	1	0	
	**no college degree**	0	1	

Fully adjusted models of Anxietry score with gender*theme interaction

The GENMOD Procedure

Class Level Information				
Class	Value	Design Variables		
kids	missing	1	0	
	no	0	0	
	yes	0	1	
marital_status	married or living as married	1	0	0
	missing	0	1	0
	single	0	0	0
	widowed, divorced	0	0	1
feltunwell	missing	1	0	
	no	0	0	
	yes	0	1	
compare_others	good to excellent	0		
	poor or fair	1		
ratehealth	good to excellent	0		
	poor or fair	1		
x1	no	0		
	yes	1		
x2	no	0		
	yes	1		
x3	no	0		
	yes	1		
x4	no	0		
	yes	1		
x5	no	0		
	yes	1		

The GENMOD Procedure

Class Level Information				
Class	Value	Design Variables		
x6	no	0		
	yes	1		
x8	no	0		
	yes	1		
x9	no	0		
	yes	1		
x10	no	0		
	yes	1		
x11	no	0		
	yes	1		
x12	no	0		
	yes	1		
x13	no	0		
	yes	1		
x14	no	0		
	yes	1		
upset	do not want to answer	1	0	0
	missing	0	0	0
	no	0	1	0
	yes	0	0	1
hadajob	no	1		
	yes	0		
infected	no	0		
	yes	1		

The GENMOD Procedure

Criteria For Assessing Goodness Of Fit			
**Criterion**	**DF**	**Value**	**Value/DF**
**Deviance**	1243	1502.7406	1.2090
**Scaled Deviance**	1243	1502.7406	1.2090
**Pearson Chi-Square**	1243	1284.8135	1.0336
**Scaled Pearson X2**	1243	1284.8135	1.0336
**Log Likelihood**		15323.2500	
**Full Log Likelihood**		-3619.3561	
**AIC (smaller is better)**		7340.7122	
**AICC (smaller is better)**		7344.9862	
**BIC (smaller is better)**		7604.1129	

Algorithm converged.

Analysis Of Maximum Likelihood Parameter Estimates									
**Parameter**			**DF**	**Estimate**	**Standard Error**	**Wald 95% Confidence Limits**		**Wald Chi-Square**	**Pr > ChiSq**
**Intercept**			1	2.2683	0.0830	2.1057	2.4309	747.51	<.0001
**gender*x2**	Male	yes	1	0.4214	0.3484	-0.2615	1.1043	1.46	0.2264
**gender*x2**	Other	yes	0	0.0000	0.0000	0.0000	0.0000	.	.
**x1**	yes		1	0.0058	0.0323	-0.0575	0.0691	0.03	0.8572
**x2**	yes		1	0.0215	0.1896	-0.3501	0.3931	0.01	0.9099
**x3**	yes		1	-0.0717	0.0552	-0.1799	0.0366	1.68	0.1943
**x4**	yes		1	0.0683	0.0463	-0.0225	0.1591	2.17	0.1406
**x5**	yes		1	0.0776	0.0401	-0.0010	0.1563	3.74	0.0531
**x6**	yes		1	-0.0483	0.0422	-0.1311	0.0345	1.31	0.2526
**x8**	yes		1	0.0988	0.0392	0.0221	0.1756	6.37	0.0116
**x9**	yes		1	0.1723	0.1368	-0.0959	0.4404	1.58	0.2081

The GENMOD Procedure

Analysis Of Maximum Likelihood Parameter Estimates									
Parameter			DF	Estimate	Standard Error	Wald 95% Confidence Limits		Wald Chi-Square	Pr > ChiSq
x10	yes		1	0.0723	0.0437	-0.0133	0.1580	2.74	0.0979
x11	yes		1	0.0207	0.1129	-0.2005	0.2420	0.03	0.8542
x12	yes		1	0.0051	0.1464	-0.2819	0.2921	0.00	0.9721
x13	yes		1	0.0955	0.0758	-0.0531	0.2441	1.59	0.2080
x14	yes		1	0.0563	0.1407	-0.2195	0.3321	0.16	0.6891
upset	do not want to answer		1	-0.0245	0.1554	-0.3290	0.2800	0.02	0.8747
upset	no		1	-0.0035	0.0312	-0.0646	0.0576	0.01	0.9104
upset	yes		1	0.1662	0.0478	0.0725	0.2599	12.09	0.0005
dur			1	0.0017	0.0005	0.0008	0.0026	14.00	0.0002
support_family			1	-0.0021	0.0005	-0.0031	-0.0011	18.48	<.0001
support_doctor			1	-0.0004	0.0005	-0.0013	0.0006	0.58	0.4470
support_fedgovt			1	-0.0008	0.0007	-0.0021	0.0005	1.49	0.2215
support_city			1	0.0010	0.0007	-0.0004	0.0025	2.00	0.1570
support_deptpubhealt			1	0.0001	0.0007	-0.0012	0.0015	0.03	0.8569
support_faithcommuni			1	-0.0007	0.0005	-0.0017	0.0003	1.72	0.1892
support_socialservic			1	0.0011	0.0007	-0.0002	0.0024	2.60	0.1068
support_neighbors			1	-0.0005	0.0005	-0.0015	0.0005	1.05	0.3065
support_other			1	-0.0002	0.0006	-0.0015	0.0010	0.16	0.6878
infected	yes		1	0.0447	0.1157	-0.1819	0.2714	0.15	0.6989
feltunwell	missing		1	-0.1000	0.2345	-0.5597	0.3597	0.18	0.6699
feltunwell	yes		1	0.0581	0.0289	0.0015	0.1148	4.04	0.0444
compare_others	poor or fair		1	0.1271	0.0464	0.0362	0.2180	7.51	0.0062
ratehealth	poor or fair		1	0.0330	0.0623	-0.0890	0.1550	0.28	0.5959
gender	Male		1	-0.1662	0.0294	-0.2238	-0.1086	31.96	<.0001
gender	Other		1	0.0283	0.1008	-0.1692	0.2258	0.08	0.7788

The GENMOD Procedure

Analysis Of Maximum Likelihood Parameter Estimates								
Parameter		DF	Estimate	Standard Error	Wald 95% Confidence Limits		Wald Chi-Square	Pr > ChiSq
**race**	black	1	-0.1311	0.0510	-0.2310	-0.0312	6.61	0.0101
**race**	other race	1	-0.0922	0.0485	-0.1872	0.0029	3.61	0.0574
**age_cat**	35-54	1	-0.0341	0.0375	-0.1075	0.0394	0.83	0.3634
**age_cat**	greater than 55	1	-0.1997	0.0406	-0.2793	-0.1201	24.19	<.0001
**inc**	40,000 - $99,999	1	0.0763	0.0306	0.0163	0.1362	6.22	0.0126
**inc**	less than $40,000	1	0.1384	0.0391	0.0619	0.2149	12.56	0.0004
**inc**	missing	1	0.0672	0.0807	-0.0910	0.2253	0.69	0.4052
**edu**	missing	1	-0.1933	0.1868	-0.5594	0.1728	1.07	0.3007
**edu**	no college degree	1	0.0135	0.0363	-0.0577	0.0848	0.14	0.7096
**marital_status**	married or living as married	1	0.0949	0.0316	0.0330	0.1568	9.02	0.0027
**marital_status**	missing	1	0.3938	0.1543	0.0913	0.6962	6.51	0.0107
**marital_status**	widowed, divorced	1	-0.0112	0.0451	-0.0996	0.0772	0.06	0.8039
**kids**	missing	1	-0.3019	0.1872	-0.6688	0.0650	2.60	0.1069
**kids**	yes	1	-0.0154	0.0354	-0.0848	0.0540	0.19	0.6642
**hadajob**	no	1	-0.0537	0.0330	-0.1183	0.0109	2.65	0.1033
**Dispersion**		1	0.0746	0.0078	0.0608	0.0916		

Note: The negative binomial dispersion parameter was estimated by maximum likelihood.

LR Statistics For Joint Tests			
Source	DF	Chi-Square	Pr > ChiSq
**gender*x2**	2	3.47	0.1763
**x1**	1	0.03	0.8572
**x2**	1	0.01	0.9099
**x3**	1	1.69	0.1941
**x4**	1	2.16	0.1413

The GENMOD Procedure

LR Statistics For Joint Tests			
Source	DF	Chi-Square	Pr > ChiSq
x5	1	3.73	0.0536
x6	1	1.31	0.2522
x8	1	6.33	0.0118
x9	1	1.58	0.2092
x10	1	2.73	0.0986
x11	1	0.03	0.8543
x12	1	0.00	0.9721
x13	1	1.58	0.2089
x14	1	0.16	0.6897
upset	3	16.95	0.0007
dur	1	13.93	0.0002
support_family	1	18.32	<.0001
support_doctor	1	0.58	0.4470
support_fedgovt	1	1.50	0.2213
support_city	1	2.00	0.1570
support_deptpubhealt	1	0.03	0.8569
support_faithcommuni	1	1.72	0.1892
support_socialservic	1	2.60	0.1071
support_neighbors	1	1.05	0.3065
support_other	1	0.16	0.6878
infected	1	0.15	0.6991
feltunwell	2	4.28	0.1175
compare_others	1	7.46	0.0063
ratehealth	1	0.28	0.5960
gender	2	32.13	<.0001
race	2	9.50	0.0087

Fully adjusted models of Anxiety score with gender*theme interaction

The GENMOD Procedure

LR Statistics For Joint Tests			
**Source**	**DF**	**Chi-Square**	**Pr > ChiSq**
**age_cat**	2	33.91	<.0001
**inc**	3	12.79	0.0051
**edu**	2	1.26	0.5329
**marital_status**	3	17.33	0.0006
**kids**	2	2.78	0.2489
**hadajob**	1	2.65	0.1034

Note: Under full-rank parameterizations, Type 3 effect tests are replaced by joint tests. The joint test for an effect is a test that all the parameters associated with that effect are zero. Such joint tests might not be equivalent to Type 3 effect tests under GLM parameterization.

Fully adjusted models of Anxietry score with gender*theme interaction

The GENMOD Procedure

Model Information		
**Data Set**	WORK.Z	
**Distribution**	Negative Binomial	
**Link Function**	Log	
**Dependent Variable**	AscoreR	HADS Anxiety score

**Number of Observations Read**	1293
**Number of Observations Used**	1293

Class Level Information				
**Class**	**Value**	**Design Variables**		
**gender**	**Female**	0	0	
	**Male**	1	0	
	**Other**	0	1	
**race**	**black**	1	0	
	**other race**	0	1	
	**white**	0	0	
**age_cat**	**35-54**	1	0	
	**greater than 55**	0	1	
	**less than 35**	0	0	
**inc**	**$100,000 and above**	0	0	0
	**40,000 - $99,999**	1	0	0
	**less than $40,000**	0	1	0
	**missing**	0	0	1
**edu**	**college degree or higher**	0	0	
	**missing**	1	0	
	**no college degree**	0	1	

The GENMOD Procedure

Class Level Information				
Class	Value	Design Variables		
kids	missing	1	0	
	no	0	0	
	yes	0	1	
marital_status	married or living as married	1	0	0
	missing	0	1	0
	single	0	0	0
	widowed, divorced	0	0	1
feltunwell	missing	1	0	
	no	0	0	
	yes	0	1	
compare_others	good to excellent	0		
	poor or fair	1		
ratehealth	good to excellent	0		
	poor or fair	1		
x1	no	0		
	yes	1		
x2	no	0		
	yes	1		
x3	no	0		
	yes	1		
x4	no	0		
	yes	1		
x5	no	0		
	yes	1		

The GENMOD Procedure

Class Level Information				
Class	Value	Design Variables		
x6	no	0		
	yes	1		
x8	no	0		
	yes	1		
x9	no	0		
	yes	1		
x10	no	0		
	yes	1		
x11	no	0		
	yes	1		
x12	no	0		
	yes	1		
x13	no	0		
	yes	1		
x14	no	0		
	yes	1		
upset	do not want to answer	1	0	0
	missing	0	0	0
	no	0	1	0
	yes	0	0	1
hadajob	no	1		
	yes	0		
infected	no	0		
	yes	1		

Fully adjusted models of Anxietry score with gender*theme interaction

The GENMOD Procedure

Criteria For Assessing Goodness Of Fit			
**Criterion**	**DF**	**Value**	**Value/DF**
**Deviance**	1242	1502.5593	1.2098
**Scaled Deviance**	1242	1502.5593	1.2098
**Pearson Chi-Square**	1242	1284.6105	1.0343
**Scaled Pearson X2**	1242	1284.6105	1.0343
**Log Likelihood**		15322.7629	
**Full Log Likelihood**		-3619.8432	
**AIC (smaller is better)**		7343.6864	
**AICC (smaller is better)**		7348.1316	
**BIC (smaller is better)**		7612.2519	

Algorithm converged.

Analysis Of Maximum Likelihood Parameter Estimates									
**Parameter**			**DF**	**Estimate**	**Standard Error**	**Wald 95% Confidence Limits**		**Wald Chi-Square**	**Pr > ChiSq**
**Intercept**			1	2.2647	0.0833	2.1015	2.4279	739.66	<.0001
**gender*x3**	Male	yes	1	0.0805	0.1270	-0.1684	0.3294	0.40	0.5261
**gender*x3**	Other	yes	1	-0.1084	0.4403	-0.9713	0.7545	0.06	0.8055
**x1**	yes		1	0.0078	0.0324	-0.0558	0.0713	0.06	0.8109
**x2**	yes		1	0.1326	0.1610	-0.1830	0.4481	0.68	0.4102
**x3**	yes		1	-0.0794	0.0596	-0.1963	0.0374	1.78	0.1826
**x4**	yes		1	0.0687	0.0464	-0.0221	0.1596	2.20	0.1383
**x5**	yes		1	0.0774	0.0402	-0.0013	0.1561	3.71	0.0540
**x6**	yes		1	-0.0481	0.0423	-0.1310	0.0347	1.30	0.2550
**x8**	yes		1	0.0993	0.0392	0.0225	0.1761	6.42	0.0113
**x9**	yes		1	0.1718	0.1369	-0.0965	0.4402	1.57	0.2095

Fully adjusted models of Anxietry score with gender*theme interaction

The GENMOD Procedure

Analysis Of Maximum Likelihood Parameter Estimates									
Parameter			DF	Estimate	Standard Error	Wald 95% Confidence Limits		Wald Chi-Square	Pr > ChiSq
**x10**	yes		1	0.0720	0.0437	-0.0137	0.1578	2.71	0.0996
**x11**	yes		1	0.0220	0.1130	-0.1996	0.2435	0.04	0.8460
**x12**	yes		1	0.0043	0.1465	-0.2828	0.2915	0.00	0.9764
**x13**	yes		1	0.0963	0.0759	-0.0524	0.2451	1.61	0.2045
**x14**	yes		1	0.0559	0.1408	-0.2201	0.3319	0.16	0.6913
**upset**	do not want to answer		1	-0.0263	0.1557	-0.3314	0.2788	0.03	0.8658
**upset**	no		1	-0.0025	0.0312	-0.0636	0.0586	0.01	0.9352
**upset**	yes		1	0.1655	0.0478	0.0717	0.2592	11.96	0.0005
**dur**			1	0.0017	0.0005	0.0008	0.0026	13.87	0.0002
**support_family**			1	-0.0021	0.0005	-0.0031	-0.0011	18.39	<.0001
**support_doctor**			1	-0.0003	0.0005	-0.0013	0.0006	0.50	0.4816
**support_fedgovt**			1	-0.0008	0.0007	-0.0021	0.0005	1.46	0.2262
**support_city**			1	0.0010	0.0007	-0.0004	0.0024	1.83	0.1757
**support_deptpubhealt**			1	0.0002	0.0007	-0.0012	0.0015	0.05	0.8193
**support_faithcommuni**			1	-0.0007	0.0005	-0.0017	0.0003	1.78	0.1815
**support_socialservic**			1	0.0011	0.0007	-0.0002	0.0025	2.70	0.1001
**support_neighbors**			1	-0.0005	0.0005	-0.0015	0.0005	1.12	0.2908
**support_other**			1	-0.0003	0.0006	-0.0015	0.0010	0.18	0.6700
**infected**	yes		1	0.0448	0.1157	-0.1820	0.2717	0.15	0.6985
**feltunwell**	missing		1	-0.1003	0.2346	-0.5602	0.3596	0.18	0.6690
**feltunwell**	yes		1	0.0577	0.0289	0.0010	0.1145	3.98	0.0461
**compare_others**	poor or fair		1	0.1298	0.0467	0.0383	0.2214	7.73	0.0054
**ratehealth**	poor or fair		1	0.0310	0.0624	-0.0913	0.1534	0.25	0.6190
**gender**	Male		1	-0.1677	0.0301	-0.2268	-0.1086	30.97	<.0001
**gender**	Other		1	0.0355	0.1040	-0.1684	0.2393	0.12	0.7329

Fully adjusted models of Anxietry score with gender*theme interaction

The GENMOD Procedure

Analysis Of Maximum Likelihood Parameter Estimates									
Parameter			DF	Estimate	Standard Error	Wald 95% Confidence Limits		Wald Chi-Square	Pr > ChiSq
**race**	black		1	-0.1283	0.0510	-0.2282	-0.0283	6.32	0.0119
**race**	other race		1	-0.0921	0.0486	-0.1872	0.0031	3.60	0.0579
**age_cat**	35-54		1	-0.0313	0.0374	-0.1047	0.0421	0.70	0.4031
**age_cat**	greater than 55		1	-0.1971	0.0406	-0.2767	-0.1175	23.54	<.0001
**inc**	40,000 - $99,999		1	0.0771	0.0306	0.0171	0.1372	6.35	0.0118
**inc**	less than $40,000		1	0.1396	0.0391	0.0631	0.2162	12.78	0.0004
**inc**	missing		1	0.0653	0.0808	-0.0931	0.2237	0.65	0.4190
**edu**	missing		1	-0.1942	0.1868	-0.5604	0.1720	1.08	0.2987
**edu**	no college degree		1	0.0125	0.0363	-0.0588	0.0837	0.12	0.7319
**marital_status**	married or living as married		1	0.0951	0.0316	0.0332	0.1571	9.05	0.0026
**marital_status**	missing		1	0.3944	0.1544	0.0918	0.6970	6.52	0.0106
**marital_status**	widowed, divorced		1	-0.0103	0.0451	-0.0987	0.0781	0.05	0.8198
**kids**	missing		1	-0.2987	0.1874	-0.6659	0.0686	2.54	0.1110
**kids**	yes		1	-0.0157	0.0354	-0.0852	0.0538	0.20	0.6583
**hadajob**	no		1	-0.0532	0.0330	-0.1178	0.0115	2.60	0.1070
**Dispersion**			1	0.0748	0.0078	0.0610	0.0918		

Note: The negative binomial dispersion parameter was estimated by maximum likelihood.

LR Statistics For Joint Tests			
Source	DF	Chi-Square	Pr > ChiSq
**gender*x3**	2	0.48	0.7878
**x1**	1	0.06	0.8109
**x2**	1	0.67	0.4115
**x3**	1	1.78	0.1824
**x4**	1	2.19	0.1390

The GENMOD Procedure

LR Statistics For Joint Tests			
**Source**	**DF**	**Chi-Square**	**Pr > ChiSq**
**x5**	1	3.70	0.0545
**x6**	1	1.30	0.2546
**x8**	1	6.39	0.0115
**x9**	1	1.57	0.2106
**x10**	1	2.70	0.1003
**x11**	1	0.04	0.8461
**x12**	1	0.00	0.9764
**x13**	1	1.60	0.2053
**x14**	1	0.16	0.6918
**upset**	3	16.60	0.0009
**dur**	1	13.80	0.0002
**support_family**	1	18.23	<.0001
**support_doctor**	1	0.50	0.4817
**support_fedgovt**	1	1.47	0.2260
**support_city**	1	1.83	0.1757
**support_deptpubhealt**	1	0.05	0.8194
**support_faithcommuni**	1	1.78	0.1816
**support_socialservic**	1	2.70	0.1004
**support_neighbors**	1	1.12	0.2908
**support_other**	1	0.18	0.6701
**infected**	1	0.15	0.6987
**feltunwell**	2	4.22	0.1213
**compare_others**	1	7.69	0.0056
**ratehealth**	1	0.25	0.6191
**gender**	2	31.25	<.0001
**race**	2	9.19	0.0101

The GENMOD Procedure

LR Statistics For Joint Tests			
**Source**	**DF**	**Chi-Square**	**Pr > ChiSq**
**age_cat**	2	33.48	<.0001
**inc**	3	13.02	0.0046
**edu**	2	1.24	0.5367
**marital_status**	3	17.28	0.0006
**kids**	2	2.73	0.2560
**hadajob**	1	2.60	0.1071

Note: Under full-rank parameterizations, Type 3 effect tests are replaced by joint tests. The joint test for an effect is a test that all the parameters associated with that effect are zero. Such joint tests might not be equivalent to Type 3 effect tests under GLM parameterization.

The GENMOD Procedure

Model Information		
**Data Set**	WORK.Z	
**Distribution**	Negative Binomial	
**Link Function**	Log	
**Dependent Variable**	AscoreR	HADS Anxiety score

**Number of Observations Read**	1293
**Number of Observations Used**	1293

Class Level Information				
**Class**	**Value**	**Design Variables**		
**gender**	**Female**	0	0	
	**Male**	1	0	
	**Other**	0	1	
**race**	**black**	1	0	
	**other race**	0	1	
	**white**	0	0	
**age_cat**	**35-54**	1	0	
	**greater than 55**	0	1	
	**less than 35**	0	0	
**inc**	**$100,000 and above**	0	0	0
	**40,000 - $99,999**	1	0	0
	**less than $40,000**	0	1	0
	**missing**	0	0	1
**edu**	**college degree or higher**	0	0	
	**missing**	1	0	
	**no college degree**	0	1	

The GENMOD Procedure

Class Level Information				
Class	Value	Design Variables		
kids	missing	1	0	
	no	0	0	
	yes	0	1	
marital_status	married or living as married	1	0	0
	missing	0	1	0
	single	0	0	0
	widowed, divorced	0	0	1
feltunwell	missing	1	0	
	no	0	0	
	yes	0	1	
compare_others	good to excellent	0		
	poor or fair	1		
ratehealth	good to excellent	0		
	poor or fair	1		
x1	no	0		
	yes	1		
x2	no	0		
	yes	1		
x3	no	0		
	yes	1		
x4	no	0		
	yes	1		
x5	no	0		
	yes	1		

Fully adjusted models of Anxietry score with gender*theme interaction

The GENMOD Procedure

Class Level Information				
**Class**	**Value**	**Design Variables**		
**x6**	**no**	0		
	**yes**	1		
**x8**	**no**	0		
	**yes**	1		
**x9**	**no**	0		
	**yes**	1		
**x10**	**no**	0		
	**yes**	1		
**x11**	**no**	0		
	**yes**	1		
**x12**	**no**	0		
	**yes**	1		
**x13**	**no**	0		
	**yes**	1		
**x14**	**no**	0		
	**yes**	1		
**upset**	**do not want to answer**	1	0	0
	**missing**	0	0	0
	**no**	0	1	0
	**yes**	0	0	1
**hadajob**	**no**	1		
	**yes**	0		
**infected**	**no**	0		
	**yes**	1		

Fully adjusted models of Anxietry score with gender*theme interaction

The GENMOD Procedure

Criteria For Assessing Goodness Of Fit			
Criterion	DF	Value	Value/DF
Deviance	1243	1502.5067	1.2088
Scaled Deviance	1243	1502.5067	1.2088
Pearson Chi-Square	1243	1284.1067	1.0331
Scaled Pearson X2	1243	1284.1067	1.0331
Log Likelihood		15322.5315	
Full Log Likelihood		-3620.0746	
AIC (smaller is better)		7342.1492	
AICC (smaller is better)		7346.4232	
BIC (smaller is better)		7605.5500	

Algorithm converged.

Analysis Of Maximum Likelihood Parameter Estimates									
Parameter			DF	Estimate	Standard Error	Wald 95% Confidence Limits		Wald Chi-Square	Pr > ChiSq
Intercept			1	2.2645	0.0830	2.1017	2.4273	743.54	<.0001
gender*x4	Male	yes	1	-0.0129	0.1086	-0.2258	0.1999	0.01	0.9051
gender*x4	Other	yes	0	0.0000	0.0000	0.0000	0.0000	.	.
x1	yes		1	0.0062	0.0323	-0.0571	0.0695	0.04	0.8479
x2	yes		1	0.1428	0.1602	-0.1713	0.4568	0.79	0.3730
x3	yes		1	-0.0670	0.0551	-0.1750	0.0411	1.48	0.2243
x4	yes		1	0.0713	0.0528	-0.0321	0.1747	1.83	0.1765
x5	yes		1	0.0779	0.0402	-0.0008	0.1566	3.76	0.0523
x6	yes		1	-0.0489	0.0423	-0.1317	0.0340	1.34	0.2475
x8	yes		1	0.1000	0.0392	0.0231	0.1769	6.50	0.0108
x9	yes		1	0.1727	0.1369	-0.0958	0.4411	1.59	0.2074

Fully adjusted models of Anxietry score with gender*theme interaction

The GENMOD Procedure

Analysis Of Maximum Likelihood Parameter Estimates									
Parameter			DF	Estimate	Standard Error	Wald 95% Confidence Limits		Wald Chi-Square	Pr > ChiSq
x10	yes		1	0.0711	0.0437	-0.0147	0.1568	2.64	0.1042
x11	yes		1	0.0198	0.1131	-0.2018	0.2414	0.03	0.8613
x12	yes		1	0.0046	0.1465	-0.2826	0.2917	0.00	0.9752
x13	yes		1	0.0955	0.0759	-0.0532	0.2443	1.59	0.2080
x14	yes		1	0.0565	0.1409	-0.2196	0.3327	0.16	0.6883
upset	do not want to answer		1	-0.0228	0.1555	-0.3275	0.2819	0.02	0.8834
upset	no		1	-0.0028	0.0312	-0.0639	0.0583	0.01	0.9274
upset	yes		1	0.1666	0.0478	0.0729	0.2603	12.14	0.0005
dur			1	0.0017	0.0005	0.0008	0.0026	13.74	0.0002
support_family			1	-0.0021	0.0005	-0.0031	-0.0011	18.43	<.0001
support_doctor			1	-0.0003	0.0005	-0.0013	0.0006	0.50	0.4808
support_fedgovt			1	-0.0008	0.0007	-0.0021	0.0005	1.39	0.2383
support_city			1	0.0010	0.0007	-0.0004	0.0024	1.88	0.1705
support_deptpubhealt			1	0.0002	0.0007	-0.0012	0.0015	0.05	0.8255
support_faithcommuni			1	-0.0007	0.0005	-0.0017	0.0003	1.83	0.1757
support_socialservic			1	0.0011	0.0007	-0.0002	0.0025	2.70	0.1001
support_neighbors			1	-0.0005	0.0005	-0.0015	0.0005	1.08	0.2981
support_other			1	-0.0003	0.0006	-0.0015	0.0010	0.17	0.6802
infected	yes		1	0.0441	0.1158	-0.1829	0.2711	0.15	0.7033
feltunwell	missing		1	-0.0992	0.2347	-0.5592	0.3607	0.18	0.6724
feltunwell	yes		1	0.0577	0.0289	0.0010	0.1144	3.98	0.0459
compare_others	poor or fair		1	0.1273	0.0464	0.0363	0.2183	7.52	0.0061
ratehealth	poor or fair		1	0.0326	0.0623	-0.0895	0.1547	0.27	0.6005
gender	Male		1	-0.1624	0.0305	-0.2222	-0.1027	28.40	<.0001
gender	Other		1	0.0300	0.1008	-0.1676	0.2277	0.09	0.7659

Fully adjusted models of Anxietry score with gender*theme interaction

The GENMOD Procedure

Analysis Of Maximum Likelihood Parameter Estimates										
Parameter			DF	Estimate	Standard Error	Wald 95% Confidence Limits		Wald Chi-Square	Pr > ChiSq
race	black		1	-0.1292	0.0510	-0.2292	-0.0293	6.43	0.0113
race	other race		1	-0.0935	0.0485	-0.1886	0.0016	3.71	0.0540
age_cat	35-54		1	-0.0312	0.0374	-0.1045	0.0422	0.69	0.4054
age_cat	greater than 55		1	-0.1976	0.0406	-0.2772	-0.1180	23.68	<.0001
inc	40,000 - $99,999		1	0.0769	0.0306	0.0169	0.1369	6.31	0.0120
inc	less than $40,000		1	0.1394	0.0391	0.0628	0.2160	12.72	0.0004
inc	missing		1	0.0676	0.0808	-0.0907	0.2258	0.70	0.4028
edu	missing		1	-0.1948	0.1869	-0.5610	0.1714	1.09	0.2972
edu	no college degree		1	0.0120	0.0363	-0.0593	0.0832	0.11	0.7419
marital_status	married or living as married		1	0.0949	0.0316	0.0329	0.1568	9.01	0.0027
marital_status	missing		1	0.3929	0.1544	0.0902	0.6956	6.47	0.0110
marital_status	widowed, divorced		1	-0.0093	0.0451	-0.0978	0.0792	0.04	0.8362
kids	missing		1	-0.3025	0.1873	-0.6696	0.0646	2.61	0.1063
kids	yes		1	-0.0160	0.0355	-0.0855	0.0534	0.20	0.6509
hadajob	no		1	-0.0536	0.0330	-0.1182	0.0111	2.64	0.1044
Dispersion			1	0.0749	0.0078	0.0610	0.0919		

Note: The negative binomial dispersion parameter was estimated by maximum likelihood.

LR Statistics For Joint Tests			
Source	DF	Chi-Square	Pr > ChiSq
gender*x4	2	2.03	0.3616
x1	1	0.04	0.8479
x2	1	0.79	0.3745
x3	1	1.48	0.2241
x4	1	1.82	0.1772

The GENMOD Procedure

LR Statistics For Joint Tests			
Source	DF	Chi-Square	Pr > ChiSq
x5	1	3.75	0.0528
x6	1	1.34	0.2471
x8	1	6.46	0.0110
x9	1	1.58	0.2085
x10	1	2.63	0.1048
x11	1	0.03	0.8614
x12	1	0.00	0.9752
x13	1	1.58	0.2088
x14	1	0.16	0.6888
upset	3	16.89	0.0007
dur	1	13.67	0.0002
support_family	1	18.27	<.0001
support_doctor	1	0.50	0.4808
support_fedgovt	1	1.39	0.2382
support_city	1	1.88	0.1704
support_deptpubhealt	1	0.05	0.8255
support_faithcommuni	1	1.83	0.1757
support_socialservic	1	2.70	0.1003
support_neighbors	1	1.08	0.2981
support_other	1	0.17	0.6803
infected	1	0.14	0.7035
feltunwell	2	4.22	0.1212
compare_others	1	7.48	0.0062
ratehealth	1	0.27	0.6006
gender	2	28.64	<.0001
race	2	9.40	0.0091

The GENMOD Procedure

LR Statistics For Joint Tests			
**Source**	**DF**	**Chi-Square**	**Pr > ChiSq**
**age_cat**	2	33.67	<.0001
**inc**	3	12.96	0.0047
**edu**	2	1.24	0.5376
**marital_status**	3	17.09	0.0007
**kids**	2	2.80	0.2463
**hadajob**	1	2.64	0.1044

Note: Under full-rank parameterizations, Type 3 effect tests are replaced by joint tests. The joint test for an effect is a test that all the parameters associated with that effect are zero. Such joint tests might not be equivalent to Type 3 effect tests under GLM parameterization.

The GENMOD Procedure

Model Information		
Data Set	WORK.Z	
Distribution	Negative Binomial	
Link Function	Log	
Dependent Variable	AscoreR	HADS Anxiety score

Number of Observations Read	1293
Number of Observations Used	1293

Class Level Information				
Class	Value	Design Variables		
gender	Female	0	0	
	Male	1	0	
	Other	0	1	
race	black	1	0	
	other race	0	1	
	white	0	0	
age_cat	35-54	1	0	
	greater than 55	0	1	
	less than 35	0	0	
inc	$100,000 and above	0	0	0
	40,000 - $99,999	1	0	0
	less than $40,000	0	1	0
	missing	0	0	1
edu	college degree or higher	0	0	
	missing	1	0	
	no college degree	0	1	

The GENMOD Procedure

Class Level Information				
Class	Value	Design Variables		
kids	missing	1	0	
	no	0	0	
	yes	0	1	
marital_status	married or living as married	1	0	0
	missing	0	1	0
	single	0	0	0
	widowed, divorced	0	0	1
feltunwell	missing	1	0	
	no	0	0	
	yes	0	1	
compare_others	good to excellent	0		
	poor or fair	1		
ratehealth	good to excellent	0		
	poor or fair	1		
x1	no	0		
	yes	1		
x2	no	0		
	yes	1		
x3	no	0		
	yes	1		
x4	no	0		
	yes	1		
x5	no	0		
	yes	1		

The GENMOD Procedure

Class Level Information				
Class	Value	Design Variables		
x6	no	0		
	yes	1		
x8	no	0		
	yes	1		
x9	no	0		
	yes	1		
x10	no	0		
	yes	1		
x11	no	0		
	yes	1		
x12	no	0		
	yes	1		
x13	no	0		
	yes	1		
x14	no	0		
	yes	1		
upset	do not want to answer	1	0	0
	missing	0	0	0
	no	0	1	0
	yes	0	0	1
hadajob	no	1		
	yes	0		
infected	no	0		
	yes	1		

Fully adjusted models of Anxietry score with gender*theme interaction

The GENMOD Procedure

Criteria For Assessing Goodness Of Fit			
**Criterion**	**DF**	**Value**	**Value/DF**
**Deviance**	1242	1503.3753	1.2104
**Scaled Deviance**	1242	1503.3753	1.2104
**Pearson Chi-Square**	1242	1284.7471	1.0344
**Scaled Pearson X2**	1242	1284.7471	1.0344
**Log Likelihood**		15323.3052	
**Full Log Likelihood**		-3619.3010	
**AIC (smaller is better)**		7342.6019	
**AICC (smaller is better)**		7347.0471	
**BIC (smaller is better)**		7611.1674	

Algorithm converged.

Analysis Of Maximum Likelihood Parameter Estimates									
**Parameter**			**DF**	**Estimate**	**Standard Error**	**Wald 95% Confidence Limits**		**Wald Chi-Square**	**Pr > ChiSq**
**Intercept**			1	2.2669	0.0829	2.1043	2.4294	746.91	<.0001
**gender*x5**	Male	yes	1	0.1181	0.0952	-0.0685	0.3047	1.54	0.2149
**gender*x5**	Other	yes	1	0.0936	0.4074	-0.7050	0.8921	0.05	0.8184
**x1**	yes		1	0.0064	0.0323	-0.0569	0.0696	0.04	0.8440
**x2**	yes		1	0.1442	0.1601	-0.1697	0.4580	0.81	0.3679
**x3**	yes		1	-0.0666	0.0551	-0.1746	0.0413	1.46	0.2265
**x4**	yes		1	0.0682	0.0463	-0.0226	0.1589	2.17	0.1411
**x5**	yes		1	0.0509	0.0456	-0.0385	0.1403	1.25	0.2644
**x6**	yes		1	-0.0486	0.0422	-0.1314	0.0342	1.32	0.2498
**x8**	yes		1	0.1015	0.0392	0.0247	0.1782	6.71	0.0096
**x9**	yes		1	0.1776	0.1368	-0.0906	0.4457	1.68	0.1944

The GENMOD Procedure

Analysis Of Maximum Likelihood Parameter Estimates									
Parameter			DF	Estimate	Standard Error	Wald 95% Confidence Limits		Wald Chi-Square	Pr > ChiSq
x10	yes		1	0.0743	0.0438	-0.0114	0.1601	2.89	0.0894
x11	yes		1	0.0161	0.1129	-0.2051	0.2373	0.02	0.8865
x12	yes		1	0.0122	0.1465	-0.2749	0.2993	0.01	0.9336
x13	yes		1	0.0964	0.0758	-0.0522	0.2450	1.62	0.2036
x14	yes		1	0.0583	0.1407	-0.2175	0.3340	0.17	0.6786
upset	do not want to answer		1	-0.0243	0.1556	-0.3292	0.2807	0.02	0.8761
upset	no		1	-0.0034	0.0312	-0.0646	0.0578	0.01	0.9135
upset	yes		1	0.1635	0.0479	0.0696	0.2574	11.65	0.0006
dur			1	0.0017	0.0005	0.0008	0.0026	14.08	0.0002
support_family			1	-0.0021	0.0005	-0.0031	-0.0012	18.62	<.0001
support_doctor			1	-0.0003	0.0005	-0.0013	0.0007	0.41	0.5208
support_fedgovt			1	-0.0008	0.0007	-0.0021	0.0005	1.37	0.2426
support_city			1	0.0010	0.0007	-0.0004	0.0025	1.99	0.1579
support_deptpubhealt			1	0.0001	0.0007	-0.0013	0.0014	0.02	0.8996
support_faithcommuni			1	-0.0007	0.0005	-0.0017	0.0003	1.90	0.1685
support_socialservic			1	0.0011	0.0007	-0.0002	0.0024	2.66	0.1032
support_neighbors			1	-0.0005	0.0005	-0.0015	0.0005	1.07	0.3007
support_other			1	-0.0002	0.0006	-0.0015	0.0010	0.16	0.6911
infected	yes		1	0.0489	0.1157	-0.1778	0.2756	0.18	0.6727
feltunwell	missing		1	-0.0990	0.2345	-0.5585	0.3606	0.18	0.6730
feltunwell	yes		1	0.0592	0.0289	0.0025	0.1158	4.18	0.0408
compare_others	poor or fair		1	0.1258	0.0464	0.0348	0.2168	7.35	0.0067
ratehealth	poor or fair		1	0.0337	0.0622	-0.0883	0.1557	0.29	0.5879
gender	Male		1	-0.1750	0.0308	-0.2354	-0.1147	32.34	<.0001
gender	Other		1	0.0232	0.1040	-0.1806	0.2270	0.05	0.8237

The GENMOD Procedure

Analysis Of Maximum Likelihood Parameter Estimates									
Parameter			DF	Estimate	Standard Error	Wald 95% Confidence Limits		Wald Chi-Square	Pr > ChiSq
race	black		1	-0.1292	0.0509	-0.2290	-0.0293	6.43	0.0112
race	other race		1	-0.0912	0.0485	-0.1863	0.0038	3.54	0.0599
age_cat	35-54		1	-0.0313	0.0374	-0.1046	0.0420	0.70	0.4028
age_cat	greater than 55		1	-0.1968	0.0406	-0.2762	-0.1173	23.53	<.0001
inc	40,000 - $99,999		1	0.0759	0.0306	0.0159	0.1358	6.15	0.0131
inc	less than $40,000		1	0.1387	0.0390	0.0622	0.2152	12.62	0.0004
inc	missing		1	0.0660	0.0807	-0.0922	0.2241	0.67	0.4137
edu	missing		1	-0.1915	0.1867	-0.5574	0.1745	1.05	0.3052
edu	no college degree		1	0.0118	0.0363	-0.0594	0.0829	0.10	0.7461
marital_status	married or living as married		1	0.0950	0.0316	0.0331	0.1569	9.04	0.0026
marital_status	missing		1	0.3840	0.1546	0.0810	0.6870	6.17	0.0130
marital_status	widowed, divorced		1	-0.0101	0.0450	-0.0984	0.0781	0.05	0.8217
kids	missing		1	-0.2979	0.1872	-0.6647	0.0690	2.53	0.1115
kids	yes		1	-0.0166	0.0354	-0.0860	0.0528	0.22	0.6390
hadajob	no		1	-0.0541	0.0330	-0.1188	0.0105	2.69	0.1009
Dispersion			1	0.0745	0.0078	0.0607	0.0915		

Note: The negative binomial dispersion parameter was estimated by maximum likelihood.

LR Statistics For Joint Tests			
Source	DF	Chi-Square	Pr > ChiSq
gender*x5	2	1.56	0.4581
x1	1	0.04	0.8440
x2	1	0.81	0.3694
x3	1	1.46	0.2263
x4	1	2.16	0.1419

The GENMOD Procedure

LR Statistics For Joint Tests			
Source	DF	Chi-Square	Pr > ChiSq
x5	1	1.24	0.2648
x6	1	1.33	0.2494
x8	1	6.67	0.0098
x9	1	1.68	0.1956
x10	1	2.87	0.0900
x11	1	0.02	0.8866
x12	1	0.01	0.9336
x13	1	1.61	0.2044
x14	1	0.17	0.6792
upset	3	16.37	0.0010
dur	1	14.01	0.0002
support_family	1	18.46	<.0001
support_doctor	1	0.41	0.5208
support_fedgovt	1	1.37	0.2425
support_city	1	1.99	0.1579
support_deptpubhealt	1	0.02	0.8996
support_faithcommuni	1	1.90	0.1685
support_socialservic	1	2.65	0.1035
support_neighbors	1	1.07	0.3006
support_other	1	0.16	0.6912
infected	1	0.18	0.6730
feltunwell	2	4.42	0.1097
compare_others	1	7.31	0.0069
ratehealth	1	0.29	0.5880
gender	2	32.44	<.0001
race	2	9.25	0.0098

The GENMOD Procedure

LR Statistics For Joint Tests			
**Source**	**DF**	**Chi-Square**	**Pr > ChiSq**
**age_cat**	2	33.43	<.0001
**inc**	3	12.83	0.0050
**edu**	2	1.20	0.5487
**marital_status**	3	16.98	0.0007
**kids**	2	2.74	0.2543
**hadajob**	1	2.69	0.1010

Note: Under full-rank parameterizations, Type 3 effect tests are replaced by joint tests. The joint test for an effect is a test that all the parameters associated with that effect are zero. Such joint tests might not be equivalent to Type 3 effect tests under GLM parameterization.

The GENMOD Procedure

Model Information		
Data Set	WORK.Z	
Distribution	Negative Binomial	
Link Function	Log	
Dependent Variable	AscoreR	HADS Anxiety score

Number of Observations Read	1293
Number of Observations Used	1293

Class Level Information				
Class	Value	Design Variables		
gender	Female	0	0	
	Male	1	0	
	Other	0	1	
race	black	1	0	
	other race	0	1	
	white	0	0	
age_cat	35-54	1	0	
	greater than 55	0	1	
	less than 35	0	0	
inc	$100,000 and above	0	0	0
	40,000 - $99,999	1	0	0
	less than $40,000	0	1	0
	missing	0	0	1
edu	college degree or higher	0	0	
	missing	1	0	
	no college degree	0	1	

The GENMOD Procedure

Class Level Information				
**Class**	**Value**	**Design Variables**		
kids	missing	1	0	
	no	0	0	
	yes	0	1	
marital_status	married or living as married	1	0	0
	missing	0	1	0
	single	0	0	0
	widowed, divorced	0	0	1
feltunwell	missing	1	0	
	no	0	0	
	yes	0	1	
compare_others	good to excellent	0		
	poor or fair	1		
ratehealth	good to excellent	0		
	poor or fair	1		
x1	no	0		
	yes	1		
x2	no	0		
	yes	1		
x3	no	0		
	yes	1		
x4	no	0		
	yes	1		
x5	no	0		
	yes	1		

The GENMOD Procedure

Class Level Information				
Class	Value	Design Variables		
x6	no	0		
	yes	1		
x8	no	0		
	yes	1		
x9	no	0		
	yes	1		
x10	no	0		
	yes	1		
x11	no	0		
	yes	1		
x12	no	0		
	yes	1		
x13	no	0		
	yes	1		
x14	no	0		
	yes	1		
upset	do not want to answer	1	0	0
	missing	0	0	0
	no	0	1	0
	yes	0	0	1
hadajob	no	1		
	yes	0		
infected	no	0		
	yes	1		

Fully adjusted models of Anxietry score with gender*theme interaction

The GENMOD Procedure

Criteria For Assessing Goodness Of Fit			
Criterion	DF	Value	Value/DF
Deviance	1242	1502.4215	1.2097
Scaled Deviance	1242	1502.4215	1.2097
Pearson Chi-Square	1242	1283.0095	1.0330
Scaled Pearson X2	1242	1283.0095	1.0330
Log Likelihood		15323.3928	
Full Log Likelihood		-3619.2133	
AIC (smaller is better)		7342.4267	
AICC (smaller is better)		7346.8718	
BIC (smaller is better)		7610.9921	

Algorithm converged.

Analysis Of Maximum Likelihood Parameter Estimates									
Parameter			DF	Estimate	Standard Error	Wald 95% Confidence Limits		Wald Chi-Square	Pr > ChiSq
Intercept			1	2.2667	0.0830	2.1040	2.4294	745.54	<.0001
gender*x6	Male	yes	1	-0.1183	0.1033	-0.3207	0.0842	1.31	0.2523
gender*x6	Other	yes	1	0.1406	0.2582	-0.3655	0.6467	0.30	0.5862
x1	yes		1	0.0053	0.0323	-0.0580	0.0686	0.03	0.8692
x2	yes		1	0.1387	0.1601	-0.1752	0.4526	0.75	0.3864
x3	yes		1	-0.0689	0.0551	-0.1769	0.0392	1.56	0.2115
x4	yes		1	0.0674	0.0463	-0.0234	0.1582	2.12	0.1458
x5	yes		1	0.0781	0.0401	-0.0006	0.1567	3.78	0.0518
x6	yes		1	-0.0286	0.0479	-0.1225	0.0653	0.36	0.5505
x8	yes		1	0.0989	0.0392	0.0220	0.1757	6.36	0.0117
x9	yes		1	0.1715	0.1368	-0.0967	0.4397	1.57	0.2100

Fully adjusted models of Anxietry score with gender*theme interaction

The GENMOD Procedure

Analysis Of Maximum Likelihood Parameter Estimates									
Parameter			DF	Estimate	Standard Error	Wald 95% Confidence Limits		Wald Chi-Square	Pr > ChiSq
**x10**	yes		1	0.0703	0.0437	-0.0154	0.1560	2.59	0.1077
**x11**	yes		1	0.0166	0.1129	-0.2047	0.2378	0.02	0.8832
**x12**	yes		1	0.0042	0.1466	-0.2831	0.2915	0.00	0.9773
**x13**	yes		1	0.0949	0.0758	-0.0537	0.2435	1.57	0.2108
**x14**	yes		1	0.0589	0.1407	-0.2169	0.3347	0.18	0.6757
**upset**	do not want to answer		1	-0.0113	0.1560	-0.3169	0.2944	0.01	0.9424
**upset**	no		1	-0.0024	0.0312	-0.0634	0.0587	0.01	0.9397
**upset**	yes		1	0.1658	0.0478	0.0721	0.2595	12.03	0.0005
**dur**			1	0.0017	0.0005	0.0008	0.0026	13.63	0.0002
**support_family**			1	-0.0021	0.0005	-0.0031	-0.0012	18.65	<.0001
**support_doctor**			1	-0.0004	0.0005	-0.0013	0.0006	0.52	0.4708
**support_fedgovt**			1	-0.0008	0.0007	-0.0021	0.0005	1.38	0.2400
**support_city**			1	0.0010	0.0007	-0.0004	0.0024	1.85	0.1734
**support_deptpubhealt**			1	0.0001	0.0007	-0.0012	0.0015	0.04	0.8368
**support_faithcommuni**			1	-0.0007	0.0005	-0.0016	0.0003	1.71	0.1912
**support_socialservic**			1	0.0011	0.0007	-0.0002	0.0025	2.82	0.0933
**support_neighbors**			1	-0.0005	0.0005	-0.0015	0.0005	1.02	0.3136
**support_other**			1	-0.0003	0.0006	-0.0015	0.0009	0.21	0.6501
**infected**	yes		1	0.0440	0.1157	-0.1827	0.2706	0.14	0.7040
**feltunwell**	missing		1	-0.0990	0.2345	-0.5587	0.3607	0.18	0.6729
**feltunwell**	yes		1	0.0582	0.0289	0.0015	0.1148	4.05	0.0442
**compare_others**	poor or fair		1	0.1271	0.0464	0.0361	0.2181	7.50	0.0062
**ratehealth**	poor or fair		1	0.0323	0.0623	-0.0898	0.1544	0.27	0.6041
**gender**	Male		1	-0.1538	0.0305	-0.2135	-0.0941	25.47	<.0001
**gender**	Other		1	0.0027	0.1107	-0.2144	0.2197	0.00	0.9809

Fully adjusted models of Anxietry score with gender*theme interaction

The GENMOD Procedure

Analysis Of Maximum Likelihood Parameter Estimates									
Parameter			DF	Estimate	Standard Error	Wald 95% Confidence Limits		Wald Chi-Square	Pr > ChiSq
race	black		1	-0.1309	0.0510	-0.2308	-0.0310	6.60	0.0102
race	other race		1	-0.0928	0.0485	-0.1878	0.0023	3.66	0.0557
age_cat	35-54		1	-0.0309	0.0374	-0.1042	0.0425	0.68	0.4092
age_cat	greater than 55		1	-0.1987	0.0406	-0.2782	-0.1191	23.97	<.0001
inc	40,000 - $99,999		1	0.0746	0.0306	0.0145	0.1346	5.93	0.0149
inc	less than $40,000		1	0.1378	0.0391	0.0613	0.2144	12.45	0.0004
inc	missing		1	0.0660	0.0807	-0.0921	0.2242	0.67	0.4131
edu	missing		1	-0.1874	0.1870	-0.5540	0.1792	1.00	0.3164
edu	no college degree		1	0.0115	0.0363	-0.0597	0.0827	0.10	0.7515
marital_status	married or living as married		1	0.0955	0.0316	0.0336	0.1575	9.14	0.0025
marital_status	missing		1	0.3899	0.1543	0.0873	0.6924	6.38	0.0115
marital_status	widowed, divorced		1	-0.0079	0.0451	-0.0963	0.0804	0.03	0.8601
kids	missing		1	-0.3062	0.1872	-0.6731	0.0608	2.67	0.1020
kids	yes		1	-0.0165	0.0354	-0.0860	0.0529	0.22	0.6412
hadajob	no		1	-0.0544	0.0330	-0.1190	0.0102	2.72	0.0991
Dispersion			1	0.0746	0.0078	0.0608	0.0916		

Note: The negative binomial dispersion parameter was estimated by maximum likelihood.

LR Statistics For Joint Tests			
Source	DF	Chi-Square	Pr > ChiSq
gender*x6	2	1.74	0.4196
x1	1	0.03	0.8692
x2	1	0.75	0.3878
x3	1	1.56	0.2112
x4	1	2.11	0.1465

The GENMOD Procedure

LR Statistics For Joint Tests			
Source	DF	Chi-Square	Pr > ChiSq
x5	1	3.77	0.0523
x6	1	0.36	0.5503
x8	1	6.33	0.0119
x9	1	1.56	0.2111
x10	1	2.58	0.1084
x11	1	0.02	0.8833
x12	1	0.00	0.9773
x13	1	1.56	0.2117
x14	1	0.17	0.6763
upset	3	16.62	0.0008
dur	1	13.56	0.0002
support_family	1	18.49	<.0001
support_doctor	1	0.52	0.4709
support_fedgovt	1	1.38	0.2399
support_city	1	1.85	0.1734
support_deptpubhealt	1	0.04	0.8368
support_faithcommuni	1	1.71	0.1912
support_socialservic	1	2.81	0.0936
support_neighbors	1	1.02	0.3135
support_other	1	0.21	0.6501
infected	1	0.14	0.7042
feltunwell	2	4.29	0.1173
compare_others	1	7.45	0.0063
ratehealth	1	0.27	0.6042
gender	2	25.45	<.0001
race	2	9.52	0.0086

The GENMOD Procedure

LR Statistics For Joint Tests			
**Source**	**DF**	**Chi-Square**	**Pr > ChiSq**
**age_cat**	2	34.17	<.0001
**inc**	3	12.62	0.0055
**edu**	2	1.15	0.5638
**marital_status**	3	17.06	0.0007
**kids**	2	2.88	0.2370
**hadajob**	1	2.72	0.0991

Note: Under full-rank parameterizations, Type 3 effect tests are replaced by joint tests. The joint test for an effect is a test that all the parameters associated with that effect are zero. Such joint tests might not be equivalent to Type 3 effect tests under GLM parameterization.

The GENMOD Procedure

Model Information		
**Data Set**	WORK.Z	
**Distribution**	Negative Binomial	
**Link Function**	Log	
**Dependent Variable**	AscoreR	HADS Anxiety score

**Number of Observations Read**	1293
**Number of Observations Used**	1293

Class Level Information				
**Class**	**Value**	**Design Variables**		
**gender**	**Female**	0	0	
	**Male**	1	0	
	**Other**	0	1	
**race**	**black**	1	0	
	**other race**	0	1	
	**white**	0	0	
**age_cat**	**35-54**	1	0	
	**greater than 55**	0	1	
	**less than 35**	0	0	
**inc**	**$100,000 and above**	0	0	0
	**40,000 - $99,999**	1	0	0
	**less than $40,000**	0	1	0
	**missing**	0	0	1
**edu**	**college degree or higher**	0	0	
	**missing**	1	0	
	**no college degree**	0	1	

Fully adjusted models of Anxietry score with gender*theme interaction

The GENMOD Procedure

Class Level Information				
**Class**	**Value**	**Design Variables**		
kids	missing	1	0	
	no	0	0	
	yes	0	1	
marital_status	married or living as married	1	0	0
	missing	0	1	0
	single	0	0	0
	widowed, divorced	0	0	1
feltunwell	missing	1	0	
	no	0	0	
	yes	0	1	
compare_others	good to excellent	0		
	poor or fair	1		
ratehealth	good to excellent	0		
	poor or fair	1		
x1	no	0		
	yes	1		
x2	no	0		
	yes	1		
x3	no	0		
	yes	1		
x4	no	0		
	yes	1		
x5	no	0		
	yes	1		

The GENMOD Procedure

Class Level Information				
**Class**	**Value**	**Design Variables**		
**x6**	**no**	0		
	**yes**	1		
**x8**	**no**	0		
	**yes**	1		
**x9**	**no**	0		
	**yes**	1		
**x10**	**no**	0		
	**yes**	1		
**x11**	**no**	0		
	**yes**	1		
**x12**	**no**	0		
	**yes**	1		
**x13**	**no**	0		
	**yes**	1		
**x14**	**no**	0		
	**yes**	1		
**upset**	**do not want to answer**	1	0	0
	**missing**	0	0	0
	**no**	0	1	0
	**yes**	0	0	1
**hadajob**	**no**	1		
	**yes**	0		
**infected**	**no**	0		
	**yes**	1		

Fully adjusted models of Anxietry score with gender*theme interaction

The GENMOD Procedure

Criteria For Assessing Goodness Of Fit			
**Criterion**	**DF**	**Value**	**Value/DF**
**Deviance**	1242	1503.4099	1.2105
**Scaled Deviance**	1242	1503.4099	1.2105
**Pearson Chi-Square**	1242	1285.8471	1.0353
**Scaled Pearson X2**	1242	1285.8471	1.0353
**Log Likelihood**		15324.9215	
**Full Log Likelihood**		-3617.6847	
**AIC (smaller is better)**		7339.3693	
**AICC (smaller is better)**		7343.8145	
**BIC (smaller is better)**		7607.9348	

Algorithm converged.

Analysis Of Maximum Likelihood Parameter Estimates									
**Parameter**			**DF**	**Estimate**	**Standard Error**	**Wald 95% Confidence Limits**		**Wald Chi-Square**	**Pr > ChiSq**
**Intercept**			1	2.2585	0.0829	2.0961	2.4209	743.01	<.0001
**gender*x8**	Male	yes	1	-0.1719	0.1095	-0.3866	0.0428	2.46	0.1166
**gender*x8**	Other	yes	1	-0.3983	0.2449	-0.8784	0.0818	2.64	0.1039
**x1**	yes		1	0.0066	0.0322	-0.0566	0.0698	0.04	0.8378
**x2**	yes		1	0.1440	0.1598	-0.1693	0.4573	0.81	0.3676
**x3**	yes		1	-0.0663	0.0550	-0.1742	0.0415	1.45	0.2279
**x4**	yes		1	0.0734	0.0463	-0.0174	0.1642	2.51	0.1132
**x5**	yes		1	0.0757	0.0401	-0.0029	0.1543	3.57	0.0590
**x6**	yes		1	-0.0532	0.0422	-0.1360	0.0295	1.59	0.2074
**x8**	yes		1	0.1339	0.0426	0.0504	0.2173	9.88	0.0017
**x9**	yes		1	0.1665	0.1367	-0.1013	0.4344	1.49	0.2230

The GENMOD Procedure

Analysis Of Maximum Likelihood Parameter Estimates									
Parameter			DF	Estimate	Standard Error	Wald 95% Confidence Limits		Wald Chi-Square	Pr > ChiSq
x10	yes		1	0.0698	0.0437	-0.0158	0.1554	2.55	0.1102
x11	yes		1	0.0147	0.1127	-0.2062	0.2357	0.02	0.8960
x12	yes		1	-0.0033	0.1463	-0.2901	0.2834	0.00	0.9819
x13	yes		1	0.1048	0.0759	-0.0440	0.2537	1.91	0.1675
x14	yes		1	0.0497	0.1406	-0.2258	0.3252	0.12	0.7238
upset	do not want to answer		1	0.0159	0.1568	-0.2914	0.3232	0.01	0.9194
upset	no		1	-0.0013	0.0311	-0.0623	0.0596	0.00	0.9654
upset	yes		1	0.1672	0.0477	0.0737	0.2607	12.28	0.0005
dur			1	0.0017	0.0005	0.0008	0.0026	14.45	0.0001
support_family			1	-0.0021	0.0005	-0.0030	-0.0011	18.01	<.0001
support_doctor			1	-0.0004	0.0005	-0.0013	0.0006	0.56	0.4546
support_fedgovt			1	-0.0008	0.0007	-0.0021	0.0005	1.42	0.2327
support_city			1	0.0011	0.0007	-0.0004	0.0025	2.14	0.1437
support_deptpubhealt			1	0.0000	0.0007	-0.0013	0.0014	0.00	0.9464
support_faithcommuni			1	-0.0007	0.0005	-0.0017	0.0003	2.06	0.1512
support_socialservic			1	0.0012	0.0007	-0.0001	0.0025	3.13	0.0768
support_neighbors			1	-0.0006	0.0005	-0.0016	0.0004	1.35	0.2448
support_other			1	-0.0002	0.0006	-0.0015	0.0010	0.15	0.6968
infected	yes		1	0.0432	0.1155	-0.1832	0.2696	0.14	0.7085
feltunwell	missing		1	-0.0938	0.2342	-0.5527	0.3652	0.16	0.6888
feltunwell	yes		1	0.0602	0.0289	0.0035	0.1169	4.33	0.0373
compare_others	poor or fair		1	0.1270	0.0463	0.0363	0.2178	7.52	0.0061
ratehealth	poor or fair		1	0.0246	0.0623	-0.0975	0.1466	0.16	0.6932
gender	Male		1	-0.1498	0.0304	-0.2093	-0.0902	24.31	<.0001
gender	Other		1	0.1071	0.1110	-0.1105	0.3247	0.93	0.3346

The GENMOD Procedure

Analysis Of Maximum Likelihood Parameter Estimates									
Parameter			DF	Estimate	Standard Error	Wald 95% Confidence Limits		Wald Chi-Square	Pr > ChiSq
race	black		1	-0.1293	0.0509	-0.2290	-0.0296	6.46	0.0110
race	other race		1	-0.0960	0.0484	-0.1909	-0.0011	3.93	0.0473
age_cat	35-54		1	-0.0306	0.0374	-0.1038	0.0426	0.67	0.4127
age_cat	greater than 55		1	-0.1963	0.0405	-0.2757	-0.1169	23.49	<.0001
inc	40,000 - $99,999		1	0.0766	0.0305	0.0168	0.1365	6.29	0.0121
inc	less than $40,000		1	0.1367	0.0390	0.0602	0.2131	12.27	0.0005
inc	missing		1	0.0658	0.0806	-0.0922	0.2237	0.67	0.4144
edu	missing		1	-0.2004	0.1866	-0.5661	0.1652	1.15	0.2827
edu	no college degree		1	0.0102	0.0363	-0.0608	0.0813	0.08	0.7777
marital_status	married or living as married		1	0.0934	0.0316	0.0316	0.1553	8.77	0.0031
marital_status	missing		1	0.3862	0.1541	0.0842	0.6883	6.28	0.0122
marital_status	widowed, divorced		1	-0.0099	0.0450	-0.0981	0.0783	0.05	0.8261
kids	missing		1	-0.2824	0.1875	-0.6498	0.0850	2.27	0.1320
kids	yes		1	-0.0148	0.0354	-0.0842	0.0546	0.18	0.6757
hadajob	no		1	-0.0524	0.0329	-0.1169	0.0121	2.53	0.1115
Dispersion			1	0.0741	0.0078	0.0603	0.0910		

Note: The negative binomial dispersion parameter was estimated by maximum likelihood.

LR Statistics For Joint Tests			
Source	DF	Chi-Square	Pr > ChiSq
gender*x8	2	4.79	0.0910
x1	1	0.04	0.8378
x2	1	0.81	0.3691
x3	1	1.46	0.2277
x4	1	2.50	0.1140

The GENMOD Procedure

LR Statistics For Joint Tests			
Source	DF	Chi-Square	Pr > ChiSq
x5	1	3.55	0.0595
x6	1	1.59	0.2069
x8	1	9.80	0.0017
x9	1	1.48	0.2241
x10	1	2.54	0.1108
x11	1	0.02	0.8961
x12	1	0.00	0.9819
x13	1	1.90	0.1684
x14	1	0.12	0.7242
upset	3	16.79	0.0008
dur	1	14.37	0.0002
support_family	1	17.86	<.0001
support_doctor	1	0.56	0.4546
support_fedgovt	1	1.43	0.2325
support_city	1	2.14	0.1437
support_deptpubhealt	1	0.00	0.9464
support_faithcommuni	1	2.06	0.1513
support_socialservic	1	3.13	0.0771
support_neighbors	1	1.35	0.2448
support_other	1	0.15	0.6969
infected	1	0.14	0.7087
feltunwell	2	4.55	0.1028
compare_others	1	7.48	0.0062
ratehealth	1	0.16	0.6933
gender	2	25.85	<.0001
race	2	9.63	0.0081

The GENMOD Procedure

LR Statistics For Joint Tests			
**Source**	**DF**	**Chi-Square**	**Pr > ChiSq**
**age_cat**	2	33.52	<.0001
**inc**	3	12.56	0.0057
**edu**	2	1.28	0.5273
**marital_status**	3	16.70	0.0008
**kids**	2	2.44	0.2959
**hadajob**	1	2.53	0.1115

Note: Under full-rank parameterizations, Type 3 effect tests are replaced by joint tests. The joint test for an effect is a test that all the parameters associated with that effect are zero. Such joint tests might not be equivalent to Type 3 effect tests under GLM parameterization.

The GENMOD Procedure

Model Information		
**Data Set**	WORK.Z	
**Distribution**	Negative Binomial	
**Link Function**	Log	
**Dependent Variable**	AscoreR	HADS Anxiety score

**Number of Observations Read**	1293
**Number of Observations Used**	1293

Class Level Information				
**Class**	**Value**	**Design Variables**		
**gender**	**Female**	0	0	
	**Male**	1	0	
	**Other**	0	1	
**race**	**black**	1	0	
	**other race**	0	1	
	**white**	0	0	
**age_cat**	**35-54**	1	0	
	**greater than 55**	0	1	
	**less than 35**	0	0	
**inc**	**$100,000 and above**	0	0	0
	**40,000 - $99,999**	1	0	0
	**less than $40,000**	0	1	0
	**missing**	0	0	1
**edu**	**college degree or higher**	0	0	
	**missing**	1	0	
	**no college degree**	0	1	

Fully adjusted models of Anxietry score with gender*theme interaction

The GENMOD Procedure

Class Level Information				
**Class**	**Value**	**Design Variables**		
kids	missing	1	0	
	no	0	0	
	yes	0	1	
marital_status	married or living as married	1	0	0
	missing	0	1	0
	single	0	0	0
	widowed, divorced	0	0	1
feltunwell	missing	1	0	
	no	0	0	
	yes	0	1	
compare_others	good to excellent	0		
	poor or fair	1		
ratehealth	good to excellent	0		
	poor or fair	1		
x1	no	0		
	yes	1		
x2	no	0		
	yes	1		
x3	no	0		
	yes	1		
x4	no	0		
	yes	1		
x5	no	0		
	yes	1		

The GENMOD Procedure

Class Level Information				
Class	Value	Design Variables		
x6	no	0		
	yes	1		
x8	no	0		
	yes	1		
x9	no	0		
	yes	1		
x10	no	0		
	yes	1		
x11	no	0		
	yes	1		
x12	no	0		
	yes	1		
x13	no	0		
	yes	1		
x14	no	0		
	yes	1		
upset	do not want to answer	1	0	0
	missing	0	0	0
	no	0	1	0
	yes	0	0	1
hadajob	no	1		
	yes	0		
infected	no	0		
	yes	1		

Fully adjusted models of Anxietry score with gender*theme interaction

The GENMOD Procedure

Criteria For Assessing Goodness Of Fit			
**Criterion**	**DF**	**Value**	**Value/DF**
**Deviance**	1243	1502.4888	1.2088
**Scaled Deviance**	1243	1502.4888	1.2088
**Pearson Chi-Square**	1243	1284.1789	1.0331
**Scaled Pearson X2**	1243	1284.1789	1.0331
**Log Likelihood**		15322.5655	
**Full Log Likelihood**		-3620.0406	
**AIC (smaller is better)**		7342.0812	
**AICC (smaller is better)**		7346.3551	
**BIC (smaller is better)**		7605.4819	

Algorithm converged.

Analysis Of Maximum Likelihood Parameter Estimates									
**Parameter**			**DF**	**Estimate**	**Standard Error**	**Wald 95% Confidence Limits**		**Wald Chi-Square**	**Pr > ChiSq**
**Intercept**			1	2.2636	0.0831	2.1007	2.4264	741.85	<.0001
**gender*x9**	Male	yes	1	0.0969	0.3374	-0.5644	0.7582	0.08	0.7739
**gender*x9**	Other	yes	0	0.0000	0.0000	0.0000	0.0000	.	.
**x1**	yes		1	0.0059	0.0323	-0.0574	0.0693	0.03	0.8550
**x2**	yes		1	0.1427	0.1602	-0.1714	0.4567	0.79	0.3733
**x3**	yes		1	-0.0666	0.0551	-0.1747	0.0415	1.46	0.2273
**x4**	yes		1	0.0684	0.0464	-0.0225	0.1593	2.18	0.1401
**x5**	yes		1	0.0783	0.0402	-0.0004	0.1571	3.80	0.0513
**x6**	yes		1	-0.0488	0.0423	-0.1316	0.0341	1.33	0.2485
**x8**	yes		1	0.0999	0.0392	0.0231	0.1767	6.50	0.0108
**x9**	yes		1	0.1525	0.1536	-0.1485	0.4536	0.99	0.3206

The GENMOD Procedure

Analysis Of Maximum Likelihood Parameter Estimates									
Parameter			DF	Estimate	Standard Error	Wald 95% Confidence Limits		Wald Chi-Square	Pr > ChiSq
x10	yes		1	0.0704	0.0438	-0.0154	0.1563	2.59	0.1078
x11	yes		1	0.0190	0.1129	-0.2023	0.2404	0.03	0.8661
x12	yes		1	0.0046	0.1465	-0.2826	0.2917	0.00	0.9751
x13	yes		1	0.0961	0.0759	-0.0526	0.2449	1.60	0.2053
x14	yes		1	0.0570	0.1408	-0.2190	0.3330	0.16	0.6857
upset	do not want to answer		1	-0.0229	0.1555	-0.3277	0.2818	0.02	0.8828
upset	no		1	-0.0028	0.0312	-0.0639	0.0583	0.01	0.9290
upset	yes		1	0.1667	0.0478	0.0730	0.2604	12.15	0.0005
dur			1	0.0017	0.0005	0.0008	0.0026	13.80	0.0002
support_family			1	-0.0021	0.0005	-0.0031	-0.0011	18.36	<.0001
support_doctor			1	-0.0004	0.0005	-0.0013	0.0006	0.51	0.4772
support_fedgovt			1	-0.0008	0.0007	-0.0021	0.0005	1.41	0.2358
support_city			1	0.0010	0.0007	-0.0004	0.0024	1.89	0.1697
support_deptpubhealt			1	0.0001	0.0007	-0.0012	0.0015	0.04	0.8321
support_faithcommuni			1	-0.0007	0.0005	-0.0017	0.0003	1.83	0.1767
support_socialservic			1	0.0011	0.0007	-0.0002	0.0025	2.76	0.0964
support_neighbors			1	-0.0005	0.0005	-0.0015	0.0005	1.10	0.2939
support_other			1	-0.0003	0.0006	-0.0015	0.0010	0.16	0.6859
infected	yes		1	0.0445	0.1157	-0.1824	0.2713	0.15	0.7009
feltunwell	missing		1	-0.0995	0.2347	-0.5594	0.3604	0.18	0.6715
feltunwell	yes		1	0.0579	0.0289	0.0012	0.1146	4.01	0.0454
compare_others	poor or fair		1	0.1275	0.0464	0.0365	0.2185	7.54	0.0060
ratehealth	poor or fair		1	0.0323	0.0623	-0.0899	0.1544	0.27	0.6045
gender	Male		1	-0.1641	0.0294	-0.2217	-0.1064	31.11	<.0001
gender	Other		1	0.0298	0.1008	-0.1678	0.2274	0.09	0.7675

Fully adjusted models of Anxietry score with gender*theme interaction

The GENMOD Procedure

Analysis Of Maximum Likelihood Parameter Estimates									
Parameter			DF	Estimate	Standard Error	Wald 95% Confidence Limits		Wald Chi-Square	Pr > ChiSq
race	black		1	-0.1290	0.0510	-0.2289	-0.0290	6.40	0.0114
race	other race		1	-0.0936	0.0485	-0.1886	0.0015	3.72	0.0538
age_cat	35-54		1	-0.0306	0.0375	-0.1041	0.0430	0.66	0.4154
age_cat	greater than 55		1	-0.1974	0.0406	-0.2770	-0.1178	23.65	<.0001
inc	40,000 - $99,999		1	0.0772	0.0306	0.0172	0.1371	6.36	0.0117
inc	less than $40,000		1	0.1396	0.0391	0.0630	0.2162	12.77	0.0004
inc	missing		1	0.0677	0.0807	-0.0905	0.2260	0.70	0.4015
edu	missing		1	-0.1946	0.1869	-0.5609	0.1716	1.08	0.2976
edu	no college degree		1	0.0118	0.0363	-0.0594	0.0830	0.11	0.7457
marital_status	married or living as married		1	0.0950	0.0316	0.0330	0.1569	9.02	0.0027
marital_status	missing		1	0.3932	0.1544	0.0905	0.6958	6.48	0.0109
marital_status	widowed, divorced		1	-0.0095	0.0451	-0.0979	0.0789	0.04	0.8333
kids	missing		1	-0.3024	0.1873	-0.6695	0.0647	2.61	0.1064
kids	yes		1	-0.0167	0.0355	-0.0864	0.0529	0.22	0.6378
hadajob	no		1	-0.0533	0.0330	-0.1180	0.0113	2.61	0.1060
Dispersion			1	0.0749	0.0078	0.0610	0.0919		

Note: The negative binomial dispersion parameter was estimated by maximum likelihood.

LR Statistics For Joint Tests			
Source	DF	Chi-Square	Pr > ChiSq
gender*x9	2	2.10	0.3495
x1	1	0.03	0.8550
x2	1	0.79	0.3748
x3	1	1.46	0.2271
x4	1	2.17	0.1408

The GENMOD Procedure

LR Statistics For Joint Tests			
Source	DF	Chi-Square	Pr > ChiSq
x5	1	3.78	0.0517
x6	1	1.33	0.2481
x8	1	6.47	0.0110
x9	1	0.98	0.3213
x10	1	2.58	0.1085
x11	1	0.03	0.8662
x12	1	0.00	0.9751
x13	1	1.60	0.2062
x14	1	0.16	0.6862
upset	3	16.91	0.0007
dur	1	13.73	0.0002
support_family	1	18.20	<.0001
support_doctor	1	0.51	0.4772
support_fedgovt	1	1.41	0.2357
support_city	1	1.89	0.1697
support_deptpubhealt	1	0.04	0.8321
support_faithcommuni	1	1.83	0.1767
support_socialservic	1	2.76	0.0967
support_neighbors	1	1.10	0.2939
support_other	1	0.16	0.6859
infected	1	0.15	0.7011
feltunwell	2	4.24	0.1199
compare_others	1	7.50	0.0062
ratehealth	1	0.27	0.6046
gender	2	31.30	<.0001
race	2	9.38	0.0092

Fully adjusted models of Anxiety score with gender*theme interaction

The GENMOD Procedure

LR Statistics For Joint Tests			
**Source**	**DF**	**Chi-Square**	**Pr > ChiSq**
**age_cat**	2	33.75	<.0001
**inc**	3	13.02	0.0046
**edu**	2	1.24	0.5391
**marital_status**	3	17.15	0.0007
**kids**	2	2.81	0.2448
**hadajob**	1	2.61	0.1060

Note: Under full-rank parameterizations, Type 3 effect tests are replaced by joint tests. The joint test for an effect is a test that all the parameters associated with that effect are zero. Such joint tests might not be equivalent to Type 3 effect tests under GLM parameterization.

The GENMOD Procedure

Model Information		
Data Set	WORK.Z	
Distribution	Negative Binomial	
Link Function	Log	
Dependent Variable	AscoreR	HADS Anxiety score

Number of Observations Read	1293
Number of Observations Used	1293

Class Level Information				
Class	Value	Design Variables		
gender	Female	0	0	
	Male	1	0	
	Other	0	1	
race	black	1	0	
	other race	0	1	
	white	0	0	
age_cat	35-54	1	0	
	greater than 55	0	1	
	less than 35	0	0	
inc	$100,000 and above	0	0	0
	40,000 - $99,999	1	0	0
	less than $40,000	0	1	0
	missing	0	0	1
edu	college degree or higher	0	0	
	missing	1	0	
	no college degree	0	1	

The GENMOD Procedure

Class Level Information				
Class	Value	Design Variables		
kids	missing	1	0	
	no	0	0	
	yes	0	1	
marital_status	married or living as married	1	0	0
	missing	0	1	0
	single	0	0	0
	widowed, divorced	0	0	1
feltunwell	missing	1	0	
	no	0	0	
	yes	0	1	
compare_others	good to excellent	0		
	poor or fair	1		
ratehealth	good to excellent	0		
	poor or fair	1		
x1	no	0		
	yes	1		
x2	no	0		
	yes	1		
x3	no	0		
	yes	1		
x4	no	0		
	yes	1		
x5	no	0		
	yes	1		

The GENMOD Procedure

Class Level Information				
Class	Value	Design Variables		
x6	no	0		
	yes	1		
x8	no	0		
	yes	1		
x9	no	0		
	yes	1		
x10	no	0		
	yes	1		
x11	no	0		
	yes	1		
x12	no	0		
	yes	1		
x13	no	0		
	yes	1		
x14	no	0		
	yes	1		
upset	do not want to answer	1	0	0
	missing	0	0	0
	no	0	1	0
	yes	0	0	1
hadajob	no	1		
	yes	0		
infected	no	0		
	yes	1		

Fully adjusted models of Anxietry score with gender*theme interaction

The GENMOD Procedure

Criteria For Assessing Goodness Of Fit			
Criterion	DF	Value	Value/DF
Deviance	1242	1503.6561	1.2107
Scaled Deviance	1242	1503.6561	1.2107
Pearson Chi-Square	1242	1286.8772	1.0361
Scaled Pearson X2	1242	1286.8772	1.0361
Log Likelihood		15326.4017	
Full Log Likelihood		-3616.2045	
AIC (smaller is better)		7336.4089	
AICC (smaller is better)		7340.8541	
BIC (smaller is better)		7604.9744	

Algorithm converged.

Analysis Of Maximum Likelihood Parameter Estimates									
Parameter			DF	Estimate	Standard Error	Wald 95% Confidence Limits		Wald Chi-Square	Pr > ChiSq
Intercept			1	2.2712	0.0828	2.1090	2.4335	752.69	<.0001
gender*x10	Male	yes	1	-0.0383	0.0997	-0.2337	0.1571	0.15	0.7008
gender*x10	Other	yes	1	-1.0587	0.4034	-1.8494	-0.2679	6.89	0.0087
x1	yes		1	0.0091	0.0322	-0.0541	0.0722	0.08	0.7785
x2	yes		1	0.1396	0.1597	-0.1734	0.4527	0.76	0.3820
x3	yes		1	-0.0672	0.0549	-0.1749	0.0405	1.50	0.2213
x4	yes		1	0.0674	0.0462	-0.0232	0.1579	2.12	0.1449
x5	yes		1	0.0756	0.0401	-0.0030	0.1542	3.56	0.0593
x6	yes		1	-0.0519	0.0421	-0.1345	0.0307	1.52	0.2183
x8	yes		1	0.1035	0.0391	0.0269	0.1801	7.01	0.0081
x9	yes		1	0.1742	0.1366	-0.0935	0.4418	1.63	0.2022

Fully adjusted models of Anxietry score with gender*theme interaction

The GENMOD Procedure

Analysis Of Maximum Likelihood Parameter Estimates									
Parameter			DF	Estimate	Standard Error	Wald 95% Confidence Limits		Wald Chi-Square	Pr > ChiSq
x10	yes		1	0.0954	0.0504	-0.0034	0.1941	3.58	0.0584
x11	yes		1	0.0184	0.1126	-0.2022	0.2390	0.03	0.8703
x12	yes		1	-0.0086	0.1462	-0.2951	0.2780	0.00	0.9533
x13	yes		1	0.0949	0.0756	-0.0533	0.2430	1.57	0.2096
x14	yes		1	0.0570	0.1403	-0.2181	0.3320	0.16	0.6849
upset	do not want to answer		1	-0.0547	0.1556	-0.3597	0.2503	0.12	0.7253
upset	no		1	-0.0058	0.0311	-0.0667	0.0551	0.03	0.8518
upset	yes		1	0.1646	0.0477	0.0712	0.2580	11.93	0.0006
dur			1	0.0017	0.0005	0.0008	0.0026	13.18	0.0003
support_family			1	-0.0021	0.0005	-0.0030	-0.0011	17.77	<.0001
support_doctor			1	-0.0003	0.0005	-0.0013	0.0006	0.48	0.4888
support_fedgovt			1	-0.0008	0.0007	-0.0021	0.0005	1.35	0.2444
support_city			1	0.0010	0.0007	-0.0004	0.0024	1.82	0.1776
support_deptpubhealt			1	0.0002	0.0007	-0.0011	0.0015	0.07	0.7881
support_faithcommuni			1	-0.0007	0.0005	-0.0017	0.0003	2.01	0.1562
support_socialservic			1	0.0011	0.0007	-0.0003	0.0024	2.52	0.1122
support_neighbors			1	-0.0005	0.0005	-0.0015	0.0005	0.98	0.3222
support_other			1	-0.0003	0.0006	-0.0015	0.0009	0.23	0.6340
infected	yes		1	0.0457	0.1153	-0.1802	0.2717	0.16	0.6917
feltunwell	missing		1	-0.0973	0.2339	-0.5558	0.3612	0.17	0.6774
feltunwell	yes		1	0.0552	0.0288	-0.0013	0.1117	3.67	0.0554
compare_others	poor or fair		1	0.1237	0.0463	0.0331	0.2144	7.15	0.0075
ratehealth	poor or fair		1	0.0358	0.0621	-0.0858	0.1575	0.33	0.5637
gender	Male		1	-0.1597	0.0305	-0.2195	-0.0999	27.37	<.0001
gender	Other		1	0.1099	0.1044	-0.0948	0.3146	1.11	0.2927

The GENMOD Procedure

Analysis Of Maximum Likelihood Parameter Estimates									
Parameter			DF	Estimate	Standard Error	Wald 95% Confidence Limits		Wald Chi-Square	Pr > ChiSq
**race**	black		1	-0.1303	0.0508	-0.2300	-0.0307	6.57	0.0104
**race**	other race		1	-0.0953	0.0484	-0.1901	-0.0005	3.88	0.0488
**age_cat**	35-54		1	-0.0345	0.0373	-0.1076	0.0386	0.86	0.3549
**age_cat**	greater than 55		1	-0.1985	0.0404	-0.2778	-0.1192	24.09	<.0001
**inc**	40,000 - $99,999		1	0.0757	0.0305	0.0159	0.1355	6.16	0.0131
**inc**	less than $40,000		1	0.1416	0.0390	0.0653	0.2180	13.21	0.0003
**inc**	missing		1	0.0669	0.0805	-0.0909	0.2246	0.69	0.4060
**edu**	missing		1	-0.1903	0.1866	-0.5560	0.1754	1.04	0.3077
**edu**	no college degree		1	0.0121	0.0362	-0.0589	0.0831	0.11	0.7382
**marital_status**	married or living as married		1	0.0917	0.0315	0.0298	0.1535	8.45	0.0037
**marital_status**	missing		1	0.3911	0.1538	0.0896	0.6926	6.46	0.0110
**marital_status**	widowed, divorced		1	-0.0101	0.0449	-0.0981	0.0780	0.05	0.8228
**kids**	missing		1	-0.3022	0.1868	-0.6683	0.0638	2.62	0.1056
**kids**	yes		1	-0.0152	0.0353	-0.0844	0.0541	0.18	0.6676
**hadajob**	no		1	-0.0549	0.0329	-0.1193	0.0095	2.79	0.0950
**Dispersion**			1	0.0736	0.0078	0.0598	0.0905		

Note: The negative binomial dispersion parameter was estimated by maximum likelihood.

LR Statistics For Joint Tests			
Source	DF	Chi-Square	Pr > ChiSq
**gender*x10**	2	7.75	0.0207
**x1**	1	0.08	0.7785
**x2**	1	0.76	0.3835
**x3**	1	1.50	0.2211
**x4**	1	2.12	0.1456

Fully adjusted models of Anxietry score with gender*theme interaction

The GENMOD Procedure

LR Statistics For Joint Tests			
**Source**	**DF**	**Chi-Square**	**Pr > ChiSq**
**x5**	1	3.54	0.0598
**x6**	1	1.52	0.2178
**x8**	1	6.96	0.0083
**x9**	1	1.62	0.2034
**x10**	1	3.57	0.0590
**x11**	1	0.03	0.8703
**x12**	1	0.00	0.9532
**x13**	1	1.57	0.2105
**x14**	1	0.16	0.6854
**upset**	3	17.22	0.0006
**dur**	1	13.12	0.0003
**support_family**	1	17.62	<.0001
**support_doctor**	1	0.48	0.4888
**support_fedgovt**	1	1.36	0.2443
**support_city**	1	1.82	0.1776
**support_deptpubhealt**	1	0.07	0.7882
**support_faithcommuni**	1	2.01	0.1562
**support_socialservic**	1	2.52	0.1125
**support_neighbors**	1	0.98	0.3222
**support_other**	1	0.23	0.6341
**infected**	1	0.16	0.6920
**feltunwell**	2	3.90	0.1425
**compare_others**	1	7.11	0.0077
**ratehealth**	1	0.33	0.5638
**gender**	2	29.09	<.0001
**race**	2	9.68	0.0079

Fully adjusted models of Anxiety score with gender*theme interaction

The GENMOD Procedure

LR Statistics For Joint Tests

Source	DF	Chi-Square	Pr > ChiSq
age_cat	2	33.55	<.0001
inc	3	13.36	0.0039
edu	2	1.20	0.5494
marital_status	3	16.44	0.0009
kids	2	2.80	0.2469
hadajob	1	2.79	0.0951

Note: Under full-rank parameterizations, Type 3 effect tests are replaced by joint tests. The joint test for an effect is a test that all the parameters associated with that effect are zero. Such joint tests might not be equivalent to Type 3 effect tests under GLM parameterization.

The MEANS Procedure

Analysis Variable : AscoreR HADS Anxiety score							
Gender	uncertainty	N Obs	N	Mean	Std Dev	Minimum	Maximum
Female	no	873	873	9.6219931	4.2452426	0	21.0000000
	yes	76	76	10.8947368	3.5045835	1.0000000	18.0000000
Male	no	297	297	8.1649832	4.2865739	0	20.0000000
	yes	29	29	8.5172414	4.2562617	0	18.0000000
Other	no	16	16	12.6250000	4.2875790	7.0000000	20.0000000
	yes	2	2	4.5000000	0.7071068	4.0000000	5.0000000

The GENMOD Procedure

Model Information		
**Data Set**	WORK.Z	
**Distribution**	Negative Binomial	
**Link Function**	Log	
**Dependent Variable**	AscoreR	HADS Anxiety score

**Number of Observations Read**	1293
**Number of Observations Used**	1293

Class Level Information				
**Class**	**Value**	**Design Variables**		
**gender**	**Female**	0	0	
	**Male**	1	0	
	**Other**	0	1	
**race**	**black**	1	0	
	**other race**	0	1	
	**white**	0	0	
**age_cat**	**35-54**	1	0	
	**greater than 55**	0	1	
	**less than 35**	0	0	
**inc**	**$100,000 and above**	0	0	0
	**40,000 - $99,999**	1	0	0
	**less than $40,000**	0	1	0
	**missing**	0	0	1
**edu**	**college degree or higher**	0	0	
	**missing**	1	0	
	**no college degree**	0	1	

The GENMOD Procedure

Class Level Information				
Class	Value	Design Variables		
kids	missing	1	0	
	no	0	0	
	yes	0	1	
marital_status	married or living as married	1	0	0
	missing	0	1	0
	single	0	0	0
	widowed, divorced	0	0	1
feltunwell	missing	1	0	
	no	0	0	
	yes	0	1	
compare_others	good to excellent	0		
	poor or fair	1		
ratehealth	good to excellent	0		
	poor or fair	1		
x1	no	0		
	yes	1		
x2	no	0		
	yes	1		
x3	no	0		
	yes	1		
x4	no	0		
	yes	1		
x5	no	0		
	yes	1		

The GENMOD Procedure

Class Level Information				
Class	Value	Design Variables		
x6	no	0		
	yes	1		
x8	no	0		
	yes	1		
x9	no	0		
	yes	1		
x10	no	0		
	yes	1		
x11	no	0		
	yes	1		
x12	no	0		
	yes	1		
x13	no	0		
	yes	1		
x14	no	0		
	yes	1		
upset	do not want to answer	1	0	0
	missing	0	0	0
	no	0	1	0
	yes	0	0	1
hadajob	no	1		
	yes	0		
infected	no	0		
	yes	1		

The GENMOD Procedure

Criteria For Assessing Goodness Of Fit			
**Criterion**	**DF**	**Value**	**Value/DF**
**Deviance**	1243	1502.6050	1.2089
**Scaled Deviance**	1243	1502.6050	1.2089
**Pearson Chi-Square**	1243	1284.5292	1.0334
**Scaled Pearson X2**	1243	1284.5292	1.0334
**Log Likelihood**		15322.6926	
**Full Log Likelihood**		-3619.9135	
**AIC (smaller is better)**		7341.8270	
**AICC (smaller is better)**		7346.1010	
**BIC (smaller is better)**		7605.2277	

Algorithm converged.

Analysis Of Maximum Likelihood Parameter Estimates									
**Parameter**			**DF**	**Estimate**	**Standard Error**	**Wald 95% Confidence Limits**		**Wald Chi-Square**	**Pr > ChiSq**
**Intercept**			1	2.2655	0.0830	2.1029	2.4281	745.67	<.0001
**gender*x11**	Male	yes	1	0.1405	0.2417	-0.3333	0.6142	0.34	0.5612
**gender*x11**	Other	yes	0	0.0000	0.0000	0.0000	0.0000	.	.
**x1**	yes		1	0.0061	0.0323	-0.0573	0.0694	0.04	0.8511
**x2**	yes		1	0.1425	0.1602	-0.1715	0.4565	0.79	0.3738
**x3**	yes		1	-0.0654	0.0552	-0.1736	0.0428	1.40	0.2361
**x4**	yes		1	0.0675	0.0464	-0.0234	0.1584	2.12	0.1457
**x5**	yes		1	0.0775	0.0402	-0.0012	0.1562	3.72	0.0536
**x6**	yes		1	-0.0484	0.0423	-0.1312	0.0344	1.31	0.2521
**x8**	yes		1	0.1005	0.0392	0.0237	0.1773	6.58	0.0103
**x9**	yes		1	0.1720	0.1369	-0.0964	0.4403	1.58	0.2091

The GENMOD Procedure

Analysis Of Maximum Likelihood Parameter Estimates									
Parameter			DF	Estimate	Standard Error	Wald 95% Confidence Limits		Wald Chi-Square	Pr > ChiSq
x10	yes		1	0.0717	0.0437	-0.0140	0.1575	2.69	0.1009
x11	yes		1	-0.0254	0.1368	-0.2936	0.2427	0.03	0.8525
x12	yes		1	0.0046	0.1465	-0.2825	0.2917	0.00	0.9749
x13	yes		1	0.0957	0.0759	-0.0530	0.2444	1.59	0.2071
x14	yes		1	0.0566	0.1408	-0.2193	0.3326	0.16	0.6875
upset	do not want to answer		1	-0.0237	0.1555	-0.3284	0.2810	0.02	0.8787
upset	no		1	-0.0030	0.0312	-0.0641	0.0581	0.01	0.9228
upset	yes		1	0.1655	0.0479	0.0717	0.2593	11.95	0.0005
dur			1	0.0017	0.0005	0.0008	0.0026	13.85	0.0002
support_family			1	-0.0021	0.0005	-0.0031	-0.0012	18.60	<.0001
support_doctor			1	-0.0003	0.0005	-0.0013	0.0006	0.50	0.4808
support_fedgovt			1	-0.0008	0.0007	-0.0021	0.0005	1.43	0.2313
support_city			1	0.0010	0.0007	-0.0004	0.0024	1.88	0.1704
support_deptpubhealt			1	0.0001	0.0007	-0.0012	0.0015	0.05	0.8305
support_faithcommuni			1	-0.0007	0.0005	-0.0017	0.0003	1.83	0.1763
support_socialservic			1	0.0011	0.0007	-0.0002	0.0025	2.68	0.1016
support_neighbors			1	-0.0005	0.0005	-0.0015	0.0005	1.07	0.3006
support_other			1	-0.0003	0.0006	-0.0015	0.0010	0.18	0.6685
infected	yes		1	0.0358	0.1167	-0.1930	0.2646	0.09	0.7589
feltunwell	missing		1	-0.0999	0.2346	-0.5597	0.3600	0.18	0.6704
feltunwell	yes		1	0.0578	0.0289	0.0011	0.1144	3.99	0.0458
compare_others	poor or fair		1	0.1274	0.0464	0.0364	0.2184	7.54	0.0060
ratehealth	poor or fair		1	0.0332	0.0623	-0.0889	0.1552	0.28	0.5945
gender	Male		1	-0.1652	0.0295	-0.2230	-0.1074	31.39	<.0001
gender	Other		1	0.0287	0.1008	-0.1689	0.2263	0.08	0.7760

The GENMOD Procedure

Analysis Of Maximum Likelihood Parameter Estimates									
Parameter			DF	Estimate	Standard Error	Wald 95% Confidence Limits		Wald Chi-Square	Pr > ChiSq
**race**	black		1	-0.1292	0.0510	-0.2291	-0.0293	6.42	0.0113
**race**	other race		1	-0.0934	0.0485	-0.1885	0.0017	3.71	0.0542
**age_cat**	35-54		1	-0.0312	0.0374	-0.1045	0.0422	0.69	0.4049
**age_cat**	greater than 55		1	-0.1973	0.0406	-0.2769	-0.1178	23.64	<.0001
**inc**	40,000 - $99,999		1	0.0778	0.0306	0.0178	0.1378	6.45	0.0111
**inc**	less than $40,000		1	0.1407	0.0391	0.0640	0.2174	12.94	0.0003
**inc**	missing		1	0.0681	0.0807	-0.0902	0.2263	0.71	0.3992
**edu**	missing		1	-0.1948	0.1868	-0.5610	0.1714	1.09	0.2971
**edu**	no college degree		1	0.0115	0.0363	-0.0597	0.0827	0.10	0.7518
**marital_status**	married or living as married		1	0.0954	0.0316	0.0335	0.1574	9.11	0.0025
**marital_status**	missing		1	0.3956	0.1545	0.0929	0.6984	6.56	0.0104
**marital_status**	widowed, divorced		1	-0.0090	0.0451	-0.0974	0.0793	0.04	0.8412
**kids**	missing		1	-0.3031	0.1873	-0.6702	0.0640	2.62	0.1056
**kids**	yes		1	-0.0164	0.0354	-0.0859	0.0531	0.21	0.6435
**hadajob**	no		1	-0.0533	0.0330	-0.1180	0.0113	2.62	0.1058
**Dispersion**			1	0.0748	0.0078	0.0610	0.0918		

Note: The negative binomial dispersion parameter was estimated by maximum likelihood.

LR Statistics For Joint Tests			
Source	DF	Chi-Square	Pr > ChiSq
**gender*x11**	2	2.36	0.3078
**x1**	1	0.04	0.8511
**x2**	1	0.79	0.3753
**x3**	1	1.40	0.2359
**x4**	1	2.11	0.1464

The GENMOD Procedure

LR Statistics For Joint Tests			
Source	DF	Chi-Square	Pr > ChiSq
x5	1	3.71	0.0541
x6	1	1.31	0.2516
x8	1	6.54	0.0106
x9	1	1.57	0.2102
x10	1	2.68	0.1016
x11	1	0.03	0.8525
x12	1	0.00	0.9749
x13	1	1.59	0.2080
x14	1	0.16	0.6880
upset	3	16.68	0.0008
dur	1	13.78	0.0002
support_family	1	18.44	<.0001
support_doctor	1	0.50	0.4808
support_fedgovt	1	1.43	0.2311
support_city	1	1.88	0.1704
support_deptpubhealt	1	0.05	0.8305
support_faithcommuni	1	1.83	0.1763
support_socialservic	1	2.68	0.1019
support_neighbors	1	1.07	0.3006
support_other	1	0.18	0.6686
infected	1	0.09	0.7590
feltunwell	2	4.23	0.1208
compare_others	1	7.49	0.0062
ratehealth	1	0.28	0.5945
gender	2	31.58	<.0001
race	2	9.39	0.0091

Fully adjusted models of Anxiety score with gender*theme interaction

The GENMOD Procedure

LR Statistics For Joint Tests			
**Source**	**DF**	**Chi-Square**	**Pr > ChiSq**
**age_cat**	2	33.64	<.0001
**inc**	3	13.18	0.0043
**edu**	2	1.23	0.5400
**marital_status**	3	17.27	0.0006
**kids**	2	2.82	0.2440
**hadajob**	1	2.62	0.1058

Note: Under full-rank parameterizations, Type 3 effect tests are replaced by joint tests. The joint test for an effect is a test that all the parameters associated with that effect are zero. Such joint tests might not be equivalent to Type 3 effect tests under GLM parameterization.

The GENMOD Procedure

Model Information		
**Data Set**	WORK.Z	
**Distribution**	Negative Binomial	
**Link Function**	Log	
**Dependent Variable**	AscoreR	HADS Anxiety score

**Number of Observations Read**	1293
**Number of Observations Used**	1293

Class Level Information				
**Class**	**Value**	**Design Variables**		
**gender**	**Female**	0	0	
	**Male**	1	0	
	**Other**	0	1	
**race**	**black**	1	0	
	**other race**	0	1	
	**white**	0	0	
**age_cat**	**35-54**	1	0	
	**greater than 55**	0	1	
	**less than 35**	0	0	
**inc**	**$100,000 and above**	0	0	0
	**40,000 - $99,999**	1	0	0
	**less than $40,000**	0	1	0
	**missing**	0	0	1
**edu**	**college degree or higher**	0	0	
	**missing**	1	0	
	**no college degree**	0	1	

The GENMOD Procedure

Class Level Information				
Class	Value	Design Variables		
kids	missing	1	0	
	no	0	0	
	yes	0	1	
marital_status	married or living as married	1	0	0
	missing	0	1	0
	single	0	0	0
	widowed, divorced	0	0	1
feltunwell	missing	1	0	
	no	0	0	
	yes	0	1	
compare_others	good to excellent	0		
	poor or fair	1		
ratehealth	good to excellent	0		
	poor or fair	1		
x1	no	0		
	yes	1		
x2	no	0		
	yes	1		
x3	no	0		
	yes	1		
x4	no	0		
	yes	1		
x5	no	0		
	yes	1		

The GENMOD Procedure

Class Level Information				
Class	Value	Design Variables		
x6	no	0		
	yes	1		
x8	no	0		
	yes	1		
x9	no	0		
	yes	1		
x10	no	0		
	yes	1		
x11	no	0		
	yes	1		
x12	no	0		
	yes	1		
x13	no	0		
	yes	1		
x14	no	0		
	yes	1		
upset	do not want to answer	1	0	0
	missing	0	0	0
	no	0	1	0
	yes	0	0	1
hadajob	no	1		
	yes	0		
infected	no	0		
	yes	1		

The GENMOD Procedure

Criteria For Assessing Goodness Of Fit			
**Criterion**	**DF**	**Value**	**Value/DF**
**Deviance**	1242	1502.7131	1.2099
**Scaled Deviance**	1242	1502.7131	1.2099
**Pearson Chi-Square**	1242	1285.7217	1.0352
**Scaled Pearson X2**	1242	1285.7217	1.0352
**Log Likelihood**		15323.0144	
**Full Log Likelihood**		-3619.5917	
**AIC (smaller is better)**		7343.1834	
**AICC (smaller is better)**		7347.6286	
**BIC (smaller is better)**		7611.7489	

Algorithm converged.

Analysis Of Maximum Likelihood Parameter Estimates									
**Parameter**			**DF**	**Estimate**	**Standard Error**	**Wald 95% Confidence Limits**		**Wald Chi-Square**	**Pr > ChiSq**
**Intercept**			1	2.2639	0.0832	2.1009	2.4270	740.79	<.0001
**gender*x12**	Male	yes	1	0.3337	0.3493	-0.3510	1.0183	0.91	0.3395
**gender*x12**	Other	yes	1	-0.0392	0.4742	-0.9687	0.8903	0.01	0.9341
**x1**	yes		1	0.0067	0.0323	-0.0566	0.0700	0.04	0.8363
**x2**	yes		1	0.1439	0.1602	-0.1701	0.4578	0.81	0.3691
**x3**	yes		1	-0.0666	0.0551	-0.1747	0.0414	1.46	0.2266
**x4**	yes		1	0.0690	0.0463	-0.0219	0.1598	2.21	0.1367
**x5**	yes		1	0.0794	0.0402	0.0006	0.1582	3.90	0.0482
**x6**	yes		1	-0.0480	0.0423	-0.1308	0.0348	1.29	0.2559
**x8**	yes		1	0.1002	0.0392	0.0234	0.1769	6.54	0.0105
**x9**	yes		1	0.1717	0.1369	-0.0966	0.4400	1.57	0.2096

Fully adjusted models of Anxietry score with gender*theme interaction

The GENMOD Procedure

Analysis Of Maximum Likelihood Parameter Estimates									
Parameter			DF	Estimate	Standard Error	Wald 95% Confidence Limits		Wald Chi-Square	Pr > ChiSq
**x10**	yes		1	0.0727	0.0438	-0.0130	0.1585	2.76	0.0964
**x11**	yes		1	0.0196	0.1129	-0.2017	0.2409	0.03	0.8621
**x12**	yes		1	-0.0680	0.1813	-0.4234	0.2875	0.14	0.7078
**x13**	yes		1	0.0957	0.0758	-0.0529	0.2444	1.59	0.2069
**x14**	yes		1	0.0569	0.1408	-0.2189	0.3328	0.16	0.6859
**upset**	do not want to answer		1	-0.0249	0.1556	-0.3298	0.2800	0.03	0.8729
**upset**	no		1	-0.0031	0.0312	-0.0643	0.0580	0.01	0.9196
**upset**	yes		1	0.1674	0.0478	0.0737	0.2611	12.26	0.0005
**dur**			1	0.0017	0.0005	0.0008	0.0026	13.91	0.0002
**support_family**			1	-0.0021	0.0005	-0.0031	-0.0012	18.65	<.0001
**support_doctor**			1	-0.0004	0.0005	-0.0013	0.0006	0.51	0.4735
**support_fedgovt**			1	-0.0008	0.0007	-0.0021	0.0005	1.42	0.2327
**support_city**			1	0.0010	0.0007	-0.0004	0.0025	1.98	0.1599
**support_deptpubhealt**			1	0.0001	0.0007	-0.0012	0.0015	0.03	0.8608
**support_faithcommuni**			1	-0.0007	0.0005	-0.0017	0.0003	1.79	0.1814
**support_socialservic**			1	0.0011	0.0007	-0.0002	0.0025	2.72	0.0991
**support_neighbors**			1	-0.0005	0.0005	-0.0015	0.0005	1.00	0.3183
**support_other**			1	-0.0002	0.0006	-0.0015	0.0010	0.14	0.7061
**infected**	yes		1	0.0466	0.1157	-0.1802	0.2734	0.16	0.6870
**feltunwell**	missing		1	-0.0986	0.2346	-0.5583	0.3611	0.18	0.6742
**feltunwell**	yes		1	0.0571	0.0289	0.0004	0.1137	3.89	0.0485
**compare_others**	poor or fair		1	0.1278	0.0464	0.0368	0.2187	7.58	0.0059
**ratehealth**	poor or fair		1	0.0316	0.0623	-0.0905	0.1537	0.26	0.6118
**gender**	Male		1	-0.1654	0.0294	-0.2230	-0.1078	31.68	<.0001
**gender**	Other		1	0.0351	0.1030	-0.1667	0.2370	0.12	0.7330

The GENMOD Procedure

Analysis Of Maximum Likelihood Parameter Estimates									
Parameter			DF	Estimate	Standard Error	Wald 95% Confidence Limits		Wald Chi-Square	Pr > ChiSq
race	black		1	-0.1284	0.0510	-0.2283	-0.0284	6.34	0.0118
race	other race		1	-0.0936	0.0485	-0.1887	0.0014	3.73	0.0535
age_cat	35-54		1	-0.0316	0.0375	-0.1050	0.0418	0.71	0.3987
age_cat	greater than 55		1	-0.1977	0.0406	-0.2773	-0.1180	23.67	<.0001
inc	40,000 - $99,999		1	0.0762	0.0306	0.0162	0.1362	6.20	0.0128
inc	less than $40,000		1	0.1395	0.0391	0.0629	0.2160	12.76	0.0004
inc	missing		1	0.0669	0.0807	-0.0913	0.2251	0.69	0.4074
edu	missing		1	-0.1935	0.1868	-0.5596	0.1726	1.07	0.3003
edu	no college degree		1	0.0120	0.0363	-0.0592	0.0833	0.11	0.7405
marital_status	married or living as married		1	0.0937	0.0316	0.0318	0.1557	8.79	0.0030
marital_status	missing		1	0.3927	0.1543	0.0901	0.6952	6.47	0.0110
marital_status	widowed, divorced		1	-0.0101	0.0451	-0.0984	0.0782	0.05	0.8229
kids	missing		1	-0.3004	0.1872	-0.6673	0.0666	2.57	0.1087
kids	yes		1	-0.0153	0.0354	-0.0848	0.0542	0.19	0.6659
hadajob	no		1	-0.0523	0.0330	-0.1171	0.0124	2.51	0.1132
Dispersion			1	0.0747	0.0078	0.0609	0.0917		

Note: The negative binomial dispersion parameter was estimated by maximum likelihood.

LR Statistics For Joint Tests			
Source	DF	Chi-Square	Pr > ChiSq
gender*x12	2	0.98	0.6126
x1	1	0.04	0.8363
x2	1	0.80	0.3705
x3	1	1.46	0.2263
x4	1	2.21	0.1374

The GENMOD Procedure

LR Statistics For Joint Tests			
Source	DF	Chi-Square	Pr > ChiSq
x5	1	3.89	0.0487
x6	1	1.29	0.2555
x8	1	6.50	0.0108
x9	1	1.57	0.2107
x10	1	2.75	0.0970
x11	1	0.03	0.8622
x12	1	0.14	0.7073
x13	1	1.59	0.2077
x14	1	0.16	0.6864
upset	3	17.10	0.0007
dur	1	13.84	0.0002
support_family	1	18.48	<.0001
support_doctor	1	0.51	0.4735
support_fedgovt	1	1.43	0.2326
support_city	1	1.98	0.1599
support_deptpubhealt	1	0.03	0.8608
support_faithcommuni	1	1.79	0.1814
support_socialservic	1	2.72	0.0993
support_neighbors	1	1.00	0.3183
support_other	1	0.14	0.7062
infected	1	0.16	0.6872
feltunwell	2	4.12	0.1271
compare_others	1	7.53	0.0061
ratehealth	1	0.26	0.6119
gender	2	31.90	<.0001
race	2	9.33	0.0094

The GENMOD Procedure

LR Statistics For Joint Tests			
**Source**	**DF**	**Chi-Square**	**Pr > ChiSq**
**age_cat**	2	33.64	<.0001
**inc**	3	12.96	0.0047
**edu**	2	1.23	0.5410
**marital_status**	3	16.91	0.0007
**kids**	2	2.75	0.2526
**hadajob**	1	2.51	0.1133

Note: Under full-rank parameterizations, Type 3 effect tests are replaced by joint tests. The joint test for an effect is a test that all the parameters associated with that effect are zero. Such joint tests might not be equivalent to Type 3 effect tests under GLM parameterization.

The GENMOD Procedure

Model Information		
**Data Set**	WORK.Z	
**Distribution**	Negative Binomial	
**Link Function**	Log	
**Dependent Variable**	AscoreR	HADS Anxiety score

**Number of Observations Read**	1293
**Number of Observations Used**	1293

Class Level Information				
**Class**	**Value**	**Design Variables**		
**gender**	**Female**	0	0	
	**Male**	1	0	
	**Other**	0	1	
**race**	**black**	1	0	
	**other race**	0	1	
	**white**	0	0	
**age_cat**	**35-54**	1	0	
	**greater than 55**	0	1	
	**less than 35**	0	0	
**inc**	**$100,000 and above**	0	0	0
	**40,000 - $99,999**	1	0	0
	**less than $40,000**	0	1	0
	**missing**	0	0	1
**edu**	**college degree or higher**	0	0	
	**missing**	1	0	
	**no college degree**	0	1	

The GENMOD Procedure

Class Level Information				
Class	Value	Design Variables		
kids	missing	1	0	
	no	0	0	
	yes	0	1	
marital_status	married or living as married	1	0	0
	missing	0	1	0
	single	0	0	0
	widowed, divorced	0	0	1
feltunwell	missing	1	0	
	no	0	0	
	yes	0	1	
compare_others	good to excellent	0		
	poor or fair	1		
ratehealth	good to excellent	0		
	poor or fair	1		
x1	no	0		
	yes	1		
x2	no	0		
	yes	1		
x3	no	0		
	yes	1		
x4	no	0		
	yes	1		
x5	no	0		
	yes	1		

The GENMOD Procedure

Class Level Information				
Class	Value	Design Variables		
x6	no	0		
	yes	1		
x8	no	0		
	yes	1		
x9	no	0		
	yes	1		
x10	no	0		
	yes	1		
x11	no	0		
	yes	1		
x12	no	0		
	yes	1		
x13	no	0		
	yes	1		
x14	no	0		
	yes	1		
upset	do not want to answer	1	0	0
	missing	0	0	0
	no	0	1	0
	yes	0	0	1
hadajob	no	1		
	yes	0		
infected	no	0		
	yes	1		

The GENMOD Procedure

Criteria For Assessing Goodness Of Fit			
Criterion	DF	Value	Value/DF
Deviance	1242	1502.5721	1.2098
Scaled Deviance	1242	1502.5721	1.2098
Pearson Chi-Square	1242	1286.0384	1.0355
Scaled Pearson X2	1242	1286.0384	1.0355
Log Likelihood		15323.0297	
Full Log Likelihood		-3619.5765	
AIC (smaller is better)		7343.1529	
AICC (smaller is better)		7347.5981	
BIC (smaller is better)		7611.7184	

Algorithm converged.

Analysis Of Maximum Likelihood Parameter Estimates									
Parameter			DF	Estimate	Standard Error	Wald 95% Confidence Limits		Wald Chi-Square	Pr > ChiSq
Intercept			1	2.2658	0.0830	2.1033	2.4284	746.13	<.0001
gender*x13	Male	yes	1	0.1718	0.1808	-0.1825	0.5261	0.90	0.3419
gender*x13	Other	yes	1	0.1791	0.4187	-0.6415	0.9997	0.18	0.6688
x1	yes		1	0.0065	0.0323	-0.0568	0.0698	0.04	0.8407
x2	yes		1	0.1425	0.1602	-0.1714	0.4565	0.79	0.3736
x3	yes		1	-0.0661	0.0551	-0.1742	0.0419	1.44	0.2301
x4	yes		1	0.0680	0.0464	-0.0229	0.1588	2.15	0.1427
x5	yes		1	0.0780	0.0401	-0.0006	0.1567	3.78	0.0519
x6	yes		1	-0.0481	0.0422	-0.1309	0.0347	1.30	0.2546
x8	yes		1	0.0995	0.0392	0.0226	0.1764	6.43	0.0112
x9	yes		1	0.1774	0.1369	-0.0910	0.4458	1.68	0.1951

The GENMOD Procedure

Analysis Of Maximum Likelihood Parameter Estimates									
Parameter			DF	Estimate	Standard Error	Wald 95% Confidence Limits		Wald Chi-Square	Pr > ChiSq
x10	yes		1	0.0720	0.0437	-0.0137	0.1577	2.71	0.0998
x11	yes		1	0.0190	0.1129	-0.2023	0.2403	0.03	0.8662
x12	yes		1	0.0034	0.1465	-0.2836	0.2905	0.00	0.9813
x13	yes		1	0.0523	0.0873	-0.1187	0.2234	0.36	0.5488
x14	yes		1	0.0561	0.1408	-0.2198	0.3320	0.16	0.6901
upset	do not want to answer		1	-0.0439	0.1671	-0.3713	0.2836	0.07	0.7930
upset	no		1	-0.0031	0.0312	-0.0641	0.0580	0.01	0.9220
upset	yes		1	0.1675	0.0478	0.0738	0.2612	12.27	0.0005
dur			1	0.0017	0.0005	0.0008	0.0026	13.40	0.0003
support_family			1	-0.0021	0.0005	-0.0030	-0.0011	18.15	<.0001
support_doctor			1	-0.0004	0.0005	-0.0013	0.0006	0.53	0.4651
support_fedgovt			1	-0.0008	0.0007	-0.0021	0.0005	1.38	0.2397
support_city			1	0.0010	0.0007	-0.0005	0.0024	1.79	0.1803
support_deptpubhealt			1	0.0002	0.0007	-0.0012	0.0015	0.06	0.7998
support_faithcommuni			1	-0.0007	0.0005	-0.0017	0.0003	1.82	0.1770
support_socialservic			1	0.0011	0.0007	-0.0002	0.0025	2.72	0.0993
support_neighbors			1	-0.0005	0.0005	-0.0015	0.0005	0.97	0.3239
support_other			1	-0.0003	0.0006	-0.0015	0.0009	0.20	0.6535
infected	yes		1	0.0467	0.1157	-0.1801	0.2735	0.16	0.6866
feltunwell	missing		1	-0.1001	0.2346	-0.5599	0.3597	0.18	0.6696
feltunwell	yes		1	0.0586	0.0290	0.0019	0.1154	4.10	0.0429
compare_others	poor or fair		1	0.1258	0.0465	0.0345	0.2170	7.30	0.0069
ratehealth	poor or fair		1	0.0333	0.0627	-0.0895	0.1562	0.28	0.5948
gender	Male		1	-0.1679	0.0297	-0.2261	-0.1097	32.01	<.0001
gender	Other		1	0.0218	0.1028	-0.1797	0.2233	0.05	0.8319

Fully adjusted models of Anxietry score with gender*theme interaction

The GENMOD Procedure

Analysis Of Maximum Likelihood Parameter Estimates									
Parameter			DF	Estimate	Standard Error	Wald 95% Confidence Limits		Wald Chi-Square	Pr > ChiSq
**race**	black		1	-0.1295	0.0510	-0.2294	-0.0296	6.45	0.0111
**race**	other race		1	-0.0934	0.0485	-0.1884	0.0016	3.71	0.0541
**age_cat**	35-54		1	-0.0303	0.0374	-0.1036	0.0431	0.65	0.4187
**age_cat**	greater than 55		1	-0.1970	0.0406	-0.2765	-0.1175	23.56	<.0001
**inc**	40,000 - $99,999		1	0.0773	0.0306	0.0173	0.1372	6.38	0.0115
**inc**	less than $40,000		1	0.1407	0.0391	0.0641	0.2173	12.96	0.0003
**inc**	missing		1	0.0675	0.0807	-0.0907	0.2257	0.70	0.4033
**edu**	missing		1	-0.1940	0.1869	-0.5602	0.1723	1.08	0.2993
**edu**	no college degree		1	0.0129	0.0363	-0.0583	0.0842	0.13	0.7217
**marital_status**	married or living as married		1	0.0946	0.0316	0.0326	0.1565	8.95	0.0028
**marital_status**	missing		1	0.3931	0.1544	0.0906	0.6957	6.49	0.0109
**marital_status**	widowed, divorced		1	-0.0100	0.0451	-0.0983	0.0783	0.05	0.8245
**kids**	missing		1	-0.3011	0.1873	-0.6681	0.0659	2.59	0.1079
**kids**	yes		1	-0.0175	0.0355	-0.0870	0.0520	0.24	0.6210
**hadajob**	no		1	-0.0530	0.0330	-0.1176	0.0116	2.58	0.1081
**Dispersion**			1	0.0747	0.0078	0.0609	0.0917		

Note: The negative binomial dispersion parameter was estimated by maximum likelihood.

LR Statistics For Joint Tests			
Source	DF	Chi-Square	Pr > ChiSq
**gender*x13**	2	1.01	0.6033
**x1**	1	0.04	0.8408
**x2**	1	0.79	0.3750
**x3**	1	1.44	0.2299
**x4**	1	2.14	0.1434

The GENMOD Procedure

LR Statistics For Joint Tests			
**Source**	**DF**	**Chi-Square**	**Pr > ChiSq**
**x5**	1	3.76	0.0524
**x6**	1	1.30	0.2541
**x8**	1	6.39	0.0115
**x9**	1	1.67	0.1963
**x10**	1	2.70	0.1004
**x11**	1	0.03	0.8663
**x12**	1	0.00	0.9813
**x13**	1	0.36	0.5492
**x14**	1	0.16	0.6906
**upset**	3	17.17	0.0007
**dur**	1	13.34	0.0003
**support_family**	1	17.99	<.0001
**support_doctor**	1	0.53	0.4651
**support_fedgovt**	1	1.38	0.2396
**support_city**	1	1.79	0.1803
**support_deptpubhealt**	1	0.06	0.7999
**support_faithcommuni**	1	1.82	0.1770
**support_socialservic**	1	2.71	0.0996
**support_neighbors**	1	0.97	0.3239
**support_other**	1	0.20	0.6535
**infected**	1	0.16	0.6868
**feltunwell**	2	4.34	0.1142
**compare_others**	1	7.26	0.0071
**ratehealth**	1	0.28	0.5949
**gender**	2	32.12	<.0001
**race**	2	9.42	0.0090

The GENMOD Procedure

LR Statistics For Joint Tests			
**Source**	**DF**	**Chi-Square**	**Pr > ChiSq**
**age_cat**	2	33.73	<.0001
**inc**	3	13.19	0.0042
**edu**	2	1.25	0.5347
**marital_status**	3	17.12	0.0007
**kids**	2	2.81	0.2451
**hadajob**	1	2.58	0.1081

Note: Under full-rank parameterizations, Type 3 effect tests are replaced by joint tests. The joint test for an effect is a test that all the parameters associated with that effect are zero. Such joint tests might not be equivalent to Type 3 effect tests under GLM parameterization.

Fully adjusted models of Anxietry score with gender*theme interaction

The GENMOD Procedure

Model Information		
**Data Set**	WORK.Z	
**Distribution**	Negative Binomial	
**Link Function**	Log	
**Dependent Variable**	AscoreR	HADS Anxiety score

**Number of Observations Read**	1293
**Number of Observations Used**	1293

Class Level Information				
**Class**	**Value**	**Design Variables**		
**gender**	**Female**	0	0	
	**Male**	1	0	
	**Other**	0	1	
**race**	**black**	1	0	
	**other race**	0	1	
	**white**	0	0	
**age_cat**	**35-54**	1	0	
	**greater than 55**	0	1	
	**less than 35**	0	0	
**inc**	**$100,000 and above**	0	0	0
	**40,000 - $99,999**	1	0	0
	**less than $40,000**	0	1	0
	**missing**	0	0	1
**edu**	**college degree or higher**	0	0	
	**missing**	1	0	
	**no college degree**	0	1	

The GENMOD Procedure

Class Level Information				
Class	Value	Design Variables		
kids	missing	1	0	
	no	0	0	
	yes	0	1	
marital_status	married or living as married	1	0	0
	missing	0	1	0
	single	0	0	0
	widowed, divorced	0	0	1
feltunwell	missing	1	0	
	no	0	0	
	yes	0	1	
compare_others	good to excellent	0		
	poor or fair	1		
ratehealth	good to excellent	0		
	poor or fair	1		
x1	no	0		
	yes	1		
x2	no	0		
	yes	1		
x3	no	0		
	yes	1		
x4	no	0		
	yes	1		
x5	no	0		
	yes	1		

The GENMOD Procedure

Class Level Information				
Class	Value	Design Variables		
x6	no	0		
	yes	1		
x8	no	0		
	yes	1		
x9	no	0		
	yes	1		
x10	no	0		
	yes	1		
x11	no	0		
	yes	1		
x12	no	0		
	yes	1		
x13	no	0		
	yes	1		
x14	no	0		
	yes	1		
upset	do not want to answer	1	0	0
	missing	0	0	0
	no	0	1	0
	yes	0	0	1
hadajob	no	1		
	yes	0		
infected	no	0		
	yes	1		

Fully adjusted models of Anxietry score with gender*theme interaction

The GENMOD Procedure

Criteria For Assessing Goodness Of Fit			
**Criterion**	**DF**	**Value**	**Value/DF**
**Deviance**	1243	1502.5515	1.2088
**Scaled Deviance**	1243	1502.5515	1.2088
**Pearson Chi-Square**	1243	1283.9965	1.0330
**Scaled Pearson X2**	1243	1283.9965	1.0330
**Log Likelihood**		15322.6681	
**Full Log Likelihood**		-3619.9380	
**AIC (smaller is better)**		7341.8760	
**AICC (smaller is better)**		7346.1500	
**BIC (smaller is better)**		7605.2768	

Algorithm converged.

Analysis Of Maximum Likelihood Parameter Estimates									
**Parameter**			**DF**	**Estimate**	**Standard Error**	**Wald 95% Confidence Limits**		**Wald Chi-Square**	**Pr > ChiSq**
**Intercept**			1	2.2651	0.0830	2.1025	2.4277	745.43	<.0001
**gender*x14**	Male	yes	1	-0.2727	0.5154	-1.2828	0.7374	0.28	0.5967
**gender*x14**	Other	yes	0	0.0000	0.0000	0.0000	0.0000	.	.
**x1**	yes		1	0.0057	0.0323	-0.0577	0.0690	0.03	0.8611
**x2**	yes		1	0.1426	0.1602	-0.1714	0.4566	0.79	0.3734
**x3**	yes		1	-0.0669	0.0551	-0.1749	0.0411	1.47	0.2249
**x4**	yes		1	0.0676	0.0464	-0.0233	0.1585	2.12	0.1452
**x5**	yes		1	0.0775	0.0402	-0.0012	0.1562	3.72	0.0537
**x6**	yes		1	-0.0490	0.0423	-0.1318	0.0338	1.34	0.2463
**x8**	yes		1	0.0994	0.0392	0.0226	0.1762	6.44	0.0112
**x9**	yes		1	0.1726	0.1369	-0.0957	0.4410	1.59	0.2074

Fully adjusted models of Anxietry score with gender*theme interaction

The GENMOD Procedure

Analysis Of Maximum Likelihood Parameter Estimates									
Parameter			DF	Estimate	Standard Error	Wald 95% Confidence Limits		Wald Chi-Square	Pr > ChiSq
x10	yes		1	0.0711	0.0437	-0.0146	0.1568	2.64	0.1041
x11	yes		1	0.0189	0.1129	-0.2024	0.2403	0.03	0.8668
x12	yes		1	0.0046	0.1465	-0.2825	0.2918	0.00	0.9747
x13	yes		1	0.0952	0.0759	-0.0535	0.2440	1.58	0.2093
x14	yes		1	0.0799	0.1470	-0.2082	0.3681	0.30	0.5866
upset	do not want to answer		1	-0.0227	0.1555	-0.3274	0.2820	0.02	0.8838
upset	no		1	-0.0027	0.0312	-0.0638	0.0584	0.01	0.9299
upset	yes		1	0.1667	0.0478	0.0730	0.2604	12.16	0.0005
dur			1	0.0017	0.0005	0.0008	0.0026	13.80	0.0002
support_family			1	-0.0021	0.0005	-0.0031	-0.0011	18.42	<.0001
support_doctor			1	-0.0004	0.0005	-0.0013	0.0006	0.52	0.4690
support_fedgovt			1	-0.0008	0.0007	-0.0021	0.0005	1.41	0.2352
support_city			1	0.0010	0.0007	-0.0004	0.0024	1.84	0.1745
support_deptpubhealt			1	0.0002	0.0007	-0.0012	0.0015	0.06	0.8132
support_faithcommuni			1	-0.0007	0.0005	-0.0017	0.0003	1.87	0.1714
support_socialservic			1	0.0011	0.0007	-0.0002	0.0025	2.72	0.0991
support_neighbors			1	-0.0005	0.0005	-0.0015	0.0005	1.08	0.2995
support_other			1	-0.0003	0.0006	-0.0015	0.0010	0.17	0.6831
infected	yes		1	0.0440	0.1157	-0.1828	0.2708	0.14	0.7037
feltunwell	missing		1	-0.0995	0.2346	-0.5594	0.3603	0.18	0.6714
feltunwell	yes		1	0.0575	0.0289	0.0008	0.1142	3.96	0.0467
compare_others	poor or fair		1	0.1273	0.0464	0.0364	0.2183	7.53	0.0061
ratehealth	poor or fair		1	0.0331	0.0623	-0.0890	0.1552	0.28	0.5952
gender	Male		1	-0.1626	0.0294	-0.2202	-0.1051	30.70	<.0001
gender	Other		1	0.0301	0.1008	-0.1675	0.2277	0.09	0.7654

Fully adjusted models of Anxietry score with gender*theme interaction

The GENMOD Procedure

Analysis Of Maximum Likelihood Parameter Estimates									
Parameter			DF	Estimate	Standard Error	Wald 95% Confidence Limits		Wald Chi-Square	Pr > ChiSq
race	black		1	-0.1290	0.0510	-0.2289	-0.0291	6.40	0.0114
race	other race		1	-0.0937	0.0485	-0.1887	0.0014	3.73	0.0535
age_cat	35-54		1	-0.0312	0.0374	-0.1045	0.0422	0.69	0.4052
age_cat	greater than 55		1	-0.1970	0.0406	-0.2766	-0.1175	23.56	<.0001
inc	40,000 - $99,999		1	0.0765	0.0306	0.0165	0.1365	6.25	0.0124
inc	less than $40,000		1	0.1391	0.0391	0.0625	0.2157	12.67	0.0004
inc	missing		1	0.0668	0.0808	-0.0914	0.2251	0.68	0.4079
edu	missing		1	-0.1940	0.1868	-0.5602	0.1722	1.08	0.2991
edu	no college degree		1	0.0119	0.0363	-0.0593	0.0831	0.11	0.7425
marital_status	married or living as married		1	0.0950	0.0316	0.0330	0.1569	9.02	0.0027
marital_status	missing		1	0.3937	0.1544	0.0910	0.6963	6.50	0.0108
marital_status	widowed, divorced		1	-0.0099	0.0451	-0.0983	0.0784	0.05	0.8255
kids	missing		1	-0.3076	0.1876	-0.6752	0.0600	2.69	0.1010
kids	yes		1	-0.0161	0.0354	-0.0856	0.0533	0.21	0.6491
hadajob	no		1	-0.0543	0.0330	-0.1190	0.0104	2.71	0.1000
Dispersion			1	0.0748	0.0078	0.0610	0.0918		

Note: The negative binomial dispersion parameter was estimated by maximum likelihood.

LR Statistics For Joint Tests			
Source	DF	Chi-Square	Pr > ChiSq
gender*x14	2	2.31	0.3155
x1	1	0.03	0.8611
x2	1	0.79	0.3749
x3	1	1.47	0.2247
x4	1	2.11	0.1459

The GENMOD Procedure

LR Statistics For Joint Tests			
Source	DF	Chi-Square	Pr > ChiSq
x5	1	3.71	0.0542
x6	1	1.35	0.2459
x8	1	6.40	0.0114
x9	1	1.58	0.2085
x10	1	2.63	0.1048
x11	1	0.03	0.8668
x12	1	0.00	0.9747
x13	1	1.57	0.2102
x14	1	0.29	0.5874
upset	3	16.90	0.0007
dur	1	13.73	0.0002
support_family	1	18.25	<.0001
support_doctor	1	0.52	0.4690
support_fedgovt	1	1.41	0.2351
support_city	1	1.84	0.1745
support_deptpubhealt	1	0.06	0.8132
support_faithcommuni	1	1.87	0.1714
support_socialservic	1	2.72	0.0994
support_neighbors	1	1.08	0.2995
support_other	1	0.17	0.6832
infected	1	0.14	0.7039
feltunwell	2	4.19	0.1228
compare_others	1	7.48	0.0062
ratehealth	1	0.28	0.5953
gender	2	30.90	<.0001
race	2	9.39	0.0091

The GENMOD Procedure

LR Statistics For Joint Tests			
**Source**	**DF**	**Chi-Square**	**Pr > ChiSq**
**age_cat**	2	33.51	<.0001
**inc**	3	12.90	0.0049
**edu**	2	1.23	0.5402
**marital_status**	3	17.20	0.0006
**kids**	2	2.89	0.2361
**hadajob**	1	2.71	0.1000

Note: Under full-rank parameterizations, Type 3 effect tests are replaced by joint tests. The joint test for an effect is a test that all the parameters associated with that effect are zero. Such joint tests might not be equivalent to Type 3 effect tests under GLM parameterization.

Fully adjusted models of Depression score with gender*theme interaction

The GENMOD Procedure

Model Information		
**Data Set**	WORK.Z	
**Distribution**	Negative Binomial	
**Link Function**	Log	
**Dependent Variable**	DscoreR	HADS Depression score

**Number of Observations Read**	1293
**Number of Observations Used**	1293

Class Level Information				
**Class**	**Value**	**Design Variables**		
**gender**	**Female**	0	0	
	**Male**	1	0	
	**Other**	0	1	
**race**	**black**	1	0	
	**other race**	0	1	
	**white**	0	0	
**age_cat**	**35-54**	1	0	
	**greater than 55**	0	1	
	**less than 35**	0	0	
**inc**	**$100,000 and above**	0	0	0
	**40,000 - $99,999**	1	0	0
	**less than $40,000**	0	1	0
	**missing**	0	0	1
**edu**	**college degree or higher**	0	0	
	**missing**	1	0	
	**no college degree**	0	1	

The GENMOD Procedure

Class Level Information				
Class	Value	Design Variables		
kids	missing	1	0	
	no	0	0	
	yes	0	1	
marital_status	married or living as married	1	0	0
	missing	0	1	0
	single	0	0	0
	widowed, divorced	0	0	1
feltunwell	missing	1	0	
	no	0	0	
	yes	0	1	
compare_others	good to excellent	0		
	poor or fair	1		
ratehealth	good to excellent	0		
	poor or fair	1		
x1	no	0		
	yes	1		
x2	no	0		
	yes	1		
x3	no	0		
	yes	1		
x4	no	0		
	yes	1		
x5	no	0		
	yes	1		

Fully adjusted models of Depression score with gender*theme interaction

The GENMOD Procedure

Class Level Information				
Class	Value	Design Variables		
x6	no	0		
	yes	1		
x8	no	0		
	yes	1		
x9	no	0		
	yes	1		
x10	no	0		
	yes	1		
x11	no	0		
	yes	1		
x12	no	0		
	yes	1		
x13	no	0		
	yes	1		
x14	no	0		
	yes	1		
upset	do not want to answer	1	0	0
	missing	0	0	0
	no	0	1	0
	yes	0	0	1
hadajob	no	1		
	yes	0		
infected	no	0		
	yes	1		

Fully adjusted models of Depression score with gender*theme interaction

The GENMOD Procedure

Criteria For Assessing Goodness Of Fit			
Criterion	DF	Value	Value/DF
Deviance	1242	1461.1701	1.1765
Scaled Deviance	1242	1461.1701	1.1765
Pearson Chi-Square	1242	1290.1800	1.0388
Scaled Pearson X2	1242	1290.1800	1.0388
Log Likelihood		7067.4664	
Full Log Likelihood		-3379.1161	
AIC (smaller is better)		6862.2322	
AICC (smaller is better)		6866.6773	
BIC (smaller is better)		7130.7976	

Algorithm converged.

Analysis Of Maximum Likelihood Parameter Estimates									
Parameter			DF	Estimate	Standard Error	Wald 95% Confidence Limits		Wald Chi-Square	Pr > ChiSq
Intercept			1	1.9073	0.1126	1.6867	2.1279	287.18	<.0001
gender*x1	Male	yes	1	-0.0607	0.1050	-0.2665	0.1450	0.33	0.5628
gender*x1	Other	yes	1	-1.0407	0.4279	-1.8793	-0.2020	5.91	0.0150
x1	yes		1	0.0268	0.0492	-0.0697	0.1233	0.30	0.5865
x2	yes		1	0.3606	0.2114	-0.0537	0.7749	2.91	0.0880
x3	yes		1	0.0802	0.0742	-0.0654	0.2257	1.17	0.2803
x4	yes		1	0.2096	0.0613	0.0895	0.3298	11.70	0.0006
x5	yes		1	0.0696	0.0545	-0.0372	0.1765	1.63	0.2015
x6	yes		1	0.0756	0.0561	-0.0343	0.1854	1.82	0.1777
x8	yes		1	0.1081	0.0533	0.0036	0.2126	4.11	0.0427
x9	yes		1	0.0790	0.1910	-0.2953	0.4534	0.17	0.6790

Fully adjusted models of Depression score with gender*theme interaction

The GENMOD Procedure

Analysis Of Maximum Likelihood Parameter Estimates									
Parameter			DF	Estimate	Standard Error	Wald 95% Confidence Limits		Wald Chi-Square	Pr > ChiSq
x10	yes		1	0.1369	0.0591	0.0210	0.2527	5.36	0.0206
x11	yes		1	-0.0191	0.1536	-0.3201	0.2820	0.02	0.9013
x12	yes		1	0.0022	0.1989	-0.3877	0.3921	0.00	0.9911
x13	yes		1	0.1355	0.1032	-0.0667	0.3377	1.73	0.1889
x14	yes		1	0.1639	0.1820	-0.1928	0.5205	0.81	0.3678
upset	do not want to answer		1	-0.0239	0.2108	-0.4371	0.3893	0.01	0.9097
upset	no		1	-0.0559	0.0417	-0.1376	0.0258	1.80	0.1797
upset	yes		1	0.1340	0.0643	0.0079	0.2600	4.34	0.0373
dur			1	0.0026	0.0006	0.0014	0.0038	17.60	<.0001
support_family			1	-0.0032	0.0007	-0.0045	-0.0019	23.91	<.0001
support_doctor			1	-0.0011	0.0007	-0.0025	0.0002	2.91	0.0878
support_fedgovt			1	0.0002	0.0009	-0.0016	0.0019	0.04	0.8393
support_city			1	0.0023	0.0010	0.0004	0.0043	5.35	0.0208
support_deptpubhealt			1	-0.0017	0.0009	-0.0035	0.0002	3.20	0.0736
support_faithcommuni			1	-0.0019	0.0007	-0.0032	-0.0005	7.60	0.0058
support_socialservic			1	0.0017	0.0009	-0.0001	0.0035	3.37	0.0662
support_neighbors			1	-0.0014	0.0007	-0.0027	0.0000	3.75	0.0527
support_other			1	-0.0013	0.0008	-0.0030	0.0003	2.44	0.1182
infected	yes		1	0.0542	0.1600	-0.2594	0.3678	0.11	0.7348
feltunwell	missing		1	0.1551	0.2935	-0.4202	0.7304	0.28	0.5972
feltunwell	yes		1	0.0906	0.0389	0.0143	0.1669	5.42	0.0199
compare_others	poor or fair		1	0.2599	0.0615	0.1394	0.3805	17.86	<.0001
ratehealth	poor or fair		1	-0.0766	0.0822	-0.2378	0.0846	0.87	0.3518
gender	Male		1	-0.1285	0.0428	-0.2123	-0.0447	9.02	0.0027
gender	Other		1	0.1935	0.1462	-0.0931	0.4801	1.75	0.1858

Fully adjusted models of Depression score with gender*theme interaction

The GENMOD Procedure

Analysis Of Maximum Likelihood Parameter Estimates									
Parameter			DF	Estimate	Standard Error	Wald 95% Confidence Limits		Wald Chi-Square	Pr > ChiSq
race	black		1	-0.0792	0.0683	-0.2130	0.0546	1.35	0.2461
race	other race		1	0.0246	0.0641	-0.1010	0.1503	0.15	0.7007
age_cat	35-54		1	0.0214	0.0511	-0.0787	0.1215	0.18	0.6752
age_cat	greater than 55		1	-0.0752	0.0552	-0.1834	0.0329	1.86	0.1728
inc	40,000 - $99,999		1	0.0585	0.0414	-0.0226	0.1396	2.00	0.1574
inc	less than $40,000		1	0.1570	0.0524	0.0544	0.2597	8.99	0.0027
inc	missing		1	0.1102	0.1070	-0.0995	0.3199	1.06	0.3029
edu	missing		1	-0.4485	0.2708	-0.9793	0.0823	2.74	0.0977
edu	no college degree		1	0.0130	0.0484	-0.0819	0.1080	0.07	0.7881
marital_status	married or living as married		1	0.0831	0.0427	-0.0005	0.1667	3.80	0.0514
marital_status	missing		1	0.4325	0.2118	0.0174	0.8476	4.17	0.0412
marital_status	widowed, divorced		1	0.0139	0.0603	-0.1043	0.1320	0.05	0.8179
kids	missing		1	-0.3876	0.2581	-0.8935	0.1183	2.26	0.1331
kids	yes		1	-0.0028	0.0476	-0.0960	0.0904	0.00	0.9532
hadajob	no		1	0.0338	0.0438	-0.0521	0.1197	0.60	0.4404
Dispersion			1	0.1667	0.0140	0.1414	0.1966		

Note: The negative binomial dispersion parameter was estimated by maximum likelihood.

LR Statistics For Joint Tests			
Source	DF	Chi-Square	Pr > ChiSq
gender*x1	2	6.36	0.0415
x1	1	0.30	0.5865
x2	1	2.94	0.0866
x3	1	1.17	0.2803
x4	1	11.67	0.0006

Fully adjusted models of Depression score with gender*theme interaction

The GENMOD Procedure

LR Statistics For Joint Tests			
**Source**	**DF**	**Chi-Square**	**Pr > ChiSq**
**x5**	1	1.63	0.2016
**x6**	1	1.82	0.1778
**x8**	1	4.10	0.0428
**x9**	1	0.17	0.6787
**x10**	1	5.36	0.0206
**x11**	1	0.02	0.9013
**x12**	1	0.00	0.9911
**x13**	1	1.73	0.1885
**x14**	1	0.81	0.3674
**upset**	3	12.18	0.0068
**dur**	1	17.49	<.0001
**support_family**	1	23.77	<.0001
**support_doctor**	1	2.91	0.0879
**support_fedgovt**	1	0.04	0.8393
**support_city**	1	5.34	0.0208
**support_deptpubhealt**	1	3.20	0.0737
**support_faithcommuni**	1	7.58	0.0059
**support_socialservic**	1	3.37	0.0662
**support_neighbors**	1	3.75	0.0528
**support_other**	1	2.44	0.1183
**infected**	1	0.11	0.7347
**feltunwell**	2	5.61	0.0607
**compare_others**	1	17.85	<.0001
**ratehealth**	1	0.87	0.3519
**gender**	2	11.46	0.0032
**race**	2	1.58	0.4541

Fully adjusted models of Depression score with gender*theme interaction

The GENMOD Procedure

LR Statistics For Joint Tests			
**Source**	**DF**	**Chi-Square**	**Pr > ChiSq**
**age_cat**	2	5.15	0.0761
**inc**	3	9.34	0.0251
**edu**	2	2.94	0.2299
**marital_status**	3	7.67	0.0532
**kids**	2	2.29	0.3177
**hadajob**	1	0.60	0.4405

Note: Under full-rank parameterizations, Type 3 effect tests are replaced by joint tests. The joint test for an effect is a test that all the parameters associated with that effect are zero. Such joint tests might not be equivalent to Type 3 effect tests under GLM parameterization.

The GENMOD Procedure

Model Information		
**Data Set**	WORK.Z	
**Distribution**	Negative Binomial	
**Link Function**	Log	
**Dependent Variable**	DscoreR	HADS Depression score

**Number of Observations Read**	1293
**Number of Observations Used**	1293

Class Level Information				
**Class**	**Value**	**Design Variables**		
**gender**	**Female**	0	0	
	**Male**	1	0	
	**Other**	0	1	
**race**	**black**	1	0	
	**other race**	0	1	
	**white**	0	0	
**age_cat**	**35-54**	1	0	
	**greater than 55**	0	1	
	**less than 35**	0	0	
**inc**	**$100,000 and above**	0	0	0
	**40,000 - $99,999**	1	0	0
	**less than $40,000**	0	1	0
	**missing**	0	0	1
**edu**	**college degree or higher**	0	0	
	**missing**	1	0	
	**no college degree**	0	1	

Fully adjusted models of Depression score with gender*theme interaction

The GENMOD Procedure

Class Level Information				
Class	Value	Design Variables		
kids	missing	1	0	
	no	0	0	
	yes	0	1	
marital_status	married or living as married	1	0	0
	missing	0	1	0
	single	0	0	0
	widowed, divorced	0	0	1
feltunwell	missing	1	0	
	no	0	0	
	yes	0	1	
compare_others	good to excellent	0		
	poor or fair	1		
ratehealth	good to excellent	0		
	poor or fair	1		
x1	no	0		
	yes	1		
x2	no	0		
	yes	1		
x3	no	0		
	yes	1		
x4	no	0		
	yes	1		
x5	no	0		
	yes	1		

The GENMOD Procedure

Class Level Information				
**Class**	**Value**	**Design Variables**		
**x6**	**no**	0		
	**yes**	1		
**x8**	**no**	0		
	**yes**	1		
**x9**	**no**	0		
	**yes**	1		
**x10**	**no**	0		
	**yes**	1		
**x11**	**no**	0		
	**yes**	1		
**x12**	**no**	0		
	**yes**	1		
**x13**	**no**	0		
	**yes**	1		
**x14**	**no**	0		
	**yes**	1		
**upset**	**do not want to answer**	1	0	0
	**missing**	0	0	0
	**no**	0	1	0
	**yes**	0	0	1
**hadajob**	**no**	1		
	**yes**	0		
**infected**	**no**	0		
	**yes**	1		

Fully adjusted models of Depression score with gender*theme interaction

The GENMOD Procedure

Criteria For Assessing Goodness Of Fit			
Criterion	DF	Value	Value/DF
Deviance	1243	1462.3003	1.1764
Scaled Deviance	1243	1462.3003	1.1764
Pearson Chi-Square	1243	1286.8839	1.0353
Scaled Pearson X2	1243	1286.8839	1.0353
Log Likelihood		7067.5999	
Full Log Likelihood		-3378.9826	
AIC (smaller is better)		6859.9652	
AICC (smaller is better)		6864.2391	
BIC (smaller is better)		7123.3659	

Algorithm converged.

Analysis Of Maximum Likelihood Parameter Estimates									
Parameter			DF	Estimate	Standard Error	Wald 95% Confidence Limits		Wald Chi-Square	Pr > ChiSq
Intercept			1	1.9472	0.1117	1.7283	2.1660	304.14	<.0001
gender*x2	Male	yes	1	1.1254	0.4446	0.2540	1.9968	6.41	0.0114
gender*x2	Other	yes	0	0.0000	0.0000	0.0000	0.0000	.	.
x1	yes		1	0.0023	0.0440	-0.0840	0.0886	0.00	0.9585
x2	yes		1	-0.0617	0.2658	-0.5826	0.4592	0.05	0.8165
x3	yes		1	0.0534	0.0743	-0.0922	0.1989	0.52	0.4725
x4	yes		1	0.2086	0.0612	0.0886	0.3286	11.60	0.0007
x5	yes		1	0.0700	0.0545	-0.0368	0.1768	1.65	0.1988
x6	yes		1	0.0737	0.0560	-0.0361	0.1834	1.73	0.1883
x8	yes		1	0.1028	0.0533	-0.0016	0.2072	3.73	0.0536
x9	yes		1	0.0733	0.1907	-0.3004	0.4470	0.15	0.7005

The GENMOD Procedure

Analysis Of Maximum Likelihood Parameter Estimates									
Parameter			DF	Estimate	Standard Error	Wald 95% Confidence Limits		Wald Chi-Square	Pr > ChiSq
x10	yes		1	0.1349	0.0590	0.0193	0.2506	5.23	0.0222
x11	yes		1	-0.0176	0.1535	-0.3185	0.2833	0.01	0.9089
x12	yes		1	0.0189	0.1985	-0.3703	0.4080	0.01	0.9243
x13	yes		1	0.1404	0.1031	-0.0616	0.3424	1.86	0.1730
x14	yes		1	0.1659	0.1817	-0.1903	0.5220	0.83	0.3614
upset	do not want to answer		1	0.0078	0.2096	-0.4030	0.4186	0.00	0.9703
upset	no		1	-0.0608	0.0416	-0.1424	0.0209	2.13	0.1445
upset	yes		1	0.1314	0.0643	0.0054	0.2574	4.18	0.0410
dur			1	0.0026	0.0006	0.0014	0.0038	17.91	<.0001
support_family			1	-0.0033	0.0007	-0.0046	-0.0020	25.33	<.0001
support_doctor			1	-0.0011	0.0007	-0.0025	0.0002	2.92	0.0877
support_fedgovt			1	0.0001	0.0009	-0.0017	0.0018	0.01	0.9387
support_city			1	0.0024	0.0010	0.0004	0.0043	5.67	0.0173
support_deptpubhealt			1	-0.0017	0.0009	-0.0035	0.0001	3.47	0.0625
support_faithcommuni			1	-0.0019	0.0007	-0.0033	-0.0006	7.98	0.0047
support_socialservic			1	0.0017	0.0009	-0.0001	0.0035	3.29	0.0695
support_neighbors			1	-0.0013	0.0007	-0.0027	0.0001	3.45	0.0632
support_other			1	-0.0014	0.0008	-0.0031	0.0002	2.89	0.0890
infected	yes		1	0.0472	0.1598	-0.2661	0.3605	0.09	0.7678
feltunwell	missing		1	0.1556	0.2934	-0.4195	0.7308	0.28	0.5958
feltunwell	yes		1	0.0967	0.0388	0.0206	0.1729	6.20	0.0128
compare_others	poor or fair		1	0.2421	0.0610	0.1225	0.3617	15.74	<.0001
ratehealth	poor or fair		1	-0.0599	0.0819	-0.2204	0.1006	0.54	0.4644
gender	Male		1	-0.1481	0.0393	-0.2252	-0.0711	14.19	0.0002
gender	Other		1	0.0589	0.1357	-0.2071	0.3250	0.19	0.6641

The GENMOD Procedure

Analysis Of Maximum Likelihood Parameter Estimates									
Parameter			DF	Estimate	Standard Error	Wald 95% Confidence Limits		Wald Chi-Square	Pr > ChiSq
race	black		1	-0.0810	0.0682	-0.2147	0.0528	1.41	0.2355
race	other race		1	0.0263	0.0639	-0.0988	0.1515	0.17	0.6801
age_cat	35-54		1	0.0113	0.0511	-0.0889	0.1115	0.05	0.8250
age_cat	greater than 55		1	-0.0886	0.0551	-0.1967	0.0194	2.58	0.1080
inc	40,000 - $99,999		1	0.0555	0.0413	-0.0254	0.1365	1.81	0.1789
inc	less than $40,000		1	0.1527	0.0524	0.0500	0.2554	8.49	0.0036
inc	missing		1	0.1083	0.1069	-0.1012	0.3178	1.03	0.3110
edu	missing		1	-0.4637	0.2700	-0.9929	0.0655	2.95	0.0859
edu	no college degree		1	0.0142	0.0484	-0.0807	0.1091	0.09	0.7686
marital_status	married or living as married		1	0.0841	0.0426	0.0005	0.1676	3.89	0.0485
marital_status	missing		1	0.4305	0.2116	0.0157	0.8453	4.14	0.0419
marital_status	widowed, divorced		1	0.0082	0.0602	-0.1099	0.1262	0.02	0.8920
kids	missing		1	-0.3925	0.2580	-0.8981	0.1131	2.31	0.1282
kids	yes		1	-0.0029	0.0475	-0.0961	0.0903	0.00	0.9514
hadajob	no		1	0.0335	0.0438	-0.0524	0.1193	0.58	0.4448
Dispersion			1	0.1664	0.0140	0.1410	0.1962		

Note: The negative binomial dispersion parameter was estimated by maximum likelihood.

LR Statistics For Joint Tests			
Source	DF	Chi-Square	Pr > ChiSq
gender*x2	2	8.45	0.0147
x1	1	0.00	0.9585
x2	1	0.05	0.8164
x3	1	0.52	0.4725
x4	1	11.58	0.0007

The GENMOD Procedure

LR Statistics For Joint Tests			
Source	DF	Chi-Square	Pr > ChiSq
x5	1	1.65	0.1988
x6	1	1.73	0.1884
x8	1	3.72	0.0537
x9	1	0.15	0.7003
x10	1	5.22	0.0223
x11	1	0.01	0.9089
x12	1	0.01	0.9243
x13	1	1.86	0.1726
x14	1	0.83	0.3610
upset	3	12.71	0.0053
dur	1	17.78	<.0001
support_family	1	25.17	<.0001
support_doctor	1	2.91	0.0878
support_fedgovt	1	0.01	0.9387
support_city	1	5.66	0.0174
support_deptpubhealt	1	3.47	0.0626
support_faithcommuni	1	7.96	0.0048
support_socialservic	1	3.29	0.0696
support_neighbors	1	3.45	0.0633
support_other	1	2.89	0.0891
infected	1	0.09	0.7677
feltunwell	2	6.38	0.0411
compare_others	1	15.73	<.0001
ratehealth	1	0.53	0.4645
gender	2	14.60	0.0007
race	2	1.67	0.4334

The GENMOD Procedure

LR Statistics For Joint Tests			
**Source**	**DF**	**Chi-Square**	**Pr > ChiSq**
**age_cat**	2	5.73	0.0570
**inc**	3	8.88	0.0310
**edu**	2	3.17	0.2044
**marital_status**	3	7.95	0.0470
**kids**	2	2.35	0.3082
**hadajob**	1	0.58	0.4449

Note: Under full-rank parameterizations, Type 3 effect tests are replaced by joint tests. The joint test for an effect is a test that all the parameters associated with that effect are zero. Such joint tests might not be equivalent to Type 3 effect tests under GLM parameterization.

Fully adjusted models of Depression score with gender*theme interaction

The MEANS Procedure

Analysis Variable : DscoreR HADS Depression score							
Gender	change in schooling	N Obs	N	Mean	Std Dev	Minimum	Maximum
Female	no	944	944	6.4088983	3.7594935	0	21.0000000
	yes	5	5	5.8000000	1.3038405	4.0000000	7.0000000
Male	no	324	324	5.5061728	3.7179956	0	18.0000000
	yes	2	2	13.0000000	7.0710678	8.0000000	18.0000000
Other	no	18	18	8.1111111	3.7868020	0	16.0000000

The GENMOD Procedure

Model Information		
**Data Set**	WORK.Z	
**Distribution**	Negative Binomial	
**Link Function**	Log	
**Dependent Variable**	DscoreR	HADS Depression score

**Number of Observations Read**	1293
**Number of Observations Used**	1293

Class Level Information				
**Class**	**Value**	**Design Variables**		
**gender**	**Female**	0	0	
	**Male**	1	0	
	**Other**	0	1	
**race**	**black**	1	0	
	**other race**	0	1	
	**white**	0	0	
**age_cat**	**35-54**	1	0	
	**greater than 55**	0	1	
	**less than 35**	0	0	
**inc**	**$100,000 and above**	0	0	0
	**40,000 - $99,999**	1	0	0
	**less than $40,000**	0	1	0
	**missing**	0	0	1
**edu**	**college degree or higher**	0	0	
	**missing**	1	0	
	**no college degree**	0	1	

Fully adjusted models of Depression score with gender*theme interaction

The GENMOD Procedure

Class Level Information				
Class	Value	Design Variables		
kids	missing	1	0	
	no	0	0	
	yes	0	1	
marital_status	married or living as married	1	0	0
	missing	0	1	0
	single	0	0	0
	widowed, divorced	0	0	1
feltunwell	missing	1	0	
	no	0	0	
	yes	0	1	
compare_others	good to excellent	0		
	poor or fair	1		
ratehealth	good to excellent	0		
	poor or fair	1		
x1	no	0		
	yes	1		
x2	no	0		
	yes	1		
x3	no	0		
	yes	1		
x4	no	0		
	yes	1		
x5	no	0		
	yes	1		

The GENMOD Procedure

Class Level Information				
Class	Value	Design Variables		
x6	no	0		
	yes	1		
x8	no	0		
	yes	1		
x9	no	0		
	yes	1		
x10	no	0		
	yes	1		
x11	no	0		
	yes	1		
x12	no	0		
	yes	1		
x13	no	0		
	yes	1		
x14	no	0		
	yes	1		
upset	do not want to answer	1	0	0
	missing	0	0	0
	no	0	1	0
	yes	0	0	1
hadajob	no	1		
	yes	0		
infected	no	0		
	yes	1		

Fully adjusted models of Depression score with gender*theme interaction

The GENMOD Procedure

Criteria For Assessing Goodness Of Fit			
**Criterion**	**DF**	**Value**	**Value/DF**
**Deviance**	1242	1460.5825	1.1760
**Scaled Deviance**	1242	1460.5825	1.1760
**Pearson Chi-Square**	1242	1284.4588	1.0342
**Scaled Pearson X2**	1242	1284.4588	1.0342
**Log Likelihood**		7065.1566	
**Full Log Likelihood**		-3381.4259	
**AIC (smaller is better)**		6866.8517	
**AICC (smaller is better)**		6871.2969	
**BIC (smaller is better)**		7135.4172	

Algorithm converged.

Analysis Of Maximum Likelihood Parameter Estimates									
**Parameter**			**DF**	**Estimate**	**Standard Error**	**Wald 95% Confidence Limits**		**Wald Chi-Square**	**Pr > ChiSq**
**Intercept**			1	1.9342	0.1123	1.7142	2.1543	296.81	<.0001
**gender*x3**	Male	yes	1	0.2018	0.1674	-0.1263	0.5299	1.45	0.2279
**gender*x3**	Other	yes	1	-0.2946	0.6080	-1.4862	0.8970	0.23	0.6279
**x1**	yes		1	0.0075	0.0443	-0.0793	0.0944	0.03	0.8654
**x2**	yes		1	0.3221	0.2144	-0.0981	0.7423	2.26	0.1330
**x3**	yes		1	0.0375	0.0804	-0.1200	0.1950	0.22	0.6410
**x4**	yes		1	0.2097	0.0614	0.0893	0.3300	11.66	0.0006
**x5**	yes		1	0.0695	0.0547	-0.0377	0.1766	1.62	0.2038
**x6**	yes		1	0.0733	0.0562	-0.0368	0.1834	1.70	0.1919
**x8**	yes		1	0.1049	0.0534	0.0003	0.2096	3.86	0.0494
**x9**	yes		1	0.0735	0.1912	-0.3014	0.4483	0.15	0.7009

The GENMOD Procedure

Analysis Of Maximum Likelihood Parameter Estimates									
Parameter			DF	Estimate	Standard Error	Wald 95% Confidence Limits		Wald Chi-Square	Pr > ChiSq
x10	yes		1	0.1342	0.0592	0.0182	0.2503	5.14	0.0234
x11	yes		1	-0.0146	0.1541	-0.3166	0.2874	0.01	0.9243
x12	yes		1	0.0162	0.1991	-0.3741	0.4064	0.01	0.9353
x13	yes		1	0.1426	0.1034	-0.0600	0.3453	1.90	0.1677
x14	yes		1	0.1660	0.1822	-0.1912	0.5232	0.83	0.3624
upset	do not want to answer		1	0.0049	0.2104	-0.4076	0.4174	0.00	0.9814
upset	no		1	-0.0575	0.0418	-0.1393	0.0244	1.89	0.1689
upset	yes		1	0.1314	0.0645	0.0050	0.2578	4.15	0.0416
dur			1	0.0026	0.0006	0.0014	0.0038	17.52	<.0001
support_family			1	-0.0033	0.0007	-0.0046	-0.0020	24.96	<.0001
support_doctor			1	-0.0011	0.0007	-0.0024	0.0003	2.51	0.1134
support_fedgovt			1	0.0001	0.0009	-0.0016	0.0019	0.02	0.8894
support_city			1	0.0023	0.0010	0.0003	0.0042	5.10	0.0239
support_deptpubhealt			1	-0.0016	0.0009	-0.0035	0.0002	3.13	0.0768
support_faithcommuni			1	-0.0020	0.0007	-0.0033	-0.0006	8.28	0.0040
support_socialservic			1	0.0018	0.0009	-0.0001	0.0036	3.56	0.0592
support_neighbors			1	-0.0013	0.0007	-0.0027	0.0000	3.66	0.0556
support_other			1	-0.0015	0.0008	-0.0031	0.0002	3.01	0.0828
infected	yes		1	0.0478	0.1603	-0.2664	0.3620	0.09	0.7656
feltunwell	missing		1	0.1551	0.2942	-0.4216	0.7318	0.28	0.5982
feltunwell	yes		1	0.0954	0.0390	0.0190	0.1718	5.99	0.0144
compare_others	poor or fair		1	0.2493	0.0616	0.1287	0.3700	16.40	<.0001
ratehealth	poor or fair		1	-0.0649	0.0823	-0.2262	0.0963	0.62	0.4300
gender	Male		1	-0.1507	0.0404	-0.2299	-0.0715	13.91	0.0002
gender	Other		1	0.0778	0.1404	-0.1973	0.3529	0.31	0.5794

Fully adjusted models of Depression score with gender*theme interaction

The GENMOD Procedure

Analysis Of Maximum Likelihood Parameter Estimates									
Parameter			DF	Estimate	Standard Error	Wald 95% Confidence Limits		Wald Chi-Square	Pr > ChiSq
race	black		1	-0.0749	0.0684	-0.2091	0.0592	1.20	0.2736
race	other race		1	0.0258	0.0641	-0.0999	0.1514	0.16	0.6878
age_cat	35-54		1	0.0197	0.0512	-0.0806	0.1201	0.15	0.6998
age_cat	greater than 55		1	-0.0804	0.0553	-0.1887	0.0279	2.12	0.1457
inc	40,000 - $99,999		1	0.0576	0.0415	-0.0236	0.1389	1.93	0.1643
inc	less than $40,000		1	0.1566	0.0525	0.0538	0.2595	8.90	0.0028
inc	missing		1	0.1022	0.1074	-0.1083	0.3128	0.91	0.3414
edu	missing		1	-0.4665	0.2705	-0.9967	0.0638	2.97	0.0847
edu	no college degree		1	0.0111	0.0485	-0.0840	0.1063	0.05	0.8191
marital_status	married or living as married		1	0.0846	0.0427	0.0008	0.1684	3.91	0.0479
marital_status	missing		1	0.4316	0.2123	0.0156	0.8476	4.14	0.0420
marital_status	widowed, divorced		1	0.0103	0.0604	-0.1081	0.1287	0.03	0.8642
kids	missing		1	-0.3831	0.2587	-0.8902	0.1240	2.19	0.1387
kids	yes		1	-0.0047	0.0476	-0.0980	0.0887	0.01	0.9218
hadajob	no		1	0.0349	0.0439	-0.0512	0.1210	0.63	0.4271
Dispersion			1	0.1681	0.0141	0.1427	0.1981		

Note: The negative binomial dispersion parameter was estimated by maximum likelihood.

LR Statistics For Joint Tests			
Source	DF	Chi-Square	Pr > ChiSq
gender*x3	2	1.74	0.4180
x1	1	0.03	0.8654
x2	1	2.28	0.1314
x3	1	0.22	0.6410
x4	1	11.64	0.0006

The GENMOD Procedure

LR Statistics For Joint Tests			
Source	DF	Chi-Square	Pr > ChiSq
x5	1	1.62	0.2038
x6	1	1.70	0.1920
x8	1	3.86	0.0495
x9	1	0.15	0.7007
x10	1	5.14	0.0234
x11	1	0.01	0.9243
x12	1	0.01	0.9353
x13	1	1.91	0.1673
x14	1	0.83	0.3618
upset	3	12.09	0.0071
dur	1	17.40	<.0001
support_family	1	24.80	<.0001
support_doctor	1	2.50	0.1135
support_fedgovt	1	0.02	0.8894
support_city	1	5.10	0.0239
support_deptpubhealt	1	3.13	0.0769
support_faithcommuni	1	8.26	0.0041
support_socialservic	1	3.56	0.0592
support_neighbors	1	3.66	0.0557
support_other	1	3.01	0.0829
infected	1	0.09	0.7655
feltunwell	2	6.17	0.0458
compare_others	1	16.39	<.0001
ratehealth	1	0.62	0.4302
gender	2	14.52	0.0007
race	2	1.45	0.4851

The GENMOD Procedure

LR Statistics For Joint Tests			
**Source**	**DF**	**Chi-Square**	**Pr > ChiSq**
**age_cat**	2	5.55	0.0623
**inc**	3	9.21	0.0266
**edu**	2	3.16	0.2064
**marital_status**	3	7.90	0.0482
**kids**	2	2.23	0.3282
**hadajob**	1	0.63	0.4273

Note: Under full-rank parameterizations, Type 3 effect tests are replaced by joint tests. The joint test for an effect is a test that all the parameters associated with that effect are zero. Such joint tests might not be equivalent to Type 3 effect tests under GLM parameterization.

Fully adjusted models of Depression score with gender*theme interaction

The GENMOD Procedure

Model Information		
**Data Set**	WORK.Z	
**Distribution**	Negative Binomial	
**Link Function**	Log	
**Dependent Variable**	DscoreR	HADS Depression score

**Number of Observations Read**	1293
**Number of Observations Used**	1293

Class Level Information				
**Class**	**Value**	**Design Variables**		
**gender**	**Female**	0	0	
	**Male**	1	0	
	**Other**	0	1	
**race**	**black**	1	0	
	**other race**	0	1	
	**white**	0	0	
**age_cat**	**35-54**	1	0	
	**greater than 55**	0	1	
	**less than 35**	0	0	
**inc**	**$100,000 and above**	0	0	0
	**40,000 - $99,999**	1	0	0
	**less than $40,000**	0	1	0
	**missing**	0	0	1
**edu**	**college degree or higher**	0	0	
	**missing**	1	0	
	**no college degree**	0	1	

The GENMOD Procedure

Class Level Information				
**Class**	**Value**	**Design Variables**		
**kids**	**missing**	1	0	
	**no**	0	0	
	**yes**	0	1	
**marital_status**	**married or living as married**	1	0	0
	**missing**	0	1	0
	**single**	0	0	0
	**widowed, divorced**	0	0	1
**feltunwell**	**missing**	1	0	
	**no**	0	0	
	**yes**	0	1	
**compare_others**	**good to excellent**	0		
	**poor or fair**	1		
**ratehealth**	**good to excellent**	0		
	**poor or fair**	1		
**x1**	**no**	0		
	**yes**	1		
**x2**	**no**	0		
	**yes**	1		
**x3**	**no**	0		
	**yes**	1		
**x4**	**no**	0		
	**yes**	1		
**x5**	**no**	0		
	**yes**	1		

The GENMOD Procedure

Class Level Information				
Class	Value	Design Variables		
x6	no	0		
	yes	1		
x8	no	0		
	yes	1		
x9	no	0		
	yes	1		
x10	no	0		
	yes	1		
x11	no	0		
	yes	1		
x12	no	0		
	yes	1		
x13	no	0		
	yes	1		
x14	no	0		
	yes	1		
upset	do not want to answer	1	0	0
	missing	0	0	0
	no	0	1	0
	yes	0	0	1
hadajob	no	1		
	yes	0		
infected	no	0		
	yes	1		

Fully adjusted models of Depression score with gender*theme interaction

The GENMOD Procedure

Criteria For Assessing Goodness Of Fit			
**Criterion**	**DF**	**Value**	**Value/DF**
**Deviance**	1243	1460.2726	1.1748
**Scaled Deviance**	1243	1460.2726	1.1748
**Pearson Chi-Square**	1243	1286.2677	1.0348
**Scaled Pearson X2**	1243	1286.2677	1.0348
**Log Likelihood**		7064.3305	
**Full Log Likelihood**		-3382.2519	
**AIC (smaller is better)**		6866.5039	
**AICC (smaller is better)**		6870.7778	
**BIC (smaller is better)**		7129.9046	

Algorithm converged.

Analysis Of Maximum Likelihood Parameter Estimates									
**Parameter**			**DF**	**Estimate**	**Standard Error**	**Wald 95% Confidence Limits**		**Wald Chi-Square**	**Pr > ChiSq**
**Intercept**			1	1.9336	0.1121	1.7137	2.1534	297.25	<.0001
**gender*x4**	Male	yes	1	-0.0437	0.1436	-0.3252	0.2378	0.09	0.7608
**gender*x4**	Other	yes	0	0.0000	0.0000	0.0000	0.0000	.	.
**x1**	yes		1	0.0032	0.0442	-0.0834	0.0899	0.01	0.9416
**x2**	yes		1	0.3605	0.2121	-0.0552	0.7761	2.89	0.0892
**x3**	yes		1	0.0705	0.0742	-0.0749	0.2158	0.90	0.3420
**x4**	yes		1	0.2190	0.0701	0.0816	0.3564	9.75	0.0018
**x5**	yes		1	0.0709	0.0547	-0.0363	0.1781	1.68	0.1949
**x6**	yes		1	0.0711	0.0562	-0.0391	0.1812	1.60	0.2059
**x8**	yes		1	0.1066	0.0535	0.0017	0.2115	3.96	0.0465
**x9**	yes		1	0.0760	0.1914	-0.2992	0.4511	0.16	0.6914

Fully adjusted models of Depression score with gender*theme interaction

The GENMOD Procedure

Analysis Of Maximum Likelihood Parameter Estimates									
Parameter			DF	Estimate	Standard Error	Wald 95% Confidence Limits		Wald Chi-Square	Pr > ChiSq
**x10**	yes		1	0.1312	0.0592	0.0151	0.2473	4.90	0.0268
**x11**	yes		1	-0.0209	0.1542	-0.3231	0.2813	0.02	0.8923
**x12**	yes		1	0.0172	0.1992	-0.3731	0.4076	0.01	0.9310
**x13**	yes		1	0.1402	0.1034	-0.0625	0.3429	1.84	0.1753
**x14**	yes		1	0.1661	0.1825	-0.1916	0.5239	0.83	0.3627
**upset**	do not want to answer		1	0.0134	0.2103	-0.3988	0.4256	0.00	0.9492
**upset**	no		1	-0.0583	0.0418	-0.1402	0.0236	1.95	0.1628
**upset**	yes		1	0.1332	0.0645	0.0067	0.2597	4.26	0.0390
**dur**			1	0.0026	0.0006	0.0014	0.0038	17.22	<.0001
**support_family**			1	-0.0033	0.0007	-0.0046	-0.0020	24.94	<.0001
**support_doctor**			1	-0.0011	0.0007	-0.0024	0.0003	2.48	0.1156
**support_fedgovt**			1	0.0002	0.0009	-0.0016	0.0019	0.04	0.8436
**support_city**			1	0.0023	0.0010	0.0003	0.0042	5.22	0.0224
**support_deptpubhealt**			1	-0.0017	0.0009	-0.0035	0.0002	3.18	0.0746
**support_faithcommuni**			1	-0.0020	0.0007	-0.0034	-0.0007	8.51	0.0035
**support_socialservic**			1	0.0018	0.0009	-0.0001	0.0036	3.54	0.0598
**support_neighbors**			1	-0.0013	0.0007	-0.0027	0.0001	3.55	0.0596
**support_other**			1	-0.0014	0.0008	-0.0031	0.0002	2.95	0.0858
**infected**	yes		1	0.0452	0.1605	-0.2695	0.3599	0.08	0.7783
**feltunwell**	missing		1	0.1583	0.2944	-0.4188	0.7353	0.29	0.5909
**feltunwell**	yes		1	0.0951	0.0390	0.0187	0.1715	5.95	0.0147
**compare_others**	poor or fair		1	0.2430	0.0613	0.1230	0.3631	15.74	<.0001
**ratehealth**	poor or fair		1	-0.0611	0.0822	-0.2222	0.1000	0.55	0.4572
**gender**	Male		1	-0.1361	0.0410	-0.2164	-0.0558	11.03	0.0009
**gender**	Other		1	0.0642	0.1363	-0.2030	0.3313	0.22	0.6378

Fully adjusted models of Depression score with gender*theme interaction

The GENMOD Procedure

Analysis Of Maximum Likelihood Parameter Estimates									
Parameter			DF	Estimate	Standard Error	Wald 95% Confidence Limits		Wald Chi-Square	Pr > ChiSq
race	black		1	-0.0781	0.0685	-0.2123	0.0561	1.30	0.2537
race	other race		1	0.0222	0.0641	-0.1034	0.1479	0.12	0.7286
age_cat	35-54		1	0.0207	0.0512	-0.0797	0.1211	0.16	0.6864
age_cat	greater than 55		1	-0.0815	0.0553	-0.1898	0.0268	2.18	0.1402
inc	40,000 - $99,999		1	0.0573	0.0414	-0.0239	0.1386	1.91	0.1665
inc	less than $40,000		1	0.1567	0.0525	0.0537	0.2597	8.89	0.0029
inc	missing		1	0.1095	0.1073	-0.1008	0.3197	1.04	0.3074
edu	missing		1	-0.4685	0.2707	-0.9990	0.0619	3.00	0.0834
edu	no college degree		1	0.0100	0.0486	-0.0852	0.1052	0.04	0.8375
marital_status	married or living as married		1	0.0844	0.0428	0.0005	0.1682	3.89	0.0486
marital_status	missing		1	0.4283	0.2124	0.0119	0.8447	4.06	0.0438
marital_status	widowed, divorced		1	0.0129	0.0606	-0.1059	0.1317	0.05	0.8317
kids	missing		1	-0.3958	0.2587	-0.9028	0.1112	2.34	0.1260
kids	yes		1	-0.0064	0.0477	-0.0998	0.0871	0.02	0.8936
hadajob	no		1	0.0331	0.0440	-0.0531	0.1193	0.57	0.4514
Dispersion			1	0.1687	0.0141	0.1432	0.1987		

Note: The negative binomial dispersion parameter was estimated by maximum likelihood.

LR Statistics For Joint Tests			
Source	DF	Chi-Square	Pr > ChiSq
gender*x4	2	1.91	0.3853
x1	1	0.01	0.9416
x2	1	2.92	0.0876
x3	1	0.90	0.3420
x4	1	9.76	0.0018

The GENMOD Procedure

LR Statistics For Joint Tests			
Source	DF	Chi-Square	Pr > ChiSq
x5	1	1.68	0.1949
x6	1	1.60	0.2059
x8	1	3.96	0.0466
x9	1	0.16	0.6912
x10	1	4.90	0.0268
x11	1	0.02	0.8923
x12	1	0.01	0.9310
x13	1	1.84	0.1748
x14	1	0.83	0.3622
upset	3	12.44	0.0060
dur	1	17.11	<.0001
support_family	1	24.79	<.0001
support_doctor	1	2.47	0.1157
support_fedgovt	1	0.04	0.8436
support_city	1	5.21	0.0224
support_deptpubhealt	1	3.18	0.0747
support_faithcommuni	1	8.48	0.0036
support_socialservic	1	3.54	0.0598
support_neighbors	1	3.55	0.0597
support_other	1	2.95	0.0859
infected	1	0.08	0.7782
feltunwell	2	6.14	0.0465
compare_others	1	15.73	<.0001
ratehealth	1	0.55	0.4573
gender	2	11.49	0.0032
race	2	1.50	0.4723

The GENMOD Procedure

LR Statistics For Joint Tests			
**Source**	**DF**	**Chi-Square**	**Pr > ChiSq**
**age_cat**	2	5.75	0.0563
**inc**	3	9.27	0.0259
**edu**	2	3.17	0.2053
**marital_status**	3	7.72	0.0523
**kids**	2	2.38	0.3040
**hadajob**	1	0.57	0.4515

Note: Under full-rank parameterizations, Type 3 effect tests are replaced by joint tests. The joint test for an effect is a test that all the parameters associated with that effect are zero. Such joint tests might not be equivalent to Type 3 effect tests under GLM parameterization.

Fully adjusted models of Depression score with gender*theme interaction

The GENMOD Procedure

Model Information		
**Data Set**	WORK.Z	
**Distribution**	Negative Binomial	
**Link Function**	Log	
**Dependent Variable**	DscoreR	HADS Depression score

**Number of Observations Read**	1293
**Number of Observations Used**	1293

Class Level Information				
**Class**	**Value**	**Design Variables**		
**gender**	**Female**	0	0	
	**Male**	1	0	
	**Other**	0	1	
**race**	**black**	1	0	
	**other race**	0	1	
	**white**	0	0	
**age_cat**	**35-54**	1	0	
	**greater than 55**	0	1	
	**less than 35**	0	0	
**inc**	**$100,000 and above**	0	0	0
	**40,000 - $99,999**	1	0	0
	**less than $40,000**	0	1	0
	**missing**	0	0	1
**edu**	**college degree or higher**	0	0	
	**missing**	1	0	
	**no college degree**	0	1	

Fully adjusted models of Depression score with gender*theme interaction

The GENMOD Procedure

Class Level Information				
Class	Value	Design Variables		
kids	missing	1	0	
	no	0	0	
	yes	0	1	
marital_status	married or living as married	1	0	0
	missing	0	1	0
	single	0	0	0
	widowed, divorced	0	0	1
feltunwell	missing	1	0	
	no	0	0	
	yes	0	1	
compare_others	good to excellent	0		
	poor or fair	1		
ratehealth	good to excellent	0		
	poor or fair	1		
x1	no	0		
	yes	1		
x2	no	0		
	yes	1		
x3	no	0		
	yes	1		
x4	no	0		
	yes	1		
x5	no	0		
	yes	1		

The GENMOD Procedure

Class Level Information				
Class	Value	Design Variables		
x6	no	0		
	yes	1		
x8	no	0		
	yes	1		
x9	no	0		
	yes	1		
x10	no	0		
	yes	1		
x11	no	0		
	yes	1		
x12	no	0		
	yes	1		
x13	no	0		
	yes	1		
x14	no	0		
	yes	1		
upset	do not want to answer	1	0	0
	missing	0	0	0
	no	0	1	0
	yes	0	0	1
hadajob	no	1		
	yes	0		
infected	no	0		
	yes	1		

Fully adjusted models of Depression score with gender*theme interaction

The GENMOD Procedure

Criteria For Assessing Goodness Of Fit			
**Criterion**	**DF**	**Value**	**Value/DF**
**Deviance**	1242	1461.0092	1.1763
**Scaled Deviance**	1242	1461.0092	1.1763
**Pearson Chi-Square**	1242	1286.9391	1.0362
**Scaled Pearson X2**	1242	1286.9391	1.0362
**Log Likelihood**		7065.1054	
**Full Log Likelihood**		-3381.4771	
**AIC (smaller is better)**		6866.9541	
**AICC (smaller is better)**		6871.3993	
**BIC (smaller is better)**		7135.5196	

Algorithm converged.

Analysis Of Maximum Likelihood Parameter Estimates									
**Parameter**			**DF**	**Estimate**	**Standard Error**	**Wald 95% Confidence Limits**		**Wald Chi-Square**	**Pr > ChiSq**
**Intercept**			1	1.9325	0.1119	1.7132	2.1519	298.16	<.0001
**gender*x5**	Male	yes	1	0.0434	0.1305	-0.2124	0.2991	0.11	0.7398
**gender*x5**	Other	yes	1	0.6288	0.5161	-0.3827	1.6403	1.48	0.2231
**x1**	yes		1	0.0039	0.0442	-0.0826	0.0905	0.01	0.9294
**x2**	yes		1	0.3626	0.2119	-0.0527	0.7779	2.93	0.0870
**x3**	yes		1	0.0709	0.0741	-0.0743	0.2161	0.92	0.3387
**x4**	yes		1	0.2083	0.0614	0.0880	0.3287	11.51	0.0007
**x5**	yes		1	0.0539	0.0621	-0.0678	0.1755	0.75	0.3856
**x6**	yes		1	0.0730	0.0561	-0.0371	0.1830	1.69	0.1937
**x8**	yes		1	0.1078	0.0534	0.0031	0.2125	4.07	0.0437
**x9**	yes		1	0.0771	0.1912	-0.2977	0.4519	0.16	0.6868

The GENMOD Procedure

Analysis Of Maximum Likelihood Parameter Estimates									
Parameter			DF	Estimate	Standard Error	Wald 95% Confidence Limits		Wald Chi-Square	Pr > ChiSq
**x10**	yes		1	0.1337	0.0593	0.0175	0.2499	5.08	0.0242
**x11**	yes		1	-0.0227	0.1539	-0.3243	0.2789	0.02	0.8828
**x12**	yes		1	0.0284	0.1991	-0.3619	0.4187	0.02	0.8865
**x13**	yes		1	0.1410	0.1033	-0.0615	0.3436	1.86	0.1723
**x14**	yes		1	0.1707	0.1822	-0.1865	0.5278	0.88	0.3490
**upset**	do not want to answer		1	0.0273	0.2103	-0.3849	0.4395	0.02	0.8968
**upset**	no		1	-0.0551	0.0419	-0.1372	0.0270	1.73	0.1885
**upset**	yes		1	0.1355	0.0646	0.0089	0.2622	4.40	0.0360
**dur**			1	0.0025	0.0006	0.0013	0.0038	16.88	<.0001
**support_family**			1	-0.0033	0.0007	-0.0046	-0.0020	25.14	<.0001
**support_doctor**			1	-0.0011	0.0007	-0.0024	0.0003	2.51	0.1128
**support_fedgovt**			1	0.0002	0.0009	-0.0016	0.0019	0.04	0.8464
**support_city**			1	0.0023	0.0010	0.0004	0.0043	5.43	0.0198
**support_deptpubhealt**			1	-0.0017	0.0009	-0.0035	0.0002	3.16	0.0756
**support_faithcommuni**			1	-0.0020	0.0007	-0.0034	-0.0007	8.57	0.0034
**support_socialservic**			1	0.0017	0.0009	-0.0001	0.0036	3.50	0.0613
**support_neighbors**			1	-0.0013	0.0007	-0.0027	0.0000	3.59	0.0582
**support_other**			1	-0.0015	0.0008	-0.0031	0.0002	2.99	0.0838
**infected**	yes		1	0.0480	0.1603	-0.2663	0.3622	0.09	0.7648
**feltunwell**	missing		1	0.1574	0.2942	-0.4191	0.7340	0.29	0.5925
**feltunwell**	yes		1	0.0966	0.0390	0.0202	0.1730	6.14	0.0132
**compare_others**	poor or fair		1	0.2446	0.0613	0.1245	0.3647	15.92	<.0001
**ratehealth**	poor or fair		1	-0.0618	0.0821	-0.2227	0.0992	0.57	0.4518
**gender**	Male		1	-0.1438	0.0411	-0.2244	-0.0632	12.23	0.0005
**gender**	Other		1	0.0130	0.1417	-0.2649	0.2908	0.01	0.9272

Fully adjusted models of Depression score with gender*theme interaction

The GENMOD Procedure

Analysis Of Maximum Likelihood Parameter Estimates									
Parameter			DF	Estimate	Standard Error	Wald 95% Confidence Limits		Wald Chi-Square	Pr > ChiSq
race	black		1	-0.0771	0.0684	-0.2111	0.0570	1.27	0.2598
race	other race		1	0.0233	0.0641	-0.1023	0.1488	0.13	0.7162
age_cat	35-54		1	0.0219	0.0512	-0.0784	0.1221	0.18	0.6694
age_cat	greater than 55		1	-0.0796	0.0552	-0.1878	0.0286	2.08	0.1494
inc	40,000 - $99,999		1	0.0577	0.0414	-0.0235	0.1389	1.94	0.1638
inc	less than $40,000		1	0.1559	0.0525	0.0530	0.2588	8.82	0.0030
inc	missing		1	0.1090	0.1072	-0.1011	0.3191	1.03	0.3094
edu	missing		1	-0.4707	0.2705	-1.0009	0.0595	3.03	0.0819
edu	no college degree		1	0.0100	0.0485	-0.0851	0.1051	0.04	0.8369
marital_status	married or living as married		1	0.0850	0.0427	0.0012	0.1687	3.95	0.0468
marital_status	missing		1	0.4301	0.2125	0.0137	0.8465	4.10	0.0429
marital_status	widowed, divorced		1	0.0121	0.0604	-0.1062	0.1305	0.04	0.8409
kids	missing		1	-0.3934	0.2585	-0.9001	0.1133	2.32	0.1281
kids	yes		1	-0.0063	0.0476	-0.0997	0.0870	0.02	0.8942
hadajob	no		1	0.0353	0.0439	-0.0508	0.1214	0.64	0.4222
Dispersion			1	0.1680	0.0141	0.1426	0.1980		

Note: The negative binomial dispersion parameter was estimated by maximum likelihood.

LR Statistics For Joint Tests			
Source	DF	Chi-Square	Pr > ChiSq
gender*x5	2	1.64	0.4399
x1	1	0.01	0.9294
x2	1	2.96	0.0855
x3	1	0.92	0.3387
x4	1	11.49	0.0007

The GENMOD Procedure

LR Statistics For Joint Tests			
Source	DF	Chi-Square	Pr > ChiSq
x5	1	0.75	0.3855
x6	1	1.69	0.1937
x8	1	4.07	0.0438
x9	1	0.16	0.6865
x10	1	5.08	0.0242
x11	1	0.02	0.8828
x12	1	0.02	0.8865
x13	1	1.87	0.1718
x14	1	0.88	0.3485
upset	3	12.24	0.0066
dur	1	16.78	<.0001
support_family	1	24.99	<.0001
support_doctor	1	2.51	0.1129
support_fedgovt	1	0.04	0.8464
support_city	1	5.42	0.0199
support_deptpubhealt	1	3.16	0.0756
support_faithcommuni	1	8.55	0.0035
support_socialservic	1	3.50	0.0613
support_neighbors	1	3.58	0.0583
support_other	1	2.99	0.0839
infected	1	0.09	0.7647
feltunwell	2	6.33	0.0422
compare_others	1	15.92	<.0001
ratehealth	1	0.57	0.4519
gender	2	12.30	0.0021
race	2	1.48	0.4766

The GENMOD Procedure

LR Statistics For Joint Tests			
**Source**	**DF**	**Chi-Square**	**Pr > ChiSq**
**age_cat**	2	5.68	0.0586
**inc**	3	9.18	0.0270
**edu**	2	3.20	0.2019
**marital_status**	3	7.85	0.0493
**kids**	2	2.36	0.3079
**hadajob**	1	0.64	0.4224

Note: Under full-rank parameterizations, Type 3 effect tests are replaced by joint tests. The joint test for an effect is a test that all the parameters associated with that effect are zero. Such joint tests might not be equivalent to Type 3 effect tests under GLM parameterization.

The GENMOD Procedure

Model Information		
**Data Set**	WORK.Z	
**Distribution**	Negative Binomial	
**Link Function**	Log	
**Dependent Variable**	DscoreR	HADS Depression score

**Number of Observations Read**	1293
**Number of Observations Used**	1293

Class Level Information				
**Class**	**Value**	**Design Variables**		
**gender**	**Female**	0	0	
	**Male**	1	0	
	**Other**	0	1	
**race**	**black**	1	0	
	**other race**	0	1	
	**white**	0	0	
**age_cat**	**35-54**	1	0	
	**greater than 55**	0	1	
	**less than 35**	0	0	
**inc**	**$100,000 and above**	0	0	0
	**40,000 - $99,999**	1	0	0
	**less than $40,000**	0	1	0
	**missing**	0	0	1
**edu**	**college degree or higher**	0	0	
	**missing**	1	0	
	**no college degree**	0	1	

The GENMOD Procedure

Class Level Information				
Class	Value	Design Variables		
kids	missing	1	0	
	no	0	0	
	yes	0	1	
marital_status	married or living as married	1	0	0
	missing	0	1	0
	single	0	0	0
	widowed, divorced	0	0	1
feltunwell	missing	1	0	
	no	0	0	
	yes	0	1	
compare_others	good to excellent	0		
	poor or fair	1		
ratehealth	good to excellent	0		
	poor or fair	1		
x1	no	0		
	yes	1		
x2	no	0		
	yes	1		
x3	no	0		
	yes	1		
x4	no	0		
	yes	1		
x5	no	0		
	yes	1		

The GENMOD Procedure

Class Level Information				
**Class**	**Value**	**Design Variables**		
**x6**	**no**	0		
	**yes**	1		
**x8**	**no**	0		
	**yes**	1		
**x9**	**no**	0		
	**yes**	1		
**x10**	**no**	0		
	**yes**	1		
**x11**	**no**	0		
	**yes**	1		
**x12**	**no**	0		
	**yes**	1		
**x13**	**no**	0		
	**yes**	1		
**x14**	**no**	0		
	**yes**	1		
**upset**	**do not want to answer**	1	0	0
	**missing**	0	0	0
	**no**	0	1	0
	**yes**	0	0	1
**hadajob**	**no**	1		
	**yes**	0		
**infected**	**no**	0		
	**yes**	1		

Fully adjusted models of Depression score with gender*theme interaction

The GENMOD Procedure

Criteria For Assessing Goodness Of Fit			
**Criterion**	**DF**	**Value**	**Value/DF**
**Deviance**	1242	1460.4696	1.1759
**Scaled Deviance**	1242	1460.4696	1.1759
**Pearson Chi-Square**	1242	1286.3221	1.0357
**Scaled Pearson X2**	1242	1286.3221	1.0357
**Log Likelihood**		7064.4478	
**Full Log Likelihood**		-3382.1346	
**AIC (smaller is better)**		6868.2693	
**AICC (smaller is better)**		6872.7144	
**BIC (smaller is better)**		7136.8347	

Algorithm converged.

Analysis Of Maximum Likelihood Parameter Estimates									
**Parameter**			**DF**	**Estimate**	**Standard Error**	**Wald 95% Confidence Limits**		**Wald Chi-Square**	**Pr > ChiSq**
**Intercept**			1	1.9326	0.1121	1.7130	2.1523	297.37	<.0001
**gender*x6**	Male	yes	1	0.0120	0.1336	-0.2499	0.2739	0.01	0.9283
**gender*x6**	Other	yes	1	-0.2045	0.3680	-0.9257	0.5167	0.31	0.5783
**x1**	yes		1	0.0039	0.0442	-0.0828	0.0905	0.01	0.9303
**x2**	yes		1	0.3613	0.2122	-0.0545	0.7771	2.90	0.0886
**x3**	yes		1	0.0708	0.0742	-0.0746	0.2162	0.91	0.3398
**x4**	yes		1	0.2088	0.0615	0.0883	0.3292	11.54	0.0007
**x5**	yes		1	0.0704	0.0547	-0.0368	0.1776	1.66	0.1978
**x6**	yes		1	0.0734	0.0640	-0.0520	0.1987	1.32	0.2512
**x8**	yes		1	0.1050	0.0535	0.0002	0.2098	3.85	0.0497
**x9**	yes		1	0.0761	0.1914	-0.2990	0.4511	0.16	0.6909

The GENMOD Procedure

Analysis Of Maximum Likelihood Parameter Estimates									
Parameter			DF	Estimate	Standard Error	Wald 95% Confidence Limits		Wald Chi-Square	Pr > ChiSq
x10	yes		1	0.1314	0.0592	0.0153	0.2474	4.92	0.0265
x11	yes		1	-0.0225	0.1540	-0.3243	0.2794	0.02	0.8841
x12	yes		1	0.0130	0.1995	-0.3780	0.4039	0.00	0.9482
x13	yes		1	0.1399	0.1034	-0.0628	0.3426	1.83	0.1762
x14	yes		1	0.1681	0.1824	-0.1894	0.5255	0.85	0.3567
upset	do not want to answer		1	0.0042	0.2110	-0.4094	0.4178	0.00	0.9842
upset	no		1	-0.0581	0.0418	-0.1401	0.0238	1.94	0.1641
upset	yes		1	0.1333	0.0645	0.0068	0.2597	4.26	0.0389
dur			1	0.0026	0.0006	0.0014	0.0038	17.16	<.0001
support_family			1	-0.0033	0.0007	-0.0046	-0.0020	24.98	<.0001
support_doctor			1	-0.0011	0.0007	-0.0024	0.0002	2.55	0.1105
support_fedgovt			1	0.0002	0.0009	-0.0016	0.0019	0.04	0.8512
support_city			1	0.0023	0.0010	0.0003	0.0042	5.18	0.0229
support_deptpubhealt			1	-0.0016	0.0009	-0.0035	0.0002	3.10	0.0781
support_faithcommuni			1	-0.0020	0.0007	-0.0034	-0.0007	8.47	0.0036
support_socialservic			1	0.0018	0.0009	-0.0001	0.0036	3.57	0.0587
support_neighbors			1	-0.0013	0.0007	-0.0027	0.0000	3.59	0.0580
support_other			1	-0.0014	0.0008	-0.0031	0.0002	2.83	0.0927
infected	yes		1	0.0477	0.1604	-0.2666	0.3620	0.09	0.7661
feltunwell	missing		1	0.1582	0.2944	-0.4188	0.7351	0.29	0.5910
feltunwell	yes		1	0.0950	0.0390	0.0186	0.1714	5.94	0.0148
compare_others	poor or fair		1	0.2435	0.0613	0.1234	0.3636	15.79	<.0001
ratehealth	poor or fair		1	-0.0621	0.0823	-0.2233	0.0991	0.57	0.4504
gender	Male		1	-0.1406	0.0410	-0.2210	-0.0602	11.74	0.0006
gender	Other		1	0.0955	0.1481	-0.1948	0.3857	0.42	0.5192

Fully adjusted models of Depression score with gender*theme interaction

The GENMOD Procedure

Analysis Of Maximum Likelihood Parameter Estimates									
Parameter			DF	Estimate	Standard Error	Wald 95% Confidence Limits		Wald Chi-Square	Pr > ChiSq
**race**	black		1	-0.0775	0.0685	-0.2117	0.0567	1.28	0.2579
**race**	other race		1	0.0211	0.0641	-0.1045	0.1468	0.11	0.7416
**age_cat**	35-54		1	0.0202	0.0512	-0.0801	0.1206	0.16	0.6928
**age_cat**	greater than 55		1	-0.0813	0.0553	-0.1895	0.0270	2.16	0.1414
**inc**	40,000 - $99,999		1	0.0581	0.0415	-0.0233	0.1395	1.96	0.1618
**inc**	less than $40,000		1	0.1573	0.0525	0.0543	0.2603	8.96	0.0028
**inc**	missing		1	0.1104	0.1073	-0.0998	0.3206	1.06	0.3034
**edu**	missing		1	-0.4677	0.2707	-0.9983	0.0629	2.98	0.0840
**edu**	no college degree		1	0.0100	0.0486	-0.0852	0.1052	0.04	0.8368
**marital_status**	married or living as married		1	0.0845	0.0428	0.0007	0.1684	3.90	0.0482
**marital_status**	missing		1	0.4302	0.2124	0.0138	0.8465	4.10	0.0429
**marital_status**	widowed, divorced		1	0.0116	0.0604	-0.1068	0.1301	0.04	0.8473
**kids**	missing		1	-0.3946	0.2587	-0.9015	0.1124	2.33	0.1271
**kids**	yes		1	-0.0061	0.0476	-0.0995	0.0873	0.02	0.8980
**hadajob**	no		1	0.0342	0.0439	-0.0520	0.1203	0.60	0.4369
**Dispersion**			1	0.1685	0.0141	0.1431	0.1986		

Note: The negative binomial dispersion parameter was estimated by maximum likelihood.

LR Statistics For Joint Tests			
Source	DF	Chi-Square	Pr > ChiSq
**gender*x6**	2	0.33	0.8491
**x1**	1	0.01	0.9303
**x2**	1	2.93	0.0870
**x3**	1	0.91	0.3398
**x4**	1	11.52	0.0007

Fully adjusted models of Depression score with gender*theme interaction

The GENMOD Procedure

LR Statistics For Joint Tests			
**Source**	**DF**	**Chi-Square**	**Pr > ChiSq**
**x5**	1	1.66	0.1979
**x6**	1	1.32	0.2512
**x8**	1	3.85	0.0498
**x9**	1	0.16	0.6907
**x10**	1	4.92	0.0265
**x11**	1	0.02	0.8841
**x12**	1	0.00	0.9482
**x13**	1	1.83	0.1757
**x14**	1	0.85	0.3562
**upset**	3	12.39	0.0062
**dur**	1	17.05	<.0001
**support_family**	1	24.83	<.0001
**support_doctor**	1	2.55	0.1105
**support_fedgovt**	1	0.04	0.8512
**support_city**	1	5.17	0.0230
**support_deptpubhealt**	1	3.10	0.0782
**support_faithcommuni**	1	8.44	0.0037
**support_socialservic**	1	3.57	0.0587
**support_neighbors**	1	3.59	0.0581
**support_other**	1	2.83	0.0928
**infected**	1	0.09	0.7660
**feltunwell**	2	6.13	0.0466
**compare_others**	1	15.78	<.0001
**ratehealth**	1	0.57	0.4505
**gender**	2	12.46	0.0020
**race**	2	1.46	0.4815

The GENMOD Procedure

LR Statistics For Joint Tests			
Source	DF	Chi-Square	Pr > ChiSq
age_cat	2	5.69	0.0581
inc	3	9.33	0.0252
edu	2	3.15	0.2065
marital_status	3	7.81	0.0500
kids	2	2.37	0.3062
hadajob	1	0.60	0.4371

Note: Under full-rank parameterizations, Type 3 effect tests are replaced by joint tests. The joint test for an effect is a test that all the parameters associated with that effect are zero. Such joint tests might not be equivalent to Type 3 effect tests under GLM parameterization.

The GENMOD Procedure

Model Information		
**Data Set**	WORK.Z	
**Distribution**	Negative Binomial	
**Link Function**	Log	
**Dependent Variable**	DscoreR	HADS Depression score

**Number of Observations Read**	1293
**Number of Observations Used**	1293

Class Level Information				
**Class**	**Value**	**Design Variables**		
**gender**	**Female**	0	0	
	**Male**	1	0	
	**Other**	0	1	
**race**	**black**	1	0	
	**other race**	0	1	
	**white**	0	0	
**age_cat**	**35-54**	1	0	
	**greater than 55**	0	1	
	**less than 35**	0	0	
**inc**	**$100,000 and above**	0	0	0
	**40,000 - $99,999**	1	0	0
	**less than $40,000**	0	1	0
	**missing**	0	0	1
**edu**	**college degree or higher**	0	0	
	**missing**	1	0	
	**no college degree**	0	1	

The GENMOD Procedure

Class Level Information				
Class	Value	Design Variables		
kids	missing	1	0	
	no	0	0	
	yes	0	1	
marital_status	married or living as married	1	0	0
	missing	0	1	0
	single	0	0	0
	widowed, divorced	0	0	1
feltunwell	missing	1	0	
	no	0	0	
	yes	0	1	
compare_others	good to excellent	0		
	poor or fair	1		
ratehealth	good to excellent	0		
	poor or fair	1		
x1	no	0		
	yes	1		
x2	no	0		
	yes	1		
x3	no	0		
	yes	1		
x4	no	0		
	yes	1		
x5	no	0		
	yes	1		

The GENMOD Procedure

Class Level Information				
Class	Value	Design Variables		
x6	no	0		
	yes	1		
x8	no	0		
	yes	1		
x9	no	0		
	yes	1		
x10	no	0		
	yes	1		
x11	no	0		
	yes	1		
x12	no	0		
	yes	1		
x13	no	0		
	yes	1		
x14	no	0		
	yes	1		
upset	do not want to answer	1	0	0
	missing	0	0	0
	no	0	1	0
	yes	0	0	1
hadajob	no	1		
	yes	0		
infected	no	0		
	yes	1		

Fully adjusted models of Depression score with gender*theme interaction

The GENMOD Procedure

Criteria For Assessing Goodness Of Fit			
**Criterion**	**DF**	**Value**	**Value/DF**
**Deviance**	1242	1460.2793	1.1757
**Scaled Deviance**	1242	1460.2793	1.1757
**Pearson Chi-Square**	1242	1288.2528	1.0372
**Scaled Pearson X2**	1242	1288.2528	1.0372
**Log Likelihood**		7065.1915	
**Full Log Likelihood**		-3381.3910	
**AIC (smaller is better)**		6866.7820	
**AICC (smaller is better)**		6871.2271	
**BIC (smaller is better)**		7135.3474	

Algorithm converged.

Analysis Of Maximum Likelihood Parameter Estimates									
**Parameter**			**DF**	**Estimate**	**Standard Error**	**Wald 95% Confidence Limits**		**Wald Chi-Square**	**Pr > ChiSq**
**Intercept**			1	1.9330	0.1121	1.7133	2.1526	297.49	<.0001
**gender*x8**	Male	yes	1	-0.0139	0.1440	-0.2962	0.2683	0.01	0.9229
**gender*x8**	Other	yes	1	-0.4464	0.3302	-1.0935	0.2007	1.83	0.1763
**x1**	yes		1	0.0034	0.0442	-0.0832	0.0900	0.01	0.9390
**x2**	yes		1	0.3625	0.2119	-0.0529	0.7779	2.93	0.0872
**x3**	yes		1	0.0693	0.0741	-0.0760	0.2146	0.87	0.3497
**x4**	yes		1	0.2105	0.0615	0.0899	0.3310	11.70	0.0006
**x5**	yes		1	0.0697	0.0547	-0.0374	0.1769	1.63	0.2022
**x6**	yes		1	0.0695	0.0562	-0.0406	0.1797	1.53	0.2161
**x8**	yes		1	0.1190	0.0586	0.0040	0.2339	4.12	0.0425
**x9**	yes		1	0.0728	0.1913	-0.3021	0.4478	0.15	0.7034

The GENMOD Procedure

Analysis Of Maximum Likelihood Parameter Estimates

Parameter			DF	Estimate	Standard Error	Wald 95% Confidence Limits		Wald Chi-Square	Pr > ChiSq
x10	yes		1	0.1323	0.0593	0.0161	0.2484	4.98	0.0256
x11	yes		1	-0.0220	0.1540	-0.3238	0.2798	0.02	0.8864
x12	yes		1	0.0078	0.1993	-0.3828	0.3984	0.00	0.9687
x13	yes		1	0.1523	0.1037	-0.0509	0.3555	2.16	0.1418
x14	yes		1	0.1667	0.1823	-0.1906	0.5240	0.84	0.3606
upset	do not want to answer		1	0.0628	0.2129	-0.3544	0.4800	0.09	0.7679
upset	no		1	-0.0575	0.0418	-0.1394	0.0243	1.90	0.1684
upset	yes		1	0.1336	0.0645	0.0071	0.2600	4.29	0.0384
dur			1	0.0026	0.0006	0.0014	0.0038	17.61	<.0001
support_family			1	-0.0033	0.0007	-0.0046	-0.0020	24.55	<.0001
support_doctor			1	-0.0011	0.0007	-0.0024	0.0002	2.52	0.1126
support_fedgovt			1	0.0002	0.0009	-0.0016	0.0019	0.04	0.8371
support_city			1	0.0024	0.0010	0.0004	0.0043	5.54	0.0186
support_deptpubhealt			1	-0.0017	0.0009	-0.0036	0.0001	3.52	0.0605
support_faithcommuni			1	-0.0020	0.0007	-0.0034	-0.0007	8.61	0.0033
support_socialservic			1	0.0018	0.0009	-0.0000	0.0036	3.64	0.0565
support_neighbors			1	-0.0014	0.0007	-0.0028	-0.0000	3.88	0.0488
support_other			1	-0.0015	0.0008	-0.0031	0.0002	3.03	0.0819
infected	yes		1	0.0460	0.1603	-0.2683	0.3602	0.08	0.7743
feltunwell	missing		1	0.1624	0.2942	-0.4141	0.7390	0.30	0.5808
feltunwell	yes		1	0.0952	0.0390	0.0187	0.1716	5.95	0.0147
compare_others	poor or fair		1	0.2436	0.0612	0.1236	0.3636	15.83	<.0001
ratehealth	poor or fair		1	-0.0679	0.0823	-0.2292	0.0934	0.68	0.4094
gender	Male		1	-0.1378	0.0409	-0.2178	-0.0577	11.37	0.0007
gender	Other		1	0.1498	0.1510	-0.1462	0.4458	0.98	0.3212

Fully adjusted models of Depression score with gender*theme interaction

The GENMOD Procedure

Analysis Of Maximum Likelihood Parameter Estimates									
Parameter			DF	Estimate	Standard Error	Wald 95% Confidence Limits		Wald Chi-Square	Pr > ChiSq
race	black		1	-0.0766	0.0684	-0.2106	0.0575	1.25	0.2631
race	other race		1	0.0213	0.0641	-0.1042	0.1469	0.11	0.7393
age_cat	35-54		1	0.0201	0.0512	-0.0802	0.1205	0.15	0.6943
age_cat	greater than 55		1	-0.0812	0.0552	-0.1894	0.0270	2.16	0.1415
inc	40,000 - $99,999		1	0.0566	0.0414	-0.0247	0.1378	1.86	0.1723
inc	less than $40,000		1	0.1554	0.0525	0.0525	0.2583	8.76	0.0031
inc	missing		1	0.1086	0.1072	-0.1015	0.3187	1.03	0.3109
edu	missing		1	-0.4727	0.2707	-1.0032	0.0578	3.05	0.0807
edu	no college degree		1	0.0087	0.0485	-0.0864	0.1039	0.03	0.8577
marital_status	married or living as married		1	0.0831	0.0428	-0.0007	0.1669	3.78	0.0520
marital_status	missing		1	0.4296	0.2124	0.0133	0.8459	4.09	0.0431
marital_status	widowed, divorced		1	0.0114	0.0604	-0.1070	0.1297	0.04	0.8506
kids	missing		1	-0.3921	0.2594	-0.9006	0.1164	2.28	0.1307
kids	yes		1	-0.0028	0.0477	-0.0963	0.0906	0.00	0.9524
hadajob	no		1	0.0351	0.0439	-0.0510	0.1212	0.64	0.4240
Dispersion			1	0.1682	0.0141	0.1428	0.1982		

Note: The negative binomial dispersion parameter was estimated by maximum likelihood.

LR Statistics For Joint Tests			
Source	DF	Chi-Square	Pr > ChiSq
gender*x8	2	1.81	0.4036
x1	1	0.01	0.9390
x2	1	2.95	0.0857
x3	1	0.87	0.3497
x4	1	11.68	0.0006

Fully adjusted models of Depression score with gender*theme interaction

The GENMOD Procedure

LR Statistics For Joint Tests			
Source	DF	Chi-Square	Pr > ChiSq
x5	1	1.63	0.2022
x6	1	1.53	0.2161
x8	1	4.11	0.0425
x9	1	0.15	0.7032
x10	1	4.98	0.0257
x11	1	0.02	0.8864
x12	1	0.00	0.9687
x13	1	2.16	0.1414
x14	1	0.84	0.3600
upset	3	12.53	0.0058
dur	1	17.50	<.0001
support_family	1	24.41	<.0001
support_doctor	1	2.52	0.1127
support_fedgovt	1	0.04	0.8371
support_city	1	5.53	0.0187
support_deptpubhealt	1	3.52	0.0606
support_faithcommuni	1	8.59	0.0034
support_socialservic	1	3.64	0.0565
support_neighbors	1	3.88	0.0489
support_other	1	3.03	0.0819
infected	1	0.08	0.7743
feltunwell	2	6.15	0.0461
compare_others	1	15.82	<.0001
ratehealth	1	0.68	0.4096
gender	2	12.85	0.0016
race	2	1.44	0.4875

The GENMOD Procedure

LR Statistics For Joint Tests			
Source	DF	Chi-Square	Pr > ChiSq
age_cat	2	5.69	0.0582
inc	3	9.14	0.0275
edu	2	3.21	0.2008
marital_status	3	7.67	0.0533
kids	2	2.32	0.3132
hadajob	1	0.64	0.4241

Note: Under full-rank parameterizations, Type 3 effect tests are replaced by joint tests. The joint test for an effect is a test that all the parameters associated with that effect are zero. Such joint tests might not be equivalent to Type 3 effect tests under GLM parameterization.

Fully adjusted models of Depression score with gender*theme interaction

The GENMOD Procedure

Model Information		
**Data Set**	WORK.Z	
**Distribution**	Negative Binomial	
**Link Function**	Log	
**Dependent Variable**	DscoreR	HADS Depression score

**Number of Observations Read**	1293
**Number of Observations Used**	1293

Class Level Information				
**Class**	**Value**	**Design Variables**		
**gender**	**Female**	0	0	
	**Male**	1	0	
	**Other**	0	1	
**race**	**black**	1	0	
	**other race**	0	1	
	**white**	0	0	
**age_cat**	**35-54**	1	0	
	**greater than 55**	0	1	
	**less than 35**	0	0	
**inc**	**$100,000 and above**	0	0	0
	**40,000 - $99,999**	1	0	0
	**less than $40,000**	0	1	0
	**missing**	0	0	1
**edu**	**college degree or higher**	0	0	
	**missing**	1	0	
	**no college degree**	0	1	

Fully adjusted models of Depression score with gender*theme interaction

The GENMOD Procedure

Class Level Information				
Class	Value	Design Variables		
kids	missing	1	0	
	no	0	0	
	yes	0	1	
marital_status	married or living as married	1	0	0
	missing	0	1	0
	single	0	0	0
	widowed, divorced	0	0	1
feltunwell	missing	1	0	
	no	0	0	
	yes	0	1	
compare_others	good to excellent	0		
	poor or fair	1		
ratehealth	good to excellent	0		
	poor or fair	1		
x1	no	0		
	yes	1		
x2	no	0		
	yes	1		
x3	no	0		
	yes	1		
x4	no	0		
	yes	1		
x5	no	0		
	yes	1		

The GENMOD Procedure

Class Level Information				
Class	Value	Design Variables		
x6	no	0		
	yes	1		
x8	no	0		
	yes	1		
x9	no	0		
	yes	1		
x10	no	0		
	yes	1		
x11	no	0		
	yes	1		
x12	no	0		
	yes	1		
x13	no	0		
	yes	1		
x14	no	0		
	yes	1		
upset	do not want to answer	1	0	0
	missing	0	0	0
	no	0	1	0
	yes	0	0	1
hadajob	no	1		
	yes	0		
infected	no	0		
	yes	1		

Fully adjusted models of Depression score with gender*theme interaction

The GENMOD Procedure

Criteria For Assessing Goodness Of Fit			
**Criterion**	**DF**	**Value**	**Value/DF**
**Deviance**	1243	1460.6449	1.1751
**Scaled Deviance**	1243	1460.6449	1.1751
**Pearson Chi-Square**	1243	1287.8469	1.0361
**Scaled Pearson X2**	1243	1287.8469	1.0361
**Log Likelihood**		7064.6621	
**Full Log Likelihood**		-3381.9204	
**AIC (smaller is better)**		6865.8408	
**AICC (smaller is better)**		6870.1147	
**BIC (smaller is better)**		7129.2415	

Algorithm converged.

Analysis Of Maximum Likelihood Parameter Estimates									
**Parameter**			**DF**	**Estimate**	**Standard Error**	**Wald 95% Confidence Limits**		**Wald Chi-Square**	**Pr > ChiSq**
**Intercept**			1	1.9301	0.1121	1.7105	2.1498	296.57	<.0001
**gender*x9**	Male	yes	1	0.3914	0.4513	-0.4932	1.2760	0.75	0.3858
**gender*x9**	Other	yes	0	0.0000	0.0000	0.0000	0.0000	.	.
**x1**	yes		1	0.0018	0.0442	-0.0848	0.0884	0.00	0.9677
**x2**	yes		1	0.3605	0.2120	-0.0550	0.7760	2.89	0.0890
**x3**	yes		1	0.0722	0.0742	-0.0731	0.2176	0.95	0.3299
**x4**	yes		1	0.2090	0.0614	0.0886	0.3294	11.58	0.0007
**x5**	yes		1	0.0721	0.0547	-0.0351	0.1793	1.74	0.1872
**x6**	yes		1	0.0715	0.0562	-0.0386	0.1815	1.62	0.2032
**x8**	yes		1	0.1062	0.0534	0.0015	0.2109	3.95	0.0469
**x9**	yes		1	-0.0171	0.2183	-0.4449	0.4107	0.01	0.9376

The GENMOD Procedure

Analysis Of Maximum Likelihood Parameter Estimates									
Parameter			DF	Estimate	Standard Error	Wald 95% Confidence Limits		Wald Chi-Square	Pr > ChiSq
**x10**	yes		1	0.1283	0.0593	0.0121	0.2446	4.68	0.0305
**x11**	yes		1	-0.0234	0.1540	-0.3252	0.2784	0.02	0.8794
**x12**	yes		1	0.0178	0.1991	-0.3724	0.4080	0.01	0.9289
**x13**	yes		1	0.1435	0.1034	-0.0592	0.3462	1.92	0.1654
**x14**	yes		1	0.1675	0.1823	-0.1898	0.5248	0.84	0.3582
**upset**	do not want to answer		1	0.0128	0.2102	-0.3992	0.4249	0.00	0.9514
**upset**	no		1	-0.0581	0.0418	-0.1400	0.0237	1.94	0.1639
**upset**	yes		1	0.1337	0.0645	0.0072	0.2601	4.29	0.0383
**dur**			1	0.0026	0.0006	0.0014	0.0038	17.50	<.0001
**support_family**			1	-0.0033	0.0007	-0.0046	-0.0020	24.61	<.0001
**support_doctor**			1	-0.0011	0.0007	-0.0024	0.0002	2.52	0.1121
**support_fedgovt**			1	0.0002	0.0009	-0.0016	0.0019	0.03	0.8549
**support_city**			1	0.0023	0.0010	0.0003	0.0042	5.23	0.0222
**support_deptpubhealt**			1	-0.0017	0.0009	-0.0035	0.0001	3.28	0.0703
**support_faithcommuni**			1	-0.0020	0.0007	-0.0034	-0.0007	8.48	0.0036
**support_socialservic**			1	0.0018	0.0009	-0.0000	0.0036	3.74	0.0532
**support_neighbors**			1	-0.0013	0.0007	-0.0027	0.0000	3.67	0.0555
**support_other**			1	-0.0014	0.0008	-0.0031	0.0002	2.88	0.0898
**infected**	yes		1	0.0463	0.1603	-0.2679	0.3606	0.08	0.7726
**feltunwell**	missing		1	0.1566	0.2943	-0.4202	0.7334	0.28	0.5946
**feltunwell**	yes		1	0.0954	0.0390	0.0190	0.1718	6.00	0.0143
**compare_others**	poor or fair		1	0.2437	0.0612	0.1237	0.3637	15.84	<.0001
**ratehealth**	poor or fair		1	-0.0627	0.0822	-0.2238	0.0984	0.58	0.4454
**gender**	Male		1	-0.1424	0.0394	-0.2197	-0.0651	13.04	0.0003
**gender**	Other		1	0.0634	0.1362	-0.2035	0.3304	0.22	0.6413

Fully adjusted models of Depression score with gender*theme interaction

The GENMOD Procedure

Analysis Of Maximum Likelihood Parameter Estimates									
Parameter			DF	Estimate	Standard Error	Wald 95% Confidence Limits		Wald Chi-Square	Pr > ChiSq
race	black		1	-0.0769	0.0684	-0.2110	0.0572	1.26	0.2609
race	other race		1	0.0220	0.0641	-0.1036	0.1476	0.12	0.7312
age_cat	35-54		1	0.0228	0.0513	-0.0777	0.1233	0.20	0.6564
age_cat	greater than 55		1	-0.0808	0.0552	-0.1891	0.0274	2.14	0.1433
inc	40,000 - $99,999		1	0.0580	0.0414	-0.0232	0.1392	1.96	0.1614
inc	less than $40,000		1	0.1569	0.0525	0.0540	0.2598	8.93	0.0028
inc	missing		1	0.1096	0.1072	-0.1005	0.3198	1.05	0.3066
edu	missing		1	-0.4676	0.2706	-0.9979	0.0627	2.99	0.0840
edu	no college degree		1	0.0092	0.0485	-0.0859	0.1044	0.04	0.8495
marital_status	married or living as married		1	0.0847	0.0428	0.0009	0.1686	3.93	0.0476
marital_status	missing		1	0.4291	0.2124	0.0129	0.8453	4.08	0.0433
marital_status	widowed, divorced		1	0.0119	0.0604	-0.1065	0.1302	0.04	0.8443
kids	missing		1	-0.3950	0.2586	-0.9018	0.1118	2.33	0.1266
kids	yes		1	-0.0091	0.0478	-0.1027	0.0845	0.04	0.8489
hadajob	no		1	0.0345	0.0439	-0.0516	0.1207	0.62	0.4317
Dispersion			1	0.1684	0.0141	0.1429	0.1984		

Note: The negative binomial dispersion parameter was estimated by maximum likelihood.

LR Statistics For Joint Tests			
Source	DF	Chi-Square	Pr > ChiSq
gender*x9	2	2.57	0.2766
x1	1	0.00	0.9677
x2	1	2.92	0.0875
x3	1	0.95	0.3299
x4	1	11.56	0.0007

The GENMOD Procedure

LR Statistics For Joint Tests			
**Source**	**DF**	**Chi-Square**	**Pr > ChiSq**
**x5**	1	1.74	0.1873
**x6**	1	1.62	0.2032
**x8**	1	3.95	0.0470
**x9**	1	0.01	0.9376
**x10**	1	4.68	0.0305
**x11**	1	0.02	0.8794
**x12**	1	0.01	0.9289
**x13**	1	1.93	0.1649
**x14**	1	0.85	0.3576
**upset**	3	12.48	0.0059
**dur**	1	17.38	<.0001
**support_family**	1	24.45	<.0001
**support_doctor**	1	2.52	0.1122
**support_fedgovt**	1	0.03	0.8549
**support_city**	1	5.23	0.0222
**support_deptpubhealt**	1	3.27	0.0704
**support_faithcommuni**	1	8.46	0.0036
**support_socialservic**	1	3.74	0.0532
**support_neighbors**	1	3.66	0.0556
**support_other**	1	2.88	0.0899
**infected**	1	0.08	0.7725
**feltunwell**	2	6.18	0.0455
**compare_others**	1	15.83	<.0001
**ratehealth**	1	0.58	0.4456
**gender**	2	13.48	0.0012
**race**	2	1.46	0.4824

The GENMOD Procedure

LR Statistics For Joint Tests			
Source	DF	Chi-Square	Pr > ChiSq
age_cat	2	5.90	0.0524
inc	3	9.29	0.0257
edu	2	3.15	0.2072
marital_status	3	7.81	0.0500
kids	2	2.38	0.3038
hadajob	1	0.62	0.4318

Note: Under full-rank parameterizations, Type 3 effect tests are replaced by joint tests. The joint test for an effect is a test that all the parameters associated with that effect are zero. Such joint tests might not be equivalent to Type 3 effect tests under GLM parameterization.

Fully adjusted models of Depression score with gender*theme interaction

The GENMOD Procedure

Model Information		
**Data Set**	WORK.Z	
**Distribution**	Negative Binomial	
**Link Function**	Log	
**Dependent Variable**	DscoreR	HADS Depression score

**Number of Observations Read**	1293
**Number of Observations Used**	1293

Class Level Information				
**Class**	**Value**	**Design Variables**		
**gender**	**Female**	0	0	
	**Male**	1	0	
	**Other**	0	1	
**race**	**black**	1	0	
	**other race**	0	1	
	**white**	0	0	
**age_cat**	**35-54**	1	0	
	**greater than 55**	0	1	
	**less than 35**	0	0	
**inc**	**$100,000 and above**	0	0	0
	**40,000 - $99,999**	1	0	0
	**less than $40,000**	0	1	0
	**missing**	0	0	1
**edu**	**college degree or higher**	0	0	
	**missing**	1	0	
	**no college degree**	0	1	

The GENMOD Procedure

Class Level Information				
Class	Value	Design Variables		
kids	missing	1	0	
	no	0	0	
	yes	0	1	
marital_status	married or living as married	1	0	0
	missing	0	1	0
	single	0	0	0
	widowed, divorced	0	0	1
feltunwell	missing	1	0	
	no	0	0	
	yes	0	1	
compare_others	good to excellent	0		
	poor or fair	1		
ratehealth	good to excellent	0		
	poor or fair	1		
x1	no	0		
	yes	1		
x2	no	0		
	yes	1		
x3	no	0		
	yes	1		
x4	no	0		
	yes	1		
x5	no	0		
	yes	1		

The GENMOD Procedure

Class Level Information				
**Class**	**Value**	**Design Variables**		
**x6**	**no**	0		
	**yes**	1		
**x8**	**no**	0		
	**yes**	1		
**x9**	**no**	0		
	**yes**	1		
**x10**	**no**	0		
	**yes**	1		
**x11**	**no**	0		
	**yes**	1		
**x12**	**no**	0		
	**yes**	1		
**x13**	**no**	0		
	**yes**	1		
**x14**	**no**	0		
	**yes**	1		
**upset**	**do not want to answer**	1	0	0
	**missing**	0	0	0
	**no**	0	1	0
	**yes**	0	0	1
**hadajob**	**no**	1		
	**yes**	0		
**infected**	**no**	0		
	**yes**	1		

Fully adjusted models of Depression score with gender*theme interaction

The GENMOD Procedure

Criteria For Assessing Goodness Of Fit			
**Criterion**	**DF**	**Value**	**Value/DF**
**Deviance**	1242	1460.6056	1.1760
**Scaled Deviance**	1242	1460.6056	1.1760
**Pearson Chi-Square**	1242	1289.2888	1.0381
**Scaled Pearson X2**	1242	1289.2888	1.0381
**Log Likelihood**		7066.1966	
**Full Log Likelihood**		-3380.3858	
**AIC (smaller is better)**		6864.7717	
**AICC (smaller is better)**		6869.2169	
**BIC (smaller is better)**		7133.3372	

Algorithm converged.

Analysis Of Maximum Likelihood Parameter Estimates									
**Parameter**			**DF**	**Estimate**	**Standard Error**	**Wald 95% Confidence Limits**		**Wald Chi-Square**	**Pr > ChiSq**
**Intercept**			1	1.9399	0.1120	1.7205	2.1594	300.19	<.0001
**gender*x10**	Male	yes	1	0.1457	0.1325	-0.1140	0.4055	1.21	0.2714
**gender*x10**	Other	yes	1	-0.7571	0.5050	-1.7469	0.2327	2.25	0.1338
**x1**	yes		1	0.0033	0.0441	-0.0832	0.0898	0.01	0.9409
**x2**	yes		1	0.3660	0.2118	-0.0491	0.7811	2.99	0.0840
**x3**	yes		1	0.0717	0.0741	-0.0735	0.2168	0.94	0.3330
**x4**	yes		1	0.2105	0.0614	0.0902	0.3308	11.76	0.0006
**x5**	yes		1	0.0727	0.0547	-0.0345	0.1800	1.77	0.1837
**x6**	yes		1	0.0704	0.0561	-0.0396	0.1803	1.57	0.2098
**x8**	yes		1	0.1090	0.0534	0.0043	0.2136	4.16	0.0413
**x9**	yes		1	0.0630	0.1912	-0.3118	0.4378	0.11	0.7419

The GENMOD Procedure

Analysis Of Maximum Likelihood Parameter Estimates									
Parameter			DF	Estimate	Standard Error	Wald 95% Confidence Limits		Wald Chi-Square	Pr > ChiSq
x10	yes		1	0.1045	0.0691	-0.0310	0.2400	2.29	0.1306
x11	yes		1	-0.0197	0.1538	-0.3212	0.2818	0.02	0.8983
x12	yes		1	0.0136	0.1992	-0.3768	0.4040	0.00	0.9456
x13	yes		1	0.1432	0.1033	-0.0593	0.3456	1.92	0.1658
x14	yes		1	0.1644	0.1821	-0.1925	0.5213	0.81	0.3667
upset	do not want to answer		1	-0.0031	0.2107	-0.4160	0.4098	0.00	0.9883
upset	no		1	-0.0611	0.0418	-0.1430	0.0207	2.14	0.1432
upset	yes		1	0.1286	0.0645	0.0023	0.2550	3.98	0.0460
dur			1	0.0026	0.0006	0.0014	0.0038	17.33	<.0001
support_family			1	-0.0032	0.0007	-0.0045	-0.0020	24.36	<.0001
support_doctor			1	-0.0010	0.0007	-0.0023	0.0003	2.39	0.1221
support_fedgovt			1	0.0002	0.0009	-0.0016	0.0019	0.04	0.8354
support_city			1	0.0023	0.0010	0.0003	0.0042	5.25	0.0220
support_deptpubhealt			1	-0.0017	0.0009	-0.0035	0.0002	3.19	0.0743
support_faithcommuni			1	-0.0021	0.0007	-0.0034	-0.0007	8.89	0.0029
support_socialservic			1	0.0018	0.0009	-0.0001	0.0036	3.55	0.0597
support_neighbors			1	-0.0013	0.0007	-0.0027	0.0001	3.39	0.0657
support_other			1	-0.0015	0.0008	-0.0032	0.0001	3.31	0.0690
infected	yes		1	0.0476	0.1601	-0.2662	0.3614	0.09	0.7663
feltunwell	missing		1	0.1599	0.2939	-0.4162	0.7360	0.30	0.5865
feltunwell	yes		1	0.0932	0.0389	0.0169	0.1695	5.74	0.0166
compare_others	poor or fair		1	0.2419	0.0612	0.1220	0.3618	15.64	<.0001
ratehealth	poor or fair		1	-0.0619	0.0821	-0.2228	0.0991	0.57	0.4511
gender	Male		1	-0.1529	0.0411	-0.2334	-0.0724	13.86	0.0002
gender	Other		1	0.1215	0.1420	-0.1567	0.3998	0.73	0.3920

Fully adjusted models of Depression score with gender*theme interaction

The GENMOD Procedure

Analysis Of Maximum Likelihood Parameter Estimates									
Parameter			DF	Estimate	Standard Error	Wald 95% Confidence Limits		Wald Chi-Square	Pr > ChiSq
race	black		1	-0.0749	0.0684	-0.2090	0.0591	1.20	0.2732
race	other race		1	0.0234	0.0640	-0.1021	0.1490	0.13	0.7144
age_cat	35-54		1	0.0185	0.0512	-0.0818	0.1187	0.13	0.7180
age_cat	greater than 55		1	-0.0812	0.0552	-0.1893	0.0269	2.17	0.1411
inc	40,000 - $99,999		1	0.0580	0.0414	-0.0232	0.1391	1.96	0.1616
inc	less than $40,000		1	0.1610	0.0525	0.0581	0.2638	9.40	0.0022
inc	missing		1	0.1105	0.1071	-0.0994	0.3204	1.06	0.3022
edu	missing		1	-0.4823	0.2706	-1.0126	0.0480	3.18	0.0747
edu	no college degree		1	0.0073	0.0485	-0.0878	0.1024	0.02	0.8800
marital_status	married or living as married		1	0.0836	0.0428	-0.0002	0.1674	3.82	0.0505
marital_status	missing		1	0.4320	0.2121	0.0163	0.8477	4.15	0.0417
marital_status	widowed, divorced		1	0.0093	0.0603	-0.1090	0.1276	0.02	0.8775
kids	missing		1	-0.3875	0.2584	-0.8939	0.1189	2.25	0.1336
kids	yes		1	-0.0065	0.0476	-0.0997	0.0868	0.02	0.8920
hadajob	no		1	0.0321	0.0439	-0.0539	0.1182	0.54	0.4640
Dispersion			1	0.1676	0.0141	0.1422	0.1975		

Note: The negative binomial dispersion parameter was estimated by maximum likelihood.

LR Statistics For Joint Tests			
Source	DF	Chi-Square	Pr > ChiSq
gender*x10	2	3.82	0.1477
x1	1	0.01	0.9409
x2	1	3.01	0.0825
x3	1	0.94	0.3330
x4	1	11.74	0.0006

The GENMOD Procedure

LR Statistics For Joint Tests			
Source	DF	Chi-Square	Pr > ChiSq
x5	1	1.77	0.1837
x6	1	1.57	0.2098
x8	1	4.16	0.0414
x9	1	0.11	0.7417
x10	1	2.29	0.1305
x11	1	0.02	0.8983
x12	1	0.00	0.9456
x13	1	1.92	0.1653
x14	1	0.82	0.3662
upset	3	12.36	0.0062
dur	1	17.22	<.0001
support_family	1	24.21	<.0001
support_doctor	1	2.39	0.1222
support_fedgovt	1	0.04	0.8354
support_city	1	5.24	0.0220
support_deptpubhealt	1	3.18	0.0743
support_faithcommuni	1	8.87	0.0029
support_socialservic	1	3.55	0.0597
support_neighbors	1	3.38	0.0658
support_other	1	3.31	0.0691
infected	1	0.09	0.7662
feltunwell	2	5.94	0.0514
compare_others	1	15.63	<.0001
ratehealth	1	0.57	0.4512
gender	2	15.04	0.0005
race	2	1.41	0.4929

The GENMOD Procedure

LR Statistics For Joint Tests			
**Source**	**DF**	**Chi-Square**	**Pr > ChiSq**
**age_cat**	2	5.53	0.0629
**inc**	3	9.81	0.0203
**edu**	2	3.33	0.1889
**marital_status**	3	7.84	0.0495
**kids**	2	2.29	0.3183
**hadajob**	1	0.54	0.4641

Note: Under full-rank parameterizations, Type 3 effect tests are replaced by joint tests. The joint test for an effect is a test that all the parameters associated with that effect are zero. Such joint tests might not be equivalent to Type 3 effect tests under GLM parameterization.

The GENMOD Procedure

Model Information		
**Data Set**	WORK.Z	
**Distribution**	Negative Binomial	
**Link Function**	Log	
**Dependent Variable**	DscoreR	HADS Depression score

**Number of Observations Read**	1293
**Number of Observations Used**	1293

Class Level Information				
**Class**	**Value**	**Design Variables**		
**gender**	**Female**	0	0	
	**Male**	1	0	
	**Other**	0	1	
**race**	**black**	1	0	
	**other race**	0	1	
	**white**	0	0	
**age_cat**	**35-54**	1	0	
	**greater than 55**	0	1	
	**less than 35**	0	0	
**inc**	**$100,000 and above**	0	0	0
	**40,000 - $99,999**	1	0	0
	**less than $40,000**	0	1	0
	**missing**	0	0	1
**edu**	**college degree or higher**	0	0	
	**missing**	1	0	
	**no college degree**	0	1	

Fully adjusted models of Depression score with gender*theme interaction

The GENMOD Procedure

Class Level Information				
Class	Value	Design Variables		
kids	missing	1	0	
	no	0	0	
	yes	0	1	
marital_status	married or living as married	1	0	0
	missing	0	1	0
	single	0	0	0
	widowed, divorced	0	0	1
feltunwell	missing	1	0	
	no	0	0	
	yes	0	1	
compare_others	good to excellent	0		
	poor or fair	1		
ratehealth	good to excellent	0		
	poor or fair	1		
x1	no	0		
	yes	1		
x2	no	0		
	yes	1		
x3	no	0		
	yes	1		
x4	no	0		
	yes	1		
x5	no	0		
	yes	1		

The GENMOD Procedure

Class Level Information				
Class	Value	Design Variables		
x6	no	0		
	yes	1		
x8	no	0		
	yes	1		
x9	no	0		
	yes	1		
x10	no	0		
	yes	1		
x11	no	0		
	yes	1		
x12	no	0		
	yes	1		
x13	no	0		
	yes	1		
x14	no	0		
	yes	1		
upset	do not want to answer	1	0	0
	missing	0	0	0
	no	0	1	0
	yes	0	0	1
hadajob	no	1		
	yes	0		
infected	no	0		
	yes	1		

The GENMOD Procedure

Criteria For Assessing Goodness Of Fit			
Criterion	DF	Value	Value/DF
Deviance	1243	1460.3445	1.1749
Scaled Deviance	1243	1460.3445	1.1749
Pearson Chi-Square	1243	1286.2168	1.0348
Scaled Pearson X2	1243	1286.2168	1.0348
Log Likelihood		7064.2890	
Full Log Likelihood		-3382.2934	
AIC (smaller is better)		6866.5869	
AICC (smaller is better)		6870.8608	
BIC (smaller is better)		7129.9876	

Algorithm converged.

Analysis Of Maximum Likelihood Parameter Estimates									
Parameter			DF	Estimate	Standard Error	Wald 95% Confidence Limits		Wald Chi-Square	Pr > ChiSq
Intercept			1	1.9354	0.1120	1.7159	2.1548	298.79	<.0001
gender*x11	Male	yes	1	-0.0332	0.3381	-0.6958	0.6294	0.01	0.9218
gender*x11	Other	yes	0	0.0000	0.0000	0.0000	0.0000	.	.
x1	yes		1	0.0031	0.0442	-0.0835	0.0897	0.00	0.9443
x2	yes		1	0.3608	0.2121	-0.0549	0.7765	2.89	0.0889
x3	yes		1	0.0701	0.0742	-0.0754	0.2156	0.89	0.3450
x4	yes		1	0.2089	0.0615	0.0884	0.3295	11.54	0.0007
x5	yes		1	0.0707	0.0547	-0.0365	0.1779	1.67	0.1961
x6	yes		1	0.0711	0.0562	-0.0391	0.1812	1.60	0.2059
x8	yes		1	0.1055	0.0535	0.0007	0.2103	3.89	0.0486
x9	yes		1	0.0754	0.1914	-0.2997	0.4505	0.16	0.6936

Fully adjusted models of Depression score with gender*theme interaction

The GENMOD Procedure

Analysis Of Maximum Likelihood Parameter Estimates									
Parameter			DF	Estimate	Standard Error	Wald 95% Confidence Limits		Wald Chi-Square	Pr > ChiSq
**x10**	yes		1	0.1314	0.0592	0.0153	0.2475	4.92	0.0265
**x11**	yes		1	-0.0132	0.1834	-0.3728	0.3463	0.01	0.9424
**x12**	yes		1	0.0175	0.1992	-0.3728	0.4079	0.01	0.9299
**x13**	yes		1	0.1404	0.1034	-0.0623	0.3431	1.84	0.1747
**x14**	yes		1	0.1682	0.1824	-0.1893	0.5257	0.85	0.3565
**upset**	do not want to answer		1	0.0133	0.2103	-0.3989	0.4255	0.00	0.9495
**upset**	no		1	-0.0583	0.0418	-0.1402	0.0236	1.95	0.1626
**upset**	yes		1	0.1336	0.0646	0.0069	0.2602	4.27	0.0387
**dur**			1	0.0026	0.0006	0.0014	0.0038	17.14	<.0001
**support_family**			1	-0.0033	0.0007	-0.0046	-0.0020	24.88	<.0001
**support_doctor**			1	-0.0011	0.0007	-0.0024	0.0002	2.54	0.1107
**support_fedgovt**			1	0.0002	0.0009	-0.0016	0.0019	0.04	0.8455
**support_city**			1	0.0023	0.0010	0.0003	0.0042	5.23	0.0221
**support_deptpubhealt**			1	-0.0017	0.0009	-0.0035	0.0001	3.24	0.0717
**support_faithcommuni**			1	-0.0020	0.0007	-0.0034	-0.0007	8.47	0.0036
**support_socialservic**			1	0.0018	0.0009	-0.0001	0.0036	3.60	0.0579
**support_neighbors**			1	-0.0013	0.0007	-0.0027	0.0001	3.55	0.0594
**support_other**			1	-0.0014	0.0008	-0.0031	0.0002	2.94	0.0866
**infected**	yes		1	0.0498	0.1626	-0.2690	0.3685	0.09	0.7595
**feltunwell**	missing		1	0.1580	0.2944	-0.4190	0.7351	0.29	0.5914
**feltunwell**	yes		1	0.0949	0.0390	0.0185	0.1712	5.92	0.0150
**compare_others**	poor or fair		1	0.2427	0.0613	0.1226	0.3627	15.70	<.0001
**ratehealth**	poor or fair		1	-0.0612	0.0822	-0.2223	0.0999	0.55	0.4566
**gender**	Male		1	-0.1392	0.0395	-0.2167	-0.0618	12.41	0.0004
**gender**	Other		1	0.0636	0.1363	-0.2035	0.3307	0.22	0.6408

Fully adjusted models of Depression score with gender*theme interaction

The GENMOD Procedure

Analysis Of Maximum Likelihood Parameter Estimates									
Parameter			DF	Estimate	Standard Error	Wald 95% Confidence Limits		Wald Chi-Square	Pr > ChiSq
race	black		1	-0.0776	0.0684	-0.2117	0.0566	1.28	0.2570
race	other race		1	0.0217	0.0641	-0.1039	0.1474	0.11	0.7345
age_cat	35-54		1	0.0203	0.0512	-0.0801	0.1206	0.16	0.6922
age_cat	greater than 55		1	-0.0812	0.0553	-0.1895	0.0271	2.16	0.1416
inc	40,000 - $99,999		1	0.0573	0.0415	-0.0240	0.1387	1.91	0.1672
inc	less than $40,000		1	0.1567	0.0526	0.0536	0.2599	8.88	0.0029
inc	missing		1	0.1096	0.1073	-0.1007	0.3198	1.04	0.3070
edu	missing		1	-0.4680	0.2706	-0.9984	0.0625	2.99	0.0838
edu	no college degree		1	0.0097	0.0486	-0.0855	0.1049	0.04	0.8416
marital_status	married or living as married		1	0.0841	0.0428	0.0002	0.1680	3.86	0.0495
marital_status	missing		1	0.4283	0.2126	0.0117	0.8449	4.06	0.0439
marital_status	widowed, divorced		1	0.0114	0.0604	-0.1071	0.1298	0.04	0.8509
kids	missing		1	-0.3951	0.2587	-0.9021	0.1119	2.33	0.1266
kids	yes		1	-0.0059	0.0477	-0.0993	0.0875	0.02	0.9022
hadajob	no		1	0.0335	0.0439	-0.0527	0.1196	0.58	0.4464
Dispersion			1	0.1687	0.0141	0.1432	0.1987		

Note: The negative binomial dispersion parameter was estimated by maximum likelihood.

LR Statistics For Joint Tests			
Source	DF	Chi-Square	Pr > ChiSq
gender*x11	2	1.82	0.4016
x1	1	0.00	0.9443
x2	1	2.92	0.0874
x3	1	0.89	0.3450
x4	1	11.53	0.0007

The GENMOD Procedure

LR Statistics For Joint Tests			
Source	DF	Chi-Square	Pr > ChiSq
x5	1	1.67	0.1962
x6	1	1.60	0.2059
x8	1	3.89	0.0487
x9	1	0.16	0.6933
x10	1	4.92	0.0265
x11	1	0.01	0.9424
x12	1	0.01	0.9299
x13	1	1.85	0.1742
x14	1	0.85	0.3560
upset	3	12.47	0.0059
dur	1	17.03	<.0001
support_family	1	24.73	<.0001
support_doctor	1	2.54	0.1108
support_fedgovt	1	0.04	0.8455
support_city	1	5.23	0.0222
support_deptpubhealt	1	3.24	0.0718
support_faithcommuni	1	8.44	0.0037
support_socialservic	1	3.60	0.0580
support_neighbors	1	3.55	0.0595
support_other	1	2.93	0.0867
infected	1	0.09	0.7594
feltunwell	2	6.11	0.0471
compare_others	1	15.69	<.0001
ratehealth	1	0.55	0.4567
gender	2	12.86	0.0016
race	2	1.48	0.4781

The GENMOD Procedure

LR Statistics For Joint Tests			
**Source**	**DF**	**Chi-Square**	**Pr > ChiSq**
**age_cat**	2	5.70	0.0580
**inc**	3	9.26	0.0260
**edu**	2	3.16	0.2063
**marital_status**	3	7.73	0.0519
**kids**	2	2.37	0.3053
**hadajob**	1	0.58	0.4465

Note: Under full-rank parameterizations, Type 3 effect tests are replaced by joint tests. The joint test for an effect is a test that all the parameters associated with that effect are zero. Such joint tests might not be equivalent to Type 3 effect tests under GLM parameterization.

The GENMOD Procedure

Model Information		
**Data Set**	WORK.Z	
**Distribution**	Negative Binomial	
**Link Function**	Log	
**Dependent Variable**	DscoreR	HADS Depression score

**Number of Observations Read**	1293
**Number of Observations Used**	1293

Class Level Information				
**Class**	**Value**	**Design Variables**		
**gender**	**Female**	0	0	
	**Male**	1	0	
	**Other**	0	1	
**race**	**black**	1	0	
	**other race**	0	1	
	**white**	0	0	
**age_cat**	**35-54**	1	0	
	**greater than 55**	0	1	
	**less than 35**	0	0	
**inc**	**$100,000 and above**	0	0	0
	**40,000 - $99,999**	1	0	0
	**less than $40,000**	0	1	0
	**missing**	0	0	1
**edu**	**college degree or higher**	0	0	
	**missing**	1	0	
	**no college degree**	0	1	

Fully adjusted models of Depression score with gender*theme interaction

The GENMOD Procedure

Class Level Information				
Class	Value	Design Variables		
kids	missing	1	0	
	no	0	0	
	yes	0	1	
marital_status	married or living as married	1	0	0
	missing	0	1	0
	single	0	0	0
	widowed, divorced	0	0	1
feltunwell	missing	1	0	
	no	0	0	
	yes	0	1	
compare_others	good to excellent	0		
	poor or fair	1		
ratehealth	good to excellent	0		
	poor or fair	1		
x1	no	0		
	yes	1		
x2	no	0		
	yes	1		
x3	no	0		
	yes	1		
x4	no	0		
	yes	1		
x5	no	0		
	yes	1		

The GENMOD Procedure

Class Level Information				
Class	Value	Design Variables		
x6	no	0		
	yes	1		
x8	no	0		
	yes	1		
x9	no	0		
	yes	1		
x10	no	0		
	yes	1		
x11	no	0		
	yes	1		
x12	no	0		
	yes	1		
x13	no	0		
	yes	1		
x14	no	0		
	yes	1		
upset	do not want to answer	1	0	0
	missing	0	0	0
	no	0	1	0
	yes	0	0	1
hadajob	no	1		
	yes	0		
infected	no	0		
	yes	1		

Fully adjusted models of Depression score with gender*theme interaction

The GENMOD Procedure

Criteria For Assessing Goodness Of Fit			
**Criterion**	**DF**	**Value**	**Value/DF**
**Deviance**	1242	1460.5065	1.1759
**Scaled Deviance**	1242	1460.5065	1.1759
**Pearson Chi-Square**	1242	1287.8760	1.0369
**Scaled Pearson X2**	1242	1287.8760	1.0369
**Log Likelihood**		7064.6611	
**Full Log Likelihood**		-3381.9214	
**AIC (smaller is better)**		6867.8427	
**AICC (smaller is better)**		6872.2879	
**BIC (smaller is better)**		7136.4082	

Algorithm converged.

Analysis Of Maximum Likelihood Parameter Estimates									
**Parameter**			**DF**	**Estimate**	**Standard Error**	**Wald 95% Confidence Limits**		**Wald Chi-Square**	**Pr > ChiSq**
**Intercept**			1	1.9278	0.1123	1.7077	2.1479	294.68	<.0001
**gender*x12**	Male	yes	1	0.2913	0.4909	-0.6708	1.2534	0.35	0.5529
**gender*x12**	Other	yes	1	0.4448	0.6015	-0.7341	1.6236	0.55	0.4596
**x1**	yes		1	0.0032	0.0442	-0.0834	0.0898	0.01	0.9422
**x2**	yes		1	0.3620	0.2120	-0.0536	0.7775	2.91	0.0878
**x3**	yes		1	0.0713	0.0741	-0.0740	0.2166	0.92	0.3363
**x4**	yes		1	0.2088	0.0614	0.0884	0.3292	11.55	0.0007
**x5**	yes		1	0.0730	0.0547	-0.0343	0.1803	1.78	0.1822
**x6**	yes		1	0.0729	0.0562	-0.0372	0.1831	1.68	0.1943
**x8**	yes		1	0.1063	0.0534	0.0016	0.2110	3.96	0.0466
**x9**	yes		1	0.0759	0.1913	-0.2991	0.4509	0.16	0.6917

The GENMOD Procedure

Analysis Of Maximum Likelihood Parameter Estimates									
Parameter			DF	Estimate	Standard Error	Wald 95% Confidence Limits		Wald Chi-Square	Pr > ChiSq
x10	yes		1	0.1339	0.0593	0.0177	0.2500	5.10	0.0239
x11	yes		1	-0.0220	0.1540	-0.3238	0.2798	0.02	0.8864
x12	yes		1	-0.1074	0.2482	-0.5939	0.3792	0.19	0.6654
x13	yes		1	0.1409	0.1034	-0.0617	0.3435	1.86	0.1730
x14	yes		1	0.1682	0.1823	-0.1892	0.5255	0.85	0.3564
upset	do not want to answer		1	0.0181	0.2103	-0.3941	0.4303	0.01	0.9314
upset	no		1	-0.0595	0.0418	-0.1414	0.0224	2.03	0.1547
upset	yes		1	0.1347	0.0645	0.0082	0.2611	4.35	0.0369
dur			1	0.0026	0.0006	0.0014	0.0038	17.41	<.0001
support_family			1	-0.0033	0.0007	-0.0046	-0.0020	24.56	<.0001
support_doctor			1	-0.0011	0.0007	-0.0024	0.0002	2.52	0.1121
support_fedgovt			1	0.0002	0.0009	-0.0016	0.0019	0.04	0.8383
support_city			1	0.0023	0.0010	0.0004	0.0043	5.40	0.0202
support_deptpubhealt			1	-0.0017	0.0009	-0.0035	0.0002	3.23	0.0722
support_faithcommuni			1	-0.0020	0.0007	-0.0034	-0.0007	8.56	0.0034
support_socialservic			1	0.0017	0.0009	-0.0001	0.0035	3.30	0.0694
support_neighbors			1	-0.0013	0.0007	-0.0027	0.0000	3.63	0.0567
support_other			1	-0.0014	0.0008	-0.0030	0.0003	2.65	0.1036
infected	yes		1	0.0461	0.1604	-0.2683	0.3604	0.08	0.7739
feltunwell	missing		1	0.1571	0.2943	-0.4197	0.7340	0.28	0.5935
feltunwell	yes		1	0.0949	0.0390	0.0185	0.1713	5.93	0.0149
compare_others	poor or fair		1	0.2441	0.0612	0.1240	0.3641	15.88	<.0001
ratehealth	poor or fair		1	-0.0620	0.0822	-0.2230	0.0991	0.57	0.4508
gender	Male		1	-0.1409	0.0394	-0.2181	-0.0637	12.79	0.0003
gender	Other		1	0.0432	0.1396	-0.2304	0.3169	0.10	0.7568

Fully adjusted models of Depression score with gender*theme interaction

The GENMOD Procedure

Analysis Of Maximum Likelihood Parameter Estimates									
Parameter			DF	Estimate	Standard Error	Wald 95% Confidence Limits		Wald Chi-Square	Pr > ChiSq
**race**	black		1	-0.0759	0.0685	-0.2101	0.0583	1.23	0.2676
**race**	other race		1	0.0220	0.0641	-0.1036	0.1476	0.12	0.7315
**age_cat**	35-54		1	0.0221	0.0513	-0.0783	0.1226	0.19	0.6657
**age_cat**	greater than 55		1	-0.0790	0.0553	-0.1874	0.0295	2.04	0.1534
**inc**	40,000 - $99,999		1	0.0577	0.0414	-0.0236	0.1389	1.94	0.1640
**inc**	less than $40,000		1	0.1574	0.0525	0.0545	0.2603	8.98	0.0027
**inc**	missing		1	0.1094	0.1072	-0.1008	0.3195	1.04	0.3078
**edu**	missing		1	-0.4661	0.2706	-0.9965	0.0642	2.97	0.0849
**edu**	no college degree		1	0.0111	0.0486	-0.0842	0.1063	0.05	0.8200
**marital_status**	married or living as married		1	0.0838	0.0428	-0.0001	0.1677	3.84	0.0502
**marital_status**	missing		1	0.4281	0.2124	0.0118	0.8443	4.06	0.0438
**marital_status**	widowed, divorced		1	0.0120	0.0604	-0.1064	0.1304	0.04	0.8422
**kids**	missing		1	-0.3939	0.2586	-0.9007	0.1130	2.32	0.1277
**kids**	yes		1	-0.0058	0.0476	-0.0992	0.0876	0.01	0.9028
**hadajob**	no		1	0.0324	0.0440	-0.0538	0.1187	0.54	0.4613
**Dispersion**			1	0.1684	0.0141	0.1430	0.1984		

Note: The negative binomial dispersion parameter was estimated by maximum likelihood.

LR Statistics For Joint Tests			
Source	DF	Chi-Square	Pr > ChiSq
**gender*x12**	2	0.75	0.6860
**x1**	1	0.01	0.9422
**x2**	1	2.94	0.0863
**x3**	1	0.92	0.3363
**x4**	1	11.53	0.0007

The GENMOD Procedure

LR Statistics For Joint Tests			
Source	DF	Chi-Square	Pr > ChiSq
x5	1	1.78	0.1823
x6	1	1.68	0.1944
x8	1	3.96	0.0467
x9	1	0.16	0.6915
x10	1	5.10	0.0239
x11	1	0.02	0.8864
x12	1	0.19	0.6653
x13	1	1.86	0.1725
x14	1	0.85	0.3559
upset	3	12.78	0.0051
dur	1	17.29	<.0001
support_family	1	24.41	<.0001
support_doctor	1	2.52	0.1122
support_fedgovt	1	0.04	0.8383
support_city	1	5.39	0.0202
support_deptpubhealt	1	3.23	0.0723
support_faithcommuni	1	8.54	0.0035
support_socialservic	1	3.30	0.0694
support_neighbors	1	3.63	0.0568
support_other	1	2.65	0.1037
infected	1	0.08	0.7739
feltunwell	2	6.12	0.0470
compare_others	1	15.87	<.0001
ratehealth	1	0.57	0.4509
gender	2	13.03	0.0015
race	2	1.42	0.4912

The GENMOD Procedure

LR Statistics For Joint Tests			
**Source**	**DF**	**Chi-Square**	**Pr > ChiSq**
**age_cat**	2	5.63	0.0600
**inc**	3	9.36	0.0249
**edu**	2	3.15	0.2071
**marital_status**	3	7.69	0.0529
**kids**	2	2.36	0.3074
**hadajob**	1	0.54	0.4614

Note: Under full-rank parameterizations, Type 3 effect tests are replaced by joint tests. The joint test for an effect is a test that all the parameters associated with that effect are zero. Such joint tests might not be equivalent to Type 3 effect tests under GLM parameterization.

The GENMOD Procedure

Model Information		
**Data Set**	WORK.Z	
**Distribution**	Negative Binomial	
**Link Function**	Log	
**Dependent Variable**	DscoreR	HADS Depression score

**Number of Observations Read**	1293
**Number of Observations Used**	1293

Class Level Information				
**Class**	**Value**	**Design Variables**		
**gender**	**Female**	0	0	
	**Male**	1	0	
	**Other**	0	1	
**race**	**black**	1	0	
	**other race**	0	1	
	**white**	0	0	
**age_cat**	**35-54**	1	0	
	**greater than 55**	0	1	
	**less than 35**	0	0	
**inc**	**$100,000 and above**	0	0	0
	**40,000 - $99,999**	1	0	0
	**less than $40,000**	0	1	0
	**missing**	0	0	1
**edu**	**college degree or higher**	0	0	
	**missing**	1	0	
	**no college degree**	0	1	

The GENMOD Procedure

Class Level Information				
Class	Value	Design Variables		
kids	missing	1	0	
	no	0	0	
	yes	0	1	
marital_status	married or living as married	1	0	0
	missing	0	1	0
	single	0	0	0
	widowed, divorced	0	0	1
feltunwell	missing	1	0	
	no	0	0	
	yes	0	1	
compare_others	good to excellent	0		
	poor or fair	1		
ratehealth	good to excellent	0		
	poor or fair	1		
x1	no	0		
	yes	1		
x2	no	0		
	yes	1		
x3	no	0		
	yes	1		
x4	no	0		
	yes	1		
x5	no	0		
	yes	1		

The GENMOD Procedure

Class Level Information				
Class	Value	Design Variables		
x6	no	0		
	yes	1		
x8	no	0		
	yes	1		
x9	no	0		
	yes	1		
x10	no	0		
	yes	1		
x11	no	0		
	yes	1		
x12	no	0		
	yes	1		
x13	no	0		
	yes	1		
x14	no	0		
	yes	1		
upset	do not want to answer	1	0	0
	missing	0	0	0
	no	0	1	0
	yes	0	0	1
hadajob	no	1		
	yes	0		
infected	no	0		
	yes	1		

Fully adjusted models of Depression score with gender*theme interaction

The GENMOD Procedure

Criteria For Assessing Goodness Of Fit			
**Criterion**	**DF**	**Value**	**Value/DF**
**Deviance**	1242	1461.1509	1.1765
**Scaled Deviance**	1242	1461.1509	1.1765
**Pearson Chi-Square**	1242	1290.3302	1.0389
**Scaled Pearson X2**	1242	1290.3302	1.0389
**Log Likelihood**		7066.4734	
**Full Log Likelihood**		-3380.1090	
**AIC (smaller is better)**		6864.2181	
**AICC (smaller is better)**		6868.6632	
**BIC (smaller is better)**		7132.7835	

Algorithm converged.

Analysis Of Maximum Likelihood Parameter Estimates									
**Parameter**			**DF**	**Estimate**	**Standard Error**	**Wald 95% Confidence Limits**		**Wald Chi-Square**	**Pr > ChiSq**
**Intercept**			1	1.9399	0.1117	1.7209	2.1589	301.39	<.0001
**gender*x13**	Male	yes	1	0.4924	0.2369	0.0281	0.9566	4.32	0.0377
**gender*x13**	Other	yes	1	0.0736	0.5734	-1.0502	1.1974	0.02	0.8979
**x1**	yes		1	0.0040	0.0441	-0.0824	0.0905	0.01	0.9269
**x2**	yes		1	0.3619	0.2116	-0.0529	0.7767	2.92	0.0873
**x3**	yes		1	0.0744	0.0740	-0.0707	0.2194	1.01	0.3152
**x4**	yes		1	0.2090	0.0613	0.0887	0.3292	11.60	0.0007
**x5**	yes		1	0.0709	0.0546	-0.0360	0.1779	1.69	0.1935
**x6**	yes		1	0.0726	0.0561	-0.0373	0.1825	1.68	0.1953
**x8**	yes		1	0.1074	0.0534	0.0026	0.2122	4.04	0.0445
**x9**	yes		1	0.0924	0.1911	-0.2822	0.4669	0.23	0.6289

The GENMOD Procedure

Analysis Of Maximum Likelihood Parameter Estimates									
Parameter			DF	Estimate	Standard Error	Wald 95% Confidence Limits		Wald Chi-Square	Pr > ChiSq
**x10**	yes		1	0.1333	0.0591	0.0175	0.2492	5.09	0.0241
**x11**	yes		1	-0.0234	0.1537	-0.3247	0.2779	0.02	0.8792
**x12**	yes		1	0.0126	0.1988	-0.3771	0.4023	0.00	0.9494
**x13**	yes		1	0.0142	0.1211	-0.2231	0.2515	0.01	0.9066
**x14**	yes		1	0.1651	0.1820	-0.1916	0.5218	0.82	0.3644
**upset**	do not want to answer		1	0.0192	0.2253	-0.4223	0.4608	0.01	0.9320
**upset**	no		1	-0.0593	0.0417	-0.1410	0.0224	2.02	0.1550
**upset**	yes		1	0.1337	0.0644	0.0075	0.2600	4.31	0.0379
**dur**			1	0.0025	0.0006	0.0013	0.0037	16.73	<.0001
**support_family**			1	-0.0033	0.0007	-0.0046	-0.0020	24.74	<.0001
**support_doctor**			1	-0.0011	0.0007	-0.0024	0.0002	2.73	0.0986
**support_fedgovt**			1	0.0002	0.0009	-0.0016	0.0019	0.04	0.8411
**support_city**			1	0.0022	0.0010	0.0003	0.0042	4.92	0.0265
**support_deptpubhealt**			1	-0.0016	0.0009	-0.0034	0.0002	2.91	0.0878
**support_faithcommuni**			1	-0.0020	0.0007	-0.0034	-0.0007	8.51	0.0035
**support_socialservic**			1	0.0018	0.0009	-0.0000	0.0036	3.64	0.0564
**support_neighbors**			1	-0.0013	0.0007	-0.0026	0.0001	3.22	0.0729
**support_other**			1	-0.0015	0.0008	-0.0032	0.0001	3.19	0.0740
**infected**	yes		1	0.0545	0.1601	-0.2593	0.3683	0.12	0.7336
**feltunwell**	missing		1	0.1575	0.2938	-0.4184	0.7333	0.29	0.5920
**feltunwell**	yes		1	0.0965	0.0390	0.0202	0.1729	6.14	0.0132
**compare_others**	poor or fair		1	0.2434	0.0613	0.1233	0.3636	15.77	<.0001
**ratehealth**	poor or fair		1	-0.0692	0.0825	-0.2310	0.0925	0.70	0.4017
**gender**	Male		1	-0.1528	0.0398	-0.2307	-0.0748	14.76	0.0001
**gender**	Other		1	0.0622	0.1389	-0.2100	0.3344	0.20	0.6544

Fully adjusted models of Depression score with gender*theme interaction

The GENMOD Procedure

Analysis Of Maximum Likelihood Parameter Estimates									
Parameter			DF	Estimate	Standard Error	Wald 95% Confidence Limits		Wald Chi-Square	Pr > ChiSq
race	black		1	-0.0767	0.0683	-0.2106	0.0572	1.26	0.2616
race	other race		1	0.0225	0.0640	-0.1028	0.1479	0.12	0.7246
age_cat	35-54		1	0.0227	0.0511	-0.0775	0.1229	0.20	0.6570
age_cat	greater than 55		1	-0.0802	0.0551	-0.1883	0.0279	2.11	0.1460
inc	40,000 - $99,999		1	0.0585	0.0414	-0.0226	0.1396	2.00	0.1576
inc	less than $40,000		1	0.1588	0.0525	0.0560	0.2616	9.17	0.0025
inc	missing		1	0.1098	0.1070	-0.1000	0.3195	1.05	0.3052
edu	missing		1	-0.4715	0.2704	-1.0015	0.0586	3.04	0.0813
edu	no college degree		1	0.0123	0.0485	-0.0827	0.1073	0.06	0.7990
marital_status	married or living as married		1	0.0822	0.0427	-0.0015	0.1659	3.70	0.0543
marital_status	missing		1	0.4310	0.2120	0.0155	0.8466	4.13	0.0420
marital_status	widowed, divorced		1	0.0098	0.0603	-0.1084	0.1280	0.03	0.8709
kids	missing		1	-0.3903	0.2582	-0.8964	0.1158	2.28	0.1307
kids	yes		1	-0.0106	0.0476	-0.1039	0.0828	0.05	0.8246
hadajob	no		1	0.0355	0.0439	-0.0505	0.1214	0.65	0.4188
Dispersion			1	0.1673	0.0140	0.1419	0.1972		

Note: The negative binomial dispersion parameter was estimated by maximum likelihood.

LR Statistics For Joint Tests			
Source	DF	Chi-Square	Pr > ChiSq
gender*x13	2	4.38	0.1120
x1	1	0.01	0.9269
x2	1	2.95	0.0858
x3	1	1.01	0.3152
x4	1	11.58	0.0007

The GENMOD Procedure

LR Statistics For Joint Tests			
Source	DF	Chi-Square	Pr > ChiSq
x5	1	1.69	0.1936
x6	1	1.68	0.1954
x8	1	4.03	0.0446
x9	1	0.23	0.6286
x10	1	5.09	0.0241
x11	1	0.02	0.8792
x12	1	0.00	0.9494
x13	1	0.01	0.9066
x14	1	0.82	0.3639
upset	3	12.71	0.0053
dur	1	16.62	<.0001
support_family	1	24.59	<.0001
support_doctor	1	2.73	0.0987
support_fedgovt	1	0.04	0.8411
support_city	1	4.92	0.0265
support_deptpubhealt	1	2.91	0.0878
support_faithcommuni	1	8.49	0.0036
support_socialservic	1	3.64	0.0564
support_neighbors	1	3.21	0.0730
support_other	1	3.19	0.0741
infected	1	0.12	0.7335
feltunwell	2	6.32	0.0423
compare_others	1	15.76	<.0001
ratehealth	1	0.70	0.4018
gender	2	15.21	0.0005
race	2	1.46	0.4813

Fully adjusted models of Depression score with gender*theme interaction

The GENMOD Procedure

LR Statistics For Joint Tests			
**Source**	**DF**	**Chi-Square**	**Pr > ChiSq**
**age_cat**	2	5.84	0.0539
**inc**	3	9.53	0.0230
**edu**	2	3.24	0.1977
**marital_status**	3	7.68	0.0530
**kids**	2	2.34	0.3102
**hadajob**	1	0.65	0.4189

Note: Under full-rank parameterizations, Type 3 effect tests are replaced by joint tests. The joint test for an effect is a test that all the parameters associated with that effect are zero. Such joint tests might not be equivalent to Type 3 effect tests under GLM parameterization.

The MEANS Procedure

Analysis Variable : DscoreR HADS Depression score							
Gender	death: family or friend	N Obs	N	Mean	Std Dev	Minimum	Maximum
Female	no	925	925	6.4064865	3.7476055	0	21.0000000
	yes	24	24	6.3750000	3.9542217	0	16.0000000
Male	no	319	319	5.4702194	3.7271176	0	18.0000000
	yes	7	7	9.2857143	4.2706083	4.0000000	16.0000000
Other	no	17	17	7.8235294	3.6951876	0	16.0000000
	yes	1	1	13.0000000	.	13.0000000	13.0000000

Fully adjusted models of Depression score with gender*theme interaction

The GENMOD Procedure

Model Information		
**Data Set**	WORK.Z	
**Distribution**	Negative Binomial	
**Link Function**	Log	
**Dependent Variable**	DscoreR	HADS Depression score

**Number of Observations Read**	1293
**Number of Observations Used**	1293

Class Level Information				
**Class**	**Value**	**Design Variables**		
**gender**	**Female**	0	0	
	**Male**	1	0	
	**Other**	0	1	
**race**	**black**	1	0	
	**other race**	0	1	
	**white**	0	0	
**age_cat**	**35-54**	1	0	
	**greater than 55**	0	1	
	**less than 35**	0	0	
**inc**	**$100,000 and above**	0	0	0
	**40,000 - $99,999**	1	0	0
	**less than $40,000**	0	1	0
	**missing**	0	0	1
**edu**	**college degree or higher**	0	0	
	**missing**	1	0	
	**no college degree**	0	1	

Fully adjusted models of Depression score with gender*theme interaction

The GENMOD Procedure

Class Level Information				
Class	Value	Design Variables		
kids	missing	1	0	
	no	0	0	
	yes	0	1	
marital_status	married or living as married	1	0	0
	missing	0	1	0
	single	0	0	0
	widowed, divorced	0	0	1
feltunwell	missing	1	0	
	no	0	0	
	yes	0	1	
compare_others	good to excellent	0		
	poor or fair	1		
ratehealth	good to excellent	0		
	poor or fair	1		
x1	no	0		
	yes	1		
x2	no	0		
	yes	1		
x3	no	0		
	yes	1		
x4	no	0		
	yes	1		
x5	no	0		
	yes	1		

Fully adjusted models of Depression score with gender*theme interaction

The GENMOD Procedure

Class Level Information				
Class	Value	Design Variables		
x6	no	0		
	yes	1		
x8	no	0		
	yes	1		
x9	no	0		
	yes	1		
x10	no	0		
	yes	1		
x11	no	0		
	yes	1		
x12	no	0		
	yes	1		
x13	no	0		
	yes	1		
x14	no	0		
	yes	1		
upset	do not want to answer	1	0	0
	missing	0	0	0
	no	0	1	0
	yes	0	0	1
hadajob	no	1		
	yes	0		
infected	no	0		
	yes	1		

The GENMOD Procedure

Criteria For Assessing Goodness Of Fit			
**Criterion**	**DF**	**Value**	**Value/DF**
**Deviance**	1243	1460.3231	1.1748
**Scaled Deviance**	1243	1460.3231	1.1748
**Pearson Chi-Square**	1243	1286.1766	1.0347
**Scaled Pearson X2**	1243	1286.1766	1.0347
**Log Likelihood**		7065.2214	
**Full Log Likelihood**		-3381.3610	
**AIC (smaller is better)**		6864.7221	
**AICC (smaller is better)**		6868.9961	
**BIC (smaller is better)**		7128.1228	

Algorithm converged.

Analysis Of Maximum Likelihood Parameter Estimates									
**Parameter**			**DF**	**Estimate**	**Standard Error**	**Wald 95% Confidence Limits**		**Wald Chi-Square**	**Pr > ChiSq**
**Intercept**			1	1.9359	0.1119	1.7167	2.1552	299.48	<.0001
**gender*x14**	Male	yes	1	-1.0815	0.8416	-2.7311	0.5680	1.65	0.1988
**gender*x14**	Other	yes	0	0.0000	0.0000	0.0000	0.0000	.	.
**x1**	yes		1	0.0015	0.0442	-0.0851	0.0880	0.00	0.9737
**x2**	yes		1	0.3604	0.2119	-0.0549	0.7758	2.89	0.0890
**x3**	yes		1	0.0705	0.0741	-0.0747	0.2158	0.91	0.3411
**x4**	yes		1	0.2066	0.0614	0.0861	0.3270	11.30	0.0008
**x5**	yes		1	0.0692	0.0546	-0.0379	0.1763	1.60	0.2052
**x6**	yes		1	0.0707	0.0561	-0.0394	0.1807	1.58	0.2082
**x8**	yes		1	0.1046	0.0534	-0.0000	0.2093	3.84	0.0501
**x9**	yes		1	0.0750	0.1912	-0.2998	0.4498	0.15	0.6948

The GENMOD Procedure

Analysis Of Maximum Likelihood Parameter Estimates									
Parameter			DF	Estimate	Standard Error	Wald 95% Confidence Limits		Wald Chi-Square	Pr > ChiSq
**x10**	yes		1	0.1313	0.0592	0.0153	0.2472	4.92	0.0265
**x11**	yes		1	-0.0234	0.1539	-0.3251	0.2783	0.02	0.8791
**x12**	yes		1	0.0175	0.1990	-0.3725	0.4075	0.01	0.9299
**x13**	yes		1	0.1393	0.1034	-0.0632	0.3419	1.82	0.1776
**x14**	yes		1	0.2318	0.1889	-0.1384	0.6020	1.51	0.2197
**upset**	do not want to answer		1	0.0140	0.2102	-0.3979	0.4259	0.00	0.9469
**upset**	no		1	-0.0578	0.0418	-0.1397	0.0240	1.92	0.1659
**upset**	yes		1	0.1337	0.0645	0.0073	0.2601	4.30	0.0381
**dur**			1	0.0026	0.0006	0.0014	0.0038	17.39	<.0001
**support_family**			1	-0.0033	0.0007	-0.0046	-0.0020	24.95	<.0001
**support_doctor**			1	-0.0011	0.0007	-0.0024	0.0002	2.61	0.1061
**support_fedgovt**			1	0.0002	0.0009	-0.0016	0.0019	0.03	0.8591
**support_city**			1	0.0023	0.0010	0.0003	0.0042	5.10	0.0240
**support_deptpubhealt**			1	-0.0016	0.0009	-0.0034	0.0002	3.08	0.0793
**support_faithcommuni**			1	-0.0020	0.0007	-0.0034	-0.0007	8.69	0.0032
**support_socialservic**			1	0.0018	0.0009	-0.0001	0.0036	3.58	0.0585
**support_neighbors**			1	-0.0013	0.0007	-0.0027	0.0001	3.51	0.0610
**support_other**			1	-0.0014	0.0008	-0.0031	0.0002	2.94	0.0866
**infected**	yes		1	0.0450	0.1603	-0.2692	0.3592	0.08	0.7789
**feltunwell**	missing		1	0.1574	0.2942	-0.4192	0.7340	0.29	0.5925
**feltunwell**	yes		1	0.0945	0.0389	0.0181	0.1708	5.88	0.0153
**compare_others**	poor or fair		1	0.2429	0.0612	0.1229	0.3629	15.75	<.0001
**ratehealth**	poor or fair		1	-0.0601	0.0821	-0.2211	0.1009	0.54	0.4643
**gender**	Male		1	-0.1373	0.0393	-0.2143	-0.0602	12.18	0.0005
**gender**	Other		1	0.0642	0.1361	-0.2026	0.3311	0.22	0.6371

The GENMOD Procedure

Analysis Of Maximum Likelihood Parameter Estimates									
Parameter			DF	Estimate	Standard Error	Wald 95% Confidence Limits		Wald Chi-Square	Pr > ChiSq
race	black		1	-0.0767	0.0684	-0.2108	0.0573	1.26	0.2620
race	other race		1	0.0218	0.0640	-0.1037	0.1473	0.12	0.7337
age_cat	35-54		1	0.0205	0.0512	-0.0798	0.1208	0.16	0.6888
age_cat	greater than 55		1	-0.0798	0.0552	-0.1880	0.0284	2.09	0.1482
inc	40,000 - $99,999		1	0.0563	0.0414	-0.0249	0.1375	1.84	0.1744
inc	less than $40,000		1	0.1557	0.0525	0.0528	0.2586	8.79	0.0030
inc	missing		1	0.1064	0.1072	-0.1038	0.3165	0.98	0.3211
edu	missing		1	-0.4664	0.2705	-0.9965	0.0637	2.97	0.0846
edu	no college degree		1	0.0098	0.0485	-0.0853	0.1049	0.04	0.8398
marital_status	married or living as married		1	0.0845	0.0427	0.0008	0.1683	3.91	0.0480
marital_status	missing		1	0.4294	0.2123	0.0133	0.8455	4.09	0.0431
marital_status	widowed, divorced		1	0.0102	0.0604	-0.1081	0.1286	0.03	0.8656
kids	missing		1	-0.4047	0.2587	-0.9118	0.1024	2.45	0.1177
kids	yes		1	-0.0064	0.0476	-0.0998	0.0869	0.02	0.8924
hadajob	no		1	0.0312	0.0439	-0.0549	0.1173	0.50	0.4775
Dispersion			1	0.1682	0.0141	0.1427	0.1981		

Note: The negative binomial dispersion parameter was estimated by maximum likelihood.

LR Statistics For Joint Tests			
Source	DF	Chi-Square	Pr > ChiSq
gender*x14	2	3.69	0.1581
x1	1	0.00	0.9737
x2	1	2.92	0.0874
x3	1	0.91	0.3411
x4	1	11.29	0.0008

The GENMOD Procedure

LR Statistics For Joint Tests			
**Source**	**DF**	**Chi-Square**	**Pr > ChiSq**
**x5**	1	1.60	0.2052
**x6**	1	1.58	0.2082
**x8**	1	3.84	0.0502
**x9**	1	0.15	0.6946
**x10**	1	4.92	0.0265
**x11**	1	0.02	0.8791
**x12**	1	0.01	0.9299
**x13**	1	1.82	0.1772
**x14**	1	1.51	0.2185
**upset**	3	12.45	0.0060
**dur**	1	17.28	<.0001
**support_family**	1	24.80	<.0001
**support_doctor**	1	2.61	0.1062
**support_fedgovt**	1	0.03	0.8591
**support_city**	1	5.09	0.0240
**support_deptpubhealt**	1	3.08	0.0793
**support_faithcommuni**	1	8.66	0.0032
**support_socialservic**	1	3.58	0.0586
**support_neighbors**	1	3.51	0.0611
**support_other**	1	2.93	0.0867
**infected**	1	0.08	0.7788
**feltunwell**	2	6.07	0.0480
**compare_others**	1	15.74	<.0001
**ratehealth**	1	0.54	0.4644
**gender**	2	12.64	0.0018
**race**	2	1.45	0.4845

The GENMOD Procedure

LR Statistics For Joint Tests			
**Source**	**DF**	**Chi-Square**	**Pr > ChiSq**
**age_cat**	2	5.57	0.0618
**inc**	3	9.17	0.0272
**edu**	2	3.14	0.2078
**marital_status**	3	7.86	0.0490
**kids**	2	2.49	0.2879
**hadajob**	1	0.50	0.4776

Note: Under full-rank parameterizations, Type 3 effect tests are replaced by joint tests. The joint test for an effect is a test that all the parameters associated with that effect are zero. Such joint tests might not be equivalent to Type 3 effect tests under GLM parameterization.

Fully adjusted models of HARDSHIPS with gender*theme interaction

The GENMOD Procedure

Model Information	
Data Set	WORK.SCORES
Distribution	Normal
Link Function	Identity
Dependent Variable	Factor1

Number of Observations Read	1293
Number of Observations Used	1293

Class Level Information				
Class	Value	Design Variables		
gender	Female	0	0	
	Male	1	0	
	Other	0	1	
race	black	1	0	
	other race	0	1	
	white	0	0	
age_cat	35-54	1	0	
	greater than 55	0	1	
	less than 35	0	0	
inc	$100,000 and above	0	0	0
	40,000 - $99,999	1	0	0
	less than $40,000	0	1	0
	missing	0	0	1
edu	college degree or higher	0	0	
	missing	1	0	
	no college degree	0	1	

The GENMOD Procedure

Class Level Information				
Class	Value	Design Variables		
kids	missing	1	0	
	no	0	0	
	yes	0	1	
marital_status	married or living as married	1	0	0
	missing	0	1	0
	single	0	0	0
	widowed, divorced	0	0	1
feltunwell	missing	1	0	
	no	0	0	
	yes	0	1	
compare_others	good to excellent	0		
	poor or fair	1		
ratehealth	good to excellent	0		
	poor or fair	1		
x1	no	0		
	yes	1		
x2	no	0		
	yes	1		
x3	no	0		
	yes	1		
x4	no	0		
	yes	1		
x5	no	0		
	yes	1		

The GENMOD Procedure

Class Level Information				
Class	Value	Design Variables		
x6	no	0		
	yes	1		
x8	no	0		
	yes	1		
x9	no	0		
	yes	1		
x10	no	0		
	yes	1		
x11	no	0		
	yes	1		
x12	no	0		
	yes	1		
x13	no	0		
	yes	1		
x14	no	0		
	yes	1		
upset	do not want to answer	1	0	0
	missing	0	0	0
	no	0	1	0
	yes	0	0	1
hadajob	no	1		
	yes	0		
infected	no	0		
	yes	1		

Fully adjusted models of HARDSHIPS with gender*theme interaction

The GENMOD Procedure

Criteria For Assessing Goodness Of Fit			
**Criterion**	**DF**	**Value**	**Value/DF**
**Deviance**	1242	887.6105	0.7147
**Scaled Deviance**	1242	1293.0000	1.0411
**Pearson Chi-Square**	1242	887.6105	0.7147
**Scaled Pearson X2**	1242	1293.0000	1.0411
**Log Likelihood**		-1591.4824	
**Full Log Likelihood**		-1591.4824	
**AIC (smaller is better)**		3286.9647	
**AICC (smaller is better)**		3291.4099	
**BIC (smaller is better)**		3555.5302	

Algorithm converged.

Analysis Of Maximum Likelihood Parameter Estimates									
**Parameter**			**DF**	**Estimate**	**Standard Error**	**Wald 95% Confidence Limits**		**Wald Chi-Square**	**Pr > ChiSq**
**Intercept**			1	-0.4996	0.1629	-0.8189	-0.1803	9.40	0.0022
**gender*x1**	Male	yes	1	0.1001	0.1450	-0.1842	0.3843	0.48	0.4902
**gender*x1**	Other	yes	1	-0.0298	0.5398	-1.0878	1.0281	0.00	0.9559
**x1**	yes		1	-0.0680	0.0717	-0.2086	0.0726	0.90	0.3430
**x2**	yes		1	-0.0097	0.3195	-0.6360	0.6165	0.00	0.9757
**x3**	yes		1	-0.1479	0.1073	-0.3581	0.0624	1.90	0.1681
**x4**	yes		1	0.3951	0.0915	0.2157	0.5745	18.63	<.0001
**x5**	yes		1	0.1221	0.0794	-0.0335	0.2777	2.36	0.1242
**x6**	yes		1	-0.0889	0.0807	-0.2471	0.0692	1.21	0.2705
**x8**	yes		1	-0.0003	0.0782	-0.1537	0.1530	0.00	0.9966
**x9**	yes		1	-0.0142	0.2812	-0.5654	0.5371	0.00	0.9598

The GENMOD Procedure

Analysis Of Maximum Likelihood Parameter Estimates									
Parameter			DF	Estimate	Standard Error	Wald 95% Confidence Limits		Wald Chi-Square	Pr > ChiSq
**x10**	yes		1	0.0738	0.0866	-0.0959	0.2435	0.73	0.3941
**x11**	yes		1	0.0921	0.2184	-0.3359	0.5201	0.18	0.6732
**x12**	yes		1	0.5304	0.2821	-0.0226	1.0834	3.53	0.0601
**x13**	yes		1	-0.0148	0.1523	-0.3133	0.2837	0.01	0.9225
**x14**	yes		1	-0.0564	0.2686	-0.5828	0.4701	0.04	0.8338
**upset**	do not want to answer		1	-0.0398	0.3115	-0.6502	0.5707	0.02	0.8984
**upset**	no		1	-0.0079	0.0599	-0.1253	0.1094	0.02	0.8945
**upset**	yes		1	0.0529	0.0954	-0.1341	0.2399	0.31	0.5796
**dur**			1	0.0008	0.0009	-0.0009	0.0025	0.83	0.3617
**support_family**			1	-0.0040	0.0010	-0.0059	-0.0021	17.32	<.0001
**support_doctor**			1	0.0013	0.0010	-0.0005	0.0032	1.92	0.1654
**support_fedgovt**			1	0.0032	0.0013	0.0007	0.0057	6.29	0.0122
**support_city**			1	0.0029	0.0014	0.0002	0.0057	4.28	0.0385
**support_deptpubhealt**			1	-0.0002	0.0013	-0.0028	0.0024	0.02	0.8766
**support_faithcommuni**			1	0.0009	0.0010	-0.0010	0.0028	0.78	0.3761
**support_socialservic**			1	0.0060	0.0013	0.0034	0.0086	20.21	<.0001
**support_neighbors**			1	-0.0010	0.0010	-0.0029	0.0009	1.08	0.2980
**support_other**			1	0.0029	0.0012	0.0006	0.0053	5.99	0.0144
**infected**	yes		1	0.3563	0.2337	-0.1017	0.8143	2.32	0.1273
**feltunwell**	missing		1	-0.4406	0.4195	-1.2628	0.3816	1.10	0.2936
**feltunwell**	yes		1	0.0506	0.0570	-0.0612	0.1623	0.79	0.3750
**compare_others**	poor or fair		1	0.1857	0.0941	0.0012	0.3701	3.89	0.0485
**ratehealth**	poor or fair		1	0.1674	0.1251	-0.0779	0.4126	1.79	0.1810
**gender**	Male		1	-0.1356	0.0605	-0.2542	-0.0171	5.03	0.0250
**gender**	Other		1	0.4217	0.2262	-0.0215	0.8650	3.48	0.0622

Fully adjusted models of HARDSHIPS with gender*theme interaction

The GENMOD Procedure

Analysis Of Maximum Likelihood Parameter Estimates

Parameter			DF	Estimate	Standard Error	Wald 95% Confidence Limits		Wald Chi-Square	Pr > ChiSq
race	black		1	0.1265	0.0955	-0.0607	0.3136	1.75	0.1854
race	other race		1	-0.0662	0.0927	-0.2478	0.1155	0.51	0.4752
age_cat	35-54		1	0.0012	0.0739	-0.1436	0.1460	0.00	0.9873
age_cat	greater than 55		1	-0.1886	0.0797	-0.3448	-0.0325	5.60	0.0179
inc	40,000 - $99,999		1	0.1189	0.0586	0.0040	0.2338	4.12	0.0425
inc	less than $40,000		1	0.2250	0.0760	0.0761	0.3739	8.77	0.0031
inc	missing		1	0.1455	0.1516	-0.1517	0.4426	0.92	0.3373
edu	missing		1	-0.1390	0.3276	-0.7811	0.5031	0.18	0.6713
edu	no college degree		1	0.2981	0.0698	0.1614	0.4348	18.26	<.0001
marital_status	married or living as married		1	0.0730	0.0613	-0.0472	0.1931	1.42	0.2341
marital_status	missing		1	0.9391	0.3163	0.3191	1.5591	8.81	0.0030
marital_status	widowed, divorced		1	0.0799	0.0856	-0.0879	0.2476	0.87	0.3506
kids	missing		1	-0.2658	0.3352	-0.9228	0.3913	0.63	0.4279
kids	yes		1	0.1505	0.0687	0.0159	0.2851	4.80	0.0285
hadajob	no		1	-0.0097	0.0633	-0.1337	0.1143	0.02	0.8779
Scale			1	0.8285	0.0163	0.7972	0.8611		

Note: The scale parameter was estimated by maximum likelihood.

Fully adjusted models of HARDSHIPS with gender*theme interaction

The GENMOD Procedure

Model Information	
Data Set	WORK.SCORES
Distribution	Normal
Link Function	Identity
Dependent Variable	Factor1

Number of Observations Read	1293
Number of Observations Used	1293

Class Level Information				
Class	Value	Design Variables		
gender	Female	0	0	
	Male	1	0	
	Other	0	1	
race	black	1	0	
	other race	0	1	
	white	0	0	
age_cat	35-54	1	0	
	greater than 55	0	1	
	less than 35	0	0	
inc	$100,000 and above	0	0	0
	40,000 - $99,999	1	0	0
	less than $40,000	0	1	0
	missing	0	0	1
edu	college degree or higher	0	0	
	missing	1	0	
	no college degree	0	1	

Fully adjusted models of HARDSHIPS with gender*theme interaction

The GENMOD Procedure

Class Level Information				
**Class**	**Value**	**Design Variables**		
**kids**	**missing**	1	0	
	**no**	0	0	
	**yes**	0	1	
**marital_status**	**married or living as married**	1	0	0
	**missing**	0	1	0
	**single**	0	0	0
	**widowed, divorced**	0	0	1
**feltunwell**	**missing**	1	0	
	**no**	0	0	
	**yes**	0	1	
**compare_others**	**good to excellent**	0		
	**poor or fair**	1		
**ratehealth**	**good to excellent**	0		
	**poor or fair**	1		
**x1**	**no**	0		
	**yes**	1		
**x2**	**no**	0		
	**yes**	1		
**x3**	**no**	0		
	**yes**	1		
**x4**	**no**	0		
	**yes**	1		
**x5**	**no**	0		
	**yes**	1		

The GENMOD Procedure

Class Level Information				
Class	Value	Design Variables		
x6	no	0		
	yes	1		
x8	no	0		
	yes	1		
x9	no	0		
	yes	1		
x10	no	0		
	yes	1		
x11	no	0		
	yes	1		
x12	no	0		
	yes	1		
x13	no	0		
	yes	1		
x14	no	0		
	yes	1		
upset	do not want to answer	1	0	0
	missing	0	0	0
	no	0	1	0
	yes	0	0	1
hadajob	no	1		
	yes	0		
infected	no	0		
	yes	1		

The GENMOD Procedure

Criteria For Assessing Goodness Of Fit			
**Criterion**	**DF**	**Value**	**Value/DF**
**Deviance**	1243	884.8637	0.7119
**Scaled Deviance**	1243	1293.0000	1.0402
**Pearson Chi-Square**	1243	884.8637	0.7119
**Scaled Pearson X2**	1243	1293.0000	1.0402
**Log Likelihood**		-1589.4786	
**Full Log Likelihood**		-1589.4786	
**AIC (smaller is better)**		3280.9572	
**AICC (smaller is better)**		3285.2312	
**BIC (smaller is better)**		3544.3580	

Algorithm converged.

Analysis Of Maximum Likelihood Parameter Estimates									
**Parameter**			**DF**	**Estimate**	**Standard Error**	**Wald 95% Confidence Limits**		**Wald Chi-Square**	**Pr > ChiSq**
**Intercept**			1	-0.4987	0.1613	-0.8148	-0.1826	9.56	0.0020
**gender*x2**	Male	yes	1	1.4899	0.7022	0.1136	2.8662	4.50	0.0339
**gender*x2**	Other	yes	0	0.0000	0.0000	0.0000	0.0000	.	.
**x1**	yes		1	-0.0471	0.0632	-0.1709	0.0767	0.56	0.4558
**x2**	yes		1	-0.4327	0.3765	-1.1706	0.3051	1.32	0.2504
**x3**	yes		1	-0.1662	0.1069	-0.3757	0.0433	2.42	0.1201
**x4**	yes		1	0.3967	0.0914	0.2176	0.5758	18.85	<.0001
**x5**	yes		1	0.1221	0.0793	-0.0333	0.2775	2.37	0.1235
**x6**	yes		1	-0.0870	0.0805	-0.2449	0.0708	1.17	0.2799
**x8**	yes		1	-0.0024	0.0781	-0.1554	0.1507	0.00	0.9758
**x9**	yes		1	-0.0111	0.2807	-0.5613	0.5392	0.00	0.9686

Fully adjusted models of HARDSHIPS with gender*theme interaction

The GENMOD Procedure

Analysis Of Maximum Likelihood Parameter Estimates									
Parameter			DF	Estimate	Standard Error	Wald 95% Confidence Limits		Wald Chi-Square	Pr > ChiSq
x10	yes		1	0.0789	0.0863	-0.0903	0.2481	0.84	0.3607
x11	yes		1	0.1012	0.2180	-0.3260	0.5284	0.22	0.6424
x12	yes		1	0.5325	0.2816	-0.0194	1.0843	3.58	0.0586
x13	yes		1	-0.0148	0.1521	-0.3128	0.2832	0.01	0.9223
x14	yes		1	-0.0620	0.2682	-0.5876	0.4636	0.05	0.8173
upset	do not want to answer		1	-0.0447	0.3100	-0.6524	0.5630	0.02	0.8854
upset	no		1	-0.0100	0.0598	-0.1272	0.1071	0.03	0.8669
upset	yes		1	0.0513	0.0953	-0.1354	0.2380	0.29	0.5902
dur			1	0.0009	0.0009	-0.0009	0.0026	0.93	0.3346
support_family			1	-0.0040	0.0010	-0.0059	-0.0021	17.21	<.0001
support_doctor			1	0.0012	0.0009	-0.0006	0.0031	1.65	0.1994
support_fedgovt			1	0.0031	0.0013	0.0006	0.0056	6.03	0.0141
support_city			1	0.0031	0.0014	0.0003	0.0058	4.74	0.0295
support_deptpubhealt			1	-0.0003	0.0013	-0.0029	0.0022	0.05	0.8156
support_faithcommuni			1	0.0009	0.0010	-0.0010	0.0028	0.94	0.3319
support_socialservic			1	0.0058	0.0013	0.0032	0.0084	19.39	<.0001
support_neighbors			1	-0.0010	0.0010	-0.0029	0.0009	1.00	0.3174
support_other			1	0.0030	0.0012	0.0006	0.0053	6.18	0.0129
infected	yes		1	0.3521	0.2332	-0.1049	0.8092	2.28	0.1310
feltunwell	missing		1	-0.4430	0.4188	-1.2639	0.3779	1.12	0.2902
feltunwell	yes		1	0.0532	0.0568	-0.0582	0.1645	0.88	0.3496
compare_others	poor or fair		1	0.1848	0.0934	0.0018	0.3679	3.92	0.0478
ratehealth	poor or fair		1	0.1663	0.1245	-0.0778	0.4104	1.78	0.1818
gender	Male		1	-0.1277	0.0555	-0.2364	-0.0190	5.30	0.0214
gender	Other		1	0.4141	0.2052	0.0119	0.8163	4.07	0.0436

Fully adjusted models of HARDSHIPS with gender*theme interaction

The GENMOD Procedure

Analysis Of Maximum Likelihood Parameter Estimates									
Parameter			DF	Estimate	Standard Error	Wald 95% Confidence Limits		Wald Chi-Square	Pr > ChiSq
race	black		1	0.1206	0.0954	-0.0663	0.3076	1.60	0.2059
race	other race		1	-0.0573	0.0923	-0.2383	0.1237	0.38	0.5351
age_cat	35-54		1	-0.0053	0.0738	-0.1499	0.1394	0.01	0.9429
age_cat	greater than 55		1	-0.1928	0.0795	-0.3485	-0.0371	5.89	0.0152
inc	40,000 - $99,999		1	0.1146	0.0585	-0.0000	0.2292	3.84	0.0500
inc	less than $40,000		1	0.2219	0.0759	0.0732	0.3706	8.55	0.0034
inc	missing		1	0.1450	0.1514	-0.1517	0.4417	0.92	0.3380
edu	missing		1	-0.1143	0.3259	-0.7531	0.5244	0.12	0.7258
edu	no college degree		1	0.3038	0.0697	0.1672	0.4403	19.00	<.0001
marital_status	married or living as married		1	0.0738	0.0612	-0.0461	0.1938	1.45	0.2278
marital_status	missing		1	0.9406	0.3158	0.3216	1.5596	8.87	0.0029
marital_status	widowed, divorced		1	0.0768	0.0854	-0.0907	0.2442	0.81	0.3689
kids	missing		1	-0.2580	0.3346	-0.9138	0.3977	0.59	0.4406
kids	yes		1	0.1526	0.0685	0.0182	0.2869	4.96	0.0260
hadajob	no		1	-0.0100	0.0632	-0.1338	0.1138	0.03	0.8739
Scale			1	0.8273	0.0163	0.7960	0.8598		

Note: The scale parameter was estimated by maximum likelihood.

The MEANS Procedure

Analysis Variable : Factor1							
Gender	change in schooling	N Obs	N	Mean	Std Dev	Minimum	Maximum
Female	no	944	944	0.0305651	0.9272863	-1.6054018	3.0498838
	yes	5	5	-0.4296907	0.5645433	-1.2946979	0.2419713
Male	no	324	324	-0.1167453	0.8561453	-1.5983860	2.8628998
	yes	2	2	1.1437242	1.2630383	0.2506213	2.0368272
Other	no	18	18	0.4907243	0.7697516	-1.2707858	1.9088938

The GENMOD Procedure

Model Information	
Data Set	WORK.SCORES
Distribution	Normal
Link Function	Identity
Dependent Variable	Factor1

Number of Observations Read	1293
Number of Observations Used	1293

Class Level Information				
Class	Value	Design Variables		
gender	Female	0	0	
	Male	1	0	
	Other	0	1	
race	black	1	0	
	other race	0	1	
	white	0	0	
age_cat	35-54	1	0	
	greater than 55	0	1	
	less than 35	0	0	
inc	$100,000 and above	0	0	0
	40,000 - $99,999	1	0	0
	less than $40,000	0	1	0
	missing	0	0	1
edu	college degree or higher	0	0	
	missing	1	0	
	no college degree	0	1	

The GENMOD Procedure

Class Level Information				
Class	Value	Design Variables		
kids	missing	1	0	
	no	0	0	
	yes	0	1	
marital_status	married or living as married	1	0	0
	missing	0	1	0
	single	0	0	0
	widowed, divorced	0	0	1
feltunwell	missing	1	0	
	no	0	0	
	yes	0	1	
compare_others	good to excellent	0		
	poor or fair	1		
ratehealth	good to excellent	0		
	poor or fair	1		
x1	no	0		
	yes	1		
x2	no	0		
	yes	1		
x3	no	0		
	yes	1		
x4	no	0		
	yes	1		
x5	no	0		
	yes	1		

The GENMOD Procedure

Class Level Information				
Class	Value	Design Variables		
x6	no	0		
	yes	1		
x8	no	0		
	yes	1		
x9	no	0		
	yes	1		
x10	no	0		
	yes	1		
x11	no	0		
	yes	1		
x12	no	0		
	yes	1		
x13	no	0		
	yes	1		
x14	no	0		
	yes	1		
upset	do not want to answer	1	0	0
	missing	0	0	0
	no	0	1	0
	yes	0	0	1
hadajob	no	1		
	yes	0		
infected	no	0		
	yes	1		

Fully adjusted models of HARDSHIPS with gender*theme interaction

The GENMOD Procedure

Criteria For Assessing Goodness Of Fit			
**Criterion**	**DF**	**Value**	**Value/DF**
**Deviance**	1242	887.6792	0.7147
**Scaled Deviance**	1242	1293.0000	1.0411
**Pearson Chi-Square**	1242	887.6792	0.7147
**Scaled Pearson X2**	1242	1293.0000	1.0411
**Log Likelihood**		-1591.5324	
**Full Log Likelihood**		-1591.5324	
**AIC (smaller is better)**		3287.0648	
**AICC (smaller is better)**		3291.5100	
**BIC (smaller is better)**		3555.6303	

Algorithm converged.

Analysis Of Maximum Likelihood Parameter Estimates									
**Parameter**			**DF**	**Estimate**	**Standard Error**	**Wald 95% Confidence Limits**		**Wald Chi-Square**	**Pr > ChiSq**
**Intercept**			1	-0.5036	0.1620	-0.8212	-0.1860	9.66	0.0019
**gender*x3**	Male	yes	1	0.1299	0.2397	-0.3399	0.5997	0.29	0.5878
**gender*x3**	Other	yes	1	0.2882	0.8737	-1.4243	2.0007	0.11	0.7415
**x1**	yes		1	-0.0453	0.0635	-0.1697	0.0791	0.51	0.4751
**x2**	yes		1	-0.0239	0.3207	-0.6524	0.6047	0.01	0.9407
**x3**	yes		1	-0.1796	0.1161	-0.4072	0.0479	2.39	0.1217
**x4**	yes		1	0.3974	0.0915	0.2180	0.5767	18.85	<.0001
**x5**	yes		1	0.1222	0.0794	-0.0334	0.2778	2.37	0.1238
**x6**	yes		1	-0.0875	0.0807	-0.2457	0.0707	1.18	0.2783
**x8**	yes		1	0.0001	0.0782	-0.1532	0.1534	0.00	0.9992
**x9**	yes		1	-0.0110	0.2812	-0.5622	0.5401	0.00	0.9688

The GENMOD Procedure

Analysis Of Maximum Likelihood Parameter Estimates									
Parameter			DF	Estimate	Standard Error	Wald 95% Confidence Limits		Wald Chi-Square	Pr > ChiSq
**x10**	yes		1	0.0762	0.0865	-0.0933	0.2457	0.78	0.3783
**x11**	yes		1	0.1004	0.2184	-0.3277	0.5286	0.21	0.6457
**x12**	yes		1	0.5328	0.2821	-0.0200	1.0856	3.57	0.0589
**x13**	yes		1	-0.0121	0.1523	-0.3107	0.2865	0.01	0.9365
**x14**	yes		1	-0.0614	0.2686	-0.5878	0.4651	0.05	0.8192
**upset**	do not want to answer		1	-0.0381	0.3108	-0.6473	0.5710	0.02	0.9023
**upset**	no		1	-0.0075	0.0599	-0.1248	0.1098	0.02	0.9006
**upset**	yes		1	0.0510	0.0955	-0.1361	0.2381	0.29	0.5932
**dur**			1	0.0008	0.0009	-0.0009	0.0025	0.80	0.3710
**support_family**			1	-0.0040	0.0010	-0.0059	-0.0021	17.19	<.0001
**support_doctor**			1	0.0013	0.0010	-0.0005	0.0032	1.95	0.1621
**support_fedgovt**			1	0.0032	0.0013	0.0007	0.0057	6.19	0.0128
**support_city**			1	0.0029	0.0014	0.0002	0.0057	4.29	0.0384
**support_deptpubhealt**			1	-0.0002	0.0013	-0.0027	0.0024	0.02	0.8820
**support_faithcommuni**			1	0.0008	0.0010	-0.0011	0.0027	0.74	0.3887
**support_socialservic**			1	0.0059	0.0013	0.0033	0.0085	20.06	<.0001
**support_neighbors**			1	-0.0010	0.0010	-0.0029	0.0009	1.06	0.3028
**support_other**			1	0.0029	0.0012	0.0006	0.0053	5.92	0.0150
**infected**	yes		1	0.3501	0.2336	-0.1078	0.8079	2.25	0.1340
**feltunwell**	missing		1	-0.4407	0.4195	-1.2629	0.3816	1.10	0.2935
**feltunwell**	yes		1	0.0526	0.0570	-0.0590	0.1643	0.85	0.3555
**compare_others**	poor or fair		1	0.1849	0.0941	0.0004	0.3694	3.86	0.0495
**ratehealth**	poor or fair		1	0.1666	0.1250	-0.0784	0.4116	1.78	0.1825
**gender**	Male		1	-0.1263	0.0569	-0.2379	-0.0146	4.92	0.0266
**gender**	Other		1	0.4008	0.2124	-0.0155	0.8170	3.56	0.0592

The GENMOD Procedure

Analysis Of Maximum Likelihood Parameter Estimates

Parameter			DF	Estimate	Standard Error	Wald 95% Confidence Limits		Wald Chi-Square	Pr > ChiSq
race	black		1	0.1285	0.0955	-0.0587	0.3158	1.81	0.1786
race	other race		1	-0.0597	0.0926	-0.2412	0.1218	0.42	0.5190
age_cat	35-54		1	0.0029	0.0738	-0.1418	0.1476	0.00	0.9686
age_cat	greater than 55		1	-0.1865	0.0796	-0.3425	-0.0306	5.49	0.0191
inc	40,000 - $99,999		1	0.1157	0.0586	0.0009	0.2306	3.90	0.0483
inc	less than $40,000		1	0.2256	0.0760	0.0767	0.3745	8.81	0.0030
inc	missing		1	0.1440	0.1517	-0.1534	0.4413	0.90	0.3426
edu	missing		1	-0.1214	0.3264	-0.7612	0.5184	0.14	0.7100
edu	no college degree		1	0.2990	0.0698	0.1623	0.4358	18.37	<.0001
marital_status	married or living as married		1	0.0738	0.0613	-0.0463	0.1940	1.45	0.2284
marital_status	missing		1	0.9387	0.3164	0.3187	1.5588	8.80	0.0030
marital_status	widowed, divorced		1	0.0802	0.0856	-0.0875	0.2479	0.88	0.3486
kids	missing		1	-0.2532	0.3353	-0.9104	0.4040	0.57	0.4502
kids	yes		1	0.1515	0.0687	0.0170	0.2861	4.87	0.0273
hadajob	no		1	-0.0088	0.0633	-0.1328	0.1152	0.02	0.8893
Scale			1	0.8286	0.0163	0.7972	0.8611		

Note: The scale parameter was estimated by maximum likelihood.

Fully adjusted models of HARDSHIPS with gender*theme interaction

The GENMOD Procedure

Model Information	
**Data Set**	WORK.SCORES
**Distribution**	Normal
**Link Function**	Identity
**Dependent Variable**	Factor1

**Number of Observations Read**	1293
**Number of Observations Used**	1293

Class Level Information				
**Class**	**Value**	**Design Variables**		
**gender**	**Female**	0	0	
	**Male**	1	0	
	**Other**	0	1	
**race**	**black**	1	0	
	**other race**	0	1	
	**white**	0	0	
**age_cat**	**35-54**	1	0	
	**greater than 55**	0	1	
	**less than 35**	0	0	
**inc**	**$100,000 and above**	0	0	0
	**40,000 - $99,999**	1	0	0
	**less than $40,000**	0	1	0
	**missing**	0	0	1
**edu**	**college degree or higher**	0	0	
	**missing**	1	0	
	**no college degree**	0	1	

The GENMOD Procedure

Class Level Information				
**Class**	**Value**	**Design Variables**		
**kids**	**missing**	1	0	
	**no**	0	0	
	**yes**	0	1	
**marital_status**	**married or living as married**	1	0	0
	**missing**	0	1	0
	**single**	0	0	0
	**widowed, divorced**	0	0	1
**feltunwell**	**missing**	1	0	
	**no**	0	0	
	**yes**	0	1	
**compare_others**	**good to excellent**	0		
	**poor or fair**	1		
**ratehealth**	**good to excellent**	0		
	**poor or fair**	1		
**x1**	**no**	0		
	**yes**	1		
**x2**	**no**	0		
	**yes**	1		
**x3**	**no**	0		
	**yes**	1		
**x4**	**no**	0		
	**yes**	1		
**x5**	**no**	0		
	**yes**	1		

The GENMOD Procedure

Class Level Information				
Class	Value	Design Variables		
x6	no	0		
	yes	1		
x8	no	0		
	yes	1		
x9	no	0		
	yes	1		
x10	no	0		
	yes	1		
x11	no	0		
	yes	1		
x12	no	0		
	yes	1		
x13	no	0		
	yes	1		
x14	no	0		
	yes	1		
upset	do not want to answer	1	0	0
	missing	0	0	0
	no	0	1	0
	yes	0	0	1
hadajob	no	1		
	yes	0		
infected	no	0		
	yes	1		

The GENMOD Procedure

Criteria For Assessing Goodness Of Fit			
**Criterion**	**DF**	**Value**	**Value/DF**
**Deviance**	1243	887.4495	0.7140
**Scaled Deviance**	1243	1293.0000	1.0402
**Pearson Chi-Square**	1243	887.4495	0.7140
**Scaled Pearson X2**	1243	1293.0000	1.0402
**Log Likelihood**		-1591.3651	
**Full Log Likelihood**		-1591.3651	
**AIC (smaller is better)**		3284.7302	
**AICC (smaller is better)**		3289.0042	
**BIC (smaller is better)**		3548.1309	

Algorithm converged.

Analysis Of Maximum Likelihood Parameter Estimates									
**Parameter**			**DF**	**Estimate**	**Standard Error**	**Wald 95% Confidence Limits**		**Wald Chi-Square**	**Pr > ChiSq**
**Intercept**			1	-0.5164	0.1616	-0.8330	-0.1997	10.22	0.0014
**gender*x4**	Male	yes	1	-0.1778	0.2093	-0.5880	0.2325	0.72	0.3958
**gender*x4**	Other	yes	0	0.0000	0.0000	0.0000	0.0000	.	.
**x1**	yes		1	-0.0450	0.0633	-0.1690	0.0790	0.51	0.4768
**x2**	yes		1	-0.0068	0.3195	-0.6330	0.6194	0.00	0.9830
**x3**	yes		1	-0.1529	0.1069	-0.3624	0.0566	2.05	0.1525
**x4**	yes		1	0.4410	0.1052	0.2348	0.6472	17.58	<.0001
**x5**	yes		1	0.1229	0.0794	-0.0327	0.2785	2.40	0.1216
**x6**	yes		1	-0.0897	0.0806	-0.2478	0.0684	1.24	0.2660
**x8**	yes		1	0.0039	0.0783	-0.1495	0.1574	0.00	0.9600
**x9**	yes		1	-0.0073	0.2812	-0.5583	0.5438	0.00	0.9794

The GENMOD Procedure

Analysis Of Maximum Likelihood Parameter Estimates

Parameter			DF	Estimate	Standard Error	Wald 95% Confidence Limits		Wald Chi-Square	Pr > ChiSq
**x10**	yes		1	0.0732	0.0864	-0.0962	0.2426	0.72	0.3970
**x11**	yes		1	0.1050	0.2185	-0.3233	0.5333	0.23	0.6308
**x12**	yes		1	0.5312	0.2820	-0.0215	1.0839	3.55	0.0596
**x13**	yes		1	-0.0146	0.1523	-0.3131	0.2838	0.01	0.9234
**x14**	yes		1	-0.0654	0.2686	-0.5919	0.4611	0.06	0.8078
**upset**	do not want to answer		1	-0.0380	0.3105	-0.6465	0.5706	0.01	0.9027
**upset**	no		1	-0.0077	0.0598	-0.1250	0.1096	0.02	0.8972
**upset**	yes		1	0.0528	0.0954	-0.1341	0.2398	0.31	0.5797
**dur**			1	0.0008	0.0009	-0.0009	0.0025	0.84	0.3601
**support_family**			1	-0.0040	0.0010	-0.0059	-0.0021	17.21	<.0001
**support_doctor**			1	0.0014	0.0010	-0.0005	0.0032	2.02	0.1549
**support_fedgovt**			1	0.0032	0.0013	0.0007	0.0057	6.41	0.0113
**support_city**			1	0.0029	0.0014	0.0002	0.0057	4.36	0.0368
**support_deptpubhealt**			1	-0.0002	0.0013	-0.0027	0.0024	0.01	0.9034
**support_faithcommuni**			1	0.0008	0.0010	-0.0011	0.0027	0.75	0.3866
**support_socialservic**			1	0.0059	0.0013	0.0033	0.0085	19.67	<.0001
**support_neighbors**			1	-0.0010	0.0010	-0.0029	0.0009	1.05	0.3046
**support_other**			1	0.0030	0.0012	0.0006	0.0053	6.09	0.0136
**infected**	yes		1	0.3443	0.2337	-0.1138	0.8023	2.17	0.1407
**feltunwell**	missing		1	-0.4388	0.4195	-1.2609	0.3833	1.09	0.2955
**feltunwell**	yes		1	0.0521	0.0569	-0.0594	0.1637	0.84	0.3596
**compare_others**	poor or fair		1	0.1862	0.0935	0.0029	0.3695	3.96	0.0465
**ratehealth**	poor or fair		1	0.1638	0.1247	-0.0806	0.4083	1.73	0.1890
**gender**	Male		1	-0.1060	0.0574	-0.2186	0.0066	3.40	0.0650
**gender**	Other		1	0.4224	0.2055	0.0196	0.8252	4.22	0.0399

Fully adjusted models of HARDSHIPS with gender*theme interaction

The GENMOD Procedure

Analysis Of Maximum Likelihood Parameter Estimates

Parameter			DF	Estimate	Standard Error	Wald 95% Confidence Limits		Wald Chi-Square	Pr > ChiSq
**race**	black		1	0.1253	0.0955	-0.0619	0.3124	1.72	0.1896
**race**	other race		1	-0.0599	0.0925	-0.2412	0.1213	0.42	0.5169
**age_cat**	35-54		1	0.0050	0.0738	-0.1396	0.1497	0.00	0.9456
**age_cat**	greater than 55		1	-0.1866	0.0795	-0.3425	-0.0308	5.51	0.0189
**inc**	40,000 - $99,999		1	0.1160	0.0586	0.0012	0.2308	3.93	0.0476
**inc**	less than $40,000		1	0.2235	0.0760	0.0746	0.3724	8.65	0.0033
**inc**	missing		1	0.1459	0.1516	-0.1512	0.4430	0.93	0.3358
**edu**	missing		1	-0.1221	0.3264	-0.7617	0.5176	0.14	0.7084
**edu**	no college degree		1	0.2997	0.0698	0.1630	0.4364	18.46	<.0001
**marital_status**	married or living as married		1	0.0740	0.0613	-0.0462	0.1941	1.46	0.2275
**marital_status**	missing		1	0.9374	0.3163	0.3174	1.5573	8.78	0.0030
**marital_status**	widowed, divorced		1	0.0858	0.0857	-0.0821	0.2537	1.00	0.3165
**kids**	missing		1	-0.2610	0.3351	-0.9177	0.3957	0.61	0.4361
**kids**	yes		1	0.1512	0.0686	0.0167	0.2857	4.85	0.0276
**hadajob**	no		1	-0.0101	0.0633	-0.1341	0.1139	0.03	0.8731
**Scale**			1	0.8285	0.0163	0.7971	0.8610		

Note: The scale parameter was estimated by maximum likelihood.

The GENMOD Procedure

Model Information	
Data Set	WORK.SCORES
Distribution	Normal
Link Function	Identity
Dependent Variable	Factor1

Number of Observations Read	1293
Number of Observations Used	1293

Class Level Information

Class	Value	Design Variables		
gender	Female	0	0	
	Male	1	0	
	Other	0	1	
race	black	1	0	
	other race	0	1	
	white	0	0	
age_cat	35-54	1	0	
	greater than 55	0	1	
	less than 35	0	0	
inc	$100,000 and above	0	0	0
	40,000 - $99,999	1	0	0
	less than $40,000	0	1	0
	missing	0	0	1
edu	college degree or higher	0	0	
	missing	1	0	
	no college degree	0	1	

The GENMOD Procedure

Class Level Information				
Class	Value	Design Variables		
kids	missing	1	0	
	no	0	0	
	yes	0	1	
marital_status	married or living as married	1	0	0
	missing	0	1	0
	single	0	0	0
	widowed, divorced	0	0	1
feltunwell	missing	1	0	
	no	0	0	
	yes	0	1	
compare_others	good to excellent	0		
	poor or fair	1		
ratehealth	good to excellent	0		
	poor or fair	1		
x1	no	0		
	yes	1		
x2	no	0		
	yes	1		
x3	no	0		
	yes	1		
x4	no	0		
	yes	1		
x5	no	0		
	yes	1		

The GENMOD Procedure

Class Level Information				
**Class**	**Value**	**Design Variables**		
**x6**	**no**	0		
	**yes**	1		
**x8**	**no**	0		
	**yes**	1		
**x9**	**no**	0		
	**yes**	1		
**x10**	**no**	0		
	**yes**	1		
**x11**	**no**	0		
	**yes**	1		
**x12**	**no**	0		
	**yes**	1		
**x13**	**no**	0		
	**yes**	1		
**x14**	**no**	0		
	**yes**	1		
**upset**	**do not want to answer**	1	0	0
	**missing**	0	0	0
	**no**	0	1	0
	**yes**	0	0	1
**hadajob**	**no**	1		
	**yes**	0		
**infected**	**no**	0		
	**yes**	1		

Fully adjusted models of HARDSHIPS with gender*theme interaction

The GENMOD Procedure

Criteria For Assessing Goodness Of Fit			
**Criterion**	**DF**	**Value**	**Value/DF**
**Deviance**	1242	887.1159	0.7143
**Scaled Deviance**	1242	1293.0000	1.0411
**Pearson Chi-Square**	1242	887.1159	0.7143
**Scaled Pearson X2**	1242	1293.0000	1.0411
**Log Likelihood**		-1591.1221	
**Full Log Likelihood**		-1591.1221	
**AIC (smaller is better)**		3286.2441	
**AICC (smaller is better)**		3290.6893	
**BIC (smaller is better)**		3554.8096	

Algorithm converged.

Analysis Of Maximum Likelihood Parameter Estimates									
**Parameter**			**DF**	**Estimate**	**Standard Error**	**Wald 95% Confidence Limits**		**Wald Chi-Square**	**Pr > ChiSq**
**Intercept**			1	-0.5079	0.1615	-0.8244	-0.1914	9.89	0.0017
**gender*x5**	Male	yes	1	0.1979	0.1861	-0.1668	0.5627	1.13	0.2876
**gender*x5**	Other	yes	1	0.2791	0.8633	-1.4130	1.9712	0.10	0.7465
**x1**	yes		1	-0.0462	0.0633	-0.1701	0.0778	0.53	0.4655
**x2**	yes		1	-0.0062	0.3194	-0.6322	0.6199	0.00	0.9846
**x3**	yes		1	-0.1519	0.1069	-0.3614	0.0575	2.02	0.1551
**x4**	yes		1	0.3971	0.0915	0.2178	0.5764	18.84	<.0001
**x5**	yes		1	0.0752	0.0905	-0.1021	0.2526	0.69	0.4058
**x6**	yes		1	-0.0880	0.0807	-0.2461	0.0700	1.19	0.2750
**x8**	yes		1	0.0033	0.0782	-0.1500	0.1566	0.00	0.9663
**x9**	yes		1	-0.0013	0.2812	-0.5524	0.5499	0.00	0.9964

The GENMOD Procedure

Analysis Of Maximum Likelihood Parameter Estimates									
Parameter			DF	Estimate	Standard Error	Wald 95% Confidence Limits		Wald Chi-Square	Pr > ChiSq
**x10**	yes		1	0.0799	0.0865	-0.0897	0.2495	0.85	0.3558
**x11**	yes		1	0.0908	0.2183	-0.3370	0.5187	0.17	0.6773
**x12**	yes		1	0.5439	0.2822	-0.0092	1.0969	3.71	0.0539
**x13**	yes		1	-0.0129	0.1523	-0.3113	0.2855	0.01	0.9325
**x14**	yes		1	-0.0576	0.2685	-0.5839	0.4687	0.05	0.8302
**upset**	do not want to answer		1	-0.0415	0.3108	-0.6506	0.5676	0.02	0.8938
**upset**	no		1	-0.0081	0.0600	-0.1256	0.1094	0.02	0.8921
**upset**	yes		1	0.0489	0.0956	-0.1384	0.2362	0.26	0.6087
**dur**			1	0.0008	0.0009	-0.0009	0.0026	0.90	0.3437
**support_family**			1	-0.0040	0.0010	-0.0059	-0.0021	17.22	<.0001
**support_doctor**			1	0.0014	0.0010	-0.0005	0.0032	2.02	0.1556
**support_fedgovt**			1	0.0032	0.0013	0.0007	0.0057	6.37	0.0116
**support_city**			1	0.0030	0.0014	0.0002	0.0057	4.48	0.0342
**support_deptpubhealt**			1	-0.0003	0.0013	-0.0029	0.0023	0.05	0.8227
**support_faithcommuni**			1	0.0008	0.0010	-0.0010	0.0027	0.77	0.3814
**support_socialservic**			1	0.0059	0.0013	0.0033	0.0085	19.92	<.0001
**support_neighbors**			1	-0.0010	0.0010	-0.0029	0.0009	1.03	0.3102
**support_other**			1	0.0030	0.0012	0.0006	0.0053	6.13	0.0133
**infected**	yes		1	0.3572	0.2335	-0.1006	0.8149	2.34	0.1262
**feltunwell**	missing		1	-0.4400	0.4194	-1.2620	0.3819	1.10	0.2941
**feltunwell**	yes		1	0.0535	0.0569	-0.0581	0.1650	0.88	0.3476
**compare_others**	poor or fair		1	0.1839	0.0936	0.0005	0.3674	3.86	0.0494
**ratehealth**	poor or fair		1	0.1671	0.1247	-0.0774	0.4115	1.79	0.1804
**gender**	Male		1	-0.1371	0.0580	-0.2507	-0.0235	5.60	0.0180
**gender**	Other		1	0.4013	0.2116	-0.0134	0.8160	3.60	0.0579

The GENMOD Procedure

Analysis Of Maximum Likelihood Parameter Estimates									
Parameter			DF	Estimate	Standard Error	Wald 95% Confidence Limits		Wald Chi-Square	Pr > ChiSq
race	black		1	0.1264	0.0955	-0.0606	0.3135	1.75	0.1853
race	other race		1	-0.0591	0.0925	-0.2403	0.1222	0.41	0.5230
age_cat	35-54		1	0.0036	0.0738	-0.1410	0.1483	0.00	0.9607
age_cat	greater than 55		1	-0.1850	0.0795	-0.3409	-0.0292	5.42	0.0199
inc	40,000 - $99,999		1	0.1152	0.0586	0.0005	0.2300	3.87	0.0491
inc	less than $40,000		1	0.2237	0.0760	0.0748	0.3725	8.67	0.0032
inc	missing		1	0.1447	0.1516	-0.1524	0.4418	0.91	0.3397
edu	missing		1	-0.1166	0.3263	-0.7562	0.5231	0.13	0.7210
edu	no college degree		1	0.2987	0.0697	0.1620	0.4354	18.35	<.0001
marital_status	married or living as married		1	0.0742	0.0613	-0.0459	0.1944	1.47	0.2257
marital_status	missing		1	0.9320	0.3164	0.3119	1.5520	8.68	0.0032
marital_status	widowed, divorced		1	0.0807	0.0855	-0.0870	0.2483	0.89	0.3456
kids	missing		1	-0.2533	0.3350	-0.9100	0.4033	0.57	0.4496
kids	yes		1	0.1507	0.0686	0.0162	0.2852	4.82	0.0281
hadajob	no		1	-0.0100	0.0633	-0.1340	0.1140	0.02	0.8746
Scale			1	0.8283	0.0163	0.7970	0.8609		

Note: The scale parameter was estimated by maximum likelihood.

The GENMOD Procedure

Model Information	
Data Set	WORK.SCORES
Distribution	Normal
Link Function	Identity
Dependent Variable	Factor1

Number of Observations Read	1293
Number of Observations Used	1293

Class Level Information				
Class	Value	Design Variables		
gender	Female	0	0	
	Male	1	0	
	Other	0	1	
race	black	1	0	
	other race	0	1	
	white	0	0	
age_cat	35-54	1	0	
	greater than 55	0	1	
	less than 35	0	0	
inc	$100,000 and above	0	0	0
	40,000 - $99,999	1	0	0
	less than $40,000	0	1	0
	missing	0	0	1
edu	college degree or higher	0	0	
	missing	1	0	
	no college degree	0	1	

The GENMOD Procedure

Class Level Information				
**Class**	**Value**	**Design Variables**		
**kids**	**missing**	1	0	
	**no**	0	0	
	**yes**	0	1	
**marital_status**	**married or living as married**	1	0	0
	**missing**	0	1	0
	**single**	0	0	0
	**widowed, divorced**	0	0	1
**feltunwell**	**missing**	1	0	
	**no**	0	0	
	**yes**	0	1	
**compare_others**	**good to excellent**	0		
	**poor or fair**	1		
**ratehealth**	**good to excellent**	0		
	**poor or fair**	1		
**x1**	**no**	0		
	**yes**	1		
**x2**	**no**	0		
	**yes**	1		
**x3**	**no**	0		
	**yes**	1		
**x4**	**no**	0		
	**yes**	1		
**x5**	**no**	0		
	**yes**	1		

The GENMOD Procedure

Class Level Information				
Class	Value	Design Variables		
x6	no	0		
	yes	1		
x8	no	0		
	yes	1		
x9	no	0		
	yes	1		
x10	no	0		
	yes	1		
x11	no	0		
	yes	1		
x12	no	0		
	yes	1		
x13	no	0		
	yes	1		
x14	no	0		
	yes	1		
upset	do not want to answer	1	0	0
	missing	0	0	0
	no	0	1	0
	yes	0	0	1
hadajob	no	1		
	yes	0		
infected	no	0		
	yes	1		

The GENMOD Procedure

Criteria For Assessing Goodness Of Fit			
**Criterion**	**DF**	**Value**	**Value/DF**
**Deviance**	1242	887.9086	0.7149
**Scaled Deviance**	1242	1293.0000	1.0411
**Pearson Chi-Square**	1242	887.9086	0.7149
**Scaled Pearson X2**	1242	1293.0000	1.0411
**Log Likelihood**		-1591.6995	
**Full Log Likelihood**		-1591.6995	
**AIC (smaller is better)**		3287.3990	
**AICC (smaller is better)**		3291.8442	
**BIC (smaller is better)**		3555.9645	

Algorithm converged.

Analysis Of Maximum Likelihood Parameter Estimates									
**Parameter**			**DF**	**Estimate**	**Standard Error**	**Wald 95% Confidence Limits**		**Wald Chi-Square**	**Pr > ChiSq**
**Intercept**			1	-0.5107	0.1616	-0.8274	-0.1940	9.99	0.0016
**gender*x6**	Male	yes	1	-0.0427	0.1872	-0.4097	0.3242	0.05	0.8195
**gender*x6**	Other	yes	1	-0.0158	0.5370	-1.0684	1.0368	0.00	0.9765
**x1**	yes		1	-0.0462	0.0633	-0.1703	0.0779	0.53	0.4655
**x2**	yes		1	-0.0105	0.3197	-0.6370	0.6160	0.00	0.9738
**x3**	yes		1	-0.1535	0.1070	-0.3631	0.0562	2.06	0.1513
**x4**	yes		1	0.3966	0.0915	0.2172	0.5760	18.77	<.0001
**x5**	yes		1	0.1227	0.0794	-0.0329	0.2783	2.39	0.1223
**x6**	yes		1	-0.0791	0.0928	-0.2609	0.1028	0.73	0.3941
**x8**	yes		1	-0.0002	0.0783	-0.1537	0.1533	0.00	0.9982
**x9**	yes		1	-0.0097	0.2812	-0.5609	0.5415	0.00	0.9724

Fully adjusted models of HARDSHIPS with gender*theme interaction

The GENMOD Procedure

Analysis Of Maximum Likelihood Parameter Estimates									
Parameter			DF	Estimate	Standard Error	Wald 95% Confidence Limits		Wald Chi-Square	Pr > ChiSq
x10	yes		1	0.0741	0.0865	-0.0954	0.2436	0.73	0.3916
x11	yes		1	0.0954	0.2184	-0.3326	0.5234	0.19	0.6623
x12	yes		1	0.5305	0.2823	-0.0228	1.0838	3.53	0.0602
x13	yes		1	-0.0149	0.1523	-0.3135	0.2837	0.01	0.9222
x14	yes		1	-0.0588	0.2686	-0.5854	0.4677	0.05	0.8266
upset	do not want to answer		1	-0.0393	0.3113	-0.6495	0.5709	0.02	0.8996
upset	no		1	-0.0072	0.0599	-0.1246	0.1101	0.01	0.9037
upset	yes		1	0.0525	0.0955	-0.1346	0.2396	0.30	0.5822
dur			1	0.0008	0.0009	-0.0009	0.0025	0.81	0.3672
support_family			1	-0.0040	0.0010	-0.0059	-0.0021	17.12	<.0001
support_doctor			1	0.0013	0.0010	-0.0006	0.0032	1.87	0.1720
support_fedgovt			1	0.0032	0.0013	0.0007	0.0057	6.31	0.0120
support_city			1	0.0029	0.0014	0.0002	0.0057	4.36	0.0369
support_deptpubhealt			1	-0.0002	0.0013	-0.0028	0.0024	0.02	0.8786
support_faithcommuni			1	0.0009	0.0010	-0.0010	0.0028	0.82	0.3654
support_socialservic			1	0.0059	0.0013	0.0033	0.0085	20.04	<.0001
support_neighbors			1	-0.0010	0.0010	-0.0029	0.0009	1.06	0.3023
support_other			1	0.0030	0.0012	0.0006	0.0053	6.06	0.0138
infected	yes		1	0.3521	0.2336	-0.1058	0.8099	2.27	0.1318
feltunwell	missing		1	-0.4407	0.4196	-1.2631	0.3816	1.10	0.2935
feltunwell	yes		1	0.0516	0.0569	-0.0599	0.1632	0.82	0.3645
compare_others	poor or fair		1	0.1861	0.0936	0.0026	0.3696	3.95	0.0469
ratehealth	poor or fair		1	0.1643	0.1248	-0.0804	0.4090	1.73	0.1883
gender	Male		1	-0.1152	0.0578	-0.2285	-0.0019	3.97	0.0463
gender	Other		1	0.4214	0.2248	-0.0193	0.8621	3.51	0.0609

Fully adjusted models of HARDSHIPS with gender*theme interaction

The GENMOD Procedure

Analysis Of Maximum Likelihood Parameter Estimates

Parameter			DF	Estimate	Standard Error	Wald 95% Confidence Limits		Wald Chi-Square	Pr > ChiSq
race	black		1	0.1258	0.0956	-0.0615	0.3131	1.73	0.1881
race	other race		1	-0.0615	0.0925	-0.2428	0.1198	0.44	0.5059
age_cat	35-54		1	0.0038	0.0738	-0.1408	0.1485	0.00	0.9584
age_cat	greater than 55		1	-0.1864	0.0796	-0.3424	-0.0304	5.48	0.0192
inc	40,000 - $99,999		1	0.1161	0.0587	0.0011	0.2311	3.91	0.0479
inc	less than $40,000		1	0.2242	0.0760	0.0752	0.3733	8.69	0.0032
inc	missing		1	0.1466	0.1516	-0.1506	0.4438	0.93	0.3337
edu	missing		1	-0.1162	0.3270	-0.7570	0.5246	0.13	0.7223
edu	no college degree		1	0.2985	0.0698	0.1618	0.4352	18.31	<.0001
marital_status	married or living as married		1	0.0739	0.0613	-0.0463	0.1941	1.45	0.2283
marital_status	missing		1	0.9387	0.3164	0.3185	1.5588	8.80	0.0030
marital_status	widowed, divorced		1	0.0826	0.0856	-0.0852	0.2504	0.93	0.3348
kids	missing		1	-0.2613	0.3352	-0.9183	0.3958	0.61	0.4358
kids	yes		1	0.1516	0.0687	0.0171	0.2862	4.88	0.0272
hadajob	no		1	-0.0095	0.0633	-0.1335	0.1145	0.02	0.8806
Scale			1	0.8287	0.0163	0.7973	0.8612		

Note: The scale parameter was estimated by maximum likelihood.

The GENMOD Procedure

Model Information	
Data Set	WORK.SCORES
Distribution	Normal
Link Function	Identity
Dependent Variable	Factor1

Number of Observations Read	1293
Number of Observations Used	1293

Class Level Information				
Class	Value	Design Variables		
gender	Female	0	0	
	Male	1	0	
	Other	0	1	
race	black	1	0	
	other race	0	1	
	white	0	0	
age_cat	35-54	1	0	
	greater than 55	0	1	
	less than 35	0	0	
inc	$100,000 and above	0	0	0
	40,000 - $99,999	1	0	0
	less than $40,000	0	1	0
	missing	0	0	1
edu	college degree or higher	0	0	
	missing	1	0	
	no college degree	0	1	

The GENMOD Procedure

Class Level Information				
Class	Value	Design Variables		
kids	missing	1	0	
	no	0	0	
	yes	0	1	
marital_status	married or living as married	1	0	0
	missing	0	1	0
	single	0	0	0
	widowed, divorced	0	0	1
feltunwell	missing	1	0	
	no	0	0	
	yes	0	1	
compare_others	good to excellent	0		
	poor or fair	1		
ratehealth	good to excellent	0		
	poor or fair	1		
x1	no	0		
	yes	1		
x2	no	0		
	yes	1		
x3	no	0		
	yes	1		
x4	no	0		
	yes	1		
x5	no	0		
	yes	1		

The GENMOD Procedure

Class Level Information				
Class	Value	Design Variables		
x6	no	0		
	yes	1		
x8	no	0		
	yes	1		
x9	no	0		
	yes	1		
x10	no	0		
	yes	1		
x11	no	0		
	yes	1		
x12	no	0		
	yes	1		
x13	no	0		
	yes	1		
x14	no	0		
	yes	1		
upset	do not want to answer	1	0	0
	missing	0	0	0
	no	0	1	0
	yes	0	0	1
hadajob	no	1		
	yes	0		
infected	no	0		
	yes	1		

Fully adjusted models of HARDSHIPS with gender*theme interaction

The GENMOD Procedure

Criteria For Assessing Goodness Of Fit			
**Criterion**	**DF**	**Value**	**Value/DF**
**Deviance**	1242	885.1465	0.7127
**Scaled Deviance**	1242	1293.0000	1.0411
**Pearson Chi-Square**	1242	885.1465	0.7127
**Scaled Pearson X2**	1242	1293.0000	1.0411
**Log Likelihood**		-1589.6852	
**Full Log Likelihood**		-1589.6852	
**AIC (smaller is better)**		3283.3705	
**AICC (smaller is better)**		3287.8156	
**BIC (smaller is better)**		3551.9359	

Algorithm converged.

Analysis Of Maximum Likelihood Parameter Estimates									
**Parameter**			**DF**	**Estimate**	**Standard Error**	**Wald 95% Confidence Limits**		**Wald Chi-Square**	**Pr > ChiSq**
**Intercept**			1	-0.5206	0.1614	-0.8368	-0.2043	10.41	0.0013
**gender*x8**	Male	yes	1	-0.3742	0.2077	-0.7813	0.0328	3.25	0.0715
**gender*x8**	Other	yes	1	0.3767	0.4849	-0.5738	1.3271	0.60	0.4373
**x1**	yes		1	-0.0476	0.0632	-0.1715	0.0762	0.57	0.4510
**x2**	yes		1	-0.0088	0.3191	-0.6341	0.6166	0.00	0.9781
**x3**	yes		1	-0.1486	0.1068	-0.3579	0.0606	1.94	0.1638
**x4**	yes		1	0.4040	0.0915	0.2247	0.5833	19.50	<.0001
**x5**	yes		1	0.1215	0.0793	-0.0339	0.2769	2.35	0.1255
**x6**	yes		1	-0.0927	0.0806	-0.2508	0.0653	1.32	0.2501
**x8**	yes		1	0.0503	0.0860	-0.1182	0.2188	0.34	0.5585
**x9**	yes		1	-0.0160	0.2808	-0.5665	0.5344	0.00	0.9545

Fully adjusted models of HARDSHIPS with gender*theme interaction

The GENMOD Procedure

Analysis Of Maximum Likelihood Parameter Estimates									
Parameter			DF	Estimate	Standard Error	Wald 95% Confidence Limits		Wald Chi-Square	Pr > ChiSq
x10	yes		1	0.0645	0.0865	-0.1049	0.2340	0.56	0.4555
x11	yes		1	0.0823	0.2181	-0.3451	0.5098	0.14	0.7058
x12	yes		1	0.5348	0.2818	-0.0175	1.0871	3.60	0.0577
x13	yes		1	-0.0266	0.1524	-0.3254	0.2722	0.03	0.8617
x14	yes		1	-0.0722	0.2683	-0.5981	0.4537	0.07	0.7879
upset	do not want to answer		1	-0.0686	0.3121	-0.6802	0.5431	0.05	0.8260
upset	no		1	-0.0060	0.0598	-0.1232	0.1111	0.01	0.9197
upset	yes		1	0.0545	0.0953	-0.1323	0.2412	0.33	0.5677
dur			1	0.0008	0.0009	-0.0009	0.0026	0.85	0.3565
support_family			1	-0.0040	0.0010	-0.0059	-0.0021	17.29	<.0001
support_doctor			1	0.0013	0.0009	-0.0006	0.0032	1.86	0.1722
support_fedgovt			1	0.0032	0.0013	0.0007	0.0056	6.18	0.0129
support_city			1	0.0029	0.0014	0.0002	0.0057	4.31	0.0379
support_deptpubhealt			1	-0.0003	0.0013	-0.0028	0.0023	0.05	0.8314
support_faithcommuni			1	0.0008	0.0010	-0.0011	0.0027	0.73	0.3933
support_socialservic			1	0.0061	0.0013	0.0035	0.0087	20.88	<.0001
support_neighbors			1	-0.0010	0.0010	-0.0029	0.0009	1.02	0.3118
support_other			1	0.0030	0.0012	0.0006	0.0053	6.21	0.0127
infected	yes		1	0.3538	0.2332	-0.1033	0.8110	2.30	0.1292
feltunwell	missing		1	-0.4411	0.4189	-1.2621	0.3800	1.11	0.2924
feltunwell	yes		1	0.0591	0.0570	-0.0526	0.1708	1.07	0.2999
compare_others	poor or fair		1	0.1853	0.0934	0.0022	0.3683	3.93	0.0473
ratehealth	poor or fair		1	0.1611	0.1247	-0.0834	0.4056	1.67	0.1966
gender	Male		1	-0.0923	0.0574	-0.2048	0.0201	2.59	0.1076
gender	Other		1	0.3362	0.2301	-0.1148	0.7871	2.13	0.1440

Fully adjusted models of HARDSHIPS with gender*theme interaction

The GENMOD Procedure

Analysis Of Maximum Likelihood Parameter Estimates									
**Parameter**			**DF**	**Estimate**	**Standard Error**	**Wald 95% Confidence Limits**		**Wald Chi-Square**	**Pr > ChiSq**
**race**	black		1	0.1232	0.0954	-0.0637	0.3101	1.67	0.1963
**race**	other race		1	-0.0657	0.0924	-0.2468	0.1154	0.51	0.4769
**age_cat**	35-54		1	0.0082	0.0737	-0.1363	0.1526	0.01	0.9120
**age_cat**	greater than 55		1	-0.1824	0.0794	-0.3380	-0.0267	5.27	0.0216
**inc**	40,000 - $99,999		1	0.1210	0.0585	0.0063	0.2356	4.27	0.0387
**inc**	less than $40,000		1	0.2246	0.0759	0.0759	0.3733	8.76	0.0031
**inc**	missing		1	0.1459	0.1514	-0.1508	0.4427	0.93	0.3350
**edu**	missing		1	-0.1263	0.3260	-0.7652	0.5126	0.15	0.6984
**edu**	no college degree		1	0.2966	0.0697	0.1601	0.4331	18.13	<.0001
**marital_status**	married or living as married		1	0.0754	0.0612	-0.0446	0.1955	1.52	0.2180
**marital_status**	missing		1	0.9186	0.3161	0.2990	1.5381	8.45	0.0037
**marital_status**	widowed, divorced		1	0.0824	0.0854	-0.0850	0.2499	0.93	0.3345
**kids**	missing		1	-0.2192	0.3354	-0.8766	0.4382	0.43	0.5134
**kids**	yes		1	0.1469	0.0686	0.0124	0.2814	4.58	0.0323
**hadajob**	no		1	-0.0102	0.0632	-0.1341	0.1136	0.03	0.8712
**Scale**			1	0.8274	0.0163	0.7961	0.8599		

Note: The scale parameter was estimated by maximum likelihood.

Fully adjusted models of HARDSHIPS with gender*theme interaction

The GENMOD Procedure

Model Information	
Data Set	WORK.SCORES
Distribution	Normal
Link Function	Identity
Dependent Variable	Factor1

Number of Observations Read	1293
Number of Observations Used	1293

Class Level Information				
Class	Value	Design Variables		
gender	Female	0	0	
	Male	1	0	
	Other	0	1	
race	black	1	0	
	other race	0	1	
	white	0	0	
age_cat	35-54	1	0	
	greater than 55	0	1	
	less than 35	0	0	
inc	$100,000 and above	0	0	0
	40,000 - $99,999	1	0	0
	less than $40,000	0	1	0
	missing	0	0	1
edu	college degree or higher	0	0	
	missing	1	0	
	no college degree	0	1	

Fully adjusted models of HARDSHIPS with gender*theme interaction

The GENMOD Procedure

Class Level Information				
Class	Value	Design Variables		
kids	missing	1	0	
	no	0	0	
	yes	0	1	
marital_status	married or living as married	1	0	0
	missing	0	1	0
	single	0	0	0
	widowed, divorced	0	0	1
feltunwell	missing	1	0	
	no	0	0	
	yes	0	1	
compare_others	good to excellent	0		
	poor or fair	1		
ratehealth	good to excellent	0		
	poor or fair	1		
x1	no	0		
	yes	1		
x2	no	0		
	yes	1		
x3	no	0		
	yes	1		
x4	no	0		
	yes	1		
x5	no	0		
	yes	1		

The GENMOD Procedure

Class Level Information				
Class	Value	Design Variables		
x6	no	0		
	yes	1		
x8	no	0		
	yes	1		
x9	no	0		
	yes	1		
x10	no	0		
	yes	1		
x11	no	0		
	yes	1		
x12	no	0		
	yes	1		
x13	no	0		
	yes	1		
x14	no	0		
	yes	1		
upset	do not want to answer	1	0	0
	missing	0	0	0
	no	0	1	0
	yes	0	0	1
hadajob	no	1		
	yes	0		
infected	no	0		
	yes	1		

Fully adjusted models of HARDSHIPS with gender*theme interaction

The GENMOD Procedure

Criteria For Assessing Goodness Of Fit			
**Criterion**	**DF**	**Value**	**Value/DF**
**Deviance**	1243	887.3110	0.7138
**Scaled Deviance**	1243	1293.0000	1.0402
**Pearson Chi-Square**	1243	887.3110	0.7138
**Scaled Pearson X2**	1243	1293.0000	1.0402
**Log Likelihood**		-1591.2642	
**Full Log Likelihood**		-1591.2642	
**AIC (smaller is better)**		3284.5284	
**AICC (smaller is better)**		3288.8024	
**BIC (smaller is better)**		3547.9292	

Algorithm converged.

Analysis Of Maximum Likelihood Parameter Estimates									
**Parameter**			**DF**	**Estimate**	**Standard Error**	**Wald 95% Confidence Limits**		**Wald Chi-Square**	**Pr > ChiSq**
**Intercept**			1	-0.5196	0.1617	-0.8364	-0.2027	10.33	0.0013
**gender*x9**	Male	yes	1	0.6471	0.6735	-0.6730	1.9671	0.92	0.3367
**gender*x9**	Other	yes	0	0.0000	0.0000	0.0000	0.0000	.	.
**x1**	yes		1	-0.0468	0.0633	-0.1708	0.0772	0.55	0.4594
**x2**	yes		1	-0.0081	0.3195	-0.6342	0.6180	0.00	0.9798
**x3**	yes		1	-0.1505	0.1069	-0.3600	0.0590	1.98	0.1592
**x4**	yes		1	0.3975	0.0915	0.2182	0.5768	18.88	<.0001
**x5**	yes		1	0.1256	0.0794	-0.0301	0.2812	2.50	0.1139
**x6**	yes		1	-0.0888	0.0806	-0.2469	0.0692	1.21	0.2707
**x8**	yes		1	0.0017	0.0782	-0.1516	0.1549	0.00	0.9828
**x9**	yes		1	-0.1546	0.3190	-0.7798	0.4707	0.23	0.6280

The GENMOD Procedure

Analysis Of Maximum Likelihood Parameter Estimates									
Parameter			DF	Estimate	Standard Error	Wald 95% Confidence Limits		Wald Chi-Square	Pr > ChiSq
x10	yes		1	0.0706	0.0865	-0.0989	0.2402	0.67	0.4144
x11	yes		1	0.0959	0.2183	-0.3319	0.5236	0.19	0.6605
x12	yes		1	0.5314	0.2820	-0.0213	1.0840	3.55	0.0595
x13	yes		1	-0.0105	0.1523	-0.3090	0.2881	0.00	0.9452
x14	yes		1	-0.0602	0.2685	-0.5865	0.4661	0.05	0.8226
upset	do not want to answer		1	-0.0398	0.3105	-0.6483	0.5686	0.02	0.8979
upset	no		1	-0.0072	0.0598	-0.1244	0.1101	0.01	0.9049
upset	yes		1	0.0537	0.0954	-0.1333	0.2406	0.32	0.5737
dur			1	0.0008	0.0009	-0.0009	0.0026	0.90	0.3427
support_family			1	-0.0040	0.0010	-0.0059	-0.0021	16.96	<.0001
support_doctor			1	0.0013	0.0010	-0.0005	0.0032	1.92	0.1661
support_fedgovt			1	0.0032	0.0013	0.0007	0.0057	6.22	0.0126
support_city			1	0.0030	0.0014	0.0002	0.0057	4.39	0.0361
support_deptpubhealt			1	-0.0002	0.0013	-0.0028	0.0023	0.03	0.8670
support_faithcommuni			1	0.0009	0.0010	-0.0010	0.0028	0.81	0.3672
support_socialservic			1	0.0060	0.0013	0.0034	0.0086	20.35	<.0001
support_neighbors			1	-0.0010	0.0010	-0.0030	0.0009	1.12	0.2889
support_other			1	0.0030	0.0012	0.0006	0.0054	6.22	0.0127
infected	yes		1	0.3507	0.2335	-0.1070	0.8084	2.25	0.1332
feltunwell	missing		1	-0.4421	0.4194	-1.2642	0.3799	1.11	0.2918
feltunwell	yes		1	0.0525	0.0569	-0.0590	0.1640	0.85	0.3562
compare_others	poor or fair		1	0.1872	0.0935	0.0039	0.3706	4.01	0.0453
ratehealth	poor or fair		1	0.1622	0.1247	-0.0823	0.4067	1.69	0.1936
gender	Male		1	-0.1228	0.0555	-0.2316	-0.0139	4.89	0.0271
gender	Other		1	0.4201	0.2055	0.0174	0.8229	4.18	0.0409

Fully adjusted models of HARDSHIPS with gender*theme interaction

The GENMOD Procedure

Analysis Of Maximum Likelihood Parameter Estimates									
Parameter			DF	Estimate	Standard Error	Wald 95% Confidence Limits		Wald Chi-Square	Pr > ChiSq
race	black		1	0.1275	0.0955	-0.0596	0.3146	1.78	0.1817
race	other race		1	-0.0612	0.0925	-0.2424	0.1200	0.44	0.5083
age_cat	35-54		1	0.0083	0.0739	-0.1366	0.1532	0.01	0.9105
age_cat	greater than 55		1	-0.1845	0.0795	-0.3403	-0.0287	5.38	0.0203
inc	40,000 - $99,999		1	0.1178	0.0585	0.0031	0.2326	4.05	0.0442
inc	less than $40,000		1	0.2249	0.0760	0.0761	0.3738	8.77	0.0031
inc	missing		1	0.1473	0.1516	-0.1497	0.4444	0.94	0.3310
edu	missing		1	-0.1196	0.3263	-0.7592	0.5200	0.13	0.7139
edu	no college degree		1	0.2981	0.0697	0.1614	0.4348	18.27	<.0001
marital_status	married or living as married		1	0.0742	0.0613	-0.0459	0.1944	1.47	0.2259
marital_status	missing		1	0.9391	0.3163	0.3192	1.5590	8.82	0.0030
marital_status	widowed, divorced		1	0.0823	0.0855	-0.0853	0.2499	0.93	0.3358
kids	missing		1	-0.2585	0.3350	-0.9151	0.3982	0.60	0.4404
kids	yes		1	0.1477	0.0688	0.0129	0.2825	4.61	0.0318
hadajob	no		1	-0.0080	0.0633	-0.1319	0.1160	0.02	0.8998
Scale			1	0.8284	0.0163	0.7971	0.8609		

Note: The scale parameter was estimated by maximum likelihood.

The GENMOD Procedure

Model Information	
Data Set	WORK.SCORES
Distribution	Normal
Link Function	Identity
Dependent Variable	Factor1

Number of Observations Read	1293
Number of Observations Used	1293

Class Level Information

Class	Value	Design Variables		
gender	Female	0	0	
	Male	1	0	
	Other	0	1	
race	black	1	0	
	other race	0	1	
	white	0	0	
age_cat	35-54	1	0	
	greater than 55	0	1	
	less than 35	0	0	
inc	$100,000 and above	0	0	0
	40,000 - $99,999	1	0	0
	less than $40,000	0	1	0
	missing	0	0	1
edu	college degree or higher	0	0	
	missing	1	0	
	no college degree	0	1	

The GENMOD Procedure

Class Level Information				
**Class**	**Value**	**Design Variables**		
**kids**	**missing**	1	0	
	**no**	0	0	
	**yes**	0	1	
**marital_status**	**married or living as married**	1	0	0
	**missing**	0	1	0
	**single**	0	0	0
	**widowed, divorced**	0	0	1
**feltunwell**	**missing**	1	0	
	**no**	0	0	
	**yes**	0	1	
**compare_others**	**good to excellent**	0		
	**poor or fair**	1		
**ratehealth**	**good to excellent**	0		
	**poor or fair**	1		
**x1**	**no**	0		
	**yes**	1		
**x2**	**no**	0		
	**yes**	1		
**x3**	**no**	0		
	**yes**	1		
**x4**	**no**	0		
	**yes**	1		
**x5**	**no**	0		
	**yes**	1		

The GENMOD Procedure

Class Level Information				
Class	Value	Design Variables		
x6	no	0		
	yes	1		
x8	no	0		
	yes	1		
x9	no	0		
	yes	1		
x10	no	0		
	yes	1		
x11	no	0		
	yes	1		
x12	no	0		
	yes	1		
x13	no	0		
	yes	1		
x14	no	0		
	yes	1		
upset	do not want to answer	1	0	0
	missing	0	0	0
	no	0	1	0
	yes	0	0	1
hadajob	no	1		
	yes	0		
infected	no	0		
	yes	1		

Fully adjusted models of HARDSHIPS with gender*theme interaction

The GENMOD Procedure

Criteria For Assessing Goodness Of Fit			
**Criterion**	**DF**	**Value**	**Value/DF**
**Deviance**	1242	886.1160	0.7135
**Scaled Deviance**	1242	1293.0000	1.0411
**Pearson Chi-Square**	1242	886.1160	0.7135
**Scaled Pearson X2**	1242	1293.0000	1.0411
**Log Likelihood**		-1590.3929	
**Full Log Likelihood**		-1590.3929	
**AIC (smaller is better)**		3284.7858	
**AICC (smaller is better)**		3289.2310	
**BIC (smaller is better)**		3553.3513	

Algorithm converged.

Analysis Of Maximum Likelihood Parameter Estimates									
**Parameter**			**DF**	**Estimate**	**Standard Error**	**Wald 95% Confidence Limits**		**Wald Chi-Square**	**Pr > ChiSq**
**Intercept**			1	-0.4994	0.1615	-0.8159	-0.1830	9.57	0.0020
**gender*x10**	Male	yes	1	-0.1464	0.1914	-0.5216	0.2287	0.59	0.4442
**gender*x10**	Other	yes	1	-0.9579	0.6363	-2.2051	0.2893	2.27	0.1322
**x1**	yes		1	-0.0426	0.0632	-0.1665	0.0814	0.45	0.5008
**x2**	yes		1	-0.0171	0.3194	-0.6430	0.6088	0.00	0.9573
**x3**	yes		1	-0.1535	0.1068	-0.3628	0.0558	2.07	0.1506
**x4**	yes		1	0.3947	0.0915	0.2155	0.5740	18.63	<.0001
**x5**	yes		1	0.1183	0.0794	-0.0374	0.2740	2.22	0.1364
**x6**	yes		1	-0.0933	0.0806	-0.2514	0.0647	1.34	0.2470
**x8**	yes		1	0.0033	0.0782	-0.1501	0.1566	0.00	0.9669
**x9**	yes		1	-0.0020	0.2812	-0.5530	0.5491	0.00	0.9944

Fully adjusted models of HARDSHIPS with gender*theme interaction

The GENMOD Procedure

Analysis Of Maximum Likelihood Parameter Estimates									
**Parameter**			**DF**	**Estimate**	**Standard Error**	**Wald 95% Confidence Limits**		**Wald Chi-Square**	**Pr > ChiSq**
**x10**	yes		1	0.1320	0.1018	-0.0675	0.3315	1.68	0.1947
**x11**	yes		1	0.0927	0.2182	-0.3349	0.5202	0.18	0.6710
**x12**	yes		1	0.5133	0.2820	-0.0394	1.0660	3.31	0.0687
**x13**	yes		1	-0.0163	0.1522	-0.3146	0.2820	0.01	0.9147
**x14**	yes		1	-0.0583	0.2684	-0.5843	0.4677	0.05	0.8280
**upset**	do not want to answer		1	-0.0771	0.3111	-0.6869	0.5326	0.06	0.8042
**upset**	no		1	-0.0113	0.0598	-0.1286	0.1059	0.04	0.8496
**upset**	yes		1	0.0523	0.0954	-0.1347	0.2392	0.30	0.5836
**dur**			1	0.0007	0.0009	-0.0010	0.0025	0.68	0.4081
**support_family**			1	-0.0039	0.0010	-0.0058	-0.0021	16.74	<.0001
**support_doctor**			1	0.0013	0.0010	-0.0006	0.0032	1.88	0.1704
**support_fedgovt**			1	0.0032	0.0013	0.0007	0.0057	6.36	0.0117
**support_city**			1	0.0029	0.0014	0.0001	0.0057	4.26	0.0390
**support_deptpubhealt**			1	-0.0002	0.0013	-0.0027	0.0024	0.01	0.9059
**support_faithcommuni**			1	0.0008	0.0010	-0.0011	0.0027	0.76	0.3837
**support_socialservic**			1	0.0059	0.0013	0.0033	0.0085	19.51	<.0001
**support_neighbors**			1	-0.0010	0.0010	-0.0029	0.0009	0.99	0.3193
**support_other**			1	0.0029	0.0012	0.0006	0.0053	5.89	0.0152
**infected**	yes		1	0.3530	0.2334	-0.1044	0.8104	2.29	0.1304
**feltunwell**	missing		1	-0.4395	0.4191	-1.2610	0.3820	1.10	0.2944
**feltunwell**	yes		1	0.0485	0.0569	-0.0630	0.1601	0.73	0.3935
**compare_others**	poor or fair		1	0.1810	0.0935	-0.0022	0.3643	3.75	0.0528
**ratehealth**	poor or fair		1	0.1701	0.1247	-0.0743	0.4145	1.86	0.1724
**gender**	Male		1	-0.1063	0.0577	-0.2194	0.0067	3.40	0.0652
**gender**	Other		1	0.5257	0.2173	0.0998	0.9517	5.85	0.0156

Fully adjusted models of HARDSHIPS with gender*theme interaction

The GENMOD Procedure

Analysis Of Maximum Likelihood Parameter Estimates									
Parameter			DF	Estimate	Standard Error	Wald 95% Confidence Limits		Wald Chi-Square	Pr > ChiSq
race	black		1	0.1238	0.0954	-0.0632	0.3109	1.68	0.1945
race	other race		1	-0.0648	0.0924	-0.2459	0.1164	0.49	0.4834
age_cat	35-54		1	-0.0010	0.0738	-0.1456	0.1436	0.00	0.9894
age_cat	greater than 55		1	-0.1880	0.0795	-0.3438	-0.0323	5.60	0.0180
inc	40,000 - $99,999		1	0.1149	0.0585	0.0002	0.2296	3.86	0.0495
inc	less than $40,000		1	0.2265	0.0760	0.0776	0.3754	8.89	0.0029
inc	missing		1	0.1469	0.1515	-0.1500	0.4438	0.94	0.3322
edu	missing		1	-0.1051	0.3266	-0.7453	0.5351	0.10	0.7476
edu	no college degree		1	0.2998	0.0697	0.1632	0.4365	18.50	<.0001
marital_status	married or living as married		1	0.0691	0.0613	-0.0510	0.1893	1.27	0.2595
marital_status	missing		1	0.9348	0.3161	0.3152	1.5544	8.75	0.0031
marital_status	widowed, divorced		1	0.0826	0.0855	-0.0850	0.2501	0.93	0.3340
kids	missing		1	-0.2647	0.3349	-0.9211	0.3917	0.62	0.4294
kids	yes		1	0.1525	0.0686	0.0181	0.2870	4.94	0.0262
hadajob	no		1	-0.0112	0.0632	-0.1351	0.1127	0.03	0.8596
Scale			1	0.8278	0.0163	0.7965	0.8604		

Note: The scale parameter was estimated by maximum likelihood.

Fully adjusted models of HARDSHIPS with gender*theme interaction

The GENMOD Procedure

Model Information	
Data Set	WORK.SCORES
Distribution	Normal
Link Function	Identity
Dependent Variable	Factor1

Number of Observations Read	1293
Number of Observations Used	1293

Class Level Information				
Class	Value	Design Variables		
gender	Female	0	0	
	Male	1	0	
	Other	0	1	
race	black	1	0	
	other race	0	1	
	white	0	0	
age_cat	35-54	1	0	
	greater than 55	0	1	
	less than 35	0	0	
inc	$100,000 and above	0	0	0
	40,000 - $99,999	1	0	0
	less than $40,000	0	1	0
	missing	0	0	1
edu	college degree or higher	0	0	
	missing	1	0	
	no college degree	0	1	

The GENMOD Procedure

Class Level Information				
Class	Value	Design Variables		
kids	missing	1	0	
	no	0	0	
	yes	0	1	
marital_status	married or living as married	1	0	0
	missing	0	1	0
	single	0	0	0
	widowed, divorced	0	0	1
feltunwell	missing	1	0	
	no	0	0	
	yes	0	1	
compare_others	good to excellent	0		
	poor or fair	1		
ratehealth	good to excellent	0		
	poor or fair	1		
x1	no	0		
	yes	1		
x2	no	0		
	yes	1		
x3	no	0		
	yes	1		
x4	no	0		
	yes	1		
x5	no	0		
	yes	1		

The GENMOD Procedure

Class Level Information				
Class	Value	Design Variables		
x6	no	0		
	yes	1		
x8	no	0		
	yes	1		
x9	no	0		
	yes	1		
x10	no	0		
	yes	1		
x11	no	0		
	yes	1		
x12	no	0		
	yes	1		
x13	no	0		
	yes	1		
x14	no	0		
	yes	1		
upset	do not want to answer	1	0	0
	missing	0	0	0
	no	0	1	0
	yes	0	0	1
hadajob	no	1		
	yes	0		
infected	no	0		
	yes	1		

The GENMOD Procedure

Criteria For Assessing Goodness Of Fit			
**Criterion**	**DF**	**Value**	**Value/DF**
**Deviance**	1243	886.8626	0.7135
**Scaled Deviance**	1243	1293.0000	1.0402
**Pearson Chi-Square**	1243	886.8626	0.7135
**Scaled Pearson X2**	1243	1293.0000	1.0402
**Log Likelihood**		-1590.9374	
**Full Log Likelihood**		-1590.9374	
**AIC (smaller is better)**		3283.8749	
**AICC (smaller is better)**		3288.1489	
**BIC (smaller is better)**		3547.2756	

Algorithm converged.

Analysis Of Maximum Likelihood Parameter Estimates									
**Parameter**			**DF**	**Estimate**	**Standard Error**	**Wald 95% Confidence Limits**		**Wald Chi-Square**	**Pr > ChiSq**
**Intercept**			1	-0.5087	0.1614	-0.8250	-0.1924	9.94	0.0016
**gender*x11**	Male	yes	1	0.5819	0.4633	-0.3262	1.4900	1.58	0.2092
**gender*x11**	Other	yes	0	0.0000	0.0000	0.0000	0.0000	.	.
**x1**	yes		1	-0.0464	0.0632	-0.1703	0.0776	0.54	0.4633
**x2**	yes		1	-0.0096	0.3194	-0.6356	0.6164	0.00	0.9760
**x3**	yes		1	-0.1462	0.1070	-0.3558	0.0635	1.87	0.1717
**x4**	yes		1	0.3933	0.0915	0.2139	0.5726	18.46	<.0001
**x5**	yes		1	0.1209	0.0794	-0.0346	0.2765	2.32	0.1276
**x6**	yes		1	-0.0871	0.0806	-0.2451	0.0710	1.17	0.2803
**x8**	yes		1	0.0042	0.0782	-0.1491	0.1575	0.00	0.9567
**x9**	yes		1	-0.0121	0.2811	-0.5629	0.5388	0.00	0.9658

The GENMOD Procedure

Analysis Of Maximum Likelihood Parameter Estimates									
Parameter			DF	Estimate	Standard Error	Wald 95% Confidence Limits		Wald Chi-Square	Pr > ChiSq
x10	yes		1	0.0772	0.0864	-0.0922	0.2466	0.80	0.3716
x11	yes		1	-0.0966	0.2669	-0.6196	0.4264	0.13	0.7173
x12	yes		1	0.5316	0.2819	-0.0209	1.0841	3.56	0.0593
x13	yes		1	-0.0140	0.1522	-0.3123	0.2844	0.01	0.9270
x14	yes		1	-0.0619	0.2685	-0.5881	0.4643	0.05	0.8176
upset	do not want to answer		1	-0.0428	0.3104	-0.6512	0.5655	0.02	0.8903
upset	no		1	-0.0079	0.0598	-0.1251	0.1094	0.02	0.8953
upset	yes		1	0.0493	0.0954	-0.1377	0.2363	0.27	0.6057
dur			1	0.0008	0.0009	-0.0009	0.0026	0.90	0.3421
support_family			1	-0.0040	0.0010	-0.0059	-0.0021	17.51	<.0001
support_doctor			1	0.0013	0.0010	-0.0005	0.0032	1.93	0.1643
support_fedgovt			1	0.0031	0.0013	0.0007	0.0056	6.12	0.0133
support_city			1	0.0030	0.0014	0.0002	0.0057	4.39	0.0361
support_deptpubhealt			1	-0.0002	0.0013	-0.0028	0.0023	0.03	0.8611
support_faithcommuni			1	0.0009	0.0010	-0.0010	0.0028	0.80	0.3703
support_socialservic			1	0.0059	0.0013	0.0033	0.0085	19.85	<.0001
support_neighbors			1	-0.0010	0.0010	-0.0029	0.0009	1.05	0.3048
support_other			1	0.0029	0.0012	0.0006	0.0053	5.92	0.0150
infected	yes		1	0.3222	0.2347	-0.1377	0.7822	1.89	0.1697
feltunwell	missing		1	-0.4425	0.4193	-1.2643	0.3793	1.11	0.2913
feltunwell	yes		1	0.0518	0.0569	-0.0597	0.1633	0.83	0.3623
compare_others	poor or fair		1	0.1862	0.0935	0.0029	0.3694	3.96	0.0465
ratehealth	poor or fair		1	0.1663	0.1247	-0.0781	0.4107	1.78	0.1823
gender	Male		1	-0.1259	0.0557	-0.2350	-0.0168	5.12	0.0237
gender	Other		1	0.4150	0.2055	0.0123	0.8177	4.08	0.0434

The GENMOD Procedure

Analysis Of Maximum Likelihood Parameter Estimates									
Parameter			DF	Estimate	Standard Error	Wald 95% Confidence Limits		Wald Chi-Square	Pr > ChiSq
race	black		1	0.1268	0.0954	-0.0603	0.3139	1.77	0.1840
race	other race		1	-0.0606	0.0924	-0.2418	0.1205	0.43	0.5119
age_cat	35-54		1	0.0040	0.0738	-0.1406	0.1485	0.00	0.9572
age_cat	greater than 55		1	-0.1850	0.0795	-0.3408	-0.0292	5.42	0.0199
inc	40,000 - $99,999		1	0.1201	0.0586	0.0053	0.2349	4.21	0.0403
inc	less than $40,000		1	0.2298	0.0760	0.0808	0.3788	9.13	0.0025
inc	missing		1	0.1488	0.1515	-0.1482	0.4458	0.96	0.3262
edu	missing		1	-0.1206	0.3263	-0.7600	0.5189	0.14	0.7117
edu	no college degree		1	0.2972	0.0697	0.1605	0.4339	18.17	<.0001
marital_status	married or living as married		1	0.0766	0.0613	-0.0436	0.1968	1.56	0.2116
marital_status	missing		1	0.9471	0.3163	0.3272	1.5670	8.97	0.0027
marital_status	widowed, divorced		1	0.0846	0.0855	-0.0830	0.2522	0.98	0.3225
kids	missing		1	-0.2608	0.3349	-0.9173	0.3957	0.61	0.4362
kids	yes		1	0.1499	0.0686	0.0154	0.2844	4.77	0.0290
hadajob	no		1	-0.0086	0.0632	-0.1325	0.1153	0.02	0.8916
Scale			1	0.8282	0.0163	0.7969	0.8607		

Note: The scale parameter was estimated by maximum likelihood.

The GENMOD Procedure

Model Information	
**Data Set**	WORK.SCORES
**Distribution**	Normal
**Link Function**	Identity
**Dependent Variable**	Factor1

**Number of Observations Read**	1293
**Number of Observations Used**	1293

Class Level Information				
**Class**	**Value**	**Design Variables**		
**gender**	**Female**	0	0	
	**Male**	1	0	
	**Other**	0	1	
**race**	**black**	1	0	
	**other race**	0	1	
	**white**	0	0	
**age_cat**	**35-54**	1	0	
	**greater than 55**	0	1	
	**less than 35**	0	0	
**inc**	**$100,000 and above**	0	0	0
	**40,000 - $99,999**	1	0	0
	**less than $40,000**	0	1	0
	**missing**	0	0	1
**edu**	**college degree or higher**	0	0	
	**missing**	1	0	
	**no college degree**	0	1	

The GENMOD Procedure

Class Level Information				
Class	Value	Design Variables		
kids	missing	1	0	
	no	0	0	
	yes	0	1	
marital_status	married or living as married	1	0	0
	missing	0	1	0
	single	0	0	0
	widowed, divorced	0	0	1
feltunwell	missing	1	0	
	no	0	0	
	yes	0	1	
compare_others	good to excellent	0		
	poor or fair	1		
ratehealth	good to excellent	0		
	poor or fair	1		
x1	no	0		
	yes	1		
x2	no	0		
	yes	1		
x3	no	0		
	yes	1		
x4	no	0		
	yes	1		
x5	no	0		
	yes	1		

The GENMOD Procedure

Class Level Information				
Class	Value	Design Variables		
x6	no	0		
	yes	1		
x8	no	0		
	yes	1		
x9	no	0		
	yes	1		
x10	no	0		
	yes	1		
x11	no	0		
	yes	1		
x12	no	0		
	yes	1		
x13	no	0		
	yes	1		
x14	no	0		
	yes	1		
upset	do not want to answer	1	0	0
	missing	0	0	0
	no	0	1	0
	yes	0	0	1
hadajob	no	1		
	yes	0		
infected	no	0		
	yes	1		

The GENMOD Procedure

Criteria For Assessing Goodness Of Fit			
**Criterion**	**DF**	**Value**	**Value/DF**
**Deviance**	1242	885.3906	0.7129
**Scaled Deviance**	1242	1293.0000	1.0411
**Pearson Chi-Square**	1242	885.3906	0.7129
**Scaled Pearson X2**	1242	1293.0000	1.0411
**Log Likelihood**		-1589.8635	
**Full Log Likelihood**		-1589.8635	
**AIC (smaller is better)**		3283.7269	
**AICC (smaller is better)**		3288.1721	
**BIC (smaller is better)**		3552.2924	

Algorithm converged.

Analysis Of Maximum Likelihood Parameter Estimates									
**Parameter**			**DF**	**Estimate**	**Standard Error**	**Wald 95% Confidence Limits**		**Wald Chi-Square**	**Pr > ChiSq**
**Intercept**			1	-0.4966	0.1616	-0.8134	-0.1799	9.44	0.0021
**gender*x12**	Male	yes	1	-1.2760	0.6835	-2.6156	0.0635	3.49	0.0619
**gender*x12**	Other	yes	1	-0.7780	0.9341	-2.6088	1.0529	0.69	0.4049
**x1**	yes		1	-0.0475	0.0632	-0.1713	0.0764	0.56	0.4526
**x2**	yes		1	-0.0125	0.3191	-0.6379	0.6130	0.00	0.9689
**x3**	yes		1	-0.1543	0.1068	-0.3635	0.0549	2.09	0.1483
**x4**	yes		1	0.3958	0.0914	0.2167	0.5750	18.75	<.0001
**x5**	yes		1	0.1169	0.0794	-0.0387	0.2724	2.17	0.1408
**x6**	yes		1	-0.0922	0.0806	-0.2501	0.0657	1.31	0.2523
**x8**	yes		1	-0.0018	0.0781	-0.1549	0.1513	0.00	0.9813
**x9**	yes		1	-0.0084	0.2808	-0.5588	0.5420	0.00	0.9762

The GENMOD Procedure

Analysis Of Maximum Likelihood Parameter Estimates									
Parameter			DF	Estimate	Standard Error	Wald 95% Confidence Limits		Wald Chi-Square	Pr > ChiSq
x10	yes		1	0.0694	0.0864	-0.0998	0.2387	0.65	0.4215
x11	yes		1	0.0940	0.2180	-0.3333	0.5214	0.19	0.6662
x12	yes		1	0.8996	0.3439	0.2255	1.5737	6.84	0.0089
x13	yes		1	-0.0149	0.1521	-0.3130	0.2832	0.01	0.9218
x14	yes		1	-0.0600	0.2682	-0.5858	0.4657	0.05	0.8230
upset	do not want to answer		1	-0.0443	0.3103	-0.6525	0.5639	0.02	0.8865
upset	no		1	-0.0046	0.0598	-0.1218	0.1126	0.01	0.9383
upset	yes		1	0.0512	0.0953	-0.1355	0.2380	0.29	0.5908
dur			1	0.0008	0.0009	-0.0010	0.0025	0.74	0.3901
support_family			1	-0.0040	0.0010	-0.0059	-0.0021	17.17	<.0001
support_doctor			1	0.0013	0.0009	-0.0005	0.0032	1.94	0.1637
support_fedgovt			1	0.0032	0.0013	0.0007	0.0057	6.31	0.0120
support_city			1	0.0028	0.0014	0.0001	0.0056	4.00	0.0456
support_deptpubhealt			1	-0.0001	0.0013	-0.0027	0.0024	0.01	0.9120
support_faithcommuni			1	0.0009	0.0010	-0.0010	0.0028	0.81	0.3673
support_socialservic			1	0.0060	0.0013	0.0034	0.0086	20.60	<.0001
support_neighbors			1	-0.0010	0.0010	-0.0030	0.0009	1.12	0.2903
support_other			1	0.0028	0.0012	0.0004	0.0052	5.37	0.0205
infected	yes		1	0.3494	0.2333	-0.1079	0.8067	2.24	0.1343
feltunwell	missing		1	-0.4408	0.4190	-1.2619	0.3804	1.11	0.2928
feltunwell	yes		1	0.0527	0.0569	-0.0588	0.1641	0.86	0.3541
compare_others	poor or fair		1	0.1812	0.0935	-0.0020	0.3644	3.76	0.0526
ratehealth	poor or fair		1	0.1701	0.1246	-0.0741	0.4144	1.86	0.1722
gender	Male		1	-0.1120	0.0555	-0.2207	-0.0034	4.08	0.0433
gender	Other		1	0.4447	0.2102	0.0327	0.8568	4.48	0.0344

Fully adjusted models of HARDSHIPS with gender*theme interaction

The GENMOD Procedure

Analysis Of Maximum Likelihood Parameter Estimates

Parameter			DF	Estimate	Standard Error	Wald 95% Confidence Limits		Wald Chi-Square	Pr > ChiSq
race	black		1	0.1220	0.0954	-0.0650	0.3090	1.64	0.2010
race	other race		1	-0.0621	0.0923	-0.2431	0.1189	0.45	0.5010
age_cat	35-54		1	0.0009	0.0738	-0.1438	0.1455	0.00	0.9906
age_cat	greater than 55		1	-0.1895	0.0795	-0.3454	-0.0337	5.68	0.0171
inc	40,000 - $99,999		1	0.1191	0.0585	0.0045	0.2338	4.15	0.0417
inc	less than $40,000		1	0.2233	0.0759	0.0746	0.3721	8.66	0.0032
inc	missing		1	0.1494	0.1514	-0.1473	0.4462	0.97	0.3237
edu	missing		1	-0.1255	0.3260	-0.7645	0.5134	0.15	0.7002
edu	no college degree		1	0.2958	0.0697	0.1592	0.4325	18.01	<.0001
marital_status	married or living as married		1	0.0771	0.0613	-0.0430	0.1972	1.58	0.2081
marital_status	missing		1	0.9409	0.3159	0.3216	1.5601	8.87	0.0029
marital_status	widowed, divorced		1	0.0840	0.0854	-0.0835	0.2514	0.97	0.3258
kids	missing		1	-0.2663	0.3347	-0.9222	0.3897	0.63	0.4263
kids	yes		1	0.1502	0.0686	0.0158	0.2846	4.80	0.0285
hadajob	no		1	-0.0102	0.0633	-0.1343	0.1138	0.03	0.8716
Scale			1	0.8275	0.0163	0.7962	0.8600		

Note: The scale parameter was estimated by maximum likelihood.

The GENMOD Procedure

Model Information	
**Data Set**	WORK.SCORES
**Distribution**	Normal
**Link Function**	Identity
**Dependent Variable**	Factor1

**Number of Observations Read**	1293
**Number of Observations Used**	1293

Class Level Information				
**Class**	**Value**	**Design Variables**		
**gender**	**Female**	0	0	
	**Male**	1	0	
	**Other**	0	1	
**race**	**black**	1	0	
	**other race**	0	1	
	**white**	0	0	
**age_cat**	**35-54**	1	0	
	**greater than 55**	0	1	
	**less than 35**	0	0	
**inc**	**$100,000 and above**	0	0	0
	**40,000 - $99,999**	1	0	0
	**less than $40,000**	0	1	0
	**missing**	0	0	1
**edu**	**college degree or higher**	0	0	
	**missing**	1	0	
	**no college degree**	0	1	

The GENMOD Procedure

Class Level Information				
Class	Value	Design Variables		
kids	missing	1	0	
	no	0	0	
	yes	0	1	
marital_status	married or living as married	1	0	0
	missing	0	1	0
	single	0	0	0
	widowed, divorced	0	0	1
feltunwell	missing	1	0	
	no	0	0	
	yes	0	1	
compare_others	good to excellent	0		
	poor or fair	1		
ratehealth	good to excellent	0		
	poor or fair	1		
x1	no	0		
	yes	1		
x2	no	0		
	yes	1		
x3	no	0		
	yes	1		
x4	no	0		
	yes	1		
x5	no	0		
	yes	1		

The GENMOD Procedure

Class Level Information				
Class	Value	Design Variables		
x6	no	0		
	yes	1		
x8	no	0		
	yes	1		
x9	no	0		
	yes	1		
x10	no	0		
	yes	1		
x11	no	0		
	yes	1		
x12	no	0		
	yes	1		
x13	no	0		
	yes	1		
x14	no	0		
	yes	1		
upset	do not want to answer	1	0	0
	missing	0	0	0
	no	0	1	0
	yes	0	0	1
hadajob	no	1		
	yes	0		
infected	no	0		
	yes	1		

The GENMOD Procedure

Criteria For Assessing Goodness Of Fit			
**Criterion**	**DF**	**Value**	**Value/DF**
**Deviance**	1242	884.4489	0.7121
**Scaled Deviance**	1242	1293.0000	1.0411
**Pearson Chi-Square**	1242	884.4489	0.7121
**Scaled Pearson X2**	1242	1293.0000	1.0411
**Log Likelihood**		-1589.1755	
**Full Log Likelihood**		-1589.1755	
**AIC (smaller is better)**		3282.3511	
**AICC (smaller is better)**		3286.7962	
**BIC (smaller is better)**		3550.9165	

Algorithm converged.

Analysis Of Maximum Likelihood Parameter Estimates									
**Parameter**			**DF**	**Estimate**	**Standard Error**	**Wald 95% Confidence Limits**		**Wald Chi-Square**	**Pr > ChiSq**
**Intercept**			1	-0.5114	0.1612	-0.8273	-0.1956	10.07	0.0015
**gender*x13**	Male	yes	1	0.4678	0.3615	-0.2407	1.1763	1.67	0.1956
**gender*x13**	Other	yes	1	1.8282	0.9302	0.0050	3.6514	3.86	0.0494
**x1**	yes		1	-0.0442	0.0632	-0.1680	0.0795	0.49	0.4837
**x2**	yes		1	-0.0112	0.3189	-0.6363	0.6140	0.00	0.9721
**x3**	yes		1	-0.1522	0.1067	-0.3614	0.0569	2.04	0.1536
**x4**	yes		1	0.3935	0.0914	0.2144	0.5726	18.55	<.0001
**x5**	yes		1	0.1236	0.0793	-0.0317	0.2790	2.43	0.1188
**x6**	yes		1	-0.0872	0.0805	-0.2450	0.0706	1.17	0.2786
**x8**	yes		1	-0.0061	0.0782	-0.1593	0.1471	0.01	0.9378
**x9**	yes		1	0.0070	0.2809	-0.5435	0.5575	0.00	0.9801

The GENMOD Procedure

Analysis Of Maximum Likelihood Parameter Estimates									
Parameter			DF	Estimate	Standard Error	Wald 95% Confidence Limits		Wald Chi-Square	Pr > ChiSq
x10	yes		1	0.0783	0.0863	-0.0909	0.2474	0.82	0.3644
x11	yes		1	0.0953	0.2179	-0.3318	0.5224	0.19	0.6619
x12	yes		1	0.5313	0.2815	-0.0205	1.0831	3.56	0.0591
x13	yes		1	-0.1651	0.1744	-0.5069	0.1767	0.90	0.3437
x14	yes		1	-0.0621	0.2681	-0.5875	0.4634	0.05	0.8169
upset	do not want to answer		1	-0.2402	0.3279	-0.8828	0.4025	0.54	0.4639
upset	no		1	-0.0070	0.0598	-0.1241	0.1101	0.01	0.9069
upset	yes		1	0.0569	0.0953	-0.1298	0.2435	0.36	0.5505
dur			1	0.0007	0.0009	-0.0010	0.0025	0.68	0.4101
support_family			1	-0.0039	0.0010	-0.0058	-0.0020	16.26	<.0001
support_doctor			1	0.0013	0.0009	-0.0006	0.0032	1.87	0.1720
support_fedgovt			1	0.0032	0.0013	0.0007	0.0057	6.49	0.0108
support_city			1	0.0029	0.0014	0.0001	0.0057	4.25	0.0393
support_deptpubhealt			1	-0.0002	0.0013	-0.0027	0.0024	0.01	0.9040
support_faithcommuni			1	0.0009	0.0010	-0.0010	0.0028	0.82	0.3639
support_socialservic			1	0.0059	0.0013	0.0033	0.0085	19.81	<.0001
support_neighbors			1	-0.0009	0.0010	-0.0028	0.0010	0.86	0.3528
support_other			1	0.0029	0.0012	0.0005	0.0052	5.72	0.0168
infected	yes		1	0.3572	0.2332	-0.0999	0.8143	2.35	0.1256
feltunwell	missing		1	-0.4461	0.4187	-1.2668	0.3746	1.13	0.2868
feltunwell	yes		1	0.0566	0.0569	-0.0549	0.1680	0.99	0.3199
compare_others	poor or fair		1	0.1723	0.0936	-0.0112	0.3558	3.39	0.0657
ratehealth	poor or fair		1	0.1836	0.1251	-0.0616	0.4289	2.15	0.1422
gender	Male		1	-0.1312	0.0559	-0.2407	-0.0216	5.51	0.0189
gender	Other		1	0.3434	0.2089	-0.0661	0.7529	2.70	0.1002

The GENMOD Procedure

Analysis Of Maximum Likelihood Parameter Estimates									
Parameter			DF	Estimate	Standard Error	Wald 95% Confidence Limits		Wald Chi-Square	Pr > ChiSq
race	black		1	0.1232	0.0953	-0.0636	0.3101	1.67	0.1961
race	other race		1	-0.0602	0.0923	-0.2411	0.1208	0.42	0.5146
age_cat	35-54		1	0.0078	0.0737	-0.1367	0.1522	0.01	0.9160
age_cat	greater than 55		1	-0.1842	0.0794	-0.3397	-0.0286	5.38	0.0203
inc	40,000 - $99,999		1	0.1170	0.0585	0.0025	0.2316	4.01	0.0452
inc	less than $40,000		1	0.2303	0.0759	0.0816	0.3790	9.22	0.0024
inc	missing		1	0.1449	0.1513	-0.1517	0.4415	0.92	0.3383
edu	missing		1	-0.0957	0.3261	-0.7349	0.5434	0.09	0.7691
edu	no college degree		1	0.3015	0.0696	0.1650	0.4380	18.74	<.0001
marital_status	married or living as married		1	0.0737	0.0612	-0.0462	0.1937	1.45	0.2281
marital_status	missing		1	0.9320	0.3158	0.3130	1.5509	8.71	0.0032
marital_status	widowed, divorced		1	0.0825	0.0854	-0.0849	0.2498	0.93	0.3343
kids	missing		1	-0.2587	0.3345	-0.9144	0.3969	0.60	0.4393
kids	yes		1	0.1463	0.0686	0.0119	0.2807	4.55	0.0329
hadajob	no		1	-0.0084	0.0631	-0.1322	0.1154	0.02	0.8941
Scale			1	0.8271	0.0163	0.7958	0.8596		

Note: The scale parameter was estimated by maximum likelihood.

The GENMOD Procedure

Model Information	
Data Set	WORK.SCORES
Distribution	Normal
Link Function	Identity
Dependent Variable	Factor1

Number of Observations Read	1293
Number of Observations Used	1293

Class Level Information				
Class	Value	Design Variables		
gender	Female	0	0	
	Male	1	0	
	Other	0	1	
race	black	1	0	
	other race	0	1	
	white	0	0	
age_cat	35-54	1	0	
	greater than 55	0	1	
	less than 35	0	0	
inc	$100,000 and above	0	0	0
	40,000 - $99,999	1	0	0
	less than $40,000	0	1	0
	missing	0	0	1
edu	college degree or higher	0	0	
	missing	1	0	
	no college degree	0	1	

The GENMOD Procedure

Class Level Information				
**Class**	**Value**	**Design Variables**		
**kids**	**missing**	1	0	
	**no**	0	0	
	**yes**	0	1	
**marital_status**	**married or living as married**	1	0	0
	**missing**	0	1	0
	**single**	0	0	0
	**widowed, divorced**	0	0	1
**feltunwell**	**missing**	1	0	
	**no**	0	0	
	**yes**	0	1	
**compare_others**	**good to excellent**	0		
	**poor or fair**	1		
**ratehealth**	**good to excellent**	0		
	**poor or fair**	1		
**x1**	**no**	0		
	**yes**	1		
**x2**	**no**	0		
	**yes**	1		
**x3**	**no**	0		
	**yes**	1		
**x4**	**no**	0		
	**yes**	1		
**x5**	**no**	0		
	**yes**	1		

The GENMOD Procedure

Class Level Information				
Class	Value	Design Variables		
x6	no	0		
	yes	1		
x8	no	0		
	yes	1		
x9	no	0		
	yes	1		
x10	no	0		
	yes	1		
x11	no	0		
	yes	1		
x12	no	0		
	yes	1		
x13	no	0		
	yes	1		
x14	no	0		
	yes	1		
upset	do not want to answer	1	0	0
	missing	0	0	0
	no	0	1	0
	yes	0	0	1
hadajob	no	1		
	yes	0		
infected	no	0		
	yes	1		

Fully adjusted models of HARDSHIPS with gender*theme interaction

The GENMOD Procedure

Criteria For Assessing Goodness Of Fit			
Criterion	DF	Value	Value/DF
Deviance	1243	887.9305	0.7143
Scaled Deviance	1243	1293.0000	1.0402
Pearson Chi-Square	1243	887.9305	0.7143
Scaled Pearson X2	1243	1293.0000	1.0402
Log Likelihood		-1591.7154	
Full Log Likelihood		-1591.7154	
AIC (smaller is better)		3285.4308	
AICC (smaller is better)		3289.7048	
BIC (smaller is better)		3548.8316	

Algorithm converged.

Analysis Of Maximum Likelihood Parameter Estimates									
Parameter			DF	Estimate	Standard Error	Wald 95% Confidence Limits		Wald Chi-Square	Pr > ChiSq
Intercept			1	-0.5106	0.1615	-0.8270	-0.1941	10.00	0.0016
gender*x14	Male	yes	1	0.1256	0.8809	-1.6009	1.8521	0.02	0.8866
gender*x14	Other	yes	0	0.0000	0.0000	0.0000	0.0000	.	.
x1	yes		1	-0.0458	0.0633	-0.1698	0.0783	0.52	0.4696
x2	yes		1	-0.0086	0.3196	-0.6349	0.6178	0.00	0.9786
x3	yes		1	-0.1527	0.1069	-0.3622	0.0569	2.04	0.1533
x4	yes		1	0.3973	0.0916	0.2179	0.5768	18.83	<.0001
x5	yes		1	0.1230	0.0794	-0.0326	0.2787	2.40	0.1213
x6	yes		1	-0.0892	0.0807	-0.2473	0.0689	1.22	0.2690
x8	yes		1	0.0008	0.0782	-0.1525	0.1541	0.00	0.9919
x9	yes		1	-0.0097	0.2812	-0.5609	0.5415	0.00	0.9724

Fully adjusted models of HARDSHIPS with gender*theme interaction

The GENMOD Procedure

Analysis Of Maximum Likelihood Parameter Estimates									
Parameter			DF	Estimate	Standard Error	Wald 95% Confidence Limits		Wald Chi-Square	Pr > ChiSq
x10	yes		1	0.0747	0.0864	-0.0948	0.2441	0.75	0.3877
x11	yes		1	0.0964	0.2183	-0.3315	0.5243	0.20	0.6588
x12	yes		1	0.5316	0.2821	-0.0212	1.0845	3.55	0.0595
x13	yes		1	-0.0142	0.1523	-0.3128	0.2843	0.01	0.9255
x14	yes		1	-0.0726	0.2834	-0.6280	0.4829	0.07	0.7979
upset	do not want to answer		1	-0.0399	0.3106	-0.6486	0.5688	0.02	0.8979
upset	no		1	-0.0076	0.0599	-0.1249	0.1097	0.02	0.8990
upset	yes		1	0.0531	0.0954	-0.1340	0.2401	0.31	0.5781
dur			1	0.0008	0.0009	-0.0009	0.0025	0.81	0.3676
support_family			1	-0.0040	0.0010	-0.0059	-0.0021	17.09	<.0001
support_doctor			1	0.0013	0.0010	-0.0006	0.0032	1.88	0.1701
support_fedgovt			1	0.0032	0.0013	0.0007	0.0057	6.31	0.0120
support_city			1	0.0030	0.0014	0.0002	0.0057	4.39	0.0362
support_deptpubhealt			1	-0.0002	0.0013	-0.0028	0.0023	0.03	0.8689
support_faithcommuni			1	0.0009	0.0010	-0.0010	0.0028	0.82	0.3665
support_socialservic			1	0.0059	0.0013	0.0033	0.0085	20.00	<.0001
support_neighbors			1	-0.0010	0.0010	-0.0029	0.0009	1.08	0.2995
support_other			1	0.0030	0.0012	0.0006	0.0053	6.08	0.0136
infected	yes		1	0.3523	0.2336	-0.1056	0.8102	2.27	0.1315
feltunwell	missing		1	-0.4410	0.4196	-1.2634	0.3813	1.11	0.2932
feltunwell	yes		1	0.0514	0.0569	-0.0601	0.1630	0.82	0.3662
compare_others	poor or fair		1	0.1858	0.0936	0.0024	0.3692	3.94	0.0470
ratehealth	poor or fair		1	0.1647	0.1248	-0.0799	0.4092	1.74	0.1869
gender	Male		1	-0.1193	0.0555	-0.2281	-0.0106	4.63	0.0315
gender	Other		1	0.4193	0.2056	0.0165	0.8222	4.16	0.0413

Fully adjusted models of HARDSHIPS with gender*theme interaction

The GENMOD Procedure

Analysis Of Maximum Likelihood Parameter Estimates									
**Parameter**			**DF**	**Estimate**	**Standard Error**	**Wald 95% Confidence Limits**		**Wald Chi-Square**	**Pr > ChiSq**
**race**	black		1	0.1264	0.0955	-0.0608	0.3136	1.75	0.1856
**race**	other race		1	-0.0617	0.0925	-0.2430	0.1195	0.45	0.5044
**age_cat**	35-54		1	0.0038	0.0738	-0.1408	0.1485	0.00	0.9587
**age_cat**	greater than 55		1	-0.1858	0.0795	-0.3417	-0.0299	5.46	0.0195
**inc**	40,000 - $99,999		1	0.1173	0.0586	0.0024	0.2321	4.01	0.0453
**inc**	less than $40,000		1	0.2252	0.0760	0.0762	0.3741	8.78	0.0031
**inc**	missing		1	0.1475	0.1516	-0.1497	0.4447	0.95	0.3308
**edu**	missing		1	-0.1207	0.3265	-0.7605	0.5192	0.14	0.7117
**edu**	no college degree		1	0.2986	0.0698	0.1618	0.4353	18.32	<.0001
**marital_status**	married or living as married		1	0.0734	0.0613	-0.0467	0.1936	1.43	0.2311
**marital_status**	missing		1	0.9391	0.3164	0.3190	1.5592	8.81	0.0030
**marital_status**	widowed, divorced		1	0.0820	0.0856	-0.0857	0.2497	0.92	0.3378
**kids**	missing		1	-0.2573	0.3355	-0.9150	0.4003	0.59	0.4432
**kids**	yes		1	0.1520	0.0687	0.0174	0.2866	4.90	0.0268
**hadajob**	no		1	-0.0089	0.0633	-0.1331	0.1152	0.02	0.8876
**Scale**			1	0.8287	0.0163	0.7974	0.8612		

Note: The scale parameter was estimated by maximum likelihood.

The GENMOD Procedure

Model Information	
Data Set	WORK.SCORES
Distribution	Normal
Link Function	Identity
Dependent Variable	Factor3

Number of Observations Read	1293
Number of Observations Used	1293

Class Level Information				
Class	Value	Design Variables		
gender	Female	0	0	
	Male	1	0	
	Other	0	1	
race	black	1	0	
	other race	0	1	
	white	0	0	
age_cat	35-54	1	0	
	greater than 55	0	1	
	less than 35	0	0	
inc	$100,000 and above	0	0	0
	40,000 - $99,999	1	0	0
	less than $40,000	0	1	0
	missing	0	0	1
edu	college degree or higher	0	0	
	missing	1	0	
	no college degree	0	1	

The GENMOD Procedure

Class Level Information				
**Class**	**Value**	**Design Variables**		
**kids**	**missing**	1	0	
	**no**	0	0	
	**yes**	0	1	
**marital_status**	**married or living as married**	1	0	0
	**missing**	0	1	0
	**single**	0	0	0
	**widowed, divorced**	0	0	1
**feltunwell**	**missing**	1	0	
	**no**	0	0	
	**yes**	0	1	
**compare_others**	**good to excellent**	0		
	**poor or fair**	1		
**ratehealth**	**good to excellent**	0		
	**poor or fair**	1		
**x1**	**no**	0		
	**yes**	1		
**x2**	**no**	0		
	**yes**	1		
**x3**	**no**	0		
	**yes**	1		
**x4**	**no**	0		
	**yes**	1		
**x5**	**no**	0		
	**yes**	1		

The GENMOD Procedure

Class Level Information				
**Class**	**Value**	**Design Variables**		
**x6**	**no**	0		
	**yes**	1		
**x8**	**no**	0		
	**yes**	1		
**x9**	**no**	0		
	**yes**	1		
**x10**	**no**	0		
	**yes**	1		
**x11**	**no**	0		
	**yes**	1		
**x12**	**no**	0		
	**yes**	1		
**x13**	**no**	0		
	**yes**	1		
**x14**	**no**	0		
	**yes**	1		
**upset**	**do not want to answer**	1	0	0
	**missing**	0	0	0
	**no**	0	1	0
	**yes**	0	0	1
**hadajob**	**no**	1		
	**yes**	0		
**infected**	**no**	0		
	**yes**	1		

The GENMOD Procedure

Criteria For Assessing Goodness Of Fit			
**Criterion**	**DF**	**Value**	**Value/DF**
**Deviance**	1242	902.7122	0.7268
**Scaled Deviance**	1242	1293.0000	1.0411
**Pearson Chi-Square**	1242	902.7122	0.7268
**Scaled Pearson X2**	1242	1293.0000	1.0411
**Log Likelihood**		-1602.3894	
**Full Log Likelihood**		-1602.3894	
**AIC (smaller is better)**		3308.7788	
**AICC (smaller is better)**		3313.2239	
**BIC (smaller is better)**		3577.3442	

Algorithm converged.

Analysis Of Maximum Likelihood Parameter Estimates									
**Parameter**			**DF**	**Estimate**	**Standard Error**	**Wald 95% Confidence Limits**		**Wald Chi-Square**	**Pr > ChiSq**
**Intercept**			1	-1.3013	0.1643	-1.6234	-0.9793	62.73	<.0001
**gender*x1**	Male	yes	1	-0.1488	0.1463	-0.4355	0.1379	1.04	0.3090
**gender*x1**	Other	yes	1	-0.3345	0.5444	-1.4015	0.7324	0.38	0.5389
**x1**	yes		1	0.0707	0.0723	-0.0711	0.2125	0.96	0.3284
**x2**	yes		1	0.1549	0.3222	-0.4766	0.7864	0.23	0.6307
**x3**	yes		1	-0.1308	0.1082	-0.3429	0.0812	1.46	0.2265
**x4**	yes		1	0.0085	0.0923	-0.1724	0.1895	0.01	0.9265
**x5**	yes		1	0.2743	0.0801	0.1173	0.4312	11.73	0.0006
**x6**	yes		1	-0.2266	0.0814	-0.3861	-0.0671	7.75	0.0054
**x8**	yes		1	0.1474	0.0789	-0.0072	0.3021	3.49	0.0617
**x9**	yes		1	-0.0634	0.2836	-0.6193	0.4925	0.05	0.8230

The GENMOD Procedure

Analysis Of Maximum Likelihood Parameter Estimates

Parameter			DF	Estimate	Standard Error	Wald 95% Confidence Limits		Wald Chi-Square	Pr > ChiSq
x10	yes		1	-0.0062	0.0873	-0.1773	0.1649	0.01	0.9436
x11	yes		1	0.1588	0.2202	-0.2728	0.5905	0.52	0.4708
x12	yes		1	0.2453	0.2845	-0.3124	0.8030	0.74	0.3886
x13	yes		1	-0.0741	0.1536	-0.3751	0.2269	0.23	0.6295
x14	yes		1	0.0936	0.2709	-0.4373	0.6246	0.12	0.7297
upset	do not want to answer		1	0.0595	0.3141	-0.5561	0.6751	0.04	0.8498
upset	no		1	0.1696	0.0604	0.0513	0.2879	7.89	0.0050
upset	yes		1	0.2101	0.0962	0.0216	0.3987	4.77	0.0290
dur			1	0.0051	0.0009	0.0033	0.0068	32.32	<.0001
support_family			1	0.0011	0.0010	-0.0008	0.0030	1.18	0.2768
support_doctor			1	0.0015	0.0010	-0.0003	0.0034	2.59	0.1078
support_fedgovt			1	0.0000	0.0013	-0.0025	0.0026	0.00	0.9711
support_city			1	0.0032	0.0014	0.0004	0.0060	5.18	0.0229
support_deptpubhealt			1	0.0017	0.0013	-0.0009	0.0043	1.66	0.1978
support_faithcommuni			1	0.0020	0.0010	0.0001	0.0039	4.14	0.0419
support_socialservic			1	0.0026	0.0013	-0.0000	0.0052	3.80	0.0512
support_neighbors			1	-0.0002	0.0010	-0.0022	0.0017	0.06	0.8055
support_other			1	0.0005	0.0012	-0.0019	0.0028	0.14	0.7071
infected	yes		1	0.1016	0.2357	-0.3603	0.5635	0.19	0.6665
feltunwell	missing		1	-0.9260	0.4230	-1.7551	-0.0968	4.79	0.0286
feltunwell	yes		1	0.0462	0.0575	-0.0664	0.1589	0.65	0.4212
compare_others	poor or fair		1	0.1609	0.0949	-0.0251	0.3469	2.87	0.0900
ratehealth	poor or fair		1	0.0450	0.1262	-0.2023	0.2923	0.13	0.7214
gender	Male		1	-0.1384	0.0610	-0.2580	-0.0188	5.14	0.0233
gender	Other		1	0.1651	0.2281	-0.2819	0.6122	0.52	0.4690

The GENMOD Procedure

Analysis Of Maximum Likelihood Parameter Estimates									
Parameter			DF	Estimate	Standard Error	Wald 95% Confidence Limits		Wald Chi-Square	Pr > ChiSq
race	black		1	0.0686	0.0963	-0.1201	0.2573	0.51	0.4763
race	other race		1	-0.0592	0.0935	-0.2424	0.1240	0.40	0.5264
age_cat	35-54		1	0.0835	0.0745	-0.0625	0.2296	1.26	0.2622
age_cat	greater than 55		1	-0.0444	0.0803	-0.2018	0.1131	0.30	0.5809
inc	40,000 - $99,999		1	0.0809	0.0591	-0.0349	0.1968	1.87	0.1711
inc	less than $40,000		1	0.1557	0.0766	0.0056	0.3059	4.13	0.0421
inc	missing		1	0.0560	0.1529	-0.2437	0.3557	0.13	0.7141
edu	missing		1	-0.5159	0.3304	-1.1634	0.1316	2.44	0.1184
edu	no college degree		1	0.0682	0.0704	-0.0697	0.2061	0.94	0.3322
marital_status	married or living as married		1	0.2814	0.0618	0.1602	0.4025	20.71	<.0001
marital_status	missing		1	0.2706	0.3190	-0.3547	0.8959	0.72	0.3964
marital_status	widowed, divorced		1	0.0703	0.0863	-0.0989	0.2395	0.66	0.4155
kids	missing		1	-0.1164	0.3381	-0.7790	0.5462	0.12	0.7307
kids	yes		1	0.0599	0.0693	-0.0759	0.1956	0.75	0.3875
hadajob	no		1	-0.0460	0.0638	-0.1710	0.0791	0.52	0.4713
Scale			1	0.8356	0.0164	0.8040	0.8684		

Note: The scale parameter was estimated by maximum likelihood.

The GENMOD Procedure

Model Information	
Data Set	WORK.SCORES
Distribution	Normal
Link Function	Identity
Dependent Variable	Factor3

Number of Observations Read	1293
Number of Observations Used	1293

Class Level Information				
Class	Value	Design Variables		
gender	Female	0	0	
	Male	1	0	
	Other	0	1	
race	black	1	0	
	other race	0	1	
	white	0	0	
age_cat	35-54	1	0	
	greater than 55	0	1	
	less than 35	0	0	
inc	$100,000 and above	0	0	0
	40,000 - $99,999	1	0	0
	less than $40,000	0	1	0
	missing	0	0	1
edu	college degree or higher	0	0	
	missing	1	0	
	no college degree	0	1	

Fully adjusted models of FEAR OF INFECTION with gender*theme interaction

The GENMOD Procedure

Class Level Information				
Class	Value	Design Variables		
kids	missing	1	0	
	no	0	0	
	yes	0	1	
marital_status	married or living as married	1	0	0
	missing	0	1	0
	single	0	0	0
	widowed, divorced	0	0	1
feltunwell	missing	1	0	
	no	0	0	
	yes	0	1	
compare_others	good to excellent	0		
	poor or fair	1		
ratehealth	good to excellent	0		
	poor or fair	1		
x1	no	0		
	yes	1		
x2	no	0		
	yes	1		
x3	no	0		
	yes	1		
x4	no	0		
	yes	1		
x5	no	0		
	yes	1		

The GENMOD Procedure

Class Level Information				
Class	Value	Design Variables		
x6	no	0		
	yes	1		
x8	no	0		
	yes	1		
x9	no	0		
	yes	1		
x10	no	0		
	yes	1		
x11	no	0		
	yes	1		
x12	no	0		
	yes	1		
x13	no	0		
	yes	1		
x14	no	0		
	yes	1		
upset	do not want to answer	1	0	0
	missing	0	0	0
	no	0	1	0
	yes	0	0	1
hadajob	no	1		
	yes	0		
infected	no	0		
	yes	1		

The GENMOD Procedure

Criteria For Assessing Goodness Of Fit			
**Criterion**	**DF**	**Value**	**Value/DF**
**Deviance**	1243	903.2474	0.7267
**Scaled Deviance**	1243	1293.0000	1.0402
**Pearson Chi-Square**	1243	903.2474	0.7267
**Scaled Pearson X2**	1243	1293.0000	1.0402
**Log Likelihood**		-1602.7725	
**Full Log Likelihood**		-1602.7725	
**AIC (smaller is better)**		3307.5450	
**AICC (smaller is better)**		3311.8190	
**BIC (smaller is better)**		3570.9458	

Algorithm converged.

Analysis Of Maximum Likelihood Parameter Estimates									
**Parameter**			**DF**	**Estimate**	**Standard Error**	**Wald 95% Confidence Limits**		**Wald Chi-Square**	**Pr > ChiSq**
**Intercept**			1	-1.2717	0.1629	-1.5910	-0.9523	60.91	<.0001
**gender*x2**	Male	yes	1	0.5342	0.7095	-0.8563	1.9247	0.57	0.4514
**gender*x2**	Other	yes	0	0.0000	0.0000	0.0000	0.0000	.	.
**x1**	yes		1	0.0324	0.0638	-0.0927	0.1575	0.26	0.6116
**x2**	yes		1	0.0008	0.3804	-0.7447	0.7463	0.00	0.9984
**x3**	yes		1	-0.1335	0.1080	-0.3452	0.0782	1.53	0.2165
**x4**	yes		1	0.0058	0.0923	-0.1751	0.1868	0.00	0.9497
**x5**	yes		1	0.2733	0.0801	0.1164	0.4303	11.65	0.0006
**x6**	yes		1	-0.2271	0.0814	-0.3866	-0.0676	7.79	0.0052
**x8**	yes		1	0.1434	0.0789	-0.0112	0.2980	3.30	0.0691
**x9**	yes		1	-0.0699	0.2836	-0.6258	0.4860	0.06	0.8053

Fully adjusted models of FEAR OF INFECTION with gender*theme interaction

The GENMOD Procedure

Analysis Of Maximum Likelihood Parameter Estimates									
Parameter			DF	Estimate	Standard Error	Wald 95% Confidence Limits		Wald Chi-Square	Pr > ChiSq
x10	yes		1	-0.0091	0.0872	-0.1800	0.1618	0.01	0.9167
x11	yes		1	0.1535	0.2202	-0.2781	0.5851	0.49	0.4858
x12	yes		1	0.2497	0.2845	-0.3079	0.8073	0.77	0.3802
x13	yes		1	-0.0735	0.1536	-0.3746	0.2277	0.23	0.6326
x14	yes		1	0.0979	0.2709	-0.4331	0.6290	0.13	0.7177
upset	do not want to answer		1	0.0746	0.3132	-0.5393	0.6886	0.06	0.8118
upset	no		1	0.1672	0.0604	0.0489	0.2856	7.67	0.0056
upset	yes		1	0.2090	0.0962	0.0204	0.3977	4.72	0.0299
dur			1	0.0051	0.0009	0.0033	0.0069	32.53	<.0001
support_family			1	0.0010	0.0010	-0.0009	0.0029	1.03	0.3104
support_doctor			1	0.0016	0.0010	-0.0003	0.0034	2.67	0.1026
support_fedgovt			1	0.0000	0.0013	-0.0025	0.0025	0.00	0.9940
support_city			1	0.0032	0.0014	0.0004	0.0060	5.15	0.0232
support_deptpubhealt			1	0.0017	0.0013	-0.0009	0.0042	1.60	0.2064
support_faithcommuni			1	0.0020	0.0010	0.0000	0.0039	4.04	0.0445
support_socialservic			1	0.0026	0.0013	0.0000	0.0053	3.91	0.0479
support_neighbors			1	-0.0002	0.0010	-0.0022	0.0017	0.05	0.8212
support_other			1	0.0004	0.0012	-0.0020	0.0028	0.10	0.7546
infected	yes		1	0.1042	0.2356	-0.3575	0.5660	0.20	0.6582
feltunwell	missing		1	-0.9248	0.4232	-1.7542	-0.0955	4.78	0.0288
feltunwell	yes		1	0.0481	0.0574	-0.0645	0.1606	0.70	0.4026
compare_others	poor or fair		1	0.1531	0.0944	-0.0318	0.3381	2.63	0.1046
ratehealth	poor or fair		1	0.0551	0.1258	-0.1915	0.3017	0.19	0.6616
gender	Male		1	-0.1668	0.0560	-0.2766	-0.0570	8.86	0.0029
gender	Other		1	0.1005	0.2073	-0.3059	0.5069	0.23	0.6279

The GENMOD Procedure

Analysis Of Maximum Likelihood Parameter Estimates									
Parameter			DF	Estimate	Standard Error	Wald 95% Confidence Limits		Wald Chi-Square	Pr > ChiSq
race	black		1	0.0668	0.0964	-0.1221	0.2556	0.48	0.4884
race	other race		1	-0.0639	0.0933	-0.2468	0.1190	0.47	0.4933
age_cat	35-54		1	0.0766	0.0746	-0.0696	0.2227	1.05	0.3046
age_cat	greater than 55		1	-0.0530	0.0803	-0.2103	0.1044	0.44	0.5094
inc	40,000 - $99,999		1	0.0821	0.0591	-0.0336	0.1979	1.93	0.1644
inc	less than $40,000		1	0.1541	0.0766	0.0039	0.3044	4.04	0.0443
inc	missing		1	0.0528	0.1529	-0.2469	0.3525	0.12	0.7299
edu	missing		1	-0.5447	0.3293	-1.1900	0.1006	2.74	0.0981
edu	no college degree		1	0.0685	0.0704	-0.0695	0.2065	0.95	0.3304
marital_status	married or living as married		1	0.2813	0.0618	0.1601	0.4025	20.70	<.0001
marital_status	missing		1	0.2683	0.3191	-0.3571	0.8938	0.71	0.4005
marital_status	widowed, divorced		1	0.0650	0.0863	-0.1041	0.2342	0.57	0.4511
kids	missing		1	-0.1264	0.3380	-0.7890	0.5361	0.14	0.7084
kids	yes		1	0.0570	0.0692	-0.0787	0.1928	0.68	0.4100
hadajob	no		1	-0.0471	0.0638	-0.1721	0.0780	0.54	0.4609
Scale			1	0.8358	0.0164	0.8042	0.8686		

Note: The scale parameter was estimated by maximum likelihood.

Fully adjusted models of FEAR OF INFECTION with gender*theme interaction

The GENMOD Procedure

Model Information	
Data Set	WORK.SCORES
Distribution	Normal
Link Function	Identity
Dependent Variable	Factor3

Number of Observations Read	1293
Number of Observations Used	1293

Class Level Information				
Class	Value	Design Variables		
gender	Female	0	0	
	Male	1	0	
	Other	0	1	
race	black	1	0	
	other race	0	1	
	white	0	0	
age_cat	35-54	1	0	
	greater than 55	0	1	
	less than 35	0	0	
inc	$100,000 and above	0	0	0
	40,000 - $99,999	1	0	0
	less than $40,000	0	1	0
	missing	0	0	1
edu	college degree or higher	0	0	
	missing	1	0	
	no college degree	0	1	

The GENMOD Procedure

Class Level Information				
Class	Value	Design Variables		
kids	missing	1	0	
	no	0	0	
	yes	0	1	
marital_status	married or living as married	1	0	0
	missing	0	1	0
	single	0	0	0
	widowed, divorced	0	0	1
feltunwell	missing	1	0	
	no	0	0	
	yes	0	1	
compare_others	good to excellent	0		
	poor or fair	1		
ratehealth	good to excellent	0		
	poor or fair	1		
x1	no	0		
	yes	1		
x2	no	0		
	yes	1		
x3	no	0		
	yes	1		
x4	no	0		
	yes	1		
x5	no	0		
	yes	1		

The GENMOD Procedure

Class Level Information				
**Class**	**Value**	**Design Variables**		
**x6**	**no**	0		
	**yes**	1		
**x8**	**no**	0		
	**yes**	1		
**x9**	**no**	0		
	**yes**	1		
**x10**	**no**	0		
	**yes**	1		
**x11**	**no**	0		
	**yes**	1		
**x12**	**no**	0		
	**yes**	1		
**x13**	**no**	0		
	**yes**	1		
**x14**	**no**	0		
	**yes**	1		
**upset**	**do not want to answer**	1	0	0
	**missing**	0	0	0
	**no**	0	1	0
	**yes**	0	0	1
**hadajob**	**no**	1		
	**yes**	0		
**infected**	**no**	0		
	**yes**	1		

The GENMOD Procedure

Criteria For Assessing Goodness Of Fit			
**Criterion**	**DF**	**Value**	**Value/DF**
**Deviance**	1242	902.9797	0.7270
**Scaled Deviance**	1242	1293.0000	1.0411
**Pearson Chi-Square**	1242	902.9797	0.7270
**Scaled Pearson X2**	1242	1293.0000	1.0411
**Log Likelihood**		-1602.5809	
**Full Log Likelihood**		-1602.5809	
**AIC (smaller is better)**		3309.1617	
**AICC (smaller is better)**		3313.6069	
**BIC (smaller is better)**		3577.7272	

Algorithm converged.

Analysis Of Maximum Likelihood Parameter Estimates									
**Parameter**			**DF**	**Estimate**	**Standard Error**	**Wald 95% Confidence Limits**		**Wald Chi-Square**	**Pr > ChiSq**
**Intercept**			1	-1.2815	0.1634	-1.6018	-0.9611	61.48	<.0001
**gender*x3**	Male	yes	1	-0.2357	0.2418	-0.7095	0.2382	0.95	0.3296
**gender*x3**	Other	yes	1	-0.0516	0.8812	-1.7788	1.6756	0.00	0.9533
**x1**	yes		1	0.0295	0.0640	-0.0959	0.1550	0.21	0.6447
**x2**	yes		1	0.1800	0.3235	-0.4540	0.8139	0.31	0.5780
**x3**	yes		1	-0.0851	0.1171	-0.3146	0.1444	0.53	0.4672
**x4**	yes		1	0.0050	0.0923	-0.1759	0.1859	0.00	0.9570
**x5**	yes		1	0.2748	0.0801	0.1178	0.4317	11.77	0.0006
**x6**	yes		1	-0.2306	0.0814	-0.3902	-0.0711	8.03	0.0046
**x8**	yes		1	0.1458	0.0789	-0.0088	0.3004	3.41	0.0646
**x9**	yes		1	-0.0672	0.2836	-0.6230	0.4887	0.06	0.8127

Fully adjusted models of FEAR OF INFECTION with gender*theme interaction

The GENMOD Procedure

Analysis Of Maximum Likelihood Parameter Estimates									
Parameter			DF	Estimate	Standard Error	Wald 95% Confidence Limits		Wald Chi-Square	Pr > ChiSq
**x10**	yes		1	-0.0133	0.0872	-0.1842	0.1576	0.02	0.8788
**x11**	yes		1	0.1441	0.2203	-0.2878	0.5759	0.43	0.5132
**x12**	yes		1	0.2492	0.2845	-0.3084	0.8067	0.77	0.3811
**x13**	yes		1	-0.0764	0.1536	-0.3776	0.2247	0.25	0.6190
**x14**	yes		1	0.1020	0.2709	-0.4290	0.6329	0.14	0.7067
**upset**	do not want to answer		1	0.0802	0.3135	-0.5342	0.6946	0.07	0.7980
**upset**	no		1	0.1675	0.0604	0.0492	0.2859	7.70	0.0055
**upset**	yes		1	0.2134	0.0963	0.0247	0.4022	4.91	0.0267
**dur**			1	0.0051	0.0009	0.0033	0.0068	32.10	<.0001
**support_family**			1	0.0010	0.0010	-0.0009	0.0029	1.05	0.3045
**support_doctor**			1	0.0016	0.0010	-0.0003	0.0034	2.66	0.1032
**support_fedgovt**			1	0.0001	0.0013	-0.0024	0.0026	0.00	0.9438
**support_city**			1	0.0032	0.0014	0.0004	0.0060	5.14	0.0234
**support_deptpubhealt**			1	0.0017	0.0013	-0.0009	0.0043	1.62	0.2027
**support_faithcommuni**			1	0.0020	0.0010	0.0000	0.0039	4.00	0.0455
**support_socialservic**			1	0.0027	0.0013	0.0001	0.0053	4.02	0.0449
**support_neighbors**			1	-0.0002	0.0010	-0.0022	0.0017	0.05	0.8168
**support_other**			1	0.0004	0.0012	-0.0020	0.0028	0.12	0.7281
**infected**	yes		1	0.1056	0.2356	-0.3562	0.5674	0.20	0.6541
**feltunwell**	missing		1	-0.9231	0.4231	-1.7524	-0.0938	4.76	0.0291
**feltunwell**	yes		1	0.0463	0.0575	-0.0663	0.1589	0.65	0.4200
**compare_others**	poor or fair		1	0.1499	0.0949	-0.0362	0.3360	2.49	0.1143
**ratehealth**	poor or fair		1	0.0556	0.1261	-0.1915	0.3026	0.19	0.6592
**gender**	Male		1	-0.1507	0.0574	-0.2633	-0.0382	6.89	0.0087
**gender**	Other		1	0.1077	0.2142	-0.3122	0.5275	0.25	0.6152

The GENMOD Procedure

Analysis Of Maximum Likelihood Parameter Estimates									
Parameter			DF	Estimate	Standard Error	Wald 95% Confidence Limits		Wald Chi-Square	Pr > ChiSq
race	black		1	0.0656	0.0964	-0.1233	0.2544	0.46	0.4962
race	other race		1	-0.0699	0.0934	-0.2529	0.1132	0.56	0.4544
age_cat	35-54		1	0.0805	0.0745	-0.0654	0.2264	1.17	0.2796
age_cat	greater than 55		1	-0.0505	0.0803	-0.2078	0.1068	0.40	0.5291
inc	40,000 - $99,999		1	0.0839	0.0591	-0.0319	0.1998	2.02	0.1557
inc	less than $40,000		1	0.1544	0.0766	0.0042	0.3046	4.06	0.0439
inc	missing		1	0.0592	0.1530	-0.2407	0.3591	0.15	0.6988
edu	missing		1	-0.5472	0.3292	-1.1925	0.0981	2.76	0.0965
edu	no college degree		1	0.0653	0.0704	-0.0726	0.2032	0.86	0.3532
marital_status	married or living as married		1	0.2804	0.0618	0.1592	0.4016	20.56	<.0001
marital_status	missing		1	0.2677	0.3191	-0.3577	0.8930	0.70	0.4015
marital_status	widowed, divorced		1	0.0692	0.0863	-0.1000	0.2384	0.64	0.4227
kids	missing		1	-0.1389	0.3382	-0.8018	0.5239	0.17	0.6812
kids	yes		1	0.0567	0.0692	-0.0790	0.1925	0.67	0.4125
hadajob	no		1	-0.0479	0.0638	-0.1729	0.0772	0.56	0.4533
Scale			1	0.8357	0.0164	0.8041	0.8685		

Note: The scale parameter was estimated by maximum likelihood.

The GENMOD Procedure

Model Information	
Data Set	WORK.SCORES
Distribution	Normal
Link Function	Identity
Dependent Variable	Factor3

Number of Observations Read	1293
Number of Observations Used	1293

Class Level Information				
Class	Value	Design Variables		
gender	Female	0	0	
	Male	1	0	
	Other	0	1	
race	black	1	0	
	other race	0	1	
	white	0	0	
age_cat	35-54	1	0	
	greater than 55	0	1	
	less than 35	0	0	
inc	$100,000 and above	0	0	0
	40,000 - $99,999	1	0	0
	less than $40,000	0	1	0
	missing	0	0	1
edu	college degree or higher	0	0	
	missing	1	0	
	no college degree	0	1	

The GENMOD Procedure

Class Level Information				
**Class**	**Value**	**Design Variables**		
**kids**	**missing**	1	0	
	**no**	0	0	
	**yes**	0	1	
**marital_status**	**married or living as married**	1	0	0
	**missing**	0	1	0
	**single**	0	0	0
	**widowed, divorced**	0	0	1
**feltunwell**	**missing**	1	0	
	**no**	0	0	
	**yes**	0	1	
**compare_others**	**good to excellent**	0		
	**poor or fair**	1		
**ratehealth**	**good to excellent**	0		
	**poor or fair**	1		
**x1**	**no**	0		
	**yes**	1		
**x2**	**no**	0		
	**yes**	1		
**x3**	**no**	0		
	**yes**	1		
**x4**	**no**	0		
	**yes**	1		
**x5**	**no**	0		
	**yes**	1		

The GENMOD Procedure

Class Level Information				
Class	Value	Design Variables		
x6	no	0		
	yes	1		
x8	no	0		
	yes	1		
x9	no	0		
	yes	1		
x10	no	0		
	yes	1		
x11	no	0		
	yes	1		
x12	no	0		
	yes	1		
x13	no	0		
	yes	1		
x14	no	0		
	yes	1		
upset	do not want to answer	1	0	0
	missing	0	0	0
	no	0	1	0
	yes	0	0	1
hadajob	no	1		
	yes	0		
infected	no	0		
	yes	1		

The GENMOD Procedure

Criteria For Assessing Goodness Of Fit			
**Criterion**	**DF**	**Value**	**Value/DF**
**Deviance**	1243	900.9363	0.7248
**Scaled Deviance**	1243	1293.0000	1.0402
**Pearson Chi-Square**	1243	900.9363	0.7248
**Scaled Pearson X2**	1243	1293.0000	1.0402
**Log Likelihood**		-1601.1162	
**Full Log Likelihood**		-1601.1162	
**AIC (smaller is better)**		3304.2325	
**AICC (smaller is better)**		3308.5065	
**BIC (smaller is better)**		3567.6332	

Algorithm converged.

Analysis Of Maximum Likelihood Parameter Estimates									
**Parameter**			**DF**	**Estimate**	**Standard Error**	**Wald 95% Confidence Limits**		**Wald Chi-Square**	**Pr > ChiSq**
**Intercept**			1	-1.2896	0.1628	-1.6086	-0.9706	62.76	<.0001
**gender*x4**	Male	yes	1	-0.4157	0.2109	-0.8291	-0.0024	3.89	0.0487
**gender*x4**	Other	yes	0	0.0000	0.0000	0.0000	0.0000	.	.
**x1**	yes		1	0.0351	0.0637	-0.0898	0.1601	0.30	0.5817
**x2**	yes		1	0.1571	0.3219	-0.4738	0.7880	0.24	0.6256
**x3**	yes		1	-0.1293	0.1077	-0.3403	0.0818	1.44	0.2299
**x4**	yes		1	0.1090	0.1060	-0.0987	0.3168	1.06	0.3038
**x5**	yes		1	0.2738	0.0800	0.1170	0.4306	11.72	0.0006
**x6**	yes		1	-0.2290	0.0813	-0.3882	-0.0697	7.94	0.0048
**x8**	yes		1	0.1523	0.0789	-0.0023	0.3068	3.73	0.0536
**x9**	yes		1	-0.0637	0.2833	-0.6190	0.4915	0.05	0.8220

The GENMOD Procedure

Analysis Of Maximum Likelihood Parameter Estimates

Parameter			DF	Estimate	Standard Error	Wald 95% Confidence Limits		Wald Chi-Square	Pr > ChiSq
**x10**	yes		1	-0.0139	0.0871	-0.1846	0.1568	0.03	0.8733
**x11**	yes		1	0.1721	0.2202	-0.2594	0.6036	0.61	0.4344
**x12**	yes		1	0.2483	0.2841	-0.3086	0.8051	0.76	0.3823
**x13**	yes		1	-0.0739	0.1534	-0.3746	0.2268	0.23	0.6301
**x14**	yes		1	0.0855	0.2707	-0.4450	0.6160	0.10	0.7521
**upset**	do not want to answer		1	0.0805	0.3128	-0.5327	0.6937	0.07	0.7969
**upset**	no		1	0.1677	0.0603	0.0495	0.2859	7.73	0.0054
**upset**	yes		1	0.2090	0.0961	0.0206	0.3974	4.73	0.0297
**dur**			1	0.0051	0.0009	0.0034	0.0069	32.68	<.0001
**support_family**			1	0.0010	0.0010	-0.0009	0.0029	0.97	0.3250
**support_doctor**			1	0.0017	0.0010	-0.0002	0.0036	3.21	0.0730
**support_fedgovt**			1	0.0001	0.0013	-0.0024	0.0026	0.01	0.9348
**support_city**			1	0.0032	0.0014	0.0004	0.0060	5.00	0.0253
**support_deptpubhealt**			1	0.0018	0.0013	-0.0008	0.0044	1.90	0.1681
**support_faithcommuni**			1	0.0019	0.0010	-0.0000	0.0038	3.65	0.0559
**support_socialservic**			1	0.0026	0.0013	-0.0000	0.0052	3.71	0.0540
**support_neighbors**			1	-0.0002	0.0010	-0.0022	0.0017	0.05	0.8282
**support_other**			1	0.0004	0.0012	-0.0020	0.0027	0.09	0.7599
**infected**	yes		1	0.0861	0.2355	-0.3754	0.5477	0.13	0.7145
**feltunwell**	missing		1	-0.9187	0.4226	-1.7471	-0.0904	4.73	0.0297
**feltunwell**	yes		1	0.0492	0.0573	-0.0631	0.1616	0.74	0.3905
**compare_others**	poor or fair		1	0.1543	0.0942	-0.0304	0.3390	2.68	0.1016
**ratehealth**	poor or fair		1	0.0522	0.1257	-0.1942	0.2985	0.17	0.6781
**gender**	Male		1	-0.1334	0.0579	-0.2469	-0.0200	5.31	0.0212
**gender**	Other		1	0.1092	0.2071	-0.2967	0.5150	0.28	0.5981

The GENMOD Procedure

Analysis Of Maximum Likelihood Parameter Estimates									
Parameter			DF	Estimate	Standard Error	Wald 95% Confidence Limits		Wald Chi-Square	Pr > ChiSq
**race**	black		1	0.0659	0.0962	-0.1227	0.2545	0.47	0.4933
**race**	other race		1	-0.0613	0.0932	-0.2439	0.1213	0.43	0.5107
**age_cat**	35-54		1	0.0826	0.0744	-0.0631	0.2284	1.23	0.2665
**age_cat**	greater than 55		1	-0.0528	0.0801	-0.2098	0.1042	0.43	0.5097
**inc**	40,000 - $99,999		1	0.0807	0.0590	-0.0350	0.1963	1.87	0.1716
**inc**	less than $40,000		1	0.1519	0.0766	0.0018	0.3019	3.94	0.0473
**inc**	missing		1	0.0507	0.1527	-0.2486	0.3501	0.11	0.7397
**edu**	missing		1	-0.5509	0.3288	-1.1954	0.0936	2.81	0.0939
**edu**	no college degree		1	0.0693	0.0703	-0.0684	0.2071	0.97	0.3238
**marital_status**	married or living as married		1	0.2823	0.0618	0.1612	0.4033	20.89	<.0001
**marital_status**	missing		1	0.2630	0.3187	-0.3617	0.8877	0.68	0.4092
**marital_status**	widowed, divorced		1	0.0761	0.0863	-0.0930	0.2453	0.78	0.3778
**kids**	missing		1	-0.1301	0.3376	-0.7918	0.5315	0.15	0.6999
**kids**	yes		1	0.0552	0.0692	-0.0804	0.1907	0.64	0.4249
**hadajob**	no		1	-0.0485	0.0637	-0.1734	0.0764	0.58	0.4464
**Scale**			1	0.8347	0.0164	0.8032	0.8675		

Note: The scale parameter was estimated by maximum likelihood.

The MEANS Procedure

Analysis Variable : Factor3							
Gender	lost income: self or family	N Obs	N	Mean	Std Dev	Minimum	Maximum
Female	no	879	879	0.0441331	0.9130179	-2.1672663	1.8860752
	yes	70	70	0.1391876	0.9087365	-1.6488815	1.8193298
Male	no	303	303	-0.1364429	0.8728880	-2.1478807	1.8420127
	yes	23	23	-0.3394604	0.8765375	-1.4251553	1.1997917
Other	no	18	18	0.0340897	1.0455503	-1.9281957	1.6937479

The GENMOD Procedure

Model Information	
**Data Set**	WORK.SCORES
**Distribution**	Normal
**Link Function**	Identity
**Dependent Variable**	Factor3

**Number of Observations Read**	1293
**Number of Observations Used**	1293

Class Level Information				
**Class**	**Value**	**Design Variables**		
**gender**	**Female**	0	0	
	**Male**	1	0	
	**Other**	0	1	
**race**	**black**	1	0	
	**other race**	0	1	
	**white**	0	0	
**age_cat**	**35-54**	1	0	
	**greater than 55**	0	1	
	**less than 35**	0	0	
**inc**	**$100,000 and above**	0	0	0
	**40,000 - $99,999**	1	0	0
	**less than $40,000**	0	1	0
	**missing**	0	0	1
**edu**	**college degree or higher**	0	0	
	**missing**	1	0	
	**no college degree**	0	1	

The GENMOD Procedure

Class Level Information				
**Class**	**Value**	**Design Variables**		
**kids**	**missing**	1	0	
	**no**	0	0	
	**yes**	0	1	
**marital_status**	**married or living as married**	1	0	0
	**missing**	0	1	0
	**single**	0	0	0
	**widowed, divorced**	0	0	1
**feltunwell**	**missing**	1	0	
	**no**	0	0	
	**yes**	0	1	
**compare_others**	**good to excellent**	0		
	**poor or fair**	1		
**ratehealth**	**good to excellent**	0		
	**poor or fair**	1		
**x1**	**no**	0		
	**yes**	1		
**x2**	**no**	0		
	**yes**	1		
**x3**	**no**	0		
	**yes**	1		
**x4**	**no**	0		
	**yes**	1		
**x5**	**no**	0		
	**yes**	1		

The GENMOD Procedure

Class Level Information				
Class	Value	Design Variables		
x6	no	0		
	yes	1		
x8	no	0		
	yes	1		
x9	no	0		
	yes	1		
x10	no	0		
	yes	1		
x11	no	0		
	yes	1		
x12	no	0		
	yes	1		
x13	no	0		
	yes	1		
x14	no	0		
	yes	1		
upset	do not want to answer	1	0	0
	missing	0	0	0
	no	0	1	0
	yes	0	0	1
hadajob	no	1		
	yes	0		
infected	no	0		
	yes	1		

Fully adjusted models of FEAR OF INFECTION with gender*theme interaction

The GENMOD Procedure

Criteria For Assessing Goodness Of Fit			
**Criterion**	**DF**	**Value**	**Value/DF**
**Deviance**	1242	901.3445	0.7257
**Scaled Deviance**	1242	1293.0000	1.0411
**Pearson Chi-Square**	1242	901.3445	0.7257
**Scaled Pearson X2**	1242	1293.0000	1.0411
**Log Likelihood**		-1601.4091	
**Full Log Likelihood**		-1601.4091	
**AIC (smaller is better)**		3306.8182	
**AICC (smaller is better)**		3311.2633	
**BIC (smaller is better)**		3575.3836	

Algorithm converged.

Analysis Of Maximum Likelihood Parameter Estimates									
**Parameter**			**DF**	**Estimate**	**Standard Error**	**Wald 95% Confidence Limits**		**Wald Chi-Square**	**Pr > ChiSq**
**Intercept**			1	-1.2710	0.1628	-1.5900	-0.9520	60.98	<.0001
**gender*x5**	Male	yes	1	0.3346	0.1876	-0.0331	0.7023	3.18	0.0745
**gender*x5**	Other	yes	1	0.3650	0.8702	-1.3406	2.0707	0.18	0.6749
**x1**	yes		1	0.0324	0.0638	-0.0925	0.1574	0.26	0.6111
**x2**	yes		1	0.1568	0.3220	-0.4743	0.7879	0.24	0.6263
**x3**	yes		1	-0.1275	0.1077	-0.3386	0.0836	1.40	0.2365
**x4**	yes		1	0.0061	0.0922	-0.1746	0.1869	0.00	0.9469
**x5**	yes		1	0.1940	0.0912	0.0152	0.3728	4.52	0.0335
**x6**	yes		1	-0.2261	0.0813	-0.3854	-0.0668	7.74	0.0054
**x8**	yes		1	0.1488	0.0788	-0.0057	0.3034	3.56	0.0590
**x9**	yes		1	-0.0553	0.2835	-0.6109	0.5002	0.04	0.8453

The GENMOD Procedure

Analysis Of Maximum Likelihood Parameter Estimates									
Parameter			DF	Estimate	Standard Error	Wald 95% Confidence Limits		Wald Chi-Square	Pr > ChiSq
x10	yes		1	-0.0018	0.0872	-0.1728	0.1692	0.00	0.9836
x11	yes		1	0.1424	0.2200	-0.2889	0.5737	0.42	0.5176
x12	yes		1	0.2692	0.2844	-0.2882	0.8267	0.90	0.3439
x13	yes		1	-0.0708	0.1535	-0.3716	0.2300	0.21	0.6445
x14	yes		1	0.1021	0.2707	-0.4283	0.6326	0.14	0.7059
upset	do not want to answer		1	0.0716	0.3133	-0.5424	0.6856	0.05	0.8191
upset	no		1	0.1667	0.0604	0.0482	0.2851	7.61	0.0058
upset	yes		1	0.2022	0.0963	0.0134	0.3910	4.40	0.0358
dur			1	0.0052	0.0009	0.0034	0.0069	33.15	<.0001
support_family			1	0.0010	0.0010	-0.0009	0.0029	0.99	0.3198
support_doctor			1	0.0017	0.0010	-0.0002	0.0036	3.07	0.0796
support_fedgovt			1	0.0001	0.0013	-0.0024	0.0026	0.00	0.9603
support_city			1	0.0032	0.0014	0.0005	0.0060	5.22	0.0223
support_deptpubhealt			1	0.0015	0.0013	-0.0010	0.0041	1.38	0.2398
support_faithcommuni			1	0.0019	0.0010	-0.0000	0.0038	3.80	0.0513
support_socialservic			1	0.0027	0.0013	0.0000	0.0053	3.97	0.0465
support_neighbors			1	-0.0002	0.0010	-0.0021	0.0017	0.04	0.8396
support_other			1	0.0004	0.0012	-0.0020	0.0028	0.10	0.7488
infected	yes		1	0.1130	0.2354	-0.3484	0.5744	0.23	0.6312
feltunwell	missing		1	-0.9223	0.4227	-1.7508	-0.0938	4.76	0.0291
feltunwell	yes		1	0.0508	0.0574	-0.0616	0.1633	0.79	0.3756
compare_others	poor or fair		1	0.1499	0.0943	-0.0350	0.3348	2.53	0.1120
ratehealth	poor or fair		1	0.0585	0.1257	-0.1880	0.3049	0.22	0.6419
gender	Male		1	-0.1944	0.0584	-0.3089	-0.0799	11.08	0.0009
gender	Other		1	0.0779	0.2133	-0.3401	0.4960	0.13	0.7149

The GENMOD Procedure

Analysis Of Maximum Likelihood Parameter Estimates

Parameter			DF	Estimate	Standard Error	Wald 95% Confidence Limits		Wald Chi-Square	Pr > ChiSq
race	black		1	0.0687	0.0962	-0.1199	0.2572	0.51	0.4755
race	other race		1	-0.0611	0.0932	-0.2437	0.1216	0.43	0.5124
age_cat	35-54		1	0.0793	0.0744	-0.0665	0.2250	1.14	0.2866
age_cat	greater than 55		1	-0.0496	0.0801	-0.2067	0.1074	0.38	0.5357
inc	40,000 - $99,999		1	0.0800	0.0590	-0.0357	0.1956	1.83	0.1756
inc	less than $40,000		1	0.1532	0.0766	0.0031	0.3033	4.00	0.0454
inc	missing		1	0.0495	0.1528	-0.2500	0.3489	0.10	0.7461
edu	missing		1	-0.5401	0.3289	-1.1848	0.1047	2.70	0.1006
edu	no college degree		1	0.0668	0.0703	-0.0709	0.2046	0.90	0.3418
marital_status	married or living as married		1	0.2824	0.0618	0.1613	0.4035	20.90	<.0001
marital_status	missing		1	0.2548	0.3189	-0.3702	0.8798	0.64	0.4243
marital_status	widowed, divorced		1	0.0648	0.0862	-0.1042	0.2337	0.56	0.4524
kids	missing		1	-0.1164	0.3377	-0.7784	0.5455	0.12	0.7303
kids	yes		1	0.0548	0.0692	-0.0808	0.1904	0.63	0.4284
hadajob	no		1	-0.0481	0.0638	-0.1731	0.0769	0.57	0.4509
Scale			1	0.8349	0.0164	0.8034	0.8677		

Note: The scale parameter was estimated by maximum likelihood.

The MEANS Procedure

Analysis Variable : Factor3							
Gender	unavoidable proximity to strangers/fear infection	N Obs	N	Mean	Std Dev	Minimum	Maximum
Female	no	850	850	0.0287384	0.9084809	-2.1672663	1.8860752
	yes	99	99	0.2435202	0.9294841	-1.7768594	1.8584198
Male	no	297	297	-0.1994672	0.8559895	-2.1478807	1.8420127
	yes	29	29	0.3479991	0.9079963	-1.6332410	1.7673727
Other	no	17	17	0.0215857	1.0763404	-1.9281957	1.6937479
	yes	1	1	0.2466571	.	0.2466571	0.2466571

The GENMOD Procedure

Model Information	
Data Set	WORK.SCORES
Distribution	Normal
Link Function	Identity
Dependent Variable	Factor3

Number of Observations Read	1293
Number of Observations Used	1293

Class Level Information				
Class	Value	Design Variables		
gender	Female	0	0	
	Male	1	0	
	Other	0	1	
race	black	1	0	
	other race	0	1	
	white	0	0	
age_cat	35-54	1	0	
	greater than 55	0	1	
	less than 35	0	0	
inc	$100,000 and above	0	0	0
	40,000 - $99,999	1	0	0
	less than $40,000	0	1	0
	missing	0	0	1
edu	college degree or higher	0	0	
	missing	1	0	
	no college degree	0	1	

The GENMOD Procedure

Class Level Information				
Class	Value	Design Variables		
kids	missing	1	0	
	no	0	0	
	yes	0	1	
marital_status	married or living as married	1	0	0
	missing	0	1	0
	single	0	0	0
	widowed, divorced	0	0	1
feltunwell	missing	1	0	
	no	0	0	
	yes	0	1	
compare_others	good to excellent	0		
	poor or fair	1		
ratehealth	good to excellent	0		
	poor or fair	1		
x1	no	0		
	yes	1		
x2	no	0		
	yes	1		
x3	no	0		
	yes	1		
x4	no	0		
	yes	1		
x5	no	0		
	yes	1		

The GENMOD Procedure

Class Level Information				
Class	Value	Design Variables		
x6	no	0		
	yes	1		
x8	no	0		
	yes	1		
x9	no	0		
	yes	1		
x10	no	0		
	yes	1		
x11	no	0		
	yes	1		
x12	no	0		
	yes	1		
x13	no	0		
	yes	1		
x14	no	0		
	yes	1		
upset	do not want to answer	1	0	0
	missing	0	0	0
	no	0	1	0
	yes	0	0	1
hadajob	no	1		
	yes	0		
infected	no	0		
	yes	1		

Fully adjusted models of FEAR OF INFECTION with gender*theme interaction

The GENMOD Procedure

Criteria For Assessing Goodness Of Fit			
**Criterion**	**DF**	**Value**	**Value/DF**
**Deviance**	1242	902.1981	0.7264
**Scaled Deviance**	1242	1293.0000	1.0411
**Pearson Chi-Square**	1242	902.1981	0.7264
**Scaled Pearson X2**	1242	1293.0000	1.0411
**Log Likelihood**		-1602.0210	
**Full Log Likelihood**		-1602.0210	
**AIC (smaller is better)**		3308.0421	
**AICC (smaller is better)**		3312.4873	
**BIC (smaller is better)**		3576.6076	

Algorithm converged.

Analysis Of Maximum Likelihood Parameter Estimates						Wald 95% Confidence Limits			
**Parameter**			**DF**	**Estimate**	**Standard Error**			**Wald Chi-Square**	**Pr > ChiSq**
**Intercept**			1	-1.2758	0.1629	-1.5950	-0.9565	61.35	<.0001
**gender*x6**	Male	yes	1	0.2707	0.1887	-0.0992	0.6406	2.06	0.1515
**gender*x6**	Other	yes	1	0.0027	0.5413	-1.0583	1.0637	0.00	0.9960
**x1**	yes		1	0.0345	0.0638	-0.0906	0.1596	0.29	0.5891
**x2**	yes		1	0.1649	0.3222	-0.4667	0.7964	0.26	0.6089
**x3**	yes		1	-0.1232	0.1078	-0.3345	0.0881	1.31	0.2532
**x4**	yes		1	0.0082	0.0923	-0.1727	0.1890	0.01	0.9294
**x5**	yes		1	0.2744	0.0800	0.1175	0.4313	11.75	0.0006
**x6**	yes		1	-0.2903	0.0935	-0.4736	-0.1071	9.64	0.0019
**x8**	yes		1	0.1487	0.0789	-0.0060	0.3035	3.55	0.0595
**x9**	yes		1	-0.0690	0.2835	-0.6246	0.4866	0.06	0.8077

The GENMOD Procedure

Analysis Of Maximum Likelihood Parameter Estimates									
Parameter			DF	Estimate	Standard Error	Wald 95% Confidence Limits		Wald Chi-Square	Pr > ChiSq
x10	yes		1	-0.0077	0.0872	-0.1785	0.1632	0.01	0.9301
x11	yes		1	0.1579	0.2201	-0.2735	0.5894	0.51	0.4731
x12	yes		1	0.2548	0.2845	-0.3029	0.8125	0.80	0.3706
x13	yes		1	-0.0706	0.1536	-0.3715	0.2304	0.21	0.6458
x14	yes		1	0.0935	0.2708	-0.4372	0.6243	0.12	0.7299
upset	do not want to answer		1	0.0698	0.3138	-0.5453	0.6848	0.05	0.8240
upset	no		1	0.1664	0.0604	0.0481	0.2847	7.60	0.0058
upset	yes		1	0.2134	0.0962	0.0248	0.4020	4.92	0.0266
dur			1	0.0051	0.0009	0.0033	0.0068	32.51	<.0001
support_family			1	0.0010	0.0010	-0.0009	0.0029	1.09	0.2969
support_doctor			1	0.0016	0.0010	-0.0003	0.0035	2.84	0.0917
support_fedgovt			1	0.0000	0.0013	-0.0025	0.0025	0.00	0.9864
support_city			1	0.0032	0.0014	0.0004	0.0060	5.12	0.0236
support_deptpubhealt			1	0.0017	0.0013	-0.0009	0.0042	1.59	0.2075
support_faithcommuni			1	0.0019	0.0010	-0.0000	0.0038	3.77	0.0522
support_socialservic			1	0.0026	0.0013	-0.0000	0.0052	3.83	0.0502
support_neighbors			1	-0.0003	0.0010	-0.0022	0.0017	0.07	0.7877
support_other			1	0.0004	0.0012	-0.0020	0.0028	0.11	0.7414
infected	yes		1	0.1040	0.2355	-0.3576	0.5655	0.19	0.6588
feltunwell	missing		1	-0.9266	0.4229	-1.7555	-0.0977	4.80	0.0285
feltunwell	yes		1	0.0457	0.0574	-0.0668	0.1582	0.63	0.4259
compare_others	poor or fair		1	0.1526	0.0944	-0.0324	0.3376	2.62	0.1059
ratehealth	poor or fair		1	0.0578	0.1258	-0.1889	0.3045	0.21	0.6460
gender	Male		1	-0.1875	0.0583	-0.3017	-0.0733	10.35	0.0013
gender	Other		1	0.1069	0.2266	-0.3373	0.5511	0.22	0.6372

Fully adjusted models of FEAR OF INFECTION with gender*theme interaction

The GENMOD Procedure

Analysis Of Maximum Likelihood Parameter Estimates

Parameter			DF	Estimate	Standard Error	Wald 95% Confidence Limits		Wald Chi-Square	Pr > ChiSq
**race**	black		1	0.0738	0.0963	-0.1150	0.2626	0.59	0.4437
**race**	other race		1	-0.0671	0.0932	-0.2499	0.1156	0.52	0.4716
**age_cat**	35-54		1	0.0798	0.0744	-0.0660	0.2257	1.15	0.2832
**age_cat**	greater than 55		1	-0.0457	0.0802	-0.2029	0.1116	0.32	0.5692
**inc**	40,000 - $99,999		1	0.0888	0.0592	-0.0271	0.2048	2.25	0.1332
**inc**	less than $40,000		1	0.1597	0.0767	0.0095	0.3100	4.34	0.0372
**inc**	missing		1	0.0569	0.1529	-0.2427	0.3565	0.14	0.7097
**edu**	missing		1	-0.5727	0.3296	-1.2187	0.0732	3.02	0.0823
**edu**	no college degree		1	0.0672	0.0703	-0.0706	0.2050	0.91	0.3391
**marital_status**	married or living as married		1	0.2790	0.0618	0.1578	0.4001	20.36	<.0001
**marital_status**	missing		1	0.2732	0.3189	-0.3520	0.8983	0.73	0.3918
**marital_status**	widowed, divorced		1	0.0624	0.0863	-0.1068	0.2315	0.52	0.4699
**kids**	missing		1	-0.1165	0.3379	-0.7788	0.5458	0.12	0.7304
**kids**	yes		1	0.0582	0.0692	-0.0775	0.1939	0.71	0.4005
**hadajob**	no		1	-0.0457	0.0638	-0.1707	0.0794	0.51	0.4741
**Scale**			1	0.8353	0.0164	0.8037	0.8681		

Note: The scale parameter was estimated by maximum likelihood.

The GENMOD Procedure

Model Information	
Data Set	WORK.SCORES
Distribution	Normal
Link Function	Identity
Dependent Variable	Factor3

Number of Observations Read	1293
Number of Observations Used	1293

Class Level Information				
Class	Value	Design Variables		
gender	Female	0	0	
	Male	1	0	
	Other	0	1	
race	black	1	0	
	other race	0	1	
	white	0	0	
age_cat	35-54	1	0	
	greater than 55	0	1	
	less than 35	0	0	
inc	$100,000 and above	0	0	0
	40,000 - $99,999	1	0	0
	less than $40,000	0	1	0
	missing	0	0	1
edu	college degree or higher	0	0	
	missing	1	0	
	no college degree	0	1	

The GENMOD Procedure

Class Level Information				
Class	Value	Design Variables		
kids	missing	1	0	
	no	0	0	
	yes	0	1	
marital_status	married or living as married	1	0	0
	missing	0	1	0
	single	0	0	0
	widowed, divorced	0	0	1
feltunwell	missing	1	0	
	no	0	0	
	yes	0	1	
compare_others	good to excellent	0		
	poor or fair	1		
ratehealth	good to excellent	0		
	poor or fair	1		
x1	no	0		
	yes	1		
x2	no	0		
	yes	1		
x3	no	0		
	yes	1		
x4	no	0		
	yes	1		
x5	no	0		
	yes	1		

The GENMOD Procedure

Class Level Information				
Class	Value	Design Variables		
x6	no	0		
	yes	1		
x8	no	0		
	yes	1		
x9	no	0		
	yes	1		
x10	no	0		
	yes	1		
x11	no	0		
	yes	1		
x12	no	0		
	yes	1		
x13	no	0		
	yes	1		
x14	no	0		
	yes	1		
upset	do not want to answer	1	0	0
	missing	0	0	0
	no	0	1	0
	yes	0	0	1
hadajob	no	1		
	yes	0		
infected	no	0		
	yes	1		

The GENMOD Procedure

Criteria For Assessing Goodness Of Fit			
**Criterion**	**DF**	**Value**	**Value/DF**
**Deviance**	1242	902.9373	0.7270
**Scaled Deviance**	1242	1293.0000	1.0411
**Pearson Chi-Square**	1242	902.9373	0.7270
**Scaled Pearson X2**	1242	1293.0000	1.0411
**Log Likelihood**		-1602.5506	
**Full Log Likelihood**		-1602.5506	
**AIC (smaller is better)**		3309.1011	
**AICC (smaller is better)**		3313.5463	
**BIC (smaller is better)**		3577.6666	

Algorithm converged.

Analysis Of Maximum Likelihood Parameter Estimates									
**Parameter**			**DF**	**Estimate**	**Standard Error**	**Wald 95% Confidence Limits**		**Wald Chi-Square**	**Pr > ChiSq**
**Intercept**			1	-1.2771	0.1630	-1.5965	-0.9577	61.41	<.0001
**gender*x8**	Male	yes	1	-0.0981	0.2098	-0.5092	0.3130	0.22	0.6401
**gender*x8**	Other	yes	1	0.4174	0.4898	-0.5426	1.3773	0.73	0.3941
**x1**	yes		1	0.0316	0.0638	-0.0934	0.1567	0.25	0.6201
**x2**	yes		1	0.1521	0.3223	-0.4796	0.7837	0.22	0.6370
**x3**	yes		1	-0.1269	0.1078	-0.3382	0.0844	1.39	0.2392
**x4**	yes		1	0.0071	0.0924	-0.1740	0.1882	0.01	0.9385
**x5**	yes		1	0.2739	0.0801	0.1169	0.4308	11.69	0.0006
**x6**	yes		1	-0.2273	0.0814	-0.3870	-0.0677	7.79	0.0052
**x8**	yes		1	0.1494	0.0868	-0.0208	0.3196	2.96	0.0854
**x9**	yes		1	-0.0696	0.2836	-0.6255	0.4863	0.06	0.8062

The GENMOD Procedure

Analysis Of Maximum Likelihood Parameter Estimates

Parameter			DF	Estimate	Standard Error	Wald 95% Confidence Limits		Wald Chi-Square	Pr > ChiSq
x10	yes		1	-0.0151	0.0873	-0.1863	0.1560	0.03	0.8625
x11	yes		1	0.1473	0.2203	-0.2845	0.5790	0.45	0.5038
x12	yes		1	0.2560	0.2846	-0.3018	0.8138	0.81	0.3683
x13	yes		1	-0.0834	0.1540	-0.3852	0.2184	0.29	0.5881
x14	yes		1	0.0966	0.2710	-0.4345	0.6278	0.13	0.7214
upset	do not want to answer		1	0.0457	0.3152	-0.5721	0.6635	0.02	0.8847
upset	no		1	0.1682	0.0604	0.0499	0.2865	7.76	0.0053
upset	yes		1	0.2101	0.0962	0.0215	0.3987	4.77	0.0290
dur			1	0.0051	0.0009	0.0033	0.0068	32.11	<.0001
support_family			1	0.0010	0.0010	-0.0009	0.0029	0.98	0.3222
support_doctor			1	0.0016	0.0010	-0.0003	0.0035	2.78	0.0952
support_fedgovt			1	0.0000	0.0013	-0.0025	0.0025	0.00	0.9897
support_city			1	0.0031	0.0014	0.0004	0.0059	4.89	0.0270
support_deptpubhealt			1	0.0017	0.0013	-0.0009	0.0043	1.70	0.1923
support_faithcommuni			1	0.0019	0.0010	0.0000	0.0038	3.92	0.0477
support_socialservic			1	0.0027	0.0013	0.0001	0.0053	4.09	0.0432
support_neighbors			1	-0.0002	0.0010	-0.0021	0.0017	0.04	0.8447
support_other			1	0.0004	0.0012	-0.0020	0.0028	0.10	0.7566
infected	yes		1	0.1056	0.2356	-0.3561	0.5673	0.20	0.6540
feltunwell	missing		1	-0.9263	0.4231	-1.7556	-0.0970	4.79	0.0286
feltunwell	yes		1	0.0495	0.0576	-0.0633	0.1623	0.74	0.3901
compare_others	poor or fair		1	0.1536	0.0943	-0.0314	0.3385	2.65	0.1036
ratehealth	poor or fair		1	0.0568	0.1260	-0.1901	0.3037	0.20	0.6522
gender	Male		1	-0.1573	0.0580	-0.2709	-0.0437	7.37	0.0066
gender	Other		1	0.0122	0.2324	-0.4432	0.4677	0.00	0.9581

Fully adjusted models of FEAR OF INFECTION with gender*theme interaction

The GENMOD Procedure

Analysis Of Maximum Likelihood Parameter Estimates									
**Parameter**			**DF**	**Estimate**	**Standard Error**	**Wald 95% Confidence Limits**		**Wald Chi-Square**	**Pr > ChiSq**
**race**	black		1	0.0673	0.0963	-0.1214	0.2561	0.49	0.4845
**race**	other race		1	-0.0658	0.0933	-0.2487	0.1171	0.50	0.4806
**age_cat**	35-54		1	0.0817	0.0745	-0.0643	0.2276	1.20	0.2727
**age_cat**	greater than 55		1	-0.0493	0.0802	-0.2065	0.1079	0.38	0.5391
**inc**	40,000 - $99,999		1	0.0851	0.0591	-0.0307	0.2009	2.07	0.1499
**inc**	less than $40,000		1	0.1562	0.0766	0.0060	0.3065	4.16	0.0415
**inc**	missing		1	0.0539	0.1529	-0.2458	0.3536	0.12	0.7244
**edu**	missing		1	-0.5462	0.3293	-1.1916	0.0991	2.75	0.0971
**edu**	no college degree		1	0.0665	0.0704	-0.0714	0.2044	0.89	0.3449
**marital_status**	married or living as married		1	0.2829	0.0619	0.1617	0.4041	20.92	<.0001
**marital_status**	missing		1	0.2615	0.3192	-0.3642	0.8872	0.67	0.4127
**marital_status**	widowed, divorced		1	0.0673	0.0863	-0.1018	0.2364	0.61	0.4352
**kids**	missing		1	-0.1170	0.3388	-0.7809	0.5470	0.12	0.7299
**kids**	yes		1	0.0535	0.0693	-0.0823	0.1894	0.60	0.4401
**hadajob**	no		1	-0.0480	0.0638	-0.1731	0.0771	0.57	0.4517
**Scale**			1	0.8357	0.0164	0.8041	0.8685		

Note: The scale parameter was estimated by maximum likelihood.

Fully adjusted models of FEAR OF INFECTION with gender*theme interaction

The GENMOD Procedure

Model Information	
Data Set	WORK.SCORES
Distribution	Normal
Link Function	Identity
Dependent Variable	Factor3

Number of Observations Read	1293
Number of Observations Used	1293

Class Level Information				
Class	Value	Design Variables		
gender	Female	0	0	
	Male	1	0	
	Other	0	1	
race	black	1	0	
	other race	0	1	
	white	0	0	
age_cat	35-54	1	0	
	greater than 55	0	1	
	less than 35	0	0	
inc	$100,000 and above	0	0	0
	40,000 - $99,999	1	0	0
	less than $40,000	0	1	0
	missing	0	0	1
edu	college degree or higher	0	0	
	missing	1	0	
	no college degree	0	1	

The GENMOD Procedure

Class Level Information				
**Class**	**Value**	**Design Variables**		
**kids**	**missing**	1	0	
	**no**	0	0	
	**yes**	0	1	
**marital_status**	**married or living as married**	1	0	0
	**missing**	0	1	0
	**single**	0	0	0
	**widowed, divorced**	0	0	1
**feltunwell**	**missing**	1	0	
	**no**	0	0	
	**yes**	0	1	
**compare_others**	**good to excellent**	0		
	**poor or fair**	1		
**ratehealth**	**good to excellent**	0		
	**poor or fair**	1		
**x1**	**no**	0		
	**yes**	1		
**x2**	**no**	0		
	**yes**	1		
**x3**	**no**	0		
	**yes**	1		
**x4**	**no**	0		
	**yes**	1		
**x5**	**no**	0		
	**yes**	1		

The GENMOD Procedure

Class Level Information				
Class	Value	Design Variables		
x6	no	0		
	yes	1		
x8	no	0		
	yes	1		
x9	no	0		
	yes	1		
x10	no	0		
	yes	1		
x11	no	0		
	yes	1		
x12	no	0		
	yes	1		
x13	no	0		
	yes	1		
x14	no	0		
	yes	1		
upset	do not want to answer	1	0	0
	missing	0	0	0
	no	0	1	0
	yes	0	0	1
hadajob	no	1		
	yes	0		
infected	no	0		
	yes	1		

The GENMOD Procedure

Criteria For Assessing Goodness Of Fit			
**Criterion**	**DF**	**Value**	**Value/DF**
**Deviance**	1243	903.6368	0.7270
**Scaled Deviance**	1243	1293.0000	1.0402
**Pearson Chi-Square**	1243	903.6368	0.7270
**Scaled Pearson X2**	1243	1293.0000	1.0402
**Log Likelihood**		-1603.0512	
**Full Log Likelihood**		-1603.0512	
**AIC (smaller is better)**		3308.1024	
**AICC (smaller is better)**		3312.3764	
**BIC (smaller is better)**		3571.5032	

Algorithm converged.

Analysis Of Maximum Likelihood Parameter Estimates									
**Parameter**			**DF**	**Estimate**	**Standard Error**	**Wald 95% Confidence Limits**		**Wald Chi-Square**	**Pr > ChiSq**
**Intercept**			1	-1.2750	0.1632	-1.5948	-0.9552	61.07	<.0001
**gender*x9**	Male	yes	1	-0.0663	0.6797	-1.3984	1.2658	0.01	0.9223
**gender*x9**	Other	yes	0	0.0000	0.0000	0.0000	0.0000	.	.
**x1**	yes		1	0.0329	0.0638	-0.0922	0.1580	0.27	0.6065
**x2**	yes		1	0.1528	0.3224	-0.4790	0.7847	0.22	0.6355
**x3**	yes		1	-0.1288	0.1079	-0.3403	0.0826	1.43	0.2323
**x4**	yes		1	0.0059	0.0923	-0.1751	0.1868	0.00	0.9494
**x5**	yes		1	0.2733	0.0802	0.1162	0.4304	11.63	0.0007
**x6**	yes		1	-0.2280	0.0814	-0.3875	-0.0685	7.85	0.0051
**x8**	yes		1	0.1443	0.0789	-0.0103	0.2990	3.35	0.0674
**x9**	yes		1	-0.0546	0.3219	-0.6856	0.5764	0.03	0.8654

The GENMOD Procedure

Analysis Of Maximum Likelihood Parameter Estimates						Wald 95% Confidence Limits			
Parameter			DF	Estimate	Standard Error			Wald Chi-Square	Pr > ChiSq
x10	yes		1	-0.0103	0.0873	-0.1814	0.1608	0.01	0.9064
x11	yes		1	0.1518	0.2203	-0.2799	0.5835	0.47	0.4907
x12	yes		1	0.2494	0.2846	-0.3083	0.8071	0.77	0.3808
x13	yes		1	-0.0737	0.1537	-0.3750	0.2276	0.23	0.6316
x14	yes		1	0.0988	0.2710	-0.4323	0.6299	0.13	0.7154
upset	do not want to answer		1	0.0764	0.3133	-0.5377	0.6905	0.06	0.8074
upset	no		1	0.1681	0.0604	0.0497	0.2865	7.75	0.0054
upset	yes		1	0.2096	0.0963	0.0209	0.3983	4.74	0.0294
dur			1	0.0051	0.0009	0.0033	0.0068	32.17	<.0001
support_family			1	0.0010	0.0010	-0.0009	0.0029	1.03	0.3102
support_doctor			1	0.0016	0.0010	-0.0003	0.0035	2.76	0.0963
support_fedgovt			1	0.0000	0.0013	-0.0025	0.0025	0.00	0.9763
support_city			1	0.0032	0.0014	0.0004	0.0060	5.03	0.0250
support_deptpubhealt			1	0.0017	0.0013	-0.0009	0.0043	1.67	0.1967
support_faithcommuni			1	0.0019	0.0010	0.0000	0.0038	3.94	0.0472
support_socialservic			1	0.0027	0.0013	0.0001	0.0053	4.00	0.0456
support_neighbors			1	-0.0002	0.0010	-0.0022	0.0017	0.06	0.8127
support_other			1	0.0004	0.0012	-0.0020	0.0027	0.09	0.7603
infected	yes		1	0.1043	0.2357	-0.3576	0.5662	0.20	0.6580
feltunwell	missing		1	-0.9241	0.4233	-1.7536	-0.0945	4.77	0.0290
feltunwell	yes		1	0.0473	0.0574	-0.0653	0.1599	0.68	0.4103
compare_others	poor or fair		1	0.1533	0.0944	-0.0317	0.3383	2.64	0.1043
ratehealth	poor or fair		1	0.0549	0.1259	-0.1919	0.3016	0.19	0.6630
gender	Male		1	-0.1633	0.0560	-0.2731	-0.0534	8.49	0.0036
gender	Other		1	0.1024	0.2074	-0.3040	0.5088	0.24	0.6215

The GENMOD Procedure

Analysis Of Maximum Likelihood Parameter Estimates									
Parameter			DF	Estimate	Standard Error	Wald 95% Confidence Limits		Wald Chi-Square	Pr > ChiSq
**race**	black		1	0.0688	0.0963	-0.1201	0.2576	0.51	0.4753
**race**	other race		1	-0.0656	0.0933	-0.2484	0.1173	0.49	0.4821
**age_cat**	35-54		1	0.0794	0.0746	-0.0668	0.2256	1.13	0.2874
**age_cat**	greater than 55		1	-0.0505	0.0802	-0.2077	0.1067	0.40	0.5291
**inc**	40,000 - $99,999		1	0.0829	0.0591	-0.0329	0.1987	1.97	0.1605
**inc**	less than $40,000		1	0.1552	0.0767	0.0050	0.3054	4.10	0.0429
**inc**	missing		1	0.0535	0.1530	-0.2463	0.3533	0.12	0.7265
**edu**	missing		1	-0.5469	0.3293	-1.1924	0.0985	2.76	0.0968
**edu**	no college degree		1	0.0667	0.0704	-0.0712	0.2046	0.90	0.3431
**marital_status**	married or living as married		1	0.2811	0.0619	0.1599	0.4024	20.66	<.0001
**marital_status**	missing		1	0.2679	0.3192	-0.3576	0.8935	0.70	0.4012
**marital_status**	widowed, divorced		1	0.0668	0.0863	-0.1023	0.2360	0.60	0.4388
**kids**	missing		1	-0.1271	0.3381	-0.7898	0.5355	0.14	0.7069
**kids**	yes		1	0.0572	0.0694	-0.0788	0.1933	0.68	0.4096
**hadajob**	no		1	-0.0470	0.0638	-0.1721	0.0782	0.54	0.4619
**Scale**			1	0.8360	0.0164	0.8044	0.8688		

Note: The scale parameter was estimated by maximum likelihood.

Fully adjusted models of FEAR OF INFECTION with gender*theme interaction

The GENMOD Procedure

Model Information	
Data Set	WORK.SCORES
Distribution	Normal
Link Function	Identity
Dependent Variable	Factor3

Number of Observations Read	1293
Number of Observations Used	1293

Class Level Information

Class	Value	Design Variables		
gender	Female	0	0	
	Male	1	0	
	Other	0	1	
race	black	1	0	
	other race	0	1	
	white	0	0	
age_cat	35-54	1	0	
	greater than 55	0	1	
	less than 35	0	0	
inc	$100,000 and above	0	0	0
	40,000 - $99,999	1	0	0
	less than $40,000	0	1	0
	missing	0	0	1
edu	college degree or higher	0	0	
	missing	1	0	
	no college degree	0	1	

The GENMOD Procedure

Class Level Information				
**Class**	**Value**	**Design Variables**		
**kids**	**missing**	1	0	
	**no**	0	0	
	**yes**	0	1	
**marital_status**	**married or living as married**	1	0	0
	**missing**	0	1	0
	**single**	0	0	0
	**widowed, divorced**	0	0	1
**feltunwell**	**missing**	1	0	
	**no**	0	0	
	**yes**	0	1	
**compare_others**	**good to excellent**	0		
	**poor or fair**	1		
**ratehealth**	**good to excellent**	0		
	**poor or fair**	1		
**x1**	**no**	0		
	**yes**	1		
**x2**	**no**	0		
	**yes**	1		
**x3**	**no**	0		
	**yes**	1		
**x4**	**no**	0		
	**yes**	1		
**x5**	**no**	0		
	**yes**	1		

The GENMOD Procedure

Class Level Information				
Class	Value	Design Variables		
x6	no	0		
	yes	1		
x8	no	0		
	yes	1		
x9	no	0		
	yes	1		
x10	no	0		
	yes	1		
x11	no	0		
	yes	1		
x12	no	0		
	yes	1		
x13	no	0		
	yes	1		
x14	no	0		
	yes	1		
upset	do not want to answer	1	0	0
	missing	0	0	0
	no	0	1	0
	yes	0	0	1
hadajob	no	1		
	yes	0		
infected	no	0		
	yes	1		

The GENMOD Procedure

Criteria For Assessing Goodness Of Fit			
**Criterion**	**DF**	**Value**	**Value/DF**
**Deviance**	1242	899.6654	0.7244
**Scaled Deviance**	1242	1293.0000	1.0411
**Pearson Chi-Square**	1242	899.6654	0.7244
**Scaled Pearson X2**	1242	1293.0000	1.0411
**Log Likelihood**		-1600.2036	
**Full Log Likelihood**		-1600.2036	
**AIC (smaller is better)**		3304.4073	
**AICC (smaller is better)**		3308.8524	
**BIC (smaller is better)**		3572.9727	

Algorithm converged.

Analysis Of Maximum Likelihood Parameter Estimates									
**Parameter**			**DF**	**Estimate**	**Standard Error**	**Wald 95% Confidence Limits**		**Wald Chi-Square**	**Pr > ChiSq**
**Intercept**			1	-1.2580	0.1627	-1.5768	-0.9391	59.78	<.0001
**gender*x10**	Male	yes	1	-0.3280	0.1929	-0.7060	0.0500	2.89	0.0890
**gender*x10**	Other	yes	1	-1.1702	0.6412	-2.4269	0.0865	3.33	0.0680
**x1**	yes		1	0.0373	0.0637	-0.0876	0.1622	0.34	0.5580
**x2**	yes		1	0.1357	0.3218	-0.4950	0.7664	0.18	0.6732
**x3**	yes		1	-0.1307	0.1076	-0.3416	0.0802	1.48	0.2244
**x4**	yes		1	0.0014	0.0922	-0.1792	0.1820	0.00	0.9877
**x5**	yes		1	0.2650	0.0800	0.1081	0.4219	10.96	0.0009
**x6**	yes		1	-0.2341	0.0812	-0.3933	-0.0749	8.31	0.0040
**x8**	yes		1	0.1453	0.0788	-0.0092	0.2999	3.40	0.0653
**x9**	yes		1	-0.0511	0.2833	-0.6064	0.5042	0.03	0.8568

The GENMOD Procedure

Analysis Of Maximum Likelihood Parameter Estimates									
Parameter			DF	Estimate	Standard Error	Wald 95% Confidence Limits		Wald Chi-Square	Pr > ChiSq
x10	yes		1	0.0995	0.1026	-0.1015	0.3006	0.94	0.3317
x11	yes		1	0.1436	0.2198	-0.2872	0.5744	0.43	0.5136
x12	yes		1	0.2227	0.2842	-0.3343	0.7796	0.61	0.4333
x13	yes		1	-0.0775	0.1533	-0.3780	0.2231	0.26	0.6135
x14	yes		1	0.1031	0.2704	-0.4269	0.6331	0.15	0.7030
upset	do not want to answer		1	0.0254	0.3135	-0.5890	0.6398	0.01	0.9354
upset	no		1	0.1631	0.0603	0.0449	0.2813	7.31	0.0068
upset	yes		1	0.2105	0.0961	0.0222	0.3989	4.80	0.0285
dur			1	0.0050	0.0009	0.0032	0.0067	30.80	<.0001
support_family			1	0.0010	0.0010	-0.0009	0.0029	1.13	0.2872
support_doctor			1	0.0016	0.0010	-0.0003	0.0035	2.72	0.0989
support_fedgovt			1	0.0001	0.0013	-0.0025	0.0026	0.00	0.9684
support_city			1	0.0031	0.0014	0.0003	0.0059	4.85	0.0277
support_deptpubhealt			1	0.0018	0.0013	-0.0008	0.0044	1.83	0.1759
support_faithcommuni			1	0.0019	0.0010	0.0000	0.0038	3.87	0.0491
support_socialservic			1	0.0026	0.0013	-0.0001	0.0052	3.68	0.0552
support_neighbors			1	-0.0002	0.0010	-0.0021	0.0018	0.03	0.8525
support_other			1	0.0003	0.0012	-0.0021	0.0027	0.07	0.7974
infected	yes		1	0.1058	0.2351	-0.3550	0.5667	0.20	0.6526
feltunwell	missing		1	-0.9230	0.4223	-1.7507	-0.0952	4.78	0.0289
feltunwell	yes		1	0.0441	0.0573	-0.0682	0.1565	0.59	0.4412
compare_others	poor or fair		1	0.1471	0.0942	-0.0376	0.3318	2.44	0.1184
ratehealth	poor or fair		1	0.0628	0.1256	-0.1834	0.3090	0.25	0.6171
gender	Male		1	-0.1357	0.0581	-0.2496	-0.0217	5.45	0.0196
gender	Other		1	0.2311	0.2190	-0.1981	0.6603	1.11	0.2913

The GENMOD Procedure

Analysis Of Maximum Likelihood Parameter Estimates									
**Parameter**			**DF**	**Estimate**	**Standard Error**	**Wald 95% Confidence Limits**		**Wald Chi-Square**	**Pr > ChiSq**
**race**	black		1	0.0634	0.0962	-0.1251	0.2519	0.43	0.5099
**race**	other race		1	-0.0709	0.0931	-0.2534	0.1116	0.58	0.4463
**age_cat**	35-54		1	0.0730	0.0743	-0.0727	0.2187	0.96	0.3260
**age_cat**	greater than 55		1	-0.0543	0.0801	-0.2112	0.1027	0.46	0.4980
**inc**	40,000 - $99,999		1	0.0796	0.0590	-0.0359	0.1952	1.82	0.1767
**inc**	less than $40,000		1	0.1553	0.0765	0.0053	0.3053	4.12	0.0425
**inc**	missing		1	0.0537	0.1526	-0.2455	0.3528	0.12	0.7251
**edu**	missing		1	-0.5139	0.3291	-1.1590	0.1311	2.44	0.1184
**edu**	no college degree		1	0.0697	0.0702	-0.0680	0.2073	0.98	0.3214
**marital_status**	married or living as married		1	0.2742	0.0618	0.1531	0.3953	19.70	<.0001
**marital_status**	missing		1	0.2582	0.3185	-0.3661	0.8825	0.66	0.4176
**marital_status**	widowed, divorced		1	0.0691	0.0861	-0.0997	0.2379	0.64	0.4225
**kids**	missing		1	-0.1397	0.3375	-0.8012	0.5217	0.17	0.6788
**kids**	yes		1	0.0578	0.0691	-0.0776	0.1933	0.70	0.4026
**hadajob**	no		1	-0.0492	0.0637	-0.1740	0.0756	0.60	0.4399
**Scale**			1	0.8341	0.0164	0.8026	0.8669		

Note: The scale parameter was estimated by maximum likelihood.

The MEANS Procedure

Analysis Variable : Factor3							
Gender	uncertainty	N Obs	N	Mean	Std Dev	Minimum	Maximum
Female	no	873	873	0.0334145	0.9136801	-2.1672663	1.8860752
	yes	76	76	0.2548061	0.8800465	-1.6841618	1.8584198
Male	no	297	297	-0.1208365	0.8796323	-2.1478807	1.8420127
	yes	29	29	-0.4572879	0.7527650	-2.0636502	1.3399161
Other	no	16	16	0.1757922	0.9770413	-1.3732372	1.6937479
	yes	2	2	-1.0995307	1.1719092	-1.9281957	-0.2708657

The GENMOD Procedure

Model Information	
Data Set	WORK.SCORES
Distribution	Normal
Link Function	Identity
Dependent Variable	Factor3

Number of Observations Read	1293
Number of Observations Used	1293

Class Level Information				
Class	Value	Design Variables		
gender	Female	0	0	
	Male	1	0	
	Other	0	1	
race	black	1	0	
	other race	0	1	
	white	0	0	
age_cat	35-54	1	0	
	greater than 55	0	1	
	less than 35	0	0	
inc	$100,000 and above	0	0	0
	40,000 - $99,999	1	0	0
	less than $40,000	0	1	0
	missing	0	0	1
edu	college degree or higher	0	0	
	missing	1	0	
	no college degree	0	1	

The GENMOD Procedure

Class Level Information				
Class	Value	Design Variables		
kids	missing	1	0	
	no	0	0	
	yes	0	1	
marital_status	married or living as married	1	0	0
	missing	0	1	0
	single	0	0	0
	widowed, divorced	0	0	1
feltunwell	missing	1	0	
	no	0	0	
	yes	0	1	
compare_others	good to excellent	0		
	poor or fair	1		
ratehealth	good to excellent	0		
	poor or fair	1		
x1	no	0		
	yes	1		
x2	no	0		
	yes	1		
x3	no	0		
	yes	1		
x4	no	0		
	yes	1		
x5	no	0		
	yes	1		

The GENMOD Procedure

Class Level Information				
Class	Value	Design Variables		
x6	no	0		
	yes	1		
x8	no	0		
	yes	1		
x9	no	0		
	yes	1		
x10	no	0		
	yes	1		
x11	no	0		
	yes	1		
x12	no	0		
	yes	1		
x13	no	0		
	yes	1		
x14	no	0		
	yes	1		
upset	do not want to answer	1	0	0
	missing	0	0	0
	no	0	1	0
	yes	0	0	1
hadajob	no	1		
	yes	0		
infected	no	0		
	yes	1		

The GENMOD Procedure

Criteria For Assessing Goodness Of Fit			
**Criterion**	**DF**	**Value**	**Value/DF**
**Deviance**	1243	903.6392	0.7270
**Scaled Deviance**	1243	1293.0000	1.0402
**Pearson Chi-Square**	1243	903.6392	0.7270
**Scaled Pearson X2**	1243	1293.0000	1.0402
**Log Likelihood**		-1603.0529	
**Full Log Likelihood**		-1603.0529	
**AIC (smaller is better)**		3308.1058	
**AICC (smaller is better)**		3312.3797	
**BIC (smaller is better)**		3571.5065	

Algorithm converged.

Analysis Of Maximum Likelihood Parameter Estimates									
**Parameter**			**DF**	**Estimate**	**Standard Error**	**Wald 95% Confidence Limits**		**Wald Chi-Square**	**Pr > ChiSq**
**Intercept**			1	-1.2760	0.1629	-1.5953	-0.9568	61.37	<.0001
**gender*x11**	Male	yes	1	-0.0367	0.4677	-0.9534	0.8799	0.01	0.9374
**gender*x11**	Other	yes	0	0.0000	0.0000	0.0000	0.0000	.	.
**x1**	yes		1	0.0328	0.0638	-0.0923	0.1579	0.26	0.6071
**x2**	yes		1	0.1529	0.3224	-0.4789	0.7848	0.23	0.6352
**x3**	yes		1	-0.1290	0.1080	-0.3406	0.0826	1.43	0.2321
**x4**	yes		1	0.0061	0.0924	-0.1749	0.1872	0.00	0.9470
**x5**	yes		1	0.2737	0.0801	0.1167	0.4308	11.67	0.0006
**x6**	yes		1	-0.2281	0.0814	-0.3876	-0.0685	7.85	0.0051
**x8**	yes		1	0.1442	0.0789	-0.0105	0.2989	3.34	0.0678
**x9**	yes		1	-0.0693	0.2837	-0.6253	0.4868	0.06	0.8071

The GENMOD Procedure

Analysis Of Maximum Likelihood Parameter Estimates

Parameter			DF	Estimate	Standard Error	Wald 95% Confidence Limits		Wald Chi-Square	Pr > ChiSq
**x10**	yes		1	-0.0108	0.0872	-0.1818	0.1601	0.02	0.9011
**x11**	yes		1	0.1639	0.2694	-0.3640	0.6919	0.37	0.5428
**x12**	yes		1	0.2494	0.2846	-0.3084	0.8071	0.77	0.3808
**x13**	yes		1	-0.0733	0.1537	-0.3745	0.2278	0.23	0.6332
**x14**	yes		1	0.0989	0.2710	-0.4323	0.6300	0.13	0.7152
**upset**	do not want to answer		1	0.0766	0.3133	-0.5375	0.6907	0.06	0.8069
**upset**	no		1	0.1682	0.0604	0.0498	0.2865	7.75	0.0054
**upset**	yes		1	0.2099	0.0963	0.0211	0.3987	4.75	0.0293
**dur**			1	0.0051	0.0009	0.0033	0.0068	32.22	<.0001
**support_family**			1	0.0010	0.0010	-0.0009	0.0029	1.04	0.3082
**support_doctor**			1	0.0016	0.0010	-0.0003	0.0035	2.77	0.0963
**support_fedgovt**			1	0.0000	0.0013	-0.0025	0.0025	0.00	0.9758
**support_city**			1	0.0032	0.0014	0.0004	0.0060	5.03	0.0250
**support_deptpubhealt**			1	0.0017	0.0013	-0.0009	0.0043	1.67	0.1967
**support_faithcommuni**			1	0.0019	0.0010	0.0000	0.0038	3.94	0.0471
**support_socialservic**			1	0.0027	0.0013	0.0001	0.0053	4.02	0.0449
**support_neighbors**			1	-0.0002	0.0010	-0.0022	0.0017	0.06	0.8102
**support_other**			1	0.0004	0.0012	-0.0020	0.0028	0.10	0.7567
**infected**	yes		1	0.1061	0.2369	-0.3582	0.5703	0.20	0.6543
**feltunwell**	missing		1	-0.9241	0.4233	-1.7536	-0.0945	4.77	0.0290
**feltunwell**	yes		1	0.0474	0.0574	-0.0652	0.1599	0.68	0.4093
**compare_others**	poor or fair		1	0.1535	0.0944	-0.0315	0.3384	2.64	0.1039
**ratehealth**	poor or fair		1	0.0545	0.1259	-0.1922	0.3012	0.19	0.6651
**gender**	Male		1	-0.1632	0.0562	-0.2733	-0.0531	8.44	0.0037
**gender**	Other		1	0.1027	0.2074	-0.3037	0.5092	0.25	0.6204

Fully adjusted models of FEAR OF INFECTION with gender*theme interaction

The GENMOD Procedure

Analysis Of Maximum Likelihood Parameter Estimates									
**Parameter**			**DF**	**Estimate**	**Standard Error**	**Wald 95% Confidence Limits**		**Wald Chi-Square**	**Pr > ChiSq**
**race**	black		1	0.0689	0.0963	-0.1200	0.2577	0.51	0.4747
**race**	other race		1	-0.0656	0.0933	-0.2485	0.1173	0.49	0.4821
**age_cat**	35-54		1	0.0798	0.0745	-0.0661	0.2257	1.15	0.2837
**age_cat**	greater than 55		1	-0.0504	0.0802	-0.2076	0.1068	0.39	0.5297
**inc**	40,000 - $99,999		1	0.0828	0.0591	-0.0331	0.1987	1.96	0.1614
**inc**	less than $40,000		1	0.1549	0.0768	0.0045	0.3053	4.07	0.0436
**inc**	missing		1	0.0534	0.1530	-0.2464	0.3532	0.12	0.7269
**edu**	missing		1	-0.5468	0.3293	-1.1923	0.0986	2.76	0.0968
**edu**	no college degree		1	0.0668	0.0704	-0.0712	0.2047	0.90	0.3429
**marital_status**	married or living as married		1	0.2810	0.0619	0.1597	0.4023	20.61	<.0001
**marital_status**	missing		1	0.2674	0.3192	-0.3583	0.8931	0.70	0.4022
**marital_status**	widowed, divorced		1	0.0667	0.0863	-0.1025	0.2359	0.60	0.4398
**kids**	missing		1	-0.1269	0.3381	-0.7896	0.5357	0.14	0.7074
**kids**	yes		1	0.0569	0.0693	-0.0789	0.1927	0.68	0.4113
**hadajob**	no		1	-0.0469	0.0638	-0.1720	0.0782	0.54	0.4628
**Scale**			1	0.8360	0.0164	0.8044	0.8688		

Note: The scale parameter was estimated by maximum likelihood.

The GENMOD Procedure

Model Information	
Data Set	WORK.SCORES
Distribution	Normal
Link Function	Identity
Dependent Variable	Factor3

Number of Observations Read	1293
Number of Observations Used	1293

Class Level Information				
Class	Value	Design Variables		
gender	Female	0	0	
	Male	1	0	
	Other	0	1	
race	black	1	0	
	other race	0	1	
	white	0	0	
age_cat	35-54	1	0	
	greater than 55	0	1	
	less than 35	0	0	
inc	$100,000 and above	0	0	0
	40,000 - $99,999	1	0	0
	less than $40,000	0	1	0
	missing	0	0	1
edu	college degree or higher	0	0	
	missing	1	0	
	no college degree	0	1	

The GENMOD Procedure

Class Level Information				
**Class**	**Value**	**Design Variables**		
**kids**	**missing**	1	0	
	**no**	0	0	
	**yes**	0	1	
**marital_status**	**married or living as married**	1	0	0
	**missing**	0	1	0
	**single**	0	0	0
	**widowed, divorced**	0	0	1
**feltunwell**	**missing**	1	0	
	**no**	0	0	
	**yes**	0	1	
**compare_others**	**good to excellent**	0		
	**poor or fair**	1		
**ratehealth**	**good to excellent**	0		
	**poor or fair**	1		
**x1**	**no**	0		
	**yes**	1		
**x2**	**no**	0		
	**yes**	1		
**x3**	**no**	0		
	**yes**	1		
**x4**	**no**	0		
	**yes**	1		
**x5**	**no**	0		
	**yes**	1		

The GENMOD Procedure

Class Level Information				
**Class**	**Value**	**Design Variables**		
x6	no	0		
	yes	1		
x8	no	0		
	yes	1		
x9	no	0		
	yes	1		
x10	no	0		
	yes	1		
x11	no	0		
	yes	1		
x12	no	0		
	yes	1		
x13	no	0		
	yes	1		
x14	no	0		
	yes	1		
upset	do not want to answer	1	0	0
	missing	0	0	0
	no	0	1	0
	yes	0	0	1
hadajob	no	1		
	yes	0		
infected	no	0		
	yes	1		

The GENMOD Procedure

Criteria For Assessing Goodness Of Fit			
**Criterion**	**DF**	**Value**	**Value/DF**
**Deviance**	1242	903.6427	0.7276
**Scaled Deviance**	1242	1293.0000	1.0411
**Pearson Chi-Square**	1242	903.6427	0.7276
**Scaled Pearson X2**	1242	1293.0000	1.0411
**Log Likelihood**		-1603.0554	
**Full Log Likelihood**		-1603.0554	
**AIC (smaller is better)**		3310.1108	
**AICC (smaller is better)**		3314.5560	
**BIC (smaller is better)**		3578.6763	

Algorithm converged.

Analysis Of Maximum Likelihood Parameter Estimates									
**Parameter**			**DF**	**Estimate**	**Standard Error**	**Wald 95% Confidence Limits**		**Wald Chi-Square**	**Pr > ChiSq**
**Intercept**			1	-1.2757	0.1633	-1.5957	-0.9556	61.04	<.0001
**gender*x12**	Male	yes	1	0.0098	0.6905	-1.3435	1.3631	0.00	0.9887
**gender*x12**	Other	yes	1	-0.0261	0.9437	-1.8757	1.8235	0.00	0.9780
**x1**	yes		1	0.0328	0.0638	-0.0923	0.1580	0.26	0.6071
**x2**	yes		1	0.1529	0.3224	-0.4790	0.7848	0.22	0.6353
**x3**	yes		1	-0.1286	0.1078	-0.3400	0.0827	1.42	0.2330
**x4**	yes		1	0.0060	0.0923	-0.1750	0.1869	0.00	0.9486
**x5**	yes		1	0.2736	0.0802	0.1165	0.4307	11.65	0.0006
**x6**	yes		1	-0.2279	0.0814	-0.3875	-0.0684	7.84	0.0051
**x8**	yes		1	0.1444	0.0789	-0.0102	0.2991	3.35	0.0672
**x9**	yes		1	-0.0695	0.2837	-0.6256	0.4866	0.06	0.8065

Fully adjusted models of FEAR OF INFECTION with gender*theme interaction

The GENMOD Procedure

Analysis Of Maximum Likelihood Parameter Estimates									
Parameter			DF	Estimate	Standard Error	Wald 95% Confidence Limits		Wald Chi-Square	Pr > ChiSq
**x10**	yes		1	-0.0107	0.0872	-0.1816	0.1603	0.01	0.9028
**x11**	yes		1	0.1517	0.2203	-0.2799	0.5834	0.47	0.4908
**x12**	yes		1	0.2500	0.3475	-0.4310	0.9310	0.52	0.4719
**x13**	yes		1	-0.0733	0.1537	-0.3745	0.2279	0.23	0.6334
**x14**	yes		1	0.0987	0.2710	-0.4324	0.6299	0.13	0.7156
**upset**	do not want to answer		1	0.0761	0.3135	-0.5383	0.6905	0.06	0.8083
**upset**	no		1	0.1682	0.0604	0.0497	0.2866	7.75	0.0054
**upset**	yes		1	0.2097	0.0963	0.0210	0.3984	4.74	0.0294
**dur**			1	0.0051	0.0009	0.0033	0.0068	32.28	<.0001
**support_family**			1	0.0010	0.0010	-0.0009	0.0029	1.02	0.3115
**support_doctor**			1	0.0016	0.0010	-0.0003	0.0035	2.77	0.0960
**support_fedgovt**			1	0.0000	0.0013	-0.0025	0.0025	0.00	0.9783
**support_city**			1	0.0032	0.0014	0.0004	0.0060	5.02	0.0251
**support_deptpubhealt**			1	0.0017	0.0013	-0.0009	0.0043	1.66	0.1981
**support_faithcommuni**			1	0.0019	0.0010	0.0000	0.0038	3.94	0.0471
**support_socialservic**			1	0.0027	0.0013	0.0001	0.0053	4.00	0.0455
**support_neighbors**			1	-0.0002	0.0010	-0.0022	0.0017	0.06	0.8130
**support_other**			1	0.0004	0.0012	-0.0020	0.0028	0.09	0.7601
**infected**	yes		1	0.1044	0.2357	-0.3576	0.5664	0.20	0.6579
**feltunwell**	missing		1	-0.9241	0.4233	-1.7537	-0.0945	4.77	0.0290
**feltunwell**	yes		1	0.0473	0.0574	-0.0652	0.1599	0.68	0.4098
**compare_others**	poor or fair		1	0.1534	0.0944	-0.0316	0.3385	2.64	0.1042
**ratehealth**	poor or fair		1	0.0546	0.1259	-0.1922	0.3013	0.19	0.6647
**gender**	Male		1	-0.1637	0.0560	-0.2735	-0.0539	8.54	0.0035
**gender**	Other		1	0.1038	0.2124	-0.3124	0.5201	0.24	0.6249

Fully adjusted models of FEAR OF INFECTION with gender*theme interaction

The GENMOD Procedure

Analysis Of Maximum Likelihood Parameter Estimates									
**Parameter**			**DF**	**Estimate**	**Standard Error**	**Wald 95% Confidence Limits**		**Wald Chi-Square**	**Pr > ChiSq**
**race**	black		1	0.0689	0.0964	-0.1200	0.2578	0.51	0.4749
**race**	other race		1	-0.0655	0.0933	-0.2484	0.1173	0.49	0.4825
**age_cat**	35-54		1	0.0797	0.0745	-0.0664	0.2258	1.14	0.2849
**age_cat**	greater than 55		1	-0.0505	0.0803	-0.2079	0.1070	0.40	0.5297
**inc**	40,000 - $99,999		1	0.0829	0.0591	-0.0329	0.1988	1.97	0.1605
**inc**	less than $40,000		1	0.1552	0.0767	0.0050	0.3055	4.10	0.0429
**inc**	missing		1	0.0535	0.1530	-0.2463	0.3533	0.12	0.7266
**edu**	missing		1	-0.5469	0.3293	-1.1923	0.0986	2.76	0.0968
**edu**	no college degree		1	0.0666	0.0704	-0.0714	0.2046	0.89	0.3443
**marital_status**	married or living as married		1	0.2811	0.0619	0.1599	0.4024	20.64	<.0001
**marital_status**	missing		1	0.2679	0.3192	-0.3577	0.8935	0.70	0.4013
**marital_status**	widowed, divorced		1	0.0668	0.0863	-0.1023	0.2360	0.60	0.4388
**kids**	missing		1	-0.1269	0.3381	-0.7896	0.5358	0.14	0.7074
**kids**	yes		1	0.0568	0.0693	-0.0789	0.1926	0.67	0.4119
**hadajob**	no		1	-0.0467	0.0639	-0.1720	0.0786	0.53	0.4652
**Scale**			1	0.8360	0.0164	0.8044	0.8688		

Note: The scale parameter was estimated by maximum likelihood.

The GENMOD Procedure

Model Information	
Data Set	WORK.SCORES
Distribution	Normal
Link Function	Identity
Dependent Variable	Factor3

Number of Observations Read	1293
Number of Observations Used	1293

Class Level Information				
Class	Value	Design Variables		
gender	Female	0	0	
	Male	1	0	
	Other	0	1	
race	black	1	0	
	other race	0	1	
	white	0	0	
age_cat	35-54	1	0	
	greater than 55	0	1	
	less than 35	0	0	
inc	$100,000 and above	0	0	0
	40,000 - $99,999	1	0	0
	less than $40,000	0	1	0
	missing	0	0	1
edu	college degree or higher	0	0	
	missing	1	0	
	no college degree	0	1	

The GENMOD Procedure

Class Level Information				
**Class**	**Value**	**Design Variables**		
**kids**	**missing**	1	0	
	**no**	0	0	
	**yes**	0	1	
**marital_status**	**married or living as married**	1	0	0
	**missing**	0	1	0
	**single**	0	0	0
	**widowed, divorced**	0	0	1
**feltunwell**	**missing**	1	0	
	**no**	0	0	
	**yes**	0	1	
**compare_others**	**good to excellent**	0		
	**poor or fair**	1		
**ratehealth**	**good to excellent**	0		
	**poor or fair**	1		
**x1**	**no**	0		
	**yes**	1		
**x2**	**no**	0		
	**yes**	1		
**x3**	**no**	0		
	**yes**	1		
**x4**	**no**	0		
	**yes**	1		
**x5**	**no**	0		
	**yes**	1		

The GENMOD Procedure

Class Level Information				
**Class**	**Value**	**Design Variables**		
**x6**	**no**	0		
	**yes**	1		
**x8**	**no**	0		
	**yes**	1		
**x9**	**no**	0		
	**yes**	1		
**x10**	**no**	0		
	**yes**	1		
**x11**	**no**	0		
	**yes**	1		
**x12**	**no**	0		
	**yes**	1		
**x13**	**no**	0		
	**yes**	1		
**x14**	**no**	0		
	**yes**	1		
**upset**	**do not want to answer**	1	0	0
	**missing**	0	0	0
	**no**	0	1	0
	**yes**	0	0	1
**hadajob**	**no**	1		
	**yes**	0		
**infected**	**no**	0		
	**yes**	1		

The GENMOD Procedure

Criteria For Assessing Goodness Of Fit			
**Criterion**	**DF**	**Value**	**Value/DF**
**Deviance**	1242	897.8422	0.7229
**Scaled Deviance**	1242	1293.0000	1.0411
**Pearson Chi-Square**	1242	897.8422	0.7229
**Scaled Pearson X2**	1242	1293.0000	1.0411
**Log Likelihood**		-1598.8921	
**Full Log Likelihood**		-1598.8921	
**AIC (smaller is better)**		3301.7843	
**AICC (smaller is better)**		3306.2295	
**BIC (smaller is better)**		3570.3498	

Algorithm converged.

Analysis Of Maximum Likelihood Parameter Estimates									
**Parameter**			**DF**	**Estimate**	**Standard Error**	**Wald 95% Confidence Limits**		**Wald Chi-Square**	**Pr > ChiSq**
**Intercept**			1	-1.2781	0.1624	-1.5963	-0.9599	61.96	<.0001
**gender*x13**	Male	yes	1	0.4546	0.3642	-0.2592	1.1685	1.56	0.2119
**gender*x13**	Other	yes	1	2.5412	0.9372	0.7042	4.3781	7.35	0.0067
**x1**	yes		1	0.0350	0.0636	-0.0897	0.1597	0.30	0.5821
**x2**	yes		1	0.1488	0.3214	-0.4810	0.7786	0.21	0.6433
**x3**	yes		1	-0.1290	0.1075	-0.3397	0.0817	1.44	0.2302
**x4**	yes		1	0.0010	0.0921	-0.1795	0.1814	0.00	0.9916
**x5**	yes		1	0.2745	0.0799	0.1180	0.4310	11.81	0.0006
**x6**	yes		1	-0.2257	0.0811	-0.3847	-0.0667	7.74	0.0054
**x8**	yes		1	0.1344	0.0788	-0.0200	0.2888	2.91	0.0879
**x9**	yes		1	-0.0515	0.2830	-0.6061	0.5031	0.03	0.8556

The GENMOD Procedure

Analysis Of Maximum Likelihood Parameter Estimates									
Parameter			DF	Estimate	Standard Error	Wald 95% Confidence Limits		Wald Chi-Square	Pr > ChiSq
x10	yes		1	-0.0062	0.0869	-0.1766	0.1642	0.01	0.9434
x11	yes		1	0.1504	0.2195	-0.2800	0.5807	0.47	0.4935
x12	yes		1	0.2505	0.2837	-0.3055	0.8064	0.78	0.3772
x13	yes		1	-0.2397	0.1757	-0.5840	0.1047	1.86	0.1725
x14	yes		1	0.0963	0.2701	-0.4331	0.6257	0.13	0.7214
upset	do not want to answer		1	-0.2067	0.3304	-0.8542	0.4408	0.39	0.5316
upset	no		1	0.1694	0.0602	0.0514	0.2874	7.92	0.0049
upset	yes		1	0.2142	0.0960	0.0261	0.4023	4.98	0.0256
dur			1	0.0050	0.0009	0.0032	0.0067	31.34	<.0001
support_family			1	0.0011	0.0010	-0.0008	0.0030	1.33	0.2483
support_doctor			1	0.0016	0.0010	-0.0003	0.0035	2.80	0.0941
support_fedgovt			1	0.0001	0.0013	-0.0024	0.0026	0.01	0.9409
support_city			1	0.0031	0.0014	0.0004	0.0059	4.89	0.0270
support_deptpubhealt			1	0.0017	0.0013	-0.0008	0.0043	1.76	0.1851
support_faithcommuni			1	0.0020	0.0010	0.0000	0.0039	4.03	0.0446
support_socialservic			1	0.0026	0.0013	0.0000	0.0052	3.88	0.0490
support_neighbors			1	-0.0001	0.0010	-0.0020	0.0018	0.01	0.9141
support_other			1	0.0003	0.0012	-0.0021	0.0026	0.04	0.8323
infected	yes		1	0.1081	0.2350	-0.3524	0.5687	0.21	0.6454
feltunwell	missing		1	-0.9311	0.4219	-1.7580	-0.1042	4.87	0.0273
feltunwell	yes		1	0.0546	0.0573	-0.0577	0.1669	0.91	0.3404
compare_others	poor or fair		1	0.1347	0.0943	-0.0501	0.3196	2.04	0.1532
ratehealth	poor or fair		1	0.0827	0.1261	-0.1644	0.3298	0.43	0.5119
gender	Male		1	-0.1764	0.0563	-0.2867	-0.0660	9.81	0.0017
gender	Other		1	-0.0041	0.2105	-0.4166	0.4085	0.00	0.9846

Fully adjusted models of FEAR OF INFECTION with gender*theme interaction

The GENMOD Procedure

Analysis Of Maximum Likelihood Parameter Estimates									
**Parameter**			**DF**	**Estimate**	**Standard Error**	**Wald 95% Confidence Limits**		**Wald Chi-Square**	**Pr > ChiSq**
**race**	black		1	0.0643	0.0960	-0.1240	0.2525	0.45	0.5034
**race**	other race		1	-0.0637	0.0930	-0.2460	0.1186	0.47	0.4935
**age_cat**	35-54		1	0.0844	0.0742	-0.0612	0.2299	1.29	0.2559
**age_cat**	greater than 55		1	-0.0492	0.0800	-0.2060	0.1076	0.38	0.5384
**inc**	40,000 - $99,999		1	0.0825	0.0589	-0.0329	0.1979	1.96	0.1613
**inc**	less than $40,000		1	0.1620	0.0764	0.0121	0.3118	4.49	0.0341
**inc**	missing		1	0.0504	0.1525	-0.2484	0.3492	0.11	0.7410
**edu**	missing		1	-0.5116	0.3286	-1.1555	0.1324	2.42	0.1195
**edu**	no college degree		1	0.0699	0.0702	-0.0677	0.2074	0.99	0.3194
**marital_status**	married or living as married		1	0.2820	0.0617	0.1611	0.4028	20.91	<.0001
**marital_status**	missing		1	0.2568	0.3182	-0.3669	0.8804	0.65	0.4197
**marital_status**	widowed, divorced		1	0.0684	0.0860	-0.1002	0.2370	0.63	0.4266
**kids**	missing		1	-0.1279	0.3370	-0.7885	0.5327	0.14	0.7043
**kids**	yes		1	0.0502	0.0691	-0.0852	0.1857	0.53	0.4672
**hadajob**	no		1	-0.0459	0.0636	-0.1706	0.0788	0.52	0.4703
**Scale**			1	0.8333	0.0164	0.8018	0.8660		

Note: The scale parameter was estimated by maximum likelihood.

Fully adjusted models of FEAR OF INFECTION with gender*theme interaction

The GENMOD Procedure

Model Information	
**Data Set**	WORK.SCORES
**Distribution**	Normal
**Link Function**	Identity
**Dependent Variable**	Factor3

**Number of Observations Read**	1293
**Number of Observations Used**	1293

Class Level Information				
**Class**	**Value**	**Design Variables**		
**gender**	**Female**	0	0	
	**Male**	1	0	
	**Other**	0	1	
**race**	**black**	1	0	
	**other race**	0	1	
	**white**	0	0	
**age_cat**	**35-54**	1	0	
	**greater than 55**	0	1	
	**less than 35**	0	0	
**inc**	**$100,000 and above**	0	0	0
	**40,000 - $99,999**	1	0	0
	**less than $40,000**	0	1	0
	**missing**	0	0	1
**edu**	**college degree or higher**	0	0	
	**missing**	1	0	
	**no college degree**	0	1	

The GENMOD Procedure

Class Level Information				
Class	Value	Design Variables		
kids	missing	1	0	
	no	0	0	
	yes	0	1	
marital_status	married or living as married	1	0	0
	missing	0	1	0
	single	0	0	0
	widowed, divorced	0	0	1
feltunwell	missing	1	0	
	no	0	0	
	yes	0	1	
compare_others	good to excellent	0		
	poor or fair	1		
ratehealth	good to excellent	0		
	poor or fair	1		
x1	no	0		
	yes	1		
x2	no	0		
	yes	1		
x3	no	0		
	yes	1		
x4	no	0		
	yes	1		
x5	no	0		
	yes	1		

The GENMOD Procedure

Class Level Information				
Class	Value	Design Variables		
x6	no	0		
	yes	1		
x8	no	0		
	yes	1		
x9	no	0		
	yes	1		
x10	no	0		
	yes	1		
x11	no	0		
	yes	1		
x12	no	0		
	yes	1		
x13	no	0		
	yes	1		
x14	no	0		
	yes	1		
upset	do not want to answer	1	0	0
	missing	0	0	0
	no	0	1	0
	yes	0	0	1
hadajob	no	1		
	yes	0		
infected	no	0		
	yes	1		

The GENMOD Procedure

Criteria For Assessing Goodness Of Fit			
**Criterion**	**DF**	**Value**	**Value/DF**
**Deviance**	1243	903.5048	0.7269
**Scaled Deviance**	1243	1293.0000	1.0402
**Pearson Chi-Square**	1243	903.5048	0.7269
**Scaled Pearson X2**	1243	1293.0000	1.0402
**Log Likelihood**		-1602.9567	
**Full Log Likelihood**		-1602.9567	
**AIC (smaller is better)**		3307.9135	
**AICC (smaller is better)**		3312.1874	
**BIC (smaller is better)**		3571.3142	

Algorithm converged.

Analysis Of Maximum Likelihood Parameter Estimates									
**Parameter**			**DF**	**Estimate**	**Standard Error**	**Wald 95% Confidence Limits**		**Wald Chi-Square**	**Pr > ChiSq**
**Intercept**			1	-1.2762	0.1629	-1.5954	-0.9569	61.40	<.0001
**gender*x14**	Male	yes	1	0.3959	0.8886	-1.3457	2.1375	0.20	0.6559
**gender*x14**	Other	yes	0	0.0000	0.0000	0.0000	0.0000	.	.
**x1**	yes		1	0.0335	0.0638	-0.0916	0.1587	0.28	0.5993
**x2**	yes		1	0.1529	0.3224	-0.4789	0.7847	0.22	0.6353
**x3**	yes		1	-0.1288	0.1078	-0.3401	0.0826	1.43	0.2324
**x4**	yes		1	0.0071	0.0924	-0.1739	0.1882	0.01	0.9386
**x5**	yes		1	0.2743	0.0801	0.1173	0.4313	11.72	0.0006
**x6**	yes		1	-0.2276	0.0814	-0.3871	-0.0681	7.82	0.0052
**x8**	yes		1	0.1451	0.0789	-0.0095	0.2998	3.38	0.0659
**x9**	yes		1	-0.0696	0.2837	-0.6256	0.4864	0.06	0.8063

The GENMOD Procedure

Analysis Of Maximum Likelihood Parameter Estimates									
Parameter			DF	Estimate	Standard Error	Wald 95% Confidence Limits		Wald Chi-Square	Pr > ChiSq
**x10**	yes		1	-0.0104	0.0872	-0.1813	0.1605	0.01	0.9049
**x11**	yes		1	0.1521	0.2202	-0.2796	0.5837	0.48	0.4899
**x12**	yes		1	0.2493	0.2845	-0.3083	0.8070	0.77	0.3808
**x13**	yes		1	-0.0728	0.1537	-0.3740	0.2284	0.22	0.6356
**x14**	yes		1	0.0582	0.2859	-0.5021	0.6185	0.04	0.8387
**upset**	do not want to answer		1	0.0759	0.3133	-0.5381	0.6900	0.06	0.8085
**upset**	no		1	0.1679	0.0604	0.0496	0.2863	7.73	0.0054
**upset**	yes		1	0.2095	0.0963	0.0208	0.3982	4.74	0.0295
**dur**			1	0.0051	0.0009	0.0033	0.0068	32.18	<.0001
**support_family**			1	0.0010	0.0010	-0.0009	0.0029	1.03	0.3092
**support_doctor**			1	0.0016	0.0010	-0.0003	0.0035	2.80	0.0942
**support_fedgovt**			1	0.0000	0.0013	-0.0025	0.0026	0.00	0.9735
**support_city**			1	0.0032	0.0014	0.0004	0.0060	5.08	0.0243
**support_deptpubhealt**			1	0.0017	0.0013	-0.0009	0.0043	1.61	0.2041
**support_faithcommuni**			1	0.0020	0.0010	0.0000	0.0039	4.00	0.0455
**support_socialservic**			1	0.0027	0.0013	0.0001	0.0053	4.03	0.0448
**support_neighbors**			1	-0.0002	0.0010	-0.0022	0.0017	0.06	0.8072
**support_other**			1	0.0004	0.0012	-0.0020	0.0027	0.09	0.7618
**infected**	yes		1	0.1052	0.2357	-0.3567	0.5671	0.20	0.6552
**feltunwell**	missing		1	-0.9239	0.4232	-1.7534	-0.0944	4.77	0.0290
**feltunwell**	yes		1	0.0477	0.0574	-0.0649	0.1602	0.69	0.4064
**compare_others**	poor or fair		1	0.1534	0.0944	-0.0315	0.3384	2.64	0.1040
**ratehealth**	poor or fair		1	0.0539	0.1259	-0.1928	0.3006	0.18	0.6684
**gender**	Male		1	-0.1649	0.0560	-0.2746	-0.0552	8.69	0.0032
**gender**	Other		1	0.1019	0.2073	-0.3045	0.5083	0.24	0.6230

Fully adjusted models of FEAR OF INFECTION with gender*theme interaction

The GENMOD Procedure

Analysis Of Maximum Likelihood Parameter Estimates									
Parameter			DF	Estimate	Standard Error	Wald 95% Confidence Limits		Wald Chi-Square	Pr > ChiSq
race	black		1	0.0685	0.0963	-0.1203	0.2573	0.51	0.4771
race	other race		1	-0.0655	0.0933	-0.2483	0.1173	0.49	0.4826
age_cat	35-54		1	0.0798	0.0744	-0.0662	0.2257	1.15	0.2840
age_cat	greater than 55		1	-0.0510	0.0802	-0.2083	0.1062	0.40	0.5249
inc	40,000 - $99,999		1	0.0838	0.0591	-0.0320	0.1996	2.01	0.1563
inc	less than $40,000		1	0.1560	0.0767	0.0057	0.3062	4.14	0.0419
inc	missing		1	0.0548	0.1530	-0.2450	0.3546	0.13	0.7203
edu	missing		1	-0.5479	0.3293	-1.1933	0.0976	2.77	0.0962
edu	no college degree		1	0.0666	0.0704	-0.0713	0.2045	0.90	0.3439
marital_status	married or living as married		1	0.2809	0.0618	0.1597	0.4022	20.63	<.0001
marital_status	missing		1	0.2668	0.3192	-0.3587	0.8924	0.70	0.4032
marital_status	widowed, divorced		1	0.0674	0.0863	-0.1018	0.2365	0.61	0.4350
kids	missing		1	-0.1197	0.3385	-0.7831	0.5437	0.13	0.7236
kids	yes		1	0.0572	0.0693	-0.0785	0.1930	0.68	0.4089
hadajob	no		1	-0.0455	0.0639	-0.1707	0.0797	0.51	0.4765
Scale			1	0.8359	0.0164	0.8043	0.8688		

Note: The scale parameter was estimated by maximum likelihood.
