# Supplementary material for: Experiences of coping with the first wave of COVID-19 epidemic in Philadelphia, PA: Mixed methods analysis of a cross-sectional survey of worries and symptoms of mood disorders
Source: PLoS One. 2021 Oct 4;16(10):e0258213. doi: 10.1371/journal.pone.0258213 (PMC8489717; doi:10.1371/journal.pone.0258213)
Supplement: S2 Table — (PDF) [file pone.0258213.s002.pdf]

Table S2: Precautions reported during survey period

		All (n=1293)									Did not have a job at start of epidemic (n=369)								
			Anxiety				Depression					Anxiety				Depression			
		Total	case				case				Total	case				case			
		N	N	%	mean	sd	N	%	mean	sd	N	N	%	mean	sd	N	%	mean	sd
**Since start of epidemic**																			
Use PPE at home or outdoors																			
Yes		1091	416	38	9.4	4.3	131	12	6.2	3.8	317	95	30	8.6	4.3	42	13	6.1	4.0
No		202	78	39	9.3	4.4	28	14	6.5	3.9	52	14	27	8.7	4.7	7	13	6.4	4.5
Type of PPE																			
Face mask		1085	413	38	9.4	4.3	130	12	6.1	3.8	315	95	30	8.6	4.3	42	13	6.1	4.0
Eye protection		203	83	41	9.6	4.2	28	14	6.5	3.9	77	22	29	8.2	4.2	9	12	5.7	4.1
Gown		9	3	33	9.8	4.7	1	11	6.7	3.7	0								
Gloves		465	192	41	9.5	4.3	54	12	6.1	3.7	146	46	32	8.8	4.0	19	13	6.0	3.7
Other		28	12	43	9.4	3.5	2	18	6.8	3.5	10	4	40	9.1	3.8	2	20	7.8	2.6
**Precautions have you taken to reduce your risk of exposure to COVID-19**																			
Stocked up on essentials at a grocery store or pharmacy	Yes	1086	437	40	9.6	4.2	132	12	6.3	3.8	306	92	30	8.7	4.2	37	12	6.2	4.0
	No	207	57	28	8.4	4.6	27	13	5.7	3.9	63	17	29	7.9	5.0	12	19	5.9	4.5
Filled prescriptions	Yes	687	277	40	9.5	4.2	84	12	6.3	3.8	235	72	31	8.6	4.3	24	10	5.9	3.8
	No	606	217	36	9.2	4.4	75	12	6.1	3.8	134	37	28	8.6	4.5	25	19	6.6	4.5
Made a plan for caring for others who are ill*	Yes	231	107	46	10.3	4.3	38	16	6.9	4	78	29	37	9.4	4.3	11	14	6.8	4.0
	No	1062	387	36	9.2	4.3	121	11	6.1	3.7	291	80	27	8.4	4.4	38	13	6.0	4.1
Made a plan for communicating with family, friends and neighbors	Yes	554	210	38	9.4	4.2	58	10	6.0	3.6	161	42	26	8.4	4.0	13	8	5.6	3.7
	No	739	284	38	9.3	4.4	101	14	6.4	3.9	208	67	32	8.7	4.7	36	17	6.6	4.3
Avoided leaving house for non-essential reasons	Yes	1078	424	39	9.5	4.2	136	13	6.3	3.8	302	94	31	8.7	4.3	43	14	6.3	4.1
	No	215	70	33	8.9	4.6	23	11	5.9	3.8	67	15	22	7.9	4.6	6	9	5.6	3.8
Used social distancing when out in public**	Yes	1209	469	39	9.4	4.2	150	12	6.3	3.7	336	103	31	8.7	4.3	47	14	6.3	4.1
	No	84	25	28	8.2	5.1	9	11	5.6	4.3	33	6	18	7.4	5.2	2	6	5.1	3.8
Avoided crowds and large gatherings	Yes	931	363	39	9.4	4.3	115	12	6.3	3.8	268	84	31	8.7	4.5	36	13	6.2	4.0
	No	362	131	36	9.2	4.2	44	12	6.1	3.8	101	25	25	8.3	4.1	13	13	6.2	4.3
Washed your hands more regularly	Yes	757	297	39	9.4	4.4	89	12	6.2	3.8	183	55	30	8.5	4.4	25	14	6.1	4.2
	No	536	197	37	9.3	4.2	70	13	6.3	3.8	186	54	29	8.6	4.4	24	13	6.3	4.0
Avoided touching your face	Yes	472	189	40	9.4	4.4	56	12	6.0	3.8	96	26	27	8.2	4.4	15	16	5.7	4.4
	No	821	305	37	9.4	4.2	103	13	6.3	3.8	273	83	30	8.7	4.4	34	12	6.3	4.0
Cancelled travel***	Yes	334	135	40	9.7	4.3	49	15	6.4	3.8	88	24	27	8.9	4.3	12	14	6.4	3.7
	No	959	359	37	9.3	4.3	110	11	6.1	3.8	281	85	30	8.5	4.4	37	13	6.1	4.2
Worked from home	Yes	355	149	42	9.8	4.3	53	15	6.5	4	NA								
	No	938	345	37	9.2	4.3	106	11	6.1	3.7									
Other	Yes	147	51	35	9.3	4.1	13	8	6.2	3.5	43	8	19	8.5	3.4	5	12	6.6	4.0
	No	1146	443	39	9.4	4.3	146	13	6.2	3.8	326	101	31	8.6	4.5	44	14	6.1	4.1
None	Yes	2	0	0	5	7.1	0	0	2.5	2.1	1	0	0			0	0		
**In the last week, how often did you do each of the following activities?**																			
Went shopping at the grocery store or drugstore	Daily or more	18	3	17	7.6	4.1	1	6	4.8	3.5	7	2	29	8.3	5.1	1	14	6.3	5.1
	4 or 5 times	33	8	24	6.8	5.1	1	3	4.3	3.5	16	2	13	5.0	4.1	0	0	3.2	2.7
	1 to 3 times	913	343	38	9.3	4.2	119	13	6.2	3.8	243	74	30	8.7	4.4	38	16	6.4	4.2
	Never	328	140	43	9.8	4.4	38	12	6.5	3.8	103	31	30	8.8	4.2	10	10	6.1	3.8
	missing	1	0	0			0	0			0								
Used a delivery service for groceries or for a drugstore	Daily or more	1	1	100			1	100			0								
	4 or 5 times	19	12	63	11.1	3.8	2	11	7.2	3.6	5	2	40	9.4	2.4	1	20	8.6	4.7
	1 to 3 times	419	174	42	9.5	4.2	43	10	6.2	3.5	111	32	32	8.8	4.1	9	8	6.0	3.4
	Never	834	304	36	9.3	4.3	111	13	6.2	3.9	239	29	29	8.5	4.6	37	15	6.2	4.4
	missing	20	3	15	8.6	3.5	2	10	6.3	3.7	14	14	14	8.2	3.4	2	14	5.9	4.1
Used a food delivery service for prepared food e.g., from a restaurant	Daily or more	3	1	33	10.0	5.2	0	0	5.7	2.3	2	1	50	11.5	6.4	0	0	5.0	2.8
	4 or 5 times	34	17	50	10.9	4.3	8	24	7.6	4.1	11	3	27	9.2	4.8	3	27	7.3	4.9
	1 to 3 times	558	238	43	9.7	4.3	67	12	6.3	3.6	113	39	35	8.8	4.6	15	13	6.1	4.2
	Never	680	233	34	9.0	4.2	80	12	6.0	3.9	233	65	28	8.5	4.2	30	13	6.1	4.0
	missing	18	5	28	9.3	5.5	4	22	7.5	4.6	10	1	10	7.8	4.8	1	10	6.3	4.2
Travelled for work	Daily or more	62	24	39	9.4	5.4	6	10	5.6	4.0	NA								
	4 or 5 times	49	23	47	10.4	4.3	10	20	7.2	4.4									
	1 to 3 times	132	54	41	9.4	4.5	13	10	6.0	3.4									
	Never	1037	390	38	9.3	4.2	129	12	6.2	3.8									
	missing	13	3	23	8.2	5.3	1	8	5.1	3.8									
Attended a social function	Daily or more	5	0	0	4.8	3.3	0	0	3.2	4.1	0								
	4 or 5 times	3	2	67	14.0	8.2	0	0	7.7	2.1	2	2	100	18.5	3.5	0	0	8.5	2.1
	1 to 3 times	168	70	42	9.7	4.1	20	12	6.5	3.6	38	13	34	8.9	4.2	4	11	6.6	3.8
	Never	1102	419	38	9.3	4.3	138	13	6.2	3.8	320	29	29	8.6	4.3	45	14	6.2	4.1
	missing	15	3	20	7.2	5.2	1	7	4.7	4.0	9	0	0	4.9	3.7	0	0	3.8	3.3
Went outside	Daily or more	622	217	35	9.0	4.3	58	9	5.8	3.6	181	42	23	7.8	4.2	17	9	5.7	3.9
	4 or 5 times	288	114	40	9.5	3.9	38	13	6.3	3.8	79	30	38	9.2	4.2	12	15	6.3	3.9
	1 to 3 times	347	149	43	9.9	4.4	56	16	6.9	3.8	94	33	35	9.6	4.5	19	20	7.1	4.3
	Never	33	14	42	9.9	5.4	7	21	7.0	5.2	15	4	27	8.3	5.4	1	7	5.7	4.9
	missing	3	0	0	6.0	5.3	0	0	4.7	5.0	0								

* Did not ask if had such people to care for

**i.e., made changes in your everyday routine to minimize close contact with others.

*** did not ask if had travel plans
