# Supplementary material for: Experiences of coping with the first wave of COVID-19 epidemic in Philadelphia, PA: Mixed methods analysis of a cross-sectional survey of worries and symptoms of mood disorders
Source: PLoS One. 2021 Oct 4;16(10):e0258213. doi: 10.1371/journal.pone.0258213 (PMC8489717; doi:10.1371/journal.pone.0258213)
Supplement: Attachment — Submitted filename: Response to reviewers01.pdf [file pone.0258213.s006.pdf]

Response to reviewers

RESPONSE: Thank you for taking the time to review our papers and providing valuable feedback on how to improve it. We have made the following changes in accordance with your suggestions.

Journal Requirements:

COMMENT 1. Please ensure that your manuscript meets PLOS ONE's style requirements, including those for file naming. The PLOS ONE style templates can be found at https://journals.plos.org/plosone/s/file?id=wjVg/PLOSOne_formatting_sample_main_body.pdf and https://journals.plos.org/plosone/s/file?id=ba62/PLOSOne_formatting_sample_title_authors_affiliations.pdf

RESPONSE: We updated style of the manuscript but in case we missed some of its features, please point out specifically what is deficient, and we will gladly remedy.

COMMENT 2. Please ensure that you include a title page within your main document. We do appreciate that you have a title page document uploaded as a separate file, however, as per our author guidelines (http://journals.plos.org/plosone/s/submission-guidelines#loc-title-page) we do require this to be part of the manuscript file itself and not uploaded separately.
Could you therefore please include the title page into the beginning of your manuscript file itself, listing all authors and affiliations.

RESPONSE: Title page is now included in the main document.

COMMENT 3. Please include additional information regarding the survey or questionnaire used in the study and ensure that you have provided sufficient details that others could replicate the analyses. For instance, if you developed a questionnaire as part of this study and it is not under a copyright more restrictive than CC-BY, please include a copy, in both the original language and English, as Supporting Information.

RESPONSE: We provided all the information required to replicate our work. Some aspects of the questionnaire are still copy-right protected and therefore are not able to include it in its entirety. Additional complication is that the actual questionnaire was online, not a paper format, such that exact replication of the instrument is not possible as part of supporting information for the manuscript. However, we provide sufficient information for our peers to replicate our method.

COMMENT Furthermore, please note that all PLOS journals ask authors to adhere to our policies for sharing of data and materials: https://journals.plos.org/plosone/s/data-availability. According to PLOS ONE’s Data Availability policy, we require that the minimal dataset underlying results reported in the submission must be made immediately and freely available at the time of publication. As such, please remove any instances of 'unpublished data' or 'data not shown' in your manuscript and replace these with either the relevant data (in the form of additional figures, tables or descriptive text, as appropriate), a citation to where the data can be found, or remove altogether any statements supported by data not presented in the manuscript.

RESPONSE: We removed all statements of "data not shown" and "unpublished data" and replaced them with supplemental materials, where appropriate.

COMMENT 4. Please provide additional information regarding the eligibility criteria for the study. In particular please clarify whether your study included any minors and If your study included minors under age 18, state whether you obtained consent from parents or guardians. If the need for consent was waived by the ethics committee, please include this information.

RESPONSE: We wrote in the original submission at the start of Recruitment section: "Eligible participants were adults aged 18 years and older and were living in Philadelphia, Pennsylvania" on page 3. Therefore, it is clear that no minors were included in the study. If there are other matters that you wish us to clarify, please ask and we will gladly illuminate them.

COMMENT 5. Please include your full ethics statement in the 'Methods' section of your manuscript file. In your statement, please include the full name of the IRB or ethics committee who approved or waived your study, as well as whether or not you obtained informed written or verbal consent. If consent was waived for your study, please include this information in your statement as well.

RESPONSE: "The study was approved by the Institutional Review Board of Drexel University. Written consent was provided on the first page of the online survey. Only those who accepted the consent can proceed with the survey." Statement inserted at the start of the description of methods, where data collection is described.

COMMENT 6. Please upload a new copy of Figure 1 as the detail is not clear. Please follow the link for more information: https://blogs.plos.org/plos/2019/06/looking-good-tips-for-creating-your-plos-figures-graphics/" https://blogs.plos.org/plos/2019/06/looking-good-tips-for-creating-your-plos-figures-graphics/.

RESPONSE: top and bottom panels of Figure 1 were created as 300 dpi TIFF files and inserted into Word document as per instructions provided. The image files of top and bottom panels were also uploaded separately. There are no instructions on how to label panels of a plot that is one Figure, so we emailed you and obtained instructions on how to handle this, which we followed.

RESPONSES TO SPECIFIC COMMENTS OF THE REVIEWERS.

Reviewer #1: The paper is well presented; the question is valid and of general interest.
A few additional points I think would improve the paper are:

Response: Thank you for your positive appraisal of our work. We undertook to improve the paper using your feedback and are prepared to take further steps to remedy any remaining shortcomings.

REV#1, COMMENT 1. A reference supporting the use of HADS in the general population, as well as the use of the specified cutoffs for significant anxiety and depression.

Response: In the paper, we provided a reference (Bjelland et al., 2002) that reported the validity of the HADS at various cutoffs. We now added a new reference (Crawford et al., 2001) (as reference #15) that supported the use of HADS in the general population in the UK.

Bjelland I, Dahl AA, Haug TT, Neckelmann D. The validity of the Hospital Anxiety and Depression Scale. An updated literature review. J Psychosom Res. 2002;52(2):69-77. Epub 2002/02/08. doi: 10.1016/s0022-3999(01)00296-3. PubMed PMID: 11832252.

Crawford, J. R., Henry, J. D., Crombie, C., & Taylor, E. P. (2001). Normative data for the HADS from a large non-clinical sample. British Journal of Clinical Psychology, 40(4), 429–434. https://doi.org/10.1348/014466501163904

REV#1, COMMENT 2- A description of the sub sample that provided qualitative data

Response: Description of demographics as shown in Table 1 for those who provided qualitative responses is now included in the expanded Table S1 Table. Those who provided qualitative data appeared to be more anxious but otherwise seem largely similar to the overall sample.

REV#1, COMMENT 3- A review of grammar and spelling (for eg: the word 'rely' seems to have been replaced with 'reply' through the manuscript)

Response: We apologize for these errors and carefully reviewed the manuscript to remedy the issues.

Reviewer #2:

REV#2, COMMENT 1. The manuscript is technically sound, with elaborate explanations on study tools used and the methods of analysis performed. The positive points include use of a validated tool (HADS) and also a deductive approach based on lived experiences in qualitative part to frame the themes.

Response: Thank you for overall positive assessment of our work. We undertook further improvements and clarifications, as you recommended.

REV#2, COMMENT 2. Statistical analysis is explained elaborately. However, there are certain points to consider for revision. The normality of the data is not mentioned in the manuscript. This seemed pertinent as the tables present the mean scores (Table 1) while the figure (Figure 1) presents the median and interquartile range.

Response: The continuous depression and anxiety scores follow binomial distribution, which is why we used negative binomial regression and a mixture of descriptive statistics; it is common in the field to present averages and medians of HADS scores, as these would be unbiased for HADS scores. On visual inspection, HADS scores appear to follow truncated normal distributions, but this is deceptive as the variables are not continuous. Factor scores are expected to follow normal distributions, as mentioned in text. (We wrote: “Factors scores were generated and those not directly related to HADS scores were

considered as outcomes in linear regression analyses due to their Gaussian distribution resulting from the additive procedure used to calculate factor scores (confirmed empirically; details not shown)". We do not think that it is important to elaborate more on this point, as it is clearly stated in the paper that normality is not assumed for HADS scores and it is assumed for factor scores. However, if the reviewer insists, we will add histograms of the outcome variables used in regressions. Medians and inter-quartile ranges appeared to be natural descriptors of trends in data that followed distribution of HADS scores: somewhat symmetrical but not normal and not easily 9and meaningfully) transformed to normal.

REV#2, COMMENT 3. Though the missing values are imputed, it is seen that they are only mentioned as a separate entity in the table.

Response: Some of the covariates were not imputed and for these we used "missing" as a separate category, e.g., income. This is a standard practice in epidemiology. We already explained this in the original manuscript and hope that the matter is now clarified.

REV#2, COMMENT 4. Use of rank biserial correlation is appreciable, considering the categorisation of HADS as a binomial outcome. However, since HADS and the other outcomes estimated (Sources of support, worries about COVID-19, "most difficult experiences") are rated on a continuous Likert scale of 0-100, it makes me think why not Pearson correlation co-efficient, especially when these values of correlates and statistical significance also indicate the univariate regression analysis.

Response: Biserial correlation was used not because we categorized HADS (used as continuous) but because themes were coded as present or absent. Clarification added in section on thematic analysis where a rejoinder is made with the quantitative analysis.

REV#2, COMMENT 5. Maximum likelihood factor analysis is not delineated.
In table 3, the standardised scoring co-efficients which have a significant factor loading are emboldened (as stated in the manuscript), but the last variable is also mentioned to be non-significant.

Response: We are unsure what the reviewer requests. Our work is not the right place to explain standard methods, such as ML factor analysis; it is true that one of the variables was not significant. We computed factor scores using procedure embedded in SAS software. Please help us understand what you want us to elaborate on and we shall.

REV#2, COMMENT 6. Oblique VARIMAX rotation is mentioned in the statistical part, but providing a picture of it or a detailed explanation could have been better. Also, the scree plot.

Response: Scree plots, projection plots and all detailed outputs of our factor analysis are now included in Supplemental Materials S4.

REV#2, COMMENT 7. In the multiple regression analysis, interaction of the gender is explained in the text and also in the description of statistical analysis, but not in the table.

Response: These analyses spanned many tables but contributed little to the overall conclusions. Tables shown in main text obviously did not include interaction terms, else these would be shown. Because the reviewer requests to see them, we now include them in (very extensive) Supplemental Materials S5, as promised in the original submission.

REV#2, COMMENT 8. The overall writing of the manuscript is cohesive and comprehensible. There are places at which the language could be improved. There are many places at which words are misspelt, lack of prepositions, wrong usage of tenses, grammatical errors.

Response: We did our best to correct such errors on revision.

Reviewer #3:

Refrain from using language establishing causal relationships between mental state and experience during COVID from data of this cross sectional study. Concluding the study with clarification about such statements is not sufficient as the specific results can be further cited by other researchers. For the same, authors have to be cautious in writing results and giving misinterpretation of research outcomes.

Response: We agree that we cannot establish causal relationships between mental state and experiences during COVID from this cross-sectional study. There are multiple places in our manuscript where we caution the readers about not drawing causal inferences such as:

> "We thus undertook regression analyses mostly to succinctly describe experiences of our participants and are hesitant to draw causal inferences" on page 4
>
> "It is not appropriate to draw causal inference from such correlations and we hope that our findings are not taken to mean that there is benefit in inducing anxiety for the sake of compliance with public health measures" on page 31
>
> "We stress that these behaviours can be both causes and consequences of mood disorders (a matter that our cross-sectional study design cannot address) and therefore are presented merely to give a fuller description of our participants, not to draw inferences" on page 8.

If there are specific instances where the reviewer think we implied causation, we would be happy to remedy.
